# Hemoglobin as a pseudoperoxidase and drug target for oxidative stress-related diseases

**DOI:** 10.1038/s41392-025-02366-w

**Published:** 2025-08-22

**Authors:** Woojin Won, Elijah Hwejin Lee, Lizaveta Gotina, Heejung Chun, Jae-Hun Lee, Mridula Bhalla, Uiyeol Park, Daeun Kim, Tai Young Kim, Ji Won Choi, Yoowon Kim, Sun Jun Park, Jiwoon Lim, Jong-Hyun Park, Hyeon Jeong Kim, Jun Young Heo, Woosuk Chung, Myung Jin Oh, Hyun Joo An, Junghee Lee, Soo-Jin Oh, Hoon Ryu, Ae Nim Pae, Ki Duk Park, C. Justin Lee

**Affiliations:** 1 Center for Cognition and Sociality, Institute for Basic Science (IBS), Daejeon, Republic of Korea; 2 Center for Brain Disorders, Brain Science Institute, Korea Institute of Science and Technology (KIST), Seoul, Republic of Korea; 3 Division of Bio-Medical Science & Technology, KIST School, University of Science and Technology, Seoul, Republic of Korea; 4 College of Pharmacy, Yonsei-SL Bigen Research Institute, Yonsei University, Incheon, Korea; 5 IBS School, University of Science and Technology (UST), Daejeon, South Korea; 6 Department of Biochemistry, Chungnam National University College of Medicine, Daejeon, Republic of Korea; 7 System Network Inflammation Control Research Center, Chungnam National University College of Medicine, Daejeon, Republic of Korea; 8 Department of Anesthesiology and Pain Medicine, Chungnam National University Hospital, Chungnam National University School of Medicine, Daejeon, Republic of Korea; 9 Graduate School of Analytical Science & Technology, Chungnam National University, Daejeon, Republic of Korea; 10 VA Boston Healthcare System, U.S. Department of Veteran Affairs, Boston, MA USA; 11 Boston University Alzheimer’s Disease Research Center and Department of Neurology, Boston University Chobanian & Avedisian School of Medicine, Boston, MA USA

**Keywords:** Diseases of the nervous system, Target identification, Target identification, Molecular neuroscience

## Abstract

Hemoglobin (Hb) is well known for transporting oxygen in the blood, but its role in the brain remains poorly understood. Here, we identified Hb in the cytosol, mitochondria, and nuclei of hippocampal and substantia nigra astrocytes and dopaminergic neurons. As a pseudoperoxidase, Hb decomposes hydrogen peroxide (H_2_O_2_) and mitigates H_2_O_2_-induced oxidative damage. However, in Alzheimer’s disease, Parkinson’s disease, and aging, excessive H_2_O_2_ diminishes astrocytic Hb, perpetuating a vicious cycle of oxidative stress and neurodegeneration. To counter the harmful effects of aberrant H_2_O_2_ production in diseases, we developed KDS12025, a BBB-permeable small molecule that enhances Hb pseudoperoxidase activity 100-fold, even at a low level of Hb. KDS12025 and its analogs achieve this enhancement through its electron-donating amine group, possibly stabilizing the complex between Hb, H_2_O_2_, and KDS12025. KDS12025 reduces astrocytic H_2_O_2_, alleviates astrogliosis, normalizes Hb, and reverts to a virtuous cycle of redox balance, preventing neurodegeneration without altering the oxygen-transport function of Hb. Gene silencing of Hb abrogates the impact of KDS12025 in both culture and animal models, confirming the necessity of Hb for the effects of KDS12025. KDS12025 extends survival and improves motor function even in severe amyotrophic lateral sclerosis and aging. Furthermore, the enrichment of astrocytic Hb in the nucleolus highlights a novel antioxidative mechanism potentially protecting against nuclear oxidative damage. Our findings suggest that Hb is a new therapeutic target for neurodegenerative diseases, with KDS12025 emerging as a first-in-class approach that enhances Hb pseudoperoxidase activity to reduce H_2_O_2_. Increasing Hb pseudoperoxidase activity with KDS12025 mitigates oxidative stress and alleviates neurodegeneration in AD, PD, and ALS patients and increases the degree of aging, with broad applicability for numerous oxidative-stress-driven diseases.

## Introduction

Hemoglobin (Hb) is a well-known heme-containing protein in red blood cells that is primarily recognized for transporting oxygen and carbon dioxide in the bloodstream.^1^ Intriguingly, Hb has also been observed in the brain, but its function remains enigmatic.^2,3^ Brain Hb degradation can exacerbate neurodegenerative diseases by releasing iron and heme, leading to inflammation and the generation of reactive oxygen species (ROS).^1,2,4^ For example, the overexpression of Hb in dopaminergic neurons or higher brain Hb levels in elderly individuals increases the risk of neurodegenerative diseases.^5,6^ This self-damaging chain reaction of Hb leads to the formation of ferryl hemoglobin (Fe^4+^=O), globin radicals, and reactive oxygen species (ROS), which further contribute to oxidative damage.^7^ Paradoxically, emerging evidence suggests that Hb exhibits pseudoperoxidase activity, enabling it to decompose hydrogen peroxide (H_2_O_2_) and potentially mitigate oxidative stress.^8^ In cultured astrocytes, Hb administration is protective and potentially suppresses amyloid β-mediated inflammation and oxidative stress.^9,10^ These observations point to a dual role of Hb in the brain, which can be either deleterious or protective depending on its redox context, subcellular localization, and interaction with cofactors such as H_2_O_2_. However, the weak efficiency of its pseudoperoxidase activity and the unclear cellular and molecular mechanisms underlying the antioxidant effects of Hb have limited its therapeutic potential.

ROS, including H_2_O_2_, superoxide, and nitric oxide, play dual roles in the central nervous system, acting as both physiological signaling molecules and pathological agents. While H_2_O_2_ serves as a second messenger, excessive production of H_2_O_2_ in pathological states disrupts the cellular redox balance and drives oxidative stress-related damage, such as neuronal loss and astrogliosis.^11^ H_2_O_2_ is known to cause a myriad of diseases, including neurodegenerative disorders such as Alzheimer’s disease (AD),^12,13^ Parkinson’s disease (PD),^14^ and amyotrophic lateral sclerosis (ALS).^15^ These pathological effects are largely mediated through oxidative modifications of proteins and nucleic acids, leading to impaired synaptic function, mitochondrial dysfunction, neuronal loss, and activation of pro-inflammatory cascades.^16^ In addition, growing evidence suggests that oxidative stress also plays a contributory role in psychiatric conditions such as depression and schizophrenia.^16^ Beyond the brain, oxidative stress also significantly contributes to aging-associated cellular dysfunction and peripheral inflammatory conditions, including atherosclerosis,^17^ acute pancreatitis,^17^ and rheumatoid arthritis (RA).^18^ Together, these findings underscore the broad impact of oxidative stress across both neurological and systemic diseases, making redox regulation a critical therapeutic target.

Among the cellular players involved, astrocytes have emerged as central regulators of redox dynamics in the brain. Our recent research highlights excessive H_2_O_2_ production by reactive astrocytes, primarily driven by monoamine oxidase B (MAOB),^19^ which is highly enriched in astrocytes, and astrocytes are a key factor in neurodegeneration, contributing to neurodegeneration in AD,^12^ PD,^14^ and inflammation in RA.^18^ Interestingly, reactive astrocytes are also implicated in psychiatric disorders such as posttraumatic stress disorder,^20^ where MAOB plays a critical role not only in aberrant GABA synthesis but also in generating H_2_O_2_, further underscoring the pathological relevance of astrocyte-derived oxidative stress. However, developing effective treatments to regulate H_2_O_2_ is particularly challenging because of its dual role as both a pathological agent and a physiological second messenger.^17,21^ Conventional antioxidants, including radical scavengers, face challenges such as prooxidant effects and nonspecific reactivity, limiting their therapeutic success.^17,22^ Moreover, dietary antioxidants such as curcumin and resveratrol face obstacles in clinical development because of their inconsistent efficacy and unclear mechanisms of action.^22^ These limitations highlight the need for precision-targeted approaches that can modulate redox states in specific cell types and subcellular compartments without disrupting essential physiological signaling. Therefore, there is a desperate need to develop novel antioxidant strategies that indirectly reduce H_2_O_2_ levels without disturbing brain function.

Through our serendipitous finding via a horseradish peroxidase (HRP)-based H_2_O_2_ assay, we elucidate an unprecedented antioxidative mechanism in which Hb functions as an H_2_O_2_-decomposing pseudoperoxidase in the brain.^23^ Unlike classical peroxidases that are specialized enzymes with high catalytic efficiency, Hb’s pseudoperoxidase activity is subtle and has long been overlooked due to its primary role in oxygen transport. However, our discovery reveals that under specific redox conditions, Hb can engage in meaningful detoxification of H_2_O_2_, opening new avenues for therapeutic modulation. This activity is significantly amplified by KDS12025, a blood‒brain barrier (BBB)-permeable small molecule that acts as an electron donor, increasing H_2_O_2_ decomposition without interfering with Hb oxygen transport. Importantly, our studies revealed that H_2_O_2_-induced oxidative stress significantly reduces astrocytic Hb levels in the hippocampus and substantia nigra pars compacta (SNpc), a pathological hallmark of both AD and PD, as well as in the aging hippocampus, which is fully reversed by KDS12025 treatment. Even at very low doses, KDS12025 shows remarkable efficacy in animal models of AD, PD, ALS, aging, and RA, highlighting its potential as a broad-spectrum therapeutic for oxidative stress-related diseases. Its efficacy highlights H_2_O_2_ dysregulation as a shared disease mechanism and validates Hb’s pseudoperoxidase enhancement as a new therapeutic strategy.

## Hemoglobin as a pseudoperoxidase and its chemical enhancement

**Fig. 1:**
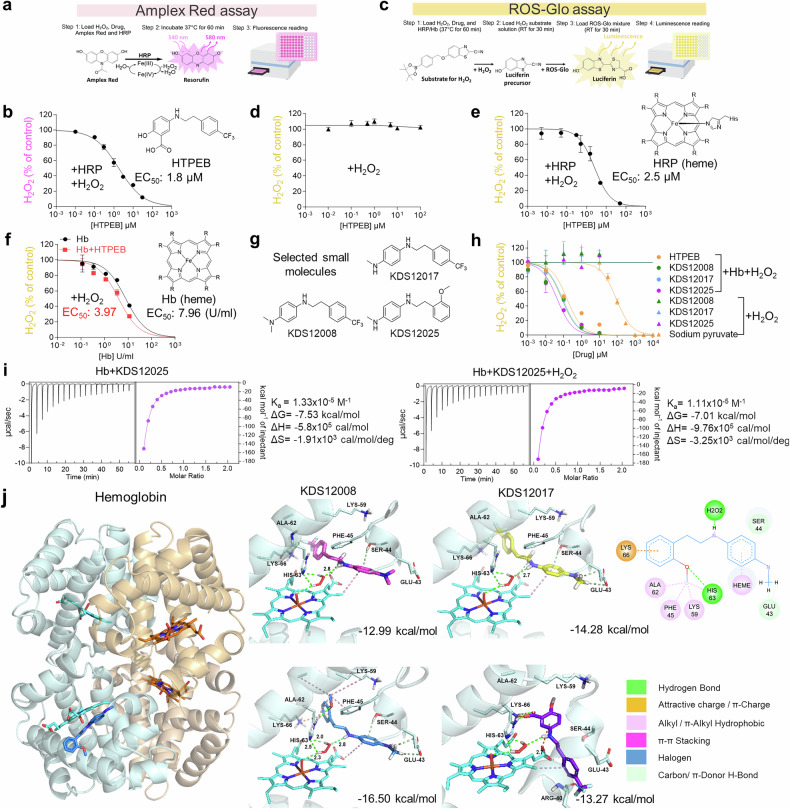
Hemoglobin’s peroxidase-like activity: developing an enhancer for decomposition of H_2_O_2_. **a** Schematic diagram of the HRP-dependent Amplex Red H_2_O_2_ assay. **b** Dose‒response curve for HTPEB in the Amplex Red assay. **c** HRP-independent ROS-Glo H_2_O_2_ assay. **d**, **e** Dose‒response curve for HTPEB in the ROS-Glo assay without or with replenishing HRP. **f** Hb-dependent H_2_O_2_ decomposition in the ROS-Glo assay with (red square) or without (black circle) HTPEB. **g** Chemical structure of KDS derivatives (KDS12008, KDS12017, and KDS12025) retaining the essential *N*-phenethylaniline core with an electron-donating group. **h** Dose‒response curve for KDS derivatives in the presence (circle) or absence (triangle) of Hb (EC_50_ with Hb in μM: HTPEB, 0.15; KDS12008, 0.08; KDS12017, 0.09; KDS12025, 0.04). **i** ITC analysis of the binding interaction between Hb (50 μM) and KDS12025 (5 μM), with or without H_2_O_2_. **j** (left) General view of KDS12025 binding to Hb as proposed by docking simulations. The protein unit color codes are as follows: Hbɑ—wheat and Hbβ—cyan. Heme cofactors and KDS12025 are represented in stick form. (middle) Predicted binding modes of selected active compounds KDS12008, KDS12017, KDS12025, and HTPEB with Hbβ and H_2_O_2_. In ligand structures, polar and interacting nonpolar hydrogens are explicitly shown. The distances between H_2_O_2_ and the ligand and protein (Å) are labeled. The binding energies (ΔG_bind_) are labeled in the bottom right corner of each panel. (right) 2D interaction map of KDS12025 binding to Hbβ. Dose‒response curves and EC_50_ values were determined via GraphPad Prism software. The data are presented as the means ± s.e.m

Previously, we utilized 2-hydroxy-5-[2-(4-trifluoromethyl-phenyl)-ethylamino]-benzoic acid (HTPEB)^12,24^ as a putative H_2_O_2_ scavenger in an animal model of AD, but its cellular and molecular mechanism was unclear. Our previous results from the HRP-based H_2_O_2_ detection assay suggested that HTPEB enhances the peroxidase activity of HRP,^12^ paving the way for the development of a new peroxidase enhancer in the field of neurodegenerative diseases. To determine the detailed molecular mechanism of HTPEB enhancement and to test whether Hb exhibits H_2_O_2_-decomposing activity, we used a series of H_2_O_2_ assays with Amplex Red or ROS-Glo (Fig. 1a, b). HTPEB showed H_2_O_2_ scavenging activity in the Amplex Red assay (Fig. 1a, c), which includes HRP. The proximal (His170) and distal histidine (His42) residues of HRP interact with the iron in the heme group and H_2_O_2,_ respectively.^25,26^ However, HTPEB showed no activity in the ROS-Glo assay lacking HRP (Fig. 1b, d), indicating that the effect of HTPEB is HRP dependent. Replenishing with HRP restored the H_2_O_2_-decomposing activity of HTPEB in ROS-Glo (Fig. 1e), lowering the EC_50_ and increasing the rate of H_2_O_2_ decomposition (Supplementary Fig. 1a, b). These results indicate that HTPEB is a chemical enhancer of peroxidase activity and requires HRP for this action.

Since HRP is a plant-derived heme-containing peroxidase absent in mammals,^25^ we next substituted HRP with animal-derived proteins similar in structure or function to HRP, such as Hb, catalase (CAT), and glutathione peroxidase (GPx),^27,28^ to determine whether the enhancement of HTPEB activity extends to mammalian peroxidases. Notably, Hb, a heme-containing pseudoperoxidase protein, significantly facilitated H_2_O_2_ decomposition by 2-fold with HTEPB, similar to HRP (Fig. 1f). In contrast, the activity of CAT^28^ did not increase (Supplementary Fig. 1c), possibly due to its inherent maximal catalytic efficiency linked to a tyrosine residue.^29^ GPx, a heme-lacking and selenium-containing peroxidase,^28^ also showed no enhancement in a standard GPx assay (Supplementary Fig. 1d, e). These results suggest that Hb retains peroxidase-like activity that can be chemically enhanced by HTPEB.

## Development of potent H_2_O_2_-decomposing enhancers for Hb pseudoperoxidase activity

Although HTPEB enhances the peroxidase-like activity of Hb, its poor BBB permeability and solubility in aqueous solutions limit its therapeutic potential (Supplementary Table 1). We performed a structure‒function analysis on different functional groups within HTPEB to develop improved enhancers. We found that only mesalazine enhanced the peroxidase-like activity of Hb to a level comparable to that of HTPEB (Supplementary Table 2), suggesting that the electron-donating amine group directly bound to an aromatic ring may be critical for the potency of HTPEB.

To improve the pharmacological properties of Hb-enhancers, we synthesized strategically designed HTEPB derivatives, retaining the essential *N-*phenethylaniline core, by modifying the carboxylic acid and hydroxyl groups with an electron-donating methylamine group (Supplementary Fig. 2, Supplementary Table 3). This led to the greater potency of KDS12017 (EC_50_ = 0.09 µM) and KDS12025 (EC_50_ = 0.04 µM), the latter being ~4-fold more potent than HTPEB (EC_50_ = 0.15 µM) (Fig. 1g, h and Supplementary Fig. 2). Unlike direct H_2_O_2_ scavengers such as sodium pyruvate,^30^ neither KDS12017 nor KDS12025 degrades H_2_O_2_ without Hb (Fig. 1h), confirming their dependence on the peroxidase-like function of Hb. Furthermore, KDS12025 did not affect other peroxidases (Supplementary Fig. 1d) or exhibit significant radical scavenging activity (Supplementary Fig. 1e), emphasizing its specificity. Additionally, KDS12025 showed high bioavailability (87.43%) and significant BBB permeability (67.43 × 10^−6 ^cm/s) (Supplementary Table 4), circumventing the limitations of HTPEB. KINOMEscan and off-target profiling confirmed its selectivity and minimal off-target interactions (Supplementary Fig. 3 and Supplementary Tables 5 and 6). While our assays were performed using human Hb, KDS12025 also exhibited efficacy comparable to that of mouse Hb, indicating that its H_2_O_2_-decomposing activity may be similar across species (Supplementary Fig. 1f).

Next, we conducted isothermal calorimetry to investigate the molecular interaction properties of KDS12025 with Hb and its interaction in the presence of H_2_O_2_. We found that KDS12025 showed a significant binding affinity (ΔG_bind_ = −7.53 kcal/mol), with highly negative enthalpy (ΔH = −5.8 × 10^5 ^cal/mol) and entropy (ΔS = −1.91 kcal/mol/deg) (Fig. 1i), indicating a strongly exothermic reaction and stable binding. When H_2_O_2_ was included in the system, the enthalpy (ΔH = −9.8 × 10^5 ^cal/mol) and entropy (ΔS = −3.25 kcal/mol/deg) were reduced (Fig. 1i), suggesting an increased order within the system, likely due to the formation of a coordination complex between KDS12025, Hb, and H_2_O_2_ (Supplementary Fig. 4). These results demonstrate the favorable thermodynamics of KDS12025 binding to Hb and suggest that it facilitates spatial arrangement to optimize Hb pseudoperoxidase activity by stabilizing its interaction with H_2_O_2_ (Supplementary Fig. 4).

Complementary molecular docking revealed a strong interaction (ΔG_bind_ = −16.5 kcal/mol) between KDS12025 and the Hbβ subunit within a wide cleft near the heme molecule, formed by residues 42–50 (Fig. 1j). Numerous hydrophobic interactions of the methoxy group and a hydrogen bonding network between the *N*1 amine group, histidine H63, and heme point are crucial for KDS12025 (Fig. 1j). HTPEB, KDS12008, and KDS12017 were also bound to this site (ΔG_bind_ values of −13.3, −13.0, and −14.3 kcal/mol, respectively) (Fig. 1j). Consistently, the less potent derivatives in the enzyme assay correlated well with less negative ΔG_bind_ values (Supplementary Table 3 and Supplementary Fig. 5). KDS12025 and HTPEB also fit within the narrow catalytic pocket of HRP (Supplementary Fig. 6), suggesting structural compatibility. These findings demonstrate that the *N*-phenethylaniline core retains the crucial amine group function, potently binds to Hbβ, and enhances Hb peroxidase activity, with KDS12025 being the most effective.

## KDS12025 enhances Hb peroxidase activity

**Fig. 2:**
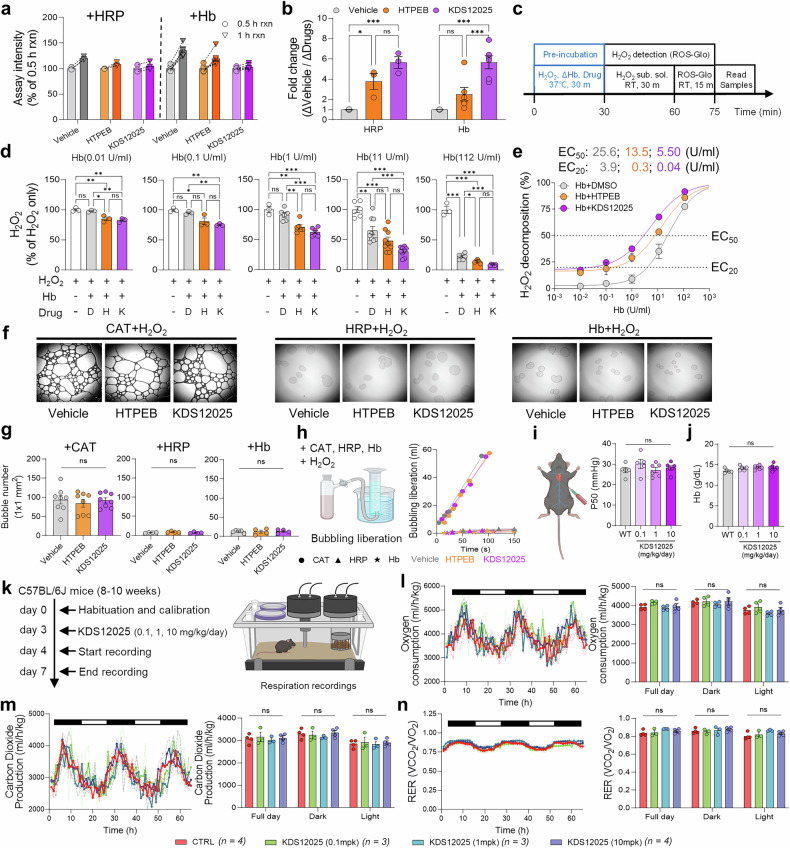
KDS12025 enhances Hb pseudoperoxidase activity. **a** Comparison of H_2_O_2_ decomposition by HRP and Hb after drug treatment (10 μM) for 30 min via the ROS-Glo assay. **b** Fold change (vehicle difference divided by drug difference) in H_2_O_2_ decomposition facilitated by HRP and Hb under drug conditions. **c** Experimental timeline investigating Hb intrinsic peroxidase activity and enhancement by drug preincubation. **d** Hb peroxidase activity and dose-dependent effects of drugs (D, DMSO; H, HTPEB; K, KDS12025) at various Hb concentrations. **e** Dose‒response curve showing the EC_50_ and EC_20_ values for Hb H_2_O_2_ decomposition activity. **f**, **g** Bubble generation observed from CAT, HRP, and Hb in reaction with H_2_O_2_ under a microscope and quantified per 1 × 1 mm^2^ area. **h** Schematic and quantification of bubble liberation volumes measured in the cylinders. **i** Schematic of arterial blood collection by direct cardiac puncture. P50 values (mmHg), calculated from pO_2_ and SO_2_ using the i-STAT analyzer, between vehicle- and KDS12025-treated mice (0.1, 1, or 10 mg/kg, i.p., 24 h). **j** Arterial Hb concentration (g/dL) across groups. **k** Timeline of the PhenoMaster experiments investigating the metabolic effects of KDS12025 at different concentrations (0.1, 1, and 10 mg/kg/day). Measurements of oxygen consumption (**l**), carbon dioxide production (**m**), and the respiratory exchange ratio (RER; L) (**n**) during night (dark) and day (white) cycles, with a summary graph provided. The EC_20_ and EC_50_ values were determined via GraphPad Prism software. The data are presented as the means ± s.e.m. *P < 0.05, **P < 0.01, ***P < 0.001; ns not significant. Additional statistics are provided in Supplementary Table 8

To quantify and compare the enhancement of Hb pseudoperoxidase activity by KDS12025 during a specific period, we measured interval-specific activity during the 30-min reaction window (0.5–1 h) (Fig. 2a). KDS12025 significantly facilitated a 5.1-fold increase in Hb pseudoperoxidase activity per 0.5 h, outperforming HTPEB (2.7-fold), with similar trends observed for HRP (Fig. 2a, b). We then assessed the intrinsic pseudoperoxidase activity of Hb by analyzing its concentration-dependent response (Fig. 2c). At low concentrations, Hb showed no significant pseudoperoxidase activity, whereas preincubation with KDS12025 or HTPEB significantly increased its activity (Fig. 2d). A higher Hb concentration further amplified its activity, with KDS12025 showing greater potency than HTPEB (Fig. 2d). Compared with Hb H_2_O_2_ decomposition in a dose-dependent manner, KDS12025 decreased Hb EC_50_ for H_2_O_2_ decomposition by 4.7-fold and EC_20_ by 98-fold (Fig. 2e). Remarkably, even at extremely low Hb concentrations (0.04 U/ml, 0.03 μg/ml), where intrinsic activity was undetectable, KDS12025 enabled up to 20% H_2_O_2_ decomposition. These findings suggest that KDS12025 has the potential to pharmacologically activate the pseudoperoxidase activity of Hb, enabling effective H_2_O_2_ decomposition events at minimal Hb levels. To further evaluate the structural requirements for Hb enzymatic activation, we fractionated recombinant Hb via size-exclusion chromatography and measured the pseudoperoxidase activity across the eluted fractions. Notably, the enzymatic activity peaked exclusively in the 64 kD tetrameric fraction, whereas the activity was minimal in the 16 kD monomeric molecular weight fractions (Supplementary Fig. 7a). This result suggests that the tetrameric integrity of Hb is essential for KDS12025-mediated H_2_O_2_ decomposition.

It has been previously reported that Hb might have catalase-like activity.^31^ To test the possibility that Hb’s pseudoperoxidase activity involves oxygen liberation, we compared Hb to CAT, which releases oxygen as a byproduct of H_2_O_2_ decomposition. Additionally, we assessed the effect of KDS12025 on this process. While CAT generated strong oxygen release, Hb and HRP produced minimal bubbles (Fig. 2f–h). Neither KDS12025 nor HTPEB increased oxygen release by CAT, which was consistent with the results of the enzyme assay (Supplementary Fig. 1). These results confirm that Hb does not function as a catalase and that KDS12025 does not facilitate oxygen release (Supplementary Fig. 7b, c). Next, to assess whether KDS12025 interferes with the ability of Hb to carry oxygen in the blood, we first measured the arterial oxygen affinity (P50) after KDS12025 treatment. The mice were administered 0.1, 1, or 10 mg/kg/kg/day KDS12025 (drinking *ad libitum)*, and arterial blood was collected from the heart after 24 h. Using the i-STAT1 blood gas analyzer, we measured pO_2_ and SO_2_ and calculated P50 via a standard equation. The P50 values did not differ between the vehicle- and KDS12025-treated groups, indicating that KDS12025 does not affect the oxygen-binding affinity of Hb (Fig. 2i). The total Hb concentration in the blood was also unaffected (Fig. 2j). To further examine whether KDS12025 perturbs whole-body respiratory or metabolic functions, we monitored the respiratory functions of behaving animals via an automated phenotyping system in mouse cages (Fig. 2k). This analysis revealed that 0.1, 1, and 10 mg/kg/day KDS12025 did not affect oxygen consumption, carbon dioxide production, or the respiratory exchange ratio (VCO_2_/O_2_) during the dark and light cycles (Fig. 2l–n). Additionally, energy expenditure, food intake, and drink consumption remained unaffected (Supplementary Fig. 8a–d). Furthermore, to assess possible hemodynamic effects, we monitored cerebral blood perfusion via laser speckle contrast imaging (LSCI). Compared with saline treatment, KDS12025 treatment at 0.1, 1, or 10 mg/kg/day did not affect overall perfusion or vessel diameter (Supplementary Fig. 8e–g), suggesting that it had no effect on cerebral blood pressure. Together, these results imply that KDS12025 potently enhances Hb pseudoperoxidase activity without altering its respiratory function or metabolism in mice.

## KDS12025 effectively reduces AD- and PD-like pathology in vitro

**Fig. 3:**
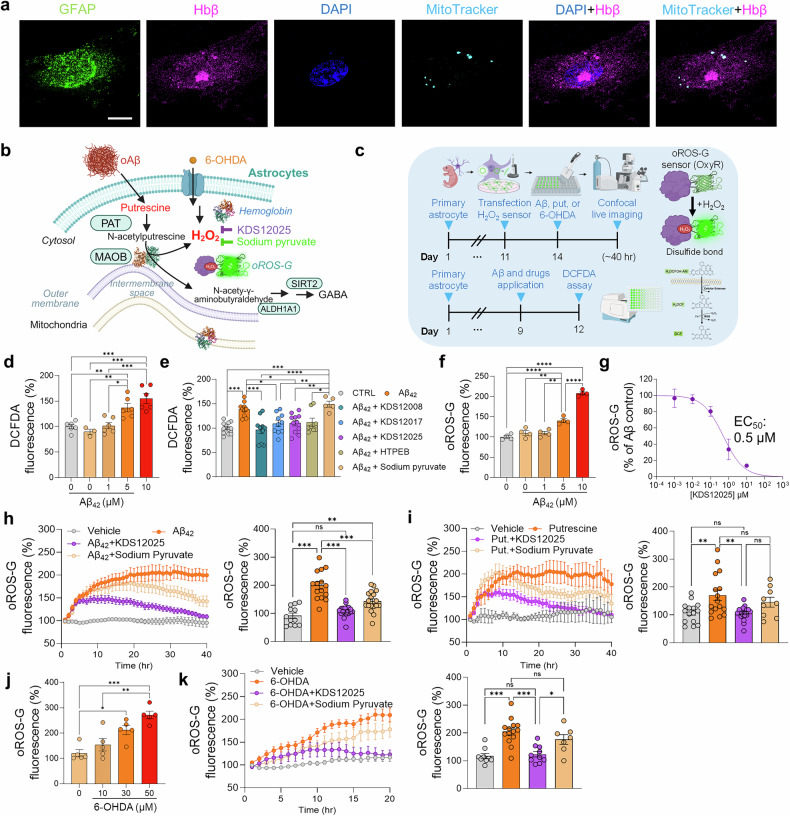
KDS12025 effectively reduces AD- and PD-like pathology in vitro. **a** Representative Lattice-SIM images showing the colocalization of a mitochondrial marker (MitoTracker), Hbβ, and DAPI in cultured astrocytes. **b** Schematic diagram of Aβ, putrescine (a precursor of MAOB-dependent H_2_O_2_ production), or 6-OHDA-induced aberrant H_2_O_2_ in astrocytes. **c** Timeline of 40-h live H_2_O_2_ imaging using an oROS-G probe in Aβ-, putrescine-, or 6-OHDA-incubated cultured hippocampal astrocytes. **d** DCFDA assay in cultured astrocytes treated with various concentrations of Aβ_42_ (0, 1, 5, or 10 μM; n values indicate the number of wells). **e** Measurement of Aβ-induced ROS, including H_2_O_2_ levels, in cultured astrocytes treated with KDS12008 (10 µM), KDS12017 (10 µM), KDS12025 (10 µM), HTPEB (10 µM), or sodium pyruvate (1 mM). **f** oROS-G fluorescence assay in cultured astrocytes expressing oROS-G treated with Aβ_42_ (0, 1, 5, or 10 μM) to assess the level of intracellular H_2_O_2_. **g** Dose‒response curve of KDS12025 in Aβ (5 µM)-treated astrocytes. 40-h continuous H_2_O_2_ imaging using an oROS-G probe following oligomerized Aβ (5 μM, **h**) and putrescine (180 μM, **i**) treatment, with the administration of KDS12025 (10 μM) and sodium pyruvate (1 mM). **j** oROS-G assay in cultured astrocytes treated with 6-OHDA (10, 30, 50 μM). **k** Live-cell H_2_O_2_ imaging via an oROS-G probe following treatment with the oligomerized 6-OHDA (30 μM), KDS12025, and sodium pyruvate. n values in oROS-G indicate the number of cells. The data are presented as the means ± s.e.m. *P < 0.05, **P < 0.01, ***P < 0.001; ns not significant. Additional statistics are provided in Supplementary Table 8

Since primary cultured astrocytes have been suggested to express Hb,^3^ we further investigated the subcellular localization of Hb in cultured hippocampal astrocytes via Lattice SIM superresolution microscopy. We found that Hbβ was localized in the cytosol and MitoTracker-positive mitochondria (Fig. 3a), which is consistent with its proposed role in mitochondrial function.^3^ Notably, Hbβ was detected within the nucleus marker (DAPI)-stained nucleus region but was primarily clustered in DAPI-negative subnuclear regions (Fig. 3a), suggesting possible localization near the nucleolus, although further validation is needed. These results indicate that astrocytic Hbβ is widely distributed across key cellular compartments, including the cytosol, mitochondria, and nucleus.

Next, we tested whether KDS12025 can reduce the aberrant intracellular H_2_O_2_ level in cultured astrocytes (Fig. 3b). Using oROS-G, a H_2_O_2_-specific circularly permuted GFP sensor (oROS-G)^32^ that does not respond to other oxidants (Supplementary Fig. 9) and the general ROS probe DCFDA, we found that oligomeric Aβ induced H_2_O_2_ levels in a dose-dependent manner after 2–3 days of incubation (Fig. 3b–f). These findings suggest that astrocytes play a critical role in generating H_2_O_2_-induced oxidative stress in response to oligomeric Aβ. KDS12025 (10 μM) significantly reduced Aβ-induced H_2_O_2_ with an EC_50_ of 0.5 μM (Fig. 3g), whereas 1 mM sodium pyruvate did not (Fig. 3e), which is consistent with its inability to mitigate menadione-induced ROS in a prior study.^33^ Moreover, long-term live-cell confocal imaging revealed that both Aβ- and putrescine (a precursor of MAOB^34^)-induced aberrant H_2_O_2_ was significantly reduced by KDS12025 treatment within 24 h, whereas 1 mM sodium pyruvate had a minimal effect (Fig. 3h, i). To mimic PD-like conditions, we treated cells with 6-hydroxydopamine (6-OHDA),^35^ a neurotoxin that generates H_2_O_2_ via autoxidation (Fig. 3b), which significantly increased H_2_O_2_ levels in a dose-dependent manner (Fig. 3j). This increase was fully reversed by KDS12025 (Fig. 3k), whereas sodium pyruvate did not (Fig. 3k). These results suggest that KDS12025 effectively mitigates Aβ-, MAOB-, and 6-OHDA-associated H_2_O_2_ levels in cultured astrocytes.

Next, we evaluated the therapeutic potential of three candidate compounds, KDS12008, KDS12017, and KDS12025, in animal models to determine which is the most effective antioxidant. Using the APP/PS1 mouse model, a widely used platform for studying AD,^36^ we treated KDS12008, KDS12017, and KDS12025 (3 mg/kg/day (mpk), 16 times, intraperitoneal injection) to assess their ability to alleviate AD-like symptoms. We found that among the three compounds, KDS12025 most effectively improved memory impairment, as assessed by the passive avoidance test (PAT), and significantly reduced hippocampal reactive astrocyte (GFAP) and microglia (Iba1) levels (Supplementary Fig. 10a–e). To further examine the efficacy of KDS12025, KDS12025 was tested at two doses, 3 and 10 mg/kg/day (7 times, intraperitoneal injection). Even at 3 mg/kg/day, KDS12025 significantly improved memory impairment, restored astrogliosis, and normalized aberrant tonic GABA currents in APP/PS1^36^ (Supplementary Fig. 10f–m). Taken together, these results suggest that KDS12025 is the most effective antioxidant with high potency both in vitro and in vivo.

## The therapeutic effect of KDS12025 in neurodegenerative mouse models, including AD, PD, and ALS

**Fig. 4:**
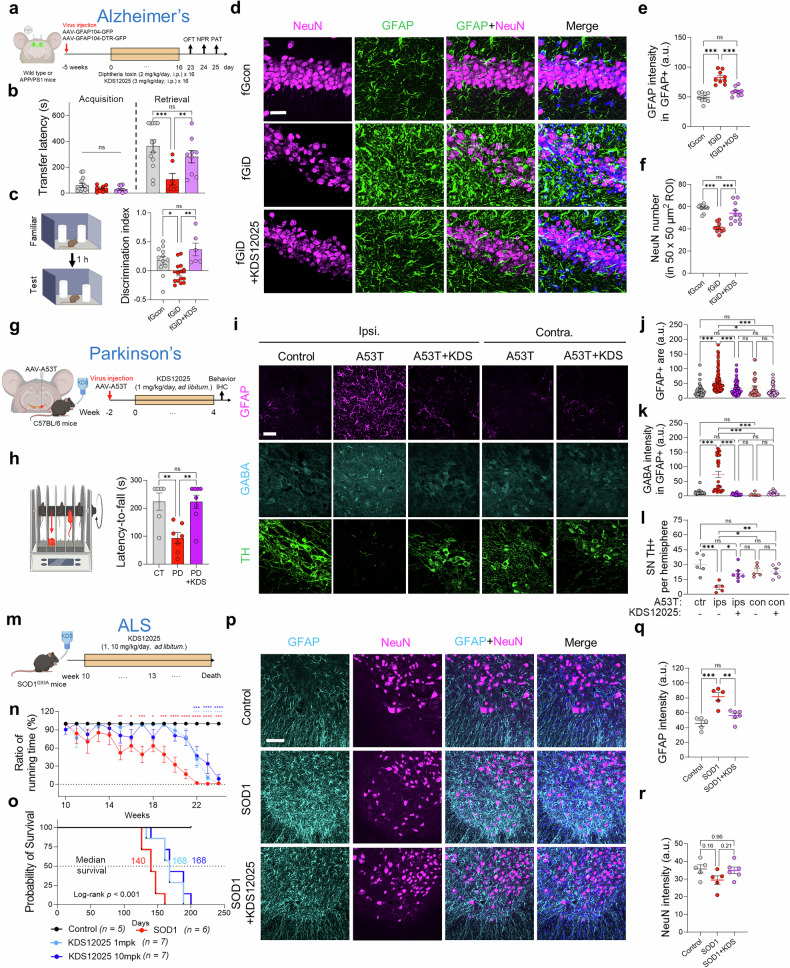
The therapeutic effect of KDS12025 in neurodegenerative mouse models. **a** Schematic timeline of reactive astrocytes in APP/PS1 mice generated via focal expression of DTR (fGiD) in astrocytes via intraperitoneal (i.p.) injection of KDS12025 (3 mg/kg/day) or DT (2 mg/ml) for 16 days. **b** Transfer latency to enter the dark chamber of control, fGiD, and fGiD+KDS mice in the passive avoidance test (PAT). **c** Discrimination indices of control, fGiD, and fGiD+KDS mice in the NPR test. The discrimination index reflects the preference for the relocated object. **d** Representative hippocampal CA1 images stained for NeuN and GFAP in WT, fGiD, and fGiD+KDS mice (*N* = 3 per group; scale bar, 10 μm). **e** Quantification of the mean GFAP intensity in the GFAP-positive area. **f** Neurodegeneration according to the number of NeuN-positive cells (50 × 50 μm^2^). **g** Schematic timeline of the A53T virus-induced PD model and KDS12025 treatment (1 mg/kg/day; water *ad libitum*). **h** Rotarod test diagram and latency-to-fall test results for control, A53T, and A53T + KDS mice. **i** Representative images of the SNpc region with ipsilateral (ipsi.) and contralateral (contra.) Side effects of GFAP, GABA, and TH in control, A53T, and A53T + KDS mice (*N* = 3 for each, scale bar, 10 µm). **j** Quantification of the GFAP-positive area in the SNpc. **k** Mean GABA intensity in GFAP-positive areas in the SNpc. **l** TH-positive neuron counts per hemisphere in the SNpc. **m** Schematic timeline of SOD1^G93A^ mice treated with KDS12025 (1 and 10 mg/kg/day, drinking water *ad libitum*). **n** Ratios of the running times of the control, SOD1, and SOD1 + KDS mice in the rotarod test. **o** Survival probability curves of control, SOD1^G93A^, and SOD1 + KDS mice. **p** Representative images of GFAP and NeuN staining in the ventral spinal cord (L2–L5) of control, SOD1, and SOD1 + KDS mice (N = 3 per group; scale bar, 50 μm). **q** Quantification of the GFAP-positive area in the ventral spinal cord of control, SOD1, and SOD1 + KDS mice. **r** Quantification of NeuN intensity in the ventral spinal cord. The data are presented as the means ± s.e.m. *P < 0.05, **P < 0.01, ***P < 0.001; ****P < 0.0001; ns not significant. Additional statistics are provided in Supplementary Table 8

Reactive astrocytes play a pivotal role in neurodegeneration by promoting oxidative stress and neuronal damage, which are hallmarks of AD, PD, and ALS pathology.^11,12^ To explore the broader therapeutic potential of KDS12025 in neurodegenerative diseases, we conducted studies in well-established animal models of these diseases. For the AD model, we used a previously described animal model of focal DTR (diphtheria receptor)-expressing astrocytes in APP/PS1 mice (fGiD).^12^ This approach addresses the limitations of APP/PS1, which exhibits amyloid pathology but minimal global neurodegeneration,^37^ by inducing severe reactive astrocytes to precipitate neuronal death.^12^ Behavioral assessments, including the PAT and novel place recognition (NPR)^38^ tests, revealed memory and cognitive impairments in fGiD mice, which were significantly reversed by KDS12025 (3 mg/kg/day, 16 times, intraperitoneal injection) (Fig. 4a–c). More importantly, severely reactive astrocytes and a decreased number of NeuN-positive neurons (signs of neurodegeneration) in fGiD mice were reversed to control (fGcon) levels by KDS12025 treatment (Fig. 4d–f). In addition, oral administration of KDS12025 (3 mg/kg/day, 2 weeks, drinking *ad libitum*) normalized the levels of astrocytic GABA and the oxidative stress marker 8-OHdG in APP/PS1 to wild-type levels (Supplementary Fig. 11a–d). Even at 0.1 mg/kg/day, KDS12025 reversed memory impairment in the GiD mouse model of AD (Supplementary Fig. 11e). These results indicate that KDS12025 effectively and potently reverses key AD pathological features, including neurodegeneration, astrogliosis, oxidative stress, and memory impairment.

Next, to assess the effectiveness of KDS12025 in other neurodegenerative diseases, such as PD, we employed a previously described human A53T α-synuclein overexpression model of PD,^39,40^ which selectively induces dopaminergic neuronal loss and motor symptoms via progressive degeneration of the substantia nigra pars compacta (SNpc), thus providing a reproducible and pathophysiologically relevant platform for evaluating oxidative stress-targeted interventions. KDS12025 was orally administered (1 mg/kg/day, 4 weeks, drinking *ad libitum*) (Fig. 4g). We observed significant motor impairment in A53T mice, which was fully rescued by KDS12025 (Fig. 4h). Consistent with AD models, KDS12025 reversed tyrosine hydroxylase (TH)-positive dopaminergic neuronal loss, astrogliosis, and abnormally elevated astrocytic GABA levels in the ipsilateral SNpc of A53T mice, restoring these levels to contralateral levels (Fig. 4j–l and Supplementary Fig. 12). Furthermore, ALS, characterized by progressive motor neuronal loss, currently lacks effective therapies that significantly prolong survival. To address this, we examined KDS12025 in SOD1^G93A^ mice (Fig. 4m), a severe ALS model that is widely used for its robust and reproducible motor neuron degeneration with prominent reactive astrocytosis. We found that KDS12025 (1 and 10 mg/kg/day, *ad libitum*) significantly delayed motor impairment onset by more than 7 weeks and extended the median survival from 140 to 168 days (Fig. 4n, o). This is the first-in-class small molecule that significantly extends survival and delays onset simultaneously in this ALS model. To further investigate the histological changes, we performed immunohistochemistry on the ventral spinal cords (lumbar 2–5) of SOD1^G93A^ mice. We found that the intensity of GFAP was significantly greater in SOD1^G93A^ mice than in WT mice and that this increase was significantly reduced by KDS12025 treatment (Fig. 4p–r). In parallel, the NeuN intensity tended to decrease in SOD1^G93A^ mice, and KDS12025 potently restored this signal (Fig. 4p–r). Taken together, these results highlight KDS12025 as a promising broad-spectrum therapeutic for various neurodegenerative diseases.

## Reduced astrocytic Hbβ in neurodegenerative diseases and its reversal by KDS12025

**Fig. 5:**
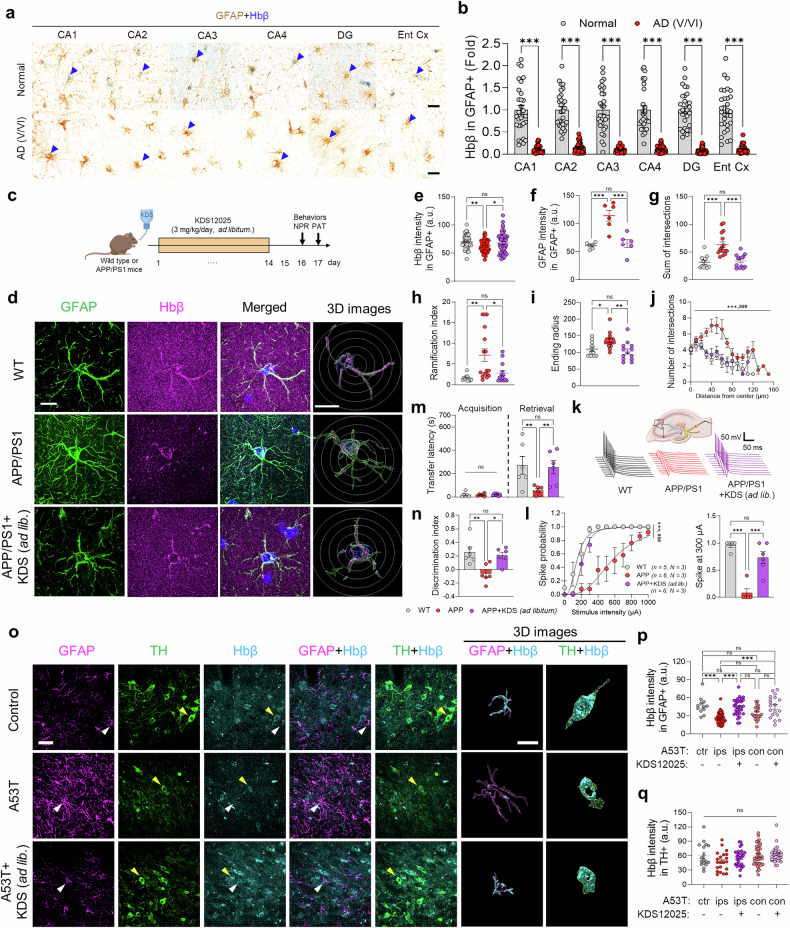
Hbβ is decreased in the hippocampus of AD patients and in the SNpc of PD patients. **a** Representative images of postmortem hippocampal tissues for GFAP and Hbβ in normal subjects and AD patients (*N* = 3 per group; n values indicate cell numbers; scale bar, 20 μm). **b** Quantification of Hbβ intensity in the GFAP-positive area across hippocampal subregions. **c** Schematic timeline of APP/PS1 mice treated with KDS12025 (3 mg/kg/day; drinking *ad libitum*), followed by behavior tests. **d** Representative Lattice-SIM images of the hippocampus for GFAP and Hbβ in WT, APP, and APP + KDS mice (*n* = 3 mice per group). Representative 3D images from Imaris software (green, GFAP; magenta, Hbβ) and Sholl analysis (circles) are shown. Scale bars, 20 μm (main); 10 μm (Imaris). **e** Mean Hbβ intensity in GFAP-positive areas in the hippocampus. **f** Mean GFAP intensity in GFAP-positive areas. **g**–**i** The summary graph shows the sum of intersections, the ramification index, and the ending radius in Sholl analysis. **j** The number of intersections relative to their distance from the center (*** indicates WT vs. APP; ^###^ indicates APP vs. APP + KDS). **k** Schematic diagram of spike probability measurements and representative traces of evoked EPSPs from perforant pathway stimulation in WT, APP, and APP + KDS mice (*n* = 2 mice per group). **l** Spike probability across stimulation intensities (100–1000 μA) (left) and comparison of spike probability at 300 μA (right). **m** Transfer latency to enter the dark chamber in the PAT for WT, APP, and APP + KDS mice (N = 6 mice per group). **n** Discrimination index in the NPR test for WT, APP, and APP + KDS mice. **o** Representative confocal images of the SNpc region with the ipsilateral and contralateral sides for GFAP, GABA, and TH in control, A53T, and A53T + KDS12025 mice. Representative 3D images from Imaris software (magenta, GFAP; green, TH; cyan, Hbβ). Scale bars, 20 μm (left); 10 μm (right). Mean intensity of Hbβ in the GFAP-positive (**p**) and TH-positive areas (**q**) in the SNpc (ctr: control, ipsi: ipsilateral, con: contralateral). Data are presented as the means ± s.e.m. *P < 0.05, **P < 0.01, ***,^###^P < 0.001; ns not significant. Additional statistics are provided in Supplementary Table 8

Although KDS12025 has shown strong effectiveness, its connection to brain Hb remains unclear. To assess the relevance of brain Hb and its clinical relevance in AD, we performed immunohistochemistry on postmortem hippocampal tissues from normal subjects and AD patients (Braak stages V and VI), with a focus on Hbβ due to computational modeling results and previous evidence of its significant impact on cognitive impairment.^41^ We detected the prevalent expression of Hbβ in hippocampal astrocytes under normal conditions, but it was significantly reduced in AD patients (Fig. 5a, b and Supplementary Fig. 13a, b). This reduction was corroborated by the publicly available AD patient astrocyte transcriptomic dataset (Supplementary Fig. 13c) and protein analysis in AD mice (Supplementary Fig. 13d), highlighting astrocytic Hbβ as a potential therapeutic target.

In APP/PS1 mice, via superresolution microscopy, we also observed a significant decrease in Hbβ in hippocampal astrocytes compared with that in wild-type mice, with minimal basal Hbβ expression in pyramidal neurons (Fig. 5c–e and Supplementary Fig. 11f). Moreover, astrocytic Hbβ was localized not only in the cytosol but also within the DAPI-stained nucleus, possibly extending into the nucleolus (Fig. 5d). Notably, KDS12025 treatment reversed the increase in astrocytic Hbβ levels observed in wild-type cells (Fig. 5d, e), which was further supported by the similar increase in protein levels observed via western blotting (Supplementary Fig. 13e), implying that H_2_O_2_ negatively regulates astrocytic Hbβ. Restoration of astrocytic Hbβ was accompanied by improvements in hippocampus-dependent memory and normalized synaptic function, as reflected by the spike probability of action potential firing^36^ (Fig. 5f–n). These findings demonstrate a clear link between reduced astrocytic Hbβ and AD pathology, suggesting its pivotal role in oxidative stress regulation.

In the A53T mouse model of PD, we investigated whether Hbβ is expressed in astrocytes and neurons in the SNpc via confocal microscopy. Unlike in the hippocampus, we observed Hbβ expression in both astrocytes and TH-positive neurons in the SNpc (Fig. 5o). Unlike astrocytes, TH-positive neurons presented prominent Hbβ expression in the cytosol but not in the nucleus under superresolution microscopy (Supplementary Fig. 14a). Consistent with the observations in AD, astrocytic Hbβ levels were significantly lower in the ipsilateral SNpc of A53T mice than in the contralateral SNpc and control SNpc (Fig. 5o–q and Supplementary Fig. 14b), suggesting its link to oxidative stress-driven pathology in PD. In contrast, no significant change in the Hbβ levels of TH-positive dopaminergic neurons was detected across the groups (Fig. 5q). Notably, KDS12025 treatment reversed the increase in astrocytic Hbβ levels in the A53T animal model of PD. Similarly, in the SOD1^G93A^ mouse model of ALS, we observed Hbβ expression in both astrocytes and NeuN-positive neurons in the ventral spinal cord (L2–L5) (Supplementary Fig. 15). In SOD1^G93A^ mice, astrocytic and neuronal Hbβ levels were reduced, but KDS12025 treatment restored their expression. Interestingly, the levels slightly exceeded those of the control animals in both cell types, suggesting that KDS12025 effectively mitigated oxidative stress. This increase may imply that a basal level of ROS exists even in healthy spinal cords. This restoration likely reflects the ability of KDS12025 to mitigate oxidative stress in astrocytes, thereby protecting against neurodegeneration. Notably, KDS12025 treatment restored Hbβ expression, even modestly exceeding control levels, suggesting that the compound effectively mitigates oxidative stress in these cells and that a low level of ROS may persist in control tissue.

Taken together, these findings suggest that astrocytic Hbβ depletion in neurodegenerative diseases, including AD, PD, and ALS, represents an H_2_O_2_-driven vicious cycle of neurodegeneration, in which KDS12025 is disrupted by reducing H_2_O_2_ and restoring Hbβ levels.

## Hbβ is necessary for the action of KDS12025

**Fig. 6:**
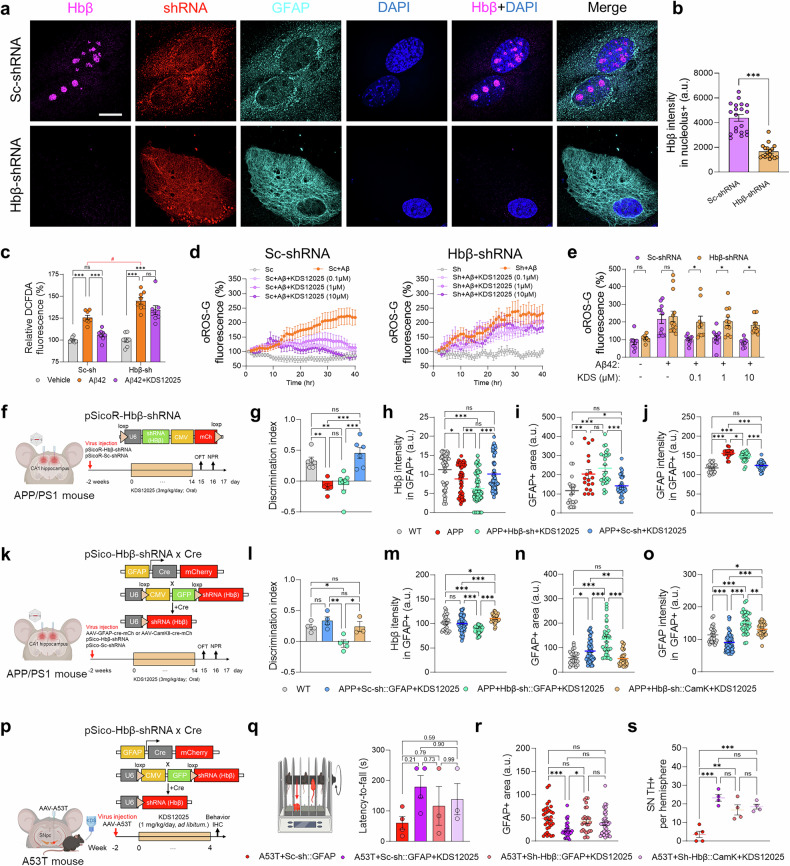
Hbβ is necessary for the action of KDS12025. **a** Representative superresolution microscopy image showing Hbβ expression and gene silencing in cultured astrocytes transfected with the AAV-Hbβ-shRNA or AAV-Sc-shRNA vector. **b** Quantification of Hbβ expression specifically in DAPI-negative nucleolus regions in Sc- and Hbβ-shRNA-transfected astrocytes. **c** Relative levels of ROS, including H_2_O_2_, in Sc- or Hbβ-shRNA-transfected astrocytes treated with vehicle, Aβ, or Aβ + KDS12025 (10 μM). The “#” symbol (red) indicates differences in Aβ-induced H_2_O_2_ between Sc-shRNA and Hbβ-shRNA (*n* values indicate the number of wells). **d** Continuous live-cell H_2_O_2_ imaging using oROS-G in response to Aβ (5 μM) treatment, with KDS12025 (0.1, 1, or 10 μM) in Sc- or Hbβ-shRNA. **e** Comparison of oROS-G intensity between the Sc-shRNA and Hbβ-shRNA groups with KDS treatment (*n* indicates the number of cells). **f** Schematic timeline of the injection of AAV-pSicoR-Hbβ (or Sc)-shRNA into the CA1 hippocampus of APP/PS1 mice. **g** Discrimination index in the NPR test across the groups (*n* = 6 mice per group). **h** Quantification of Hbβ intensity in GFAP-positive areas (*n* indicates cell numbers; 3 mice per group; representative images in Supplementary Fig. 16). Quantification of the GFAP-positive area (**i**) and GFAP intensity (**j**). **k** Schematic timeline of the injection of AAV-pSico-Hbβ (or Sc)-shRNA combined with AAV-GFAP- (or CaMKII-) Cre virus into the CA1 hippocampus of APP/PS1 mice. **l** Discrimination index from the NPR test across the groups (*n* = 4 mice per group, CaMKII; *n* = 3). **m** Quantification of Hbβ intensity in GFAP-positive areas (*n* indicates cell numbers; 3 mice per group; representative images in Supplementary Fig. 17). GFAP-positive area (**n**) and GFAP intensity (**o**) quantification. **p** Schematic timeline of the injection of AAV-pSico-Hbβ (or Sc)-shRNA combined with AAV-GFAP- (or CaMKII-) Cre virus into the SNpc of A53T mice. **q** Schematic diagram of the rotarod test and latency-to-fall test of A53T+Sc::GFAP, A53T+Sc::GFAP + KDS, A53T+Sh::GFAP + KDS, and A53T+Sh::CaMKII+KDS mice. **r**, **s** Quantification of the GFAP-positive area and number of TH-positive neurons per hemisphere in the SNpc (representative images in Supplementary Fig. 18). The data are presented as the means ± s.e.m. *P < 0.05, **P < 0.01, ***P < 0.001; ns not significant. Additional statistics are provided in Supplementary Table 8

To determine whether astrocytic Hbβ is the molecular target of KDS12025, we developed short hairpin RNA (shRNA) to silence Hbβ expression (AAV-pSicoR-Hbβ-shRNA) in primary astrocytes and determine its efficacy (Supplementary Fig. 16a, b). The AAV-pSicoR-Hbβ-shRNA vector was transfected into cultured astrocytes, followed by immunocytochemistry for Hbβ and GFAP to assess the effectiveness of gene silencing (Fig. 6a). We observed a significant reduction in Hbβ levels after shRNA treatment, particularly in the nucleolus, a DAPI-negative nuclear region (Fig. 6b), demonstrating the efficacy of gene silencing. Furthermore, these findings validated the specificity and reliability of the Hbβ antibody, as silenced Hbβ was no longer detected.

Next, we aimed to investigate whether astrocytic Hbβ is necessary for the H_2_O_2_-decomposing activity of KDS12025 in cultured astrocytes via the DCFDA probe (Fig. 6c). In Aβ-treated astrocytes, KDS12025 significantly reduced H_2_O_2_ levels in control pSicoR-scrambled shRNA (Sc-shRNA) astrocytes, but this effect was abolished in Hbβ-silenced astrocytes, indicating the necessity of Hbβ for the action of KDS12025 (Fig. 6c). Interestingly, compared with Sc-shRNA, Hbβ knockdown exacerbated Aβ-induced increases in ROS levels (Fig. 6c), indicating increased vulnerability to oxidative stress in the absence of Hbβ. Consistently, long-term live-cell imaging with the oROS-G sensor demonstrated that the H_2_O_2_-induced effects of KDS12025 (0.1, 1, and 10 μM) were significantly diminished in Hbβ-silenced astrocytes (Fig. 6d, e), indicating the necessity of astrocytic Hbβ-mediated action of KDS12025 in vitro.

To evaluate whether Hbβ is necessary for the effects of KDS12025 in vivo, we injected the AAV-pSicoR-Hbβ-shRNA virus to achieve global Hbβ knockdown in the CA1 hippocampus of APP/PS1 mice (Fig. 6f). This treatment successfully reduced Hbβ in GFAP-positive astrocytes (Fig. 6f and Supplementary Fig. 16c–g), indicating reliable gene silencing in vivo. KDS12025 significantly improved memory impairment and reversed astrogliosis in the Sc-shRNA group, but these effects disappeared in the Hbβ-shRNA group (Fig. 6h–j and Supplementary Fig. 16c–g). Owing to the limitations of the pSicoR system, which generally involves the knockdown of Hbβ across multiple cell types, we employed the AAV-pSico-Hbβ-shRNA virus in combination with the AAV-GFAP-Cre or AAV-CaMKII-Cre virus to selectively knockdown Hbβ in astrocytes or neurons, respectively (Fig. 6k). The therapeutic effects of KDS12025 on cognitive recovery were abolished in the GFAP-Cre group, but the effects of KDS12025 remained in the CaMKII-Cre group (Fig. 6l). Furthermore, immunostaining revealed a significant reduction in astrocytic Hbβ in the GFAP-Cre group, accompanied by the loss of KDS12025-induced recovery of astrogliosis and Hbβ levels (Fig. 6m–o and Supplementary Fig. 17a–d). In contrast, neuronal Hbβ knockdown in the CaMKII-Cre group did not affect the therapeutic outcomes of KDS12025 (Fig. 6m–o and Supplementary Fig. 17a–d), likely because of the minimal levels of neuronal Hbβ. Collectively, these results indicate that hippocampal astrocytic Hbβ, rather than CA1 pyramidal neuronal Hbβ, is necessary for the antioxidative action of KDS12025, effectively alleviating astrogliosis and hippocampus-dependent memory impairment in an animal model of AD.

To investigate whether these findings extend to PD, we focused on the role of astrocytic and neuronal Hbβ in the SNpc of an A53T mouse model of PD. We injected AAV-pSico-Hbβ-shRNA combined with either AAV-GFAP-Cre or AAV-CaMKII-Cre to selectively knock down Hbβ in astrocytes or neurons, respectively (Fig. 6p). KDS12025 treatment effectively restored motor function, reduced astrogliosis, and prevented TH-positive neuronal death in the control Sc-shRNA group (Fig. 6q–s and Supplementary Fig. 18). Interestingly, both the GFAP-Cre and CaMKII-Cre groups exhibited partial recovery of motor function, astrogliosis, and TH-positive neurons when treated with KDS12025 (Fig. 6q–s and Supplementary Fig. 18). These results suggest that both astrocytic and neuronal Hbβ contribute to the therapeutic effects of KDS12025 in the SNpc. Taken together, these findings demonstrate that Hbβ, particularly astrocytic Hbβ in the hippocampus and both astrocytic and neuronal Hbβ in the SNpc, is essential for the therapeutic effects of KDS12025, underscoring its pivotal role in mitigating oxidative stress and neurodegeneration in AD and PD models.

## Potent anti-aging effects of KDS12025

**Fig. 7:**
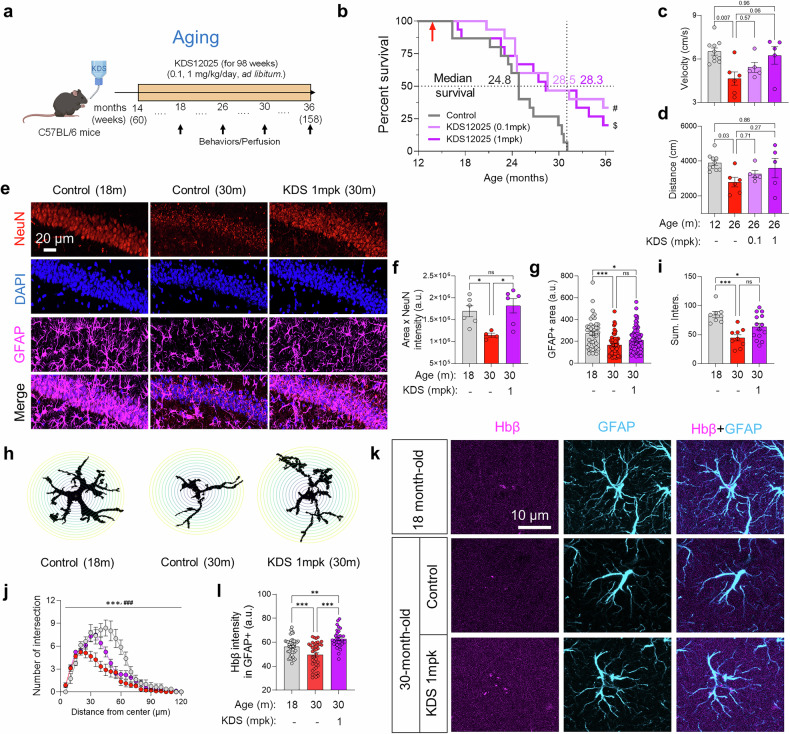
Broad effects of KDS12025. **a** Schematic timeline of aging evaluation and KDS12025 treatment (0.1, 1 mg/kg/day; drinking *ad libitum*) administered from 14 to 36 months (158 weeks, humane endpoint). Arrows indicate the behavior assessments or sacrifice periods. **b** Survival probability curves for the aged control and control+KDS12025 groups (0.1 and 1 mg/kg). The red arrow indicates the starting point of drug administration (*N* = 15 mice per group). **c**, **d** Evaluation of locomotor activity in terms of velocity and distance traveled by young and old (26-month-old) mice treated with KDS12025. **e** Representative hippocampal CA1 images of NeuN and GFAP in control (18- and 30-month-old) and KDS12025-treated (1 mg/kg/day, 30 months old) mice (*N* = 4 mice per group; scale bar, 20 μm). **f** Quantification of NeuN-positive neurons in the CA1 pyramidal layer, calculated as NeuN intensity × neuron number (*n* indicates mouse number). **g** Quantification of the GFAP-positive area (*n* indicates the number of cells). **h** Representative Sholl analysis of astrocytes across groups. Sum of intersections (**i**) and number of intersections (**j**) from Sholl analysis. **k** Representative images of hippocampal astrocytic Hbβ expression in control mice at 18 months and 30 months (control and KDS12025 treatment) (scale bar, 10 μm). **l** Quantification of age-dependent astrocytic Hbβ expression in the hippocampus. Data are presented as the means ± s.e.m. *P < 0.05, **P < 0.01, ***,^###^P < 0.001; ns not significant. Additional statistics are provided in Supplementary Table 8

To further evaluate whether the therapeutic potential of KDS12025 extends to broader H_2_O_2_-related pathologies, we investigated its effects on aging, a condition characterized by H_2_O_2_ accumulation and associated cellular and DNA damage.^42,43^ Fourteen-month-old mice (60 weeks) were treated with KDS12025 (0.1 and 1 mg/kg/day, drinking *ad libitum*) for 22 months and observed until the humane endpoint of 36 months (158 weeks) (Fig. 7a). Notably, median survival was increased by ~14% in both the 0.1 and 1 mg/kg/day KDS12025 groups, with 46.7% (7 out of 15) of the female mice surviving at 31 months when all the control mice had died (Fig. 7b), and no apparent adverse effects were observed throughout the 22-month chronic administration. Concomitantly, a motor deficit, assessed by the open field test, was observed in 26-month-old mice, which was restored in the KDS12025 groups, with locomotor function comparable to that of 12-month-old mice (Fig. 7c, d). Next, to examine whether KDS12025 can mitigate neuronal loss associated with aging, we quantified the severity of neuronal loss by measuring the area and intensity of NeuN- and TH-positive regions in the CA1 hippocampus and the SNpc, respectively. Compared with 18-month-old control mice, 30-month-old mice presented significant neuronal loss (Fig. 7e, f). KDS12025 (1 mg/kg/day) effectively prevented neuronal loss in the hippocampus and preserved TH-positive dopaminergic neurons in the SNpc (Fig. 7e, f and Supplementary Fig. 19d), further supporting its neuroprotective effects. In addition, we observed astrocytic atrophy and aberrant MAOB activity in aging mice (Supplementary Fig. 19a–c). Astrocytic atrophy, characterized by a reduction in cellular processes and disrupted homeostasis,^44^ was measured via Sholl analysis, which revealed a significant reduction in astrocytic arborization in 30-month-old mice compared with 18-month-old mice (Fig. 7e–j). KDS12025 treatment also reverted atrophic astrocytes to those in younger 18-month-old controls (Fig. 7e–j). These results suggest that KDS12025 effectively mitigates aging-associated oxidative stress by preventing neuronal loss, preserving dopaminergic neurons, and reversing astrocytic atrophy.

We further examined astrocytic Hbβ levels in the hippocampus to assess its involvement in aging-related neurodegeneration. Similar to findings in AD and PD patients, we observed a significant reduction in astrocytic Hbβ in 30-month-old mice (Fig. 7k, l), which is consistent with previous findings of decreased Hbβ mRNA levels in aging brains.^45^ Notably, KDS12025 restored astrocytic Hbβ expression to levels exceeding those in 18-month-old mice (Fig. 7k, l), indicating the potent ability of KDS12025 to increase astrocytic Hbβ even in aging conditions. Taken together, these findings suggest that KDS12025 exerts potent anti-aging effects by preventing neuronal loss, reversing atrophic astrocytes, extending lifespan, and enhancing astrocytic Hbβ expression in aging mice, highlighting its potential as a breakthrough therapeutic for combating H_2_O_2_-driven oxidative stress during aging.

To explore the broader potential of KDS12025 against inflammation-induced oxidative stress beyond the brain, we utilized a well-established collagen-induced arthritis (CIA) mouse model of rheumatoid arthritis^18,46^ characterized by systemic inflammation. Remarkably, KDS12025 demonstrated significant efficacy in mitigating RA pathology, as evidenced by a reduction in the CIA score, a clinical measure of joint inflammation severity, and improvements in histological parameters, including synovial hyperplasia and cartilage destruction, as shown via H&E and toluidine blue staining (Supplementary Fig. 20). In addition, KDS12025 at 1 mg/kg restored body weight to normal levels, reflecting systemic recovery alongside reduced inflammation (Supplementary Fig. 20p). Taken together, these findings extend the H_2_O_2_-decomposing activity of KDS12025 beyond the brain, illustrating its capacity to target systemic inflammation-associated oxidative stress and highlighting its potential as a broad-spectrum therapeutic for diverse H_2_O_2_-driven diseases.

## Discussion

In this study, we demonstrated that Hb has peroxidase activity and highlighted the significant decrease in astrocytic Hbβ levels in the brains of both mice and humans with neurodegenerative diseases. We synthesized KDS12025, a small molecule with high BBB permeability and nanomolar potency, which enhances the peroxidase activity of Hb by 100-fold, enabling effective H_2_O_2_ decomposition even at extremely low Hb levels. Importantly, KDS12025 restored astrogliosis, memory, motor function, oxidative stress, and astrocytic Hbβ levels in AD and PD model mice without affecting Hb primary respiratory function. Gene silencing of astrocytic Hbβ recapitulates the necessity of the H_2_O_2_-decomposing effect of KDS12025. KDS12025 also shows efficacy in ALS, aging, and RA, suggesting that it has broader applications for various oxidative stress-associated diseases. Therefore, our findings establish KDS12025 as a groundbreaking strategy to target Hb’s pseudoperoxidase function to effectively treat oxidative stress-driven disorders (Supplementary Fig. 21).

Our study provides compelling evidence that Hb has antioxidative effects through H_2_O_2_-induced pseudoperoxidase activity, not peroxidase- or catalase-like activity.^47^ Unlike real peroxidases, which are structurally optimized for efficient substrate binding and electron transfer, the activity of Hb relies on its heme group interacting with H_2_O_2_ in a process akin to the Fenton reaction.^7^ This involves ferrous iron (Fe²⁺) in Hb’s heme, which catalyzes the decomposition of H_2_O_2_. However, this reaction is naturally much slower and less efficient because of the primary role of Hb as an oxygen transporter rather than an enzyme. The heme pocket of Hb, particularly in the β subunit, lacks key structural features such as H-bond-donating residues near distal His63, which are essential for stabilizing catalytic intermediates and facilitating proton transfer.^48^ As a result, Hb exhibits limited pseudoperoxidase activity under physiological conditions, offering only modest antioxidative protection. Moreover, this activity can generate ROS, such as hydroxyl radicals, especially under pathological conditions where H_2_O_2_ levels are elevated. In contrast, true peroxidases, such as HRP, contain residues such as Arg38 and distal His42, which are critical for efficient proton transfer during peroxidase activity^26,48^ (Supplementary Fig. 4). In contrast, Hbβ lacks H-bond-donating residues near distal His63,^26^ which may explain the lower peroxidase activity of Hb than that of HRP (Supplementary Fig. 4). Hb’s structural constraints not only reduce its catalytic efficiency but also make it prone to ROS-mediated damage under oxidative stress. The introduction of KDS12025 addresses this limitation by incorporating an aniline group that mimics the missing polar residue, enhancing H_2_O_2_ and H_2_O coordination with the heme iron and facilitating electron transfer. Consistent with this idea, ITC data reveal strongly negative ΔH and ΔS values, indicating an exothermic reaction that increases system order (Fig. 1). This finding supports the formation of a stable coordination complex with KDS12025, improving the thermodynamic and kinetic efficiency of Hb in decomposing H_2_O_2_. Future investigations employing cryoelectron microscopy-based structural analyses and mass spectrometry techniques should clarify the precise molecular interactions among KDS12025, Hb, and H_2_O_2_ in the pseudoperoxidase reaction process.

H_2_O_2_ significantly contributes to neurodegenerative diseases,^12,17,21^ exacerbating protein aggregation and impairing Aβ clearance.^49^ Traditional small-molecule antioxidants have failed to alleviate oxidative stress in vivo because of poor brain compatibility, high dosage requirements, and high reactivity, limiting their clinical use.^17,21^ Hence, KDS12025 is the first-in-class drug candidate that enhances the H_2_O_2_-decomposing pseudoperoxidase activity of Hb without directly scavenging H_2_O_2_, suggesting a new therapeutic approach. It increases Hb’s capacity to decompose H_2_O_2_, enabling up to 20% activity even at concentrations where native Hb alone exhibits negligible pseudoperoxidase function, highlighting KDS12025’s potential to activate Hb’s enzymatic role pharmacologically. This unique feature circumvents the limitations of antioxidants such as sodium pyruvate, a direct H_2_O_2_ scavenger, and N-acetylcysteine (NAC), which indirectly reduce H_2_O_2_ by replenishing glutathione for GPx-mediated detoxification, both of which require high doses for efficacy (250 mg/kg and 200 mg/kg, respectively).^50,51^ In contrast, we observed a potent effect in alleviating AD symptoms with an extremely low dose of 0.1 mg/kg/day, even in the drinking water. In addition to reducing oxidative stress, KDS12025 normalized aberrant astrocytic tonic GABA levels and restored the spike probability in hippocampal neurons, suggesting recovery of neuronal and circuit excitability. This finding highlights that KDS12025 not only protects against redox imbalance but also counteracts astrocyte-derived tonic inhibition, which contributes to cognitive dysfunction. Thus, KDS12025 is a promising effective treatment option for neurodegenerative diseases because it enhances the antioxidative properties of Hb at low doses with minimal side effects.

Our findings also suggest that oxidative stress influences astrocytic Hbβ, as shown in our AD and PD models, similar to reduced brain Hbβ mRNA and protein levels in patients.^41,45,52^ Paradoxically, while Hbβ increases in the cerebrospinal fluid of mild cognitive impairment patients,^53^ acute H_2_O_2_ exposure in astrocytes increases Hbβ mRNA and opens its promoter regions (Supplementary Fig. 17e–g), suggesting its initial antioxidative role. In addition, ATAC-seq analysis revealed that Hbβ promoter accessibility is increased at D1 and subsequently reduced by D3 under chronic H_2_O_2_ exposure, indicating early activation followed by epigenetic suppression during prolonged stress. Moreover, increased accessibility at the Hmox1 locus at D3 (Supplementary Fig. 17f) suggests delayed activation of heme degradation pathways, which may promote breakdown of the heme moiety and destabilize the Hbβ protein. Together, these findings support a dual mechanism of transcriptional repression and posttranslational degradation that depletes astrocytic Hbβ under chronic redox stress.

On the basis of these observations, we hypothesize that excessive H_2_O_2_, if unresolved in time, leads to irreversible severe astrogliosis and chronic oxidative stress that further depletes astrocytic Hbβ, perpetuating a vicious cycle to accelerate AD progression (Supplementary Fig. 21). In other words, once reactive astrocytes become severely reactive and engage in a vicious cycle, the condition may become irreversible, where targeting and removing Aβ plaques might be ineffective, particularly in late-stage AD. These new concepts can provide plausible explanations for the recent setbacks of immunotherapy-based drug candidates that attempt to remove Aβ plaques. In the PD model, Hbβ is also observed in TH-positive neurons.^3^ Although we did not find any changes in Hbβ levels, we observed significant changes in astrocytic Hbβ, suggesting that astrocytic Hbβ possibly plays a more critical role in oxidative stress and neurodegeneration in our experiments. In contrast, KDS12025 can liberate hippocampal and SNpc astrocytes from the vicious cycle to enter a virtuous cycle of recovering astrocytic Hbβ levels and decomposing H_2_O_2,_ which further reduces oxidative stress and accelerates recovery from AD and PD. To more rigorously test this recovery under severe pathological conditions, we utilized a previously established severe reactive astrocyte model, fGiD,^12^ which induces more pronounced neurodegeneration, and KDS12025 remarkably reversed cognitive and neuronal deficits. KDS12025 can counteract the decline in antioxidative functions in neurodegenerative diseases, suggesting a viable therapeutic strategy in which natural defenses are limited.

We observed that astrocytic Hbβ is localized not only in the cytosol and mitochondria but also in the nuclear region, particularly in DAPI-negative areas, suggesting possible enrichment in the nucleolus. The nucleolus is pivotal for ribosome biogenesis, DNA repair, and redox regulation,^54^ processes that are highly sensitive to oxidative stress. While the exact function of Hbβ in this compartment remains unclear, its presence raises the possibility that astrocytic Hbβ may contribute to nuclear redox regulation. This hypothesis is further supported by previous findings that Hb can protect against DNA damage^55^ and that cytoglobin interacts with HMGB2 to preserve nuclear DNA integrity.^56^ Furthermore, Hbβ deficiency in disorders such as sickle cell disease and β-thalassemia is associated with astrocyte activation, oxidative stress, and neurodegeneration, providing additional rationale for investigating its nuclear function.^57,58^ Although further studies are needed to clarify the role of astrocytic Hbβ in the nucleolus, our findings reveal a novel antioxidative mechanism mediated by its therapeutic potential to contribute to nuclear homeostasis and genomic stability and potentially mitigate neurodegenerative damage.

Extracellular free Hb has been traditionally viewed as toxic because of its tendency to elicit inflammation and damage through ferroptosis.^2,59^ However, emerging reports suggest that endogenous Hb can regulate oxidative stress.^9,60^ Our study highlights the antioxidative role of endogenous Hbβ in the hippocampus and SNpc (Figs. 4 and 5) under certain conditions, primarily when modulated by enhancers such as KDS12025. The gene-silencing results suggest that the absence of Hbβ increases vulnerability to H_2_O_2_ (Fig. 6). The unexpected antioxidative role of Hbβ suggests that brain Hbβ is an effective therapeutic target in neurodegenerative diseases to manage oxidative stress potently and selectively. The therapeutic potential of KDS12025 can be further expanded to relieve H_2_O_2_-mediated neurodegeneration and oxidative stress in various diseases, such as ischemic stroke, spinal cord injury, and inflammation, which are associated with a reduction in brain Hb levels.^17^

Furthermore, because Hb is abundant in the blood, systemically administered KDS12025 initially may also act on blood Hb to exert its antioxidative effects. Conditions such as anemia, sickle cell disease, and hypoxia, characterized by impaired Hb functionality or reduced Hb levels, have been linked to increased risk or worse outcomes in AD, PD, and ALS patients, suggesting the potential importance of adequate Hb levels to mitigate oxidative stress. In support of this, erythropoietin, a hematopoietic cytokine that upregulates Hb in red blood cells, has been suggested to exert protective effects in AD and PD^61,62^ and delay disease onset in ALS.^63^ Moreover, our experiments revealed that LPS-induced systemic inflammation significantly increased blood H_2_O_2_ levels and that KDS12025 treatment effectively decreased this inflammation-induced increase in H_2_O_2_ levels (Supplementary Fig. 20q, r). Furthermore, we demonstrated that purified red blood cells from peripheral blood were capable of decomposing H_2_O_2_ in response to KDS12025 in a dose-dependent manner (0.01, 0.1, and 1 μM), further confirming its peripheral activity (Supplementary Fig. 20s, t). While our data strongly support the role of Hb as the primary target, we cannot fully exclude the possibility that KDS12025 may also interact with other heme-containing proteins in peripheral tissues, such as cyclooxygenase. Future investigations should be carried out to examine the efficacy of KDS12025 in treating diseases related to peripheral or systemic H_2_O_2_ dysregulation by targeting Hb in the blood.

In summary, we have delineated the unprecedented antioxidative function of Hb, revealing its potential as a key player in combating oxidative stress. This function, which is significantly enhanced by KDS12025, represents a novel mechanism distinct from traditional antioxidants. The newly developed tools and concepts in this study provide a strong foundation for advancing therapeutic strategies to overcome diverse brain and peripheral diseases whose etiology involves oxidative stress. Moreover, this work lays critical groundwork for future disease-specific investigations and highlights the translational potential of KDS12025 as a first-in-class therapeutic that targets oxidative stress and reactive astrocytes.

## Material and methods

### In vitro H_2_O_2_ assay

Hb (Sigma‒Aldrich, H7379), H_2_O_2_ (Sigma‒Aldrich, H1009), and Amplex Red reagents (Invitrogen, A12222) were obtained for the study. A 4 μM H_2_O_2_ solution was prepared in 50 mM sodium phosphate buffer (pH 7.2). Then, 49 μl of this mixture was mixed with 1 μl of the test compound in DMSO or distilled water. The mixture was combined with an assay mixture of Amplex Red reagent (20 mM stock to 0.1 mM) and HRP (200 U/ml stock to 0.2 U/ml) and added to the wells at 50 μl/well. After a 60-min incubation at 37 °C, resorufin production was quantified via a microplate fluorescence reader (SpectraMax iD5, Molecular Devices) at ex/em = 535/580 nm. The H_2_O_2_-decomposing activity was estimated via the following equation: (sample fluorescence-blank fluorescence)/(control absorbance-blank fluorescence) × 100. For the ROS-Glo assay, the H_2_O_2_ substrate solution and ROS-Glo detection solution were prepared according to the manufacturer’s instructions. 10 μM H_2_O_2_ and drugs were prepared as in the Amplex Red assay, with HRP, Hb, CAT, or GPx added as needed. The mixture was added to the wells at 100 μl/well and incubated for 60 min at 37 °C. H_2_O_2_ substrate solution was added, and the mixture was incubated at room temperature for 30 min. After the H_2_O_2_ substrate solution was added and incubated at room temperature for 30 min, the ROS-Glo detection solution was added, and the mixture was incubated for 15 min. Luminescence was measured via iD5 microplate readers. The H_2_O_2_-decomposing activity was estimated via the following equation: (sample luminescence-blank luminescence)/(control luminescence-blank luminescence) × 100. The U/ml was calculated on the basis of the amount of H_2_O_2_ reduced by Hb during the reaction, using μg/ml and U/mg.

To evaluate the extent of peroxidase function facilitation by HTPEB (1 μM) and KDS12025 (1 μM) in HRP (0.2 U/ml) and Hb (10 μg/ml), we used the ROS-Glo assay over 30 and 60 min. The fold change was calculated by dividing the delta values during the 30-min reaction for the vehicle over drug conditions.

To assess enzyme activity, the amount of H_2_O_2_ consumed over a specific time was quantified alongside a standard curve. For the peroxidase function of Hb, Hb was administered at doses ranging from 0.01 to 112 U/ml and preincubated with H_2_O_2_ and either DMSO, HTPEB (1 μM), or KDS12025 (1 μM) at 37 °C for 30 min. The remaining H_2_O_2_ was measured via the ROS-Glo assay and normalized against conditions without Hb. The dose‒response curve, EC_50,_ and EC_20_ for H_2_O_2_ decomposition were calculated and determined by fitting the data with GraphPad Prism software. The fitting method had no special handling of outliers, and least squares regression with confidence intervals of the parameters was 95%.

### Docking and binding energy calculations

Molecular modeling was performed via BIOVIA Discovery Studio software^64^ at pH 7.4 with standard protocol settings unless otherwise specified. HRP and Hb protein structures were obtained from protein databanks (PDB ID 7ATJ^65^ and 2DN1,^66^ respectively). In both systems, the heme iron Fe(IV)O state was set up, mimicking the HRP Compound II and ferryl Hb catalytic stages. In the HRP catalytic pocket, the crucial crystal water molecule HOH1054^67^ was retained. Conversely, in the Hb catalytic pocket, a hydrogen peroxide molecule was placed 2.6 Å under the porphyrin ring pyrrole and within 2.2 Å of both the oxoiron and distal histidine (H58 and H63 in the ɑ and β chains, respectively). Small-molecule docking was performed via the CDOCKER protocol. In the HRP protein system, compounds KDS12025 and HTPEB were docked into the catalytic binding site, and the robustness of the docking protocol was verified through the successful redocking of ferulic acid within 1.5 Å of the cocrystallized ligand pose. For Hb, multiple binding sites in the Hbα and Hbβ subunits of the Hb tetramer have been explored, guided by previously published Hb-small-molecule docking studies.^68–72^ The ligand binding energy (ΔG_bind_) for the top 5 docking poses was determined via the MM-GBSA method under the generalized bound with molecular volume (GBMV) solvent model. Additionally, in situ ligand minimization was included in the protocol, where all protein residues within 6 Å of the ligand were treated as flexible.

### Binding affinity assay

All ITC experiments were conducted via a MicroCal AutoiTC200 (Malvern Panalytical) at the Korea Basic Science Institute (Ochang, Korea). Binding affinity was measured via the addition of 40 µl of 5 mM KDS12025 (ligand) and 200 µl of 200 μM purified human Hb (protein) (MP Biomedical, 55914) in sodium phosphate buffer (pH 7.2). For each titration experiment, 2 µl of KDS12025 was injected into purified human Hb for 4 s at intervals of 180 s at 25 °C. Overall, 19 injections were performed for each experiment, and the data were analyzed via MicroCal Origin 8.0 software.

### Bubbling liberation

CAT, HRP, and Hb were reacted with H_2_O_2_ to assess oxygen production through liberation through bubbling. The reactions were observed under a microscope, and bubbles in a 1 × 1 mm^2^ area were quantified via ImageJ (NIH) software. The effects of bubbling with DMSO, HTPEB, and KDS12025 were evaluated. To measure the degree of bubbling quantitatively, the reactions were conducted in sealed tubes connected to an inverted cylinder submerged in a water bath to record the time and volume of the bubbles produced. To visualize bubble formation, CAT and Hb were prepared at 10 mg/ml and 0.08 mg/ml concentrations. H_2_O_2_ was added at 10 M and 0.1 M. Vehicle, HTPEB, and KDS12025 were added to assess their effects. A similar procedure was followed for Hb. Bubble formation was visually monitored to evaluate catalytic H_2_O_2_ breakdown and oxygen release.

### Arterial blood collection and P50 calculation

To assess whether KDS12025 affects the oxygen-binding affinity of hemoglobin, C57BL/6J mice were treated with KDS12025 (0.1, 1, or 10 mg/kg/day, *ad libitum* in the drinking water) for 24 h. The mice were placed in a supine position to expose the thoracic area. The mice were anesthetized with 2.5% sevoflurane and placed in the supine position. Mechanical ventilation was provided via a custom-made face mask connected to a ventilator (Ventstar small animal ventilator R415, RWD) set to deliver room air (FiO_2_ = 0.21), with a respiratory rate of 180 breaths per min and a tidal volume of 10 μl/g body weight. Without a surgical incision, a 1 ml syringe was directly inserted into the heart to collect bright red arterial blood. Care was taken to avoid venous sampling, and blood was passively drawn into the syringe under physiological arterial pressure. The collected arterial blood was immediately analyzed via an i-STAT® 1 Analyzer (Abbott) with CG8+ cartridges to measure pO_2_ (mmHg), SO_2_ (%), and hemoglobin concentrations (g/dL). P₅₀ values were calculated via the following equation:

$$\,P50={pO}2\,\times \,{\left(\frac{1-{SO}2}{{SO}2}\right)}^{\frac{1}{n}},{n}=2.711$$

### Cultured primary astrocytes

Primary hippocampal astrocytes were obtained from postnatal day 0‒2 (P0‒2) C57BL/6 mice as previously described.^73^ The hippocampal tissue was dissected free of meninges, minced, and dissociated into a single-cell suspension by trituration. The cells were grown in Dulbecco’s modified Eagle’s medium supplemented with D-glucose (4500 mg/L), L-glutamine, sodium pyruvate (110 mg/L), 10% heat-inactivated horse serum, 10% heat-inactivated fetal bovine serum, and 1000 U/ml penicillin‒streptomycin. The cells were maintained at 37 °C in a humidified atmosphere containing 5% CO_2_.

### Oligomerized Aβ_42_ aggregates

Amyloid-beta (Aβ)_42_ monomers (Abcam; DAEFRHDSGYEVHHQKLVFFAEDVGSNK GAIIGLMVGGVVIA) were dissolved to 1 mM in 1% ammonium hydroxide. Aβ_42_ monomers were further diluted to 100 µM in phosphate-buffered saline (PBS). The mixture was incubated at 4 °C for 24 h and stored at −80 °C for further use as an aggregated form of Aβ_42_ as previously described.^74,75^

### Intracellular ROS and H_2_O_2_ detection

The intracellular ROS levels of astrocytes were determined via 2′,7′- dichlorodihydrofluorescein diacetate (DCFDA; Thermo Fisher). Primary cultured hippocampal astrocytes were seeded into black 96-well plates (Ibidi) at a density of 5 × 10⁴ cells per well. The plates were incubated with oligomerized Aβ_42_ in the presence of KDS12008, KDS12017, KDS12025, HTPEB (10 µM each), and sodium pyruvate (1 mM). These cells were subsequently washed with Ca^2+^ and Mg^2+^-containing PBS, followed by the application of 30 µM DCFDA and incubation at 37 °C for 30 min in a dark incubator. The mean fluorescence intensity of each well was measured via iD5 microplate readers at 485 nm and 530 nm. The fluorescence values were calculated by subtracting the blank readings and normalizing them to those of the control group.

H_2_O_2_ specific, highly sensitive, and fast on-and-off kinetics, a genetically encoded oROS-G plasmid kindly provided by Dr. Andre Berndt. oROS-G was cloned under the GFAP104 promoter and delivered into primary cultured hippocampal astrocytes via the NeoN^TM^ transfection system (Invitrogen). The cells were treated with various concentrations of Aβ (1 to 10 μM) or 6-OHDA (Tocris, 2547; 10 to 50 μM) to induce aberrant H_2_O_2_. For subsequent experiments, 5 μM Aβ and 30 μM 6-OHDA were selected on the basis of initial dose‒response studies. KDS12025 (10 μM) and sodium pyruvate (1 mM) were applied as indicated in each experiment. For live-cell imaging, astrocytes were maintained in a humidified chamber at 37 °C with 5% C during imaging to preserve physiological conditions. Changes in the fluorescence of oROS-G were recorded via an A1 Nikon confocal microscope equipped with a temperature- and humidity-controlled stage. Images were captured every 20 min for up to 40 h (20for the 6-OHDA experiments) via the NIS-Elements software (Nikon, Japan) for time-lapse imaging. The excitation and emission wavelengths for oROS-G fluorescence were set to 485 nm and 538 nm, respectively. Endpoint fluorescence measurements were also collected for KDS12025 dose-dependent experiments. To analyze the fluorescence intensity, individual regions of interest (ROIs) were manually selected for each astrocyte via NIS-Elements software (Nikon, Japan). The fluorescence intensity changes were normalized to the baseline values (ΔF/F0) to quantify the H_2_O_2_ dynamics. To determine the EC_50_ of KDS12025, endpoint fluorescence intensity data from oROS-G experiments were normalized to those of the Aβ+vehicle control group. The normalized data were analyzed via GraphPad Prism software to fit a four-parameter logistic curve, yielding the EC_50_ value for the H_2_O_2_-decomposing activity of KDS12025.

### Mouse husbandry and AD mouse model

All the mice, both male and female, were housed in a temperature- and humidity-controlled environment with a 12-h light/dark cycle. Handling and animal care were performed according to the Institutional Animal Care and Use Committee of the Institute for Basic Science (IBS-2023-006, Daejeon, Korea).

For an animal model of AD, both sexes of 10- to 18-month-old APP/PS1 mice were maintained and used as previously described.^76,77^ To test the effects of novel H_2_O_2_-decomposing enhancers in the AD model, the mice were treated by i.p. injection with KDS12008 (3 mg/kg/day), KDS12017 (3 mg/kg/day), or KDS12025 (3 or 10 mg/kg/day) once per day for 1 or 2 weeks, depending on the experimental protocol. All drugs were dissolved in 200 μl of saline, and the amount of each drug was calculated on the basis of the weight (kg) of the mouse. Moreover, KDS12025 (3 mg/kg/day) was orally administered to the AD model for 2 weeks *ad libitum*.

To address the variability in oral drug intake, we measured individual water consumption for 3 consecutive days prior to drug administration to establish a baseline for each mouse. After introducing KDS12025 into the drinking water, we monitored water intake for 3 days to confirm consistent consumption. As KDS12025 is highly water soluble and palatable, no significant changes in water intake were observed following drug administration. Both sexes of 8- to 10-week-old C57BL/6J mice were used for PhenoMaster (TSD systems, Germany). GFAP-CreERT2/iDTR (GiD) lines were maintained by crossing iDTR transgenic mice with GFAP-CreERT2. All the mice were randomly distributed into different groups of matched ages.

### Human α-synuclein-overexpressing mouse model

Stereotaxic injections were performed to deliver the AAV-CMV-A53T virus or PBS (control) into the right SNpc (AP, −3.2 mm; ML, −1.3 mm; DV, −4.0 mm from the bregma) of the mice. The injections were administered at a rate of 0.2 μl/min, with a total volume of 1 μl. The procedure was conducted under general anesthesia induced by 1% isoflurane.

The mice were habituated to the rotarod at speeds of 5, 10, and 15 rpm. Motor coordination and endurance were tested at 20 rpm for a maximum of 3 min. The latency to fall was recorded during the test to evaluate motor deficits associated with PD and the therapeutic effects of KDS12025.

### Respiration and metabolic analysis

For respiration and metabolic analysis, PhenoMaster (TSE Systems, Germany) was employed. All C57BL/6J mice aged 8–10 weeks underwent initial chamber habituation and gas calibration on day 0. Starting on day 3, the mice were administered KDS12025 dissolved in water at dosages of 0.1, 1, and 10 mg/kg/day (*ad libitum* drinking water). Respiratory and metabolic measurements, including oxygen consumption, carbon dioxide production, and the respiratory exchange ratio (RER, calculated as VCO_2_/VO_2_), as well as food and drink intake and energy expenditure, were conducted from day 4. The recording ends on day 8 with data collection.

### Passive avoidance test

Sufficiently handled mice were ready before the PAT. The mice were placed in a two-compartment (light and dark) shuttle chamber with a shock generator (Ugo Basile, Italy). On the first day, the mice were placed in the light compartment for acquisition. The mice were allowed to explore the light compartment for 60 s, after which the door separating the light and dark compartments was raised to allow the mice to enter the dark compartment. When the mice entered the dark compartment, the separating door was closed immediately, and an electrical foot shock (0.5 mA, 2 s duration) was delivered through the floor grid. The mice were then moved back to the home cage, and the retention trial was conducted 24 h after the acquisition. On the second day, the mice were again placed in the light compartment for the retention trial. After 60 s, the door was raised to allow the mice to enter the dark compartment. The latency to step through the dark compartment before and after the electric shock was recorded for up to 540 s.

### Novel place recognition

Before the novel place recognition (NPR) test, the mice were subjected to sufficient handling and were concurrently habituated to an open field test (OFT) in a square chamber (40 × 40 × 40 cm). After habituation, the mice were placed in the open field with two identical objects positioned in the first and second quadrants of the cage. The mice were allowed to explore the objects freely for 10 min and then returned to their home cages for 1 h. To test spatial recognition memory, one of the two identical objects was placed in a novel place (quadrant), and the mice were re-entered into the open field chamber for 10 min. The discrimination index (DI) was calculated as the percentage of time spent on the novel place over the total time spent on both the novel and the familiar place. Frequencies were quantified via EthoVision XT software (Noldus, Netherlands).

### Virus and diphtheria toxin injection

The mice were deeply anesthetized with vaporized 1% isoflurane and placed into stereotaxic frames (RWD Life Science, China). Following an incision on the midline of the scalp, a hole was drilled into the skull above the hippocampus. AAV-GFAP104-GFP or AAV-GFAP104-DTR-GFP was bilaterally microinjected into the hippocampal CA1 region (AP, −2.0 mm; ML, ±1.5 mm; DV, −1.65 mm from the bregma). Diphtheria toxin (2 mg/ml) was administered for 16 days as previously described.^77^ For shRNA knockdown, AAV-pSicoR-Hbβ-shRNA-mCherry or AAV-pSicoR-Sc-shRNA-mCherry was microinjected into the same coordinate system. A total of 0.8 µl of the virus was injected via a syringe pump (KD Scientific, USA). The injection procedures were implemented before 5 weeks of the experiments. For cell type-specific knockdown, AAV-pSico-Hbβ-shRNA-GFP or AAV-pSico-Sc-shRNA-GFP was combined with either AAV-CaMKII-Cre-mCherry for neuronal targeting or AAV-GFAP-Cre-mCherry for astrocyte targeting. The viruses were bilaterally injected into the hippocampus and substantia nigra via the same stereotaxic coordinates and injection procedures. All viruses used in this study were produced at the Institute for Basic Science virus facility (IBS virus facility, Korea). To investigate the effects of 0.1 mg/kg/day KDS12025, GiD mice were injected with diphtheria toxin (2 mg/ml) daily for 16 days. Concurrently, KDS12025 was administered *ad libitum* (0.1 mg/kg/day) during the same period.

### ALS mouse model and survival analysis

SOD1^G93A^ mice (B6SJL-Tg(SOD1*G93A)1Gur/J) were purchased from Jackson Laboratory (strain number 002726) and used in the ALS mouse model. The mice were divided into four groups: Control, SOD1, and KDS12025 (1 and 10 mg/kg/day). Starting at 10 weeks of age, KDS12025 was administered via *ad libitum drinking* water at doses of 1 mg/kg/day and 10 mg/kg/day. The control and SOD1 groups received standard drinking water. Motor function was evaluated weekly via the rotarod performance test, and the ratio of running time was normalized to that of littermate controls to assess motor coordination and endurance over time. Survival of the mice was monitored for up to 24 weeks, and median survival times were calculated for each group.

### Aging mouse

Aged 1-year-old C57BL/6J animals were purchased from Janvier Labs (France) and quarantined in the IBS Animal Facility for 2 weeks before the experiment. The animals were weighed, and their water consumption (in grams) was monitored for 3 days before the appropriate drug concentration was calculated for each dose group. The animals were randomly assigned to the drug/control and dose groups. Water consumption was observed for 1 week after the drug was introduced to ensure that there was no change in daily intake. The drug concentration (in water) was modified on the basis of changes in animal weight and corresponding water consumption every 3 months. Basal body temperature and body weight were monitored every 2 weeks. The care and handling of the animals were performed in accordance with the guidelines of the Institutional Animal Care and Use Committee of IBS (Daejeon, South Korea).

### Immunohistochemistry

The mice were deeply anesthetized with 2% isoflurane and positioned on the operating table. After the abdomen was opened and the heart was exposed, the rats were perfused with saline, followed by 4% paraformaldehyde (PFA) in 0.1 M PBS. The mice were decapitated, and the brains were excised from the skull. Excised brains were postfixed in 4% PFA overnight at 4 °C and dehydrated in 30% sucrose for 48 h. Coronal hippocampal sections were cut at 30 µm thickness with a cryostat and stored in storage solution. For the ALS experiments, lumbar spinal cords (L2–L5) were also carefully dissected and immediately immersed in 4% PFA for postfixation. The sections were fully washed in 0.1 M PBS and incubated for 1 h in blocking solution (0.3% Triton X-100, 2% donkey and goat serum in 0.1 M PBS). Next, a mixture of primary antibodies in the blocking solution was used for immunostaining on a shaker overnight at 4 °C. The sections were washed in PBS three times. After washing, the sections were incubated with the corresponding fluorescent secondary antibodies for 1 h at RT. DAPI staining was performed by adding a DAPI solution (1:2000) during the second washing step. After the washing step was complete, the sections were mounted on cover slides with mounting medium. A series of fluorescence images were obtained with an LSM900 (Zeiss, Germany) confocal microscope or Lattice SIM Elyra 7 (Zeiss, Germany). The 15–22 μm Z stack images in 1–2 μm steps were processed for image analysis, which was performed via the ImageJ (NIH, US) and ZEN digital imaging for light microscopy blue system (Zeiss, Germany) programs. The primary antibodies used were as follows: chicken anti-GFAP (1:500, Millipore, ab5541), rabbit anti-amyloid beta (1:500, Abcam, ab2539), mouse anti-NeuN (1:200, Millipore, ab377), rabbit anti-Hbβ (1:200, Abcam, ab214049), mouse anti-Hbβ (1:200, Santacruz, sc-21757), mouse anti-MAOB (1:200, Santacruz, sc-515354), mouse anti-8-OhdG (1:500, Santacruz, sc-393871), rabbit anti-TH (1:500, Pelfreez, p40101), and mouse anti-TH (1:200, Sigma, T2928). The following secondary antibodies from Jackson were diluted (1:500) in blocking solution: donkey-anti-chicken 405, donkey-anti-chicken 647, donkey-anti-rabbit 488, donkey-anti-rabbit 594, donkey-anti-rabbit 647, donkey-anti-guinea pig 488, donkey-anti-guinea pig 647, donkey-anti-mouse 594, and donkey-anti-mouse 647. Serial confocal sections immunolabeled with GFAP were compiled into a maximal projection to assess the GFAP signal within the hippocampus (Z-stacked images, 15–22 μm Z stack images in 1–2 μm steps of brain sections). Sholl analysis, performed via the ImageJ plugin, automatically generated concentric circles with 10-μm spacing from the nucleus (soma) to the farthest astrocytic process. This analysis quantified the intersections of GFAP-labeled processes within each circle, calculating the ramification index and ending radius, enabling the evaluation of astrocytic hypertrophy and morphological changes, as previously described.^78^ In the focal GiD mouse model, NeuN-positive CA1 pyramidal neurons were quantified within a 50 × 50 μm^2^ region via ImageJ to determine the number of CA1 pyramidal neurons.

### Immunocytochemistry

Hippocampal astrocytes were cultured on coverslips and incubated with 500 nM MitoTracker (Thermo Fisher, M7514) in prewarmed medium for 20 min at 37 °C to label mitochondria in live cells. After incubation, the cells were washed three times with warm PBS to remove unbound dye and then fixed with 4% paraformaldehyde (PFA) in PBS for 15 min at room temperature. Fixed cells were washed three times with PBS, permeabilized with PBS containing 0.3% Triton X-100 and 4% donkey serum for 1 h at room temperature, and incubated overnight at 4 °C with primary antibodies (chicken anti-GFAP, 1:1500; rabbit anti-Hbβ, 1:500) diluted in blocking solution. The next day, the cells were washed three times with PBS and incubated with secondary antibodies for 2 h at room temperature in the dark. DAPI (1:2000) was added during the second PBS wash for nuclear counterstaining. Coverslips were mounted onto glass slides with fluorescence mounting medium, and images were captured via a Nikon A1R confocal microscope.

For shRNA ICC experiments, hippocampal astrocytes were transfected with AAV-pSicoR-Sc-shRNA or AAV-pSicoR-Hbβ-shRNA vectors via the NeoNTM transfection system and incubated for 7–10 days to allow transgene expression. The cells were fixed with 4% PFA in PBS for 15 min, washed, permeabilized, and blocked as described above. Primary antibodies (chicken anti-GFAP, 1:1500; rabbit anti-Hbβ, 1:500) were applied overnight at 4 °C, followed by incubation with secondary antibodies for 2 h in the dark. DAPI (1:2000) was added to the second PBS wash. Transfected cells (e.g., mCherry-positive astrocytes) were identified, and fluorescence intensities for GFAP and Hbβ were quantified via ImageJ, with regions of interest (ROIs) defined for each astrocyte. Sholl analysis and neuron counting.

### Imaris software

Raw confocal image files were processed via Imaris software (version 9.0.1; Oxford Instruments) to analyze Hbβ expression in astrocytes and neurons from the CA1 region of the hippocampus and the SNpc. For hippocampal astrocytes, the stratum radiatum of the CA1 region was manually reconstructed via the surface reconstruction tool in Imaris. GFAP signals were masked within the reconstructed surface, and a new GFAP channel was created for astrocyte-specific analysis. The surface reconstruction parameters included surface details set to 0.6 μm, the diameter of the largest sphere at 2.0 μm, a manual threshold value minimum of 1000, and diffusion transparency set to 60%. After surface reconstruction, Hbβ signals within the astrocytic surface were quantified via the “mask all” function. Nonspecific background signals and incomplete surfaces were excluded by applying a volume filter with a minimum threshold of 350 μm^3^. DAPI staining was used to confirm the nuclear localization of Hbβ, and regions of interest (ROIs) for DAPI-negative nucleolar regions were manually selected to quantify Hbβ signal intensity specifically within these regions.

For the SNpc, astrocytes and dopaminergic neurons were analyzed separately. The SNpc region was manually reconstructed using anatomical landmarks and TH or GFAP signals. GFAP-positive astrocyte surfaces were reconstructed with surface details set to 0.6 μm, the diameter of the largest sphere at 2.0 μm, a manual threshold value minimum of 800, and diffusion transparency set to 60%. Hbβ signals within the astrocytic surface were quantified via the “mask all” function, and incomplete surfaces and background signals were excluded via a volume filter with a minimum volume threshold of 350 μm^3^. For dopaminergic neurons, TH-positive cells were identified, and surfaces were reconstructed with similar parameters: surface details of 0.6 μm, the diameter of the largest sphere of 2.5 μm, and a manual threshold value of a minimum of 900. Hbβ signal intensity within TH-positive neuron surfaces was quantified, and colocalization analysis was performed to evaluate Hbβ expression in both GFAP-positive astrocytes and TH-positive neurons. Representative 3D reconstructions of GFAP, Hbβ, and TH signals were generated via Imaris, with consistent scale bars and color maps applied across all experimental groups. The data were normalized to those of the control groups to ensure comparability across conditions.

### Electrophysiology

The mice were deeply anesthetized with 3% isoflurane followed by decapitation. The brain was quickly excised from the skull and submerged in a chilled cutting solution that contained 234 mM sucrose, 24 mM NaHCO_3_, 11 mM d(+)-glucose, 10 mM MgSO_4_, 2.5 mM KCl, 12.5 mM NaH_2_PO_4_, and 0.5 mM CaCl_2_. Transverse hippocampi (300 µm thick) were prepared with a vibrating microtome (PRO7N; DSK) and transferred to an artificial cerebrospinal fluid (aCSF) solution that contained 130 mM NaCl, 24 mM NaHCO_3_, 1.25 mM NaH_2_PO_4_, 3.5 mM KCl, 1.5 mM CaCl_2_, 1.5 mM MgCl_2_, and 10 mM d(+)-glucose, pH 7.4. The slices were incubated at RT for at least 1 h before recording. All the solutions were saturated with 95% O_2_ and 5% CO_2_.

For recording of tonic GABA currents, slices were transferred to a recording chamber that was mounted on an upright Zeiss microscope and continuously perfused with aCSF solution. The slice was viewed with a 60× water immersion objective (numerical aperture = 90) with infrared differential interference contrast optics. A cellular CMOS camera (Hamamatsu Photonics, Japan) was used. Cellular morphology was visualized by a CMOS camera (Hamamatsu Photonics, Japan) and Imaging Workbench software (INDEC Biosystems). Whole-cell patch recordings were made from pyramidal neurons located in the DG granule cells of the hippocampus. The holding potential was -70 mV. The pipette resistance was typically 6–8 MΩ, and the pipette was filled with an internal solution that contained (in mM): 135 CsCl; 4 NaCl; 0.5 CaCl_2_; 10 HEPES; 5 EGTA; 2 Mg-ATP; 0.5 Na_2_-GTP; 10 QX-314; pH adjusted to 7.2 with CsOH (278 to 285 mOsmol). The baseline current before the tonic current was measured was recorded with CNQX (20 µM) and D-AP5 (50 µM). The frequency and amplitude of spontaneous inhibitory postsynaptic currents before bicuculline (50 µM) administration were detected and measured via Mini Analysis (Synaptosoft, USA).

Dentate granule cell synaptic responses were elicited at a low frequency of 0.1 Hz by stimulating perforant path fibers. The stimulation was delivered over 100 ms at intensities ranging from 0 to 1000 μA using a constant current isolation unit. A tungsten bipolar electrode was positioned in the molecular layer to stimulate the perforant path fibers. Recordings of the evoked excitatory postsynaptic potentials (EPSPs) were typically 6–8 MΩ, and the pipette was filled with an internal solution that contained the following (in mM): 120 potassium gluconate; 10 KCl; 1 MgCl_2_; 0.5 EGTA; 40 HEPES; pH adjusted to 7.2 with KOH. The spiking probability was determined by the number of spikes generated per total stimulation. Electrical signals were digitized and sampled at 10-ms intervals with the Digidata 1550B data acquisition system and the Multiclamp 700B Amplifier (Molecular Devices, USA) via pClamp software.

### *Hbb*-shRNA vector and reverse transcription‒PCR

The shRNA sequence for mouse *hbb* (NM_001278161.1) was designed with a Broad Institute RNAi designer (UK). The shNRA sequence was 5′-gagcatctgtcagttgttggc-3′ inserted into the pSico and pSicoR systems.^79^ For gene silencing of *hbb*, an adenovirus-associated virus (AAV) carrying Hbβ-shRNA was transfected into primary cultured astrocytes. Four days after infection, total RNA was extracted via an AllPrep RNA kit (Qiagen, Netherlands), and cDNA was synthesized via SuperScript III Reverse Transcriptase (Invitrogen, USA). Gene silencing of *hbb* was tested via reverse transcription‒PCR (5′-gcacctgactgatgctgaga-3′).

### Western blot

Western blotting was conducted following established protocols. The membranes were probed with primary antibodies targeting Hbβ and GAPDH overnight at 4 °C. After three washes in Tris-buffered saline containing 0.05% Tween 20, the blots were incubated with HRP-conjugated secondary antibodies for 2 h at room temperature. Bands were detected via Immobilon Western ECL solution and captured on an Image Station 4000MM.

### Human brain samples

Neuropathological examination of postmortem brain samples from normal subjects and AD patients was performed via procedures previously established by the Boston University Alzheimer’s Disease Center (BUADC). The next of kin provided informed consent for participation and brain donation. Institutional review board approval for ethical permission was obtained through the BUADC center. This study was reviewed by the Institutional Review Board of the Boston University School of Medicine and was approved for exemption because it included only tissues collected from postmortem subjects not classified as human subjects. The study was performed in accordance with institutional regulatory guidelines and principles of human subject protection in the Declaration of Helsinki. The sample information is listed in Supplementary Table 7.

### First staining

Paraffin-embedded human postmortem brain tissues were sectioned in the coronal plane at 10 μm. BLOXALL® Blocking solution (Vector Laboratories, Burlingame, CA, USA) was used to block endogenous alkaline phosphatase. Hippocampal tissue sections were blocked with 2.5% normal horse serum (Vector Laboratories) for 1 h and then incubated with a GFAP antibody (1:400, AB5541, Millipore, Burlington, MA, USA) for 24 h. After being washed three times with PBS, the tissue slides were processed with a Vector ABC Kit (Vector Laboratories, Burlingame, CA, USA). The GFAP immunoreactive signals were developed with DAB chromogen (Thermo Fisher Scientific, Meridian, Rockford, IL, USA).

### Second staining

The tissue slides stained with GFAP were incubated with a hemoglobin beta (Hbβ) antibody (1:50, sc-21757, Santa Cruz) for 24 h. After reacting with secondary antibodies, the sections were incubated with ImmPRESS-AP anti-mouse IgG (alkaline phosphatase) polymer detection reagent (Vector Laboratories: MP-5402) for 2 h at room temperature. A Vector Blue alkaline phosphatase substrate kit (Vector Laboratories: SK-5300) was used to develop the HBβ signals. Double-stained tissue slides were gradually processed back to Histo-clear (HS-200) through an increasing ethanol gradient [70%, 80%, 90%, 95%, and 100% (1 time)] and subsequently mounted. The chromogenic signals of GFAP (brown) and Hbβ (blue) were examined via a light microscope (BX63) (Olympus, Japan) equipped with a high-definition (1920 × 1200 pixels) digital camera (DP74) (Olympus, Japan).

### Quantification and statistical analysis

All analyses were performed in a blinded manner. The number of experimental samples for each group is listed in the figure legends or Supplementary Table 8. The numbers and individual data points refer to individual cells unless otherwise specified in the figure legends. *N* represents the number of patients or animals used for the experiment. At least 3 animals were subjected to immunohistochemistry, and at least 2 animals were subjected to electrophysiology. Data representation and statistical analysis were performed via GraphPad software. ImageJ (NIH), ZEN (Zeiss), and NIS-Elements (Nikon) were used for image analysis. Statistical significance was set at ∗P < 0.05, ∗∗P < 0.01, and ∗∗∗P < 0.001 (or #; unless mentioned otherwise in the figure legends). Detailed statistics are also provided in Supplementary Table 8.

## Supplementary information

Supplementary Materials

## Data Availability

We state that the data supporting the results of this study are available from this manuscript and its supplementary information files.
