# Supplementary material for: Hemoglobin as a pseudoperoxidase and drug target for oxidative stress-related diseases
Source: Signal Transduct Target Ther. 2025 Aug 22;10:270. doi: 10.1038/s41392-025-02366-w (PMC12373846; doi:10.1038/s41392-025-02366-w)

Supplementary Materials for

Hemoglobin as a pseudoperoxidase and drug target for oxidative stress-related diseases

Woojin Won†, Elijah Hwejin Lee†, Lizaveta Gotina, Heejung Chun, Jae-Hun Lee_,_ Mridula Bhalla, Uiyeol Park, Daeun Kim, Tai Young Kim, Ji Won Choi, Yoowon Kim, Sun Jun Park, Jiwoon Lim, Jong-Hyun Park, Hyeon Jeong Kim, Jun Young Heo, Woosuk Chung, Myung Jin Oh, Hyun Joo An, Junghee Lee, Soo-Jin Oh, Hoon Ryu, Ae Nim Pae, Ki Duk Park* and C. Justin Lee*

† These authors contributed equally to this work.

Correspondence to: C. Justin Lee ([cjl@ibs.re.kr](mailto:cjl@ibs.re.kr)) or Ki Duk Park ([kdpark@kist.re.kr](mailto:kdpark@kist.re.kr))

**This PDF File includes:**

Materials and Methods

Figures S1 to S21

Tables S1 to S8

Chemical Information, CI I to IV

**Supplementary Materials and Methods**

GPx assay

To assess the GPx activity, the reaction was prepared in a 96-well plate by adding glutathione reductase and glutathione solution, GPx enzyme (5µl, final concentration 60 U/ml), and the drug (1 µl of KDS12025 and HTEPB) to a total volume of 100 µl per well, followed by manufactural protocol (Abcam, ab102530). The plate was incubated at room temperature for 15 min to allow pre-reactions and drug interactions to occur. Following the incubation, the reaction was initiated by adding cumene hydroperoxide as the substrate to each well. The absorbance at 340 nm was measured in kinetic mode for 10–20 min, and the rate of NADPH consumption was used to calculate GPx activity, reflecting the enzyme’s ability to reduce the hydroperoxide substrate in the presence of the test compound.

DPPH assay

In a 96-well microplate, samples (20 μl) at concentrations ranging from 0.1 to 100 μM were mixed with 80 μL of 50 mM sodium phosphate buffer (pH 7.2) and 100 μL of DPPH (1,1-diphenyl-2-picrylhydrazyl) solution (250 μM in methanol) and incubated for 30-min in the dark. Each sample was quantified using a microplate fluorescence reader (SpectraMax iD5, Molecular Devices) at absorbance (517 nm). Edaravone was used as a positive control. The radical scavenging activity was estimated using the following equation: sample absorbance / control absorbance ×100. The dose-response curve, EC_50_ for the DPPH, was calculated and determined by fitting data with GraphPad Prism software. The fitting method had no special handling of outliers.

Mouse hemoglobin *in vitro* H_2_O_2_ assay

Mouse hemoglobin (Hb) purified from erythrocytes (Cat. CSB-NP004901m, CUSABIO) was used for the ROS-Glo H_2_O_2_ assay. KDS12025 was added in a dose-dependent manner to a reaction mixture containing mouse Hb and H_2_O_2_. The assay was performed according to the manufacturer’s protocol (Promega), and luminescence was measured using an iD5 plate reader. H_2_O_2_-decomposing activity was calculated as the percentage reduction in luminescence relative to controls. Assays with human Hb were conducted in parallel for comparison.

Pharmacokinetic Studies

In vivo pharmacokinetic studies of KDS12025 were conducted by Medicilon (Shanghai, China). For both intravenous (IV) and oral (PO) administration, the compound was dissolved in a vehicle consisting of 10% Solutol and 90% distilled water. Male ICR mice (n = 4 per group) were fasted overnight and allowed access to food 4 h post-dosing, with water provided ad libitum. Following administration, blood samples (0.03 mL per time point) were collected via the submandibular vein. Samples were transferred to tubes containing sodium heparin and centrifuged at 6,800 x g for 6 min at 2–8 °C. Plasma was separated, transferred to Eppendorf tubes, and internal standard (IS: 10 ng/mL verapamil) was added. After vortexing for 1 min and centrifugation at 18,000 x g for 7 min, the supernatant was analyzed by liquid chromatography–tandem mass spectrometry (LC-MS/MS; TQ5500 Triple Quad, SCIEX). Pharmacokinetic parameters including area under the curve (AUC_0–t_ and AUC_0–∞_), elimination half-life (t_1/2_), maximum plasma concentration (C_max_), and time to reach maximum concentration (T_max_) were calculated using noncompartmental analysis in Phoenix WinNonlin 7.0 (Pharsight, USA), an FDA-certified pharmacokinetic software.

For the brain-plasma permeability assessment, brain tissues from male ICR mice (n = 3 per time point) were collected following euthanasia by CO_2_ inhalation. Brains were excised, rinsed with saline, blotted dry, and transferred into pre-labeled Eppendorf tubes (one brain per tube). Samples were snap-frozen on dry ice and stored at -80 °C until analysis. Brain samples were processed using the same LC-MS/MS method. The brain-to-plasma concentration ratio was calculated as the concentration of drug in brain tissue divided by the corresponding plasma concentration.

BBB-permeability test

A parallel artificial membrane permeability assay (PAMPA)^70^ was performed to validate the blood-brain barrier permeability of KDS12025. The procedure began by preparing a donor solution of the compound in a pH 7.4 donor buffer. This solution was added to the wells of a deep well plate, followed by the addition of a high-sensitivity UV plate filled with donor buffer to serve as the blank sample. An initial sample for the UV plate was taken to establish a baseline reading. The PAMPA sandwich was then assembled by adding the donor plate mixture to the donor plate well, followed by placing a BBB-lipid coated membrane on top and an acceptor plate filled with acceptor buffer. This setup was incubated at 25 °C for a specific duration to allow diffusion. Post-incubation, a sample from the acceptor plate was taken, and the UV absorbance was measured to determine the permeated amount of the test compound. The permeability was then calculated using the PAMPA explorer program, considering the initial donor concentration and the acceptor's concentration after incubation.

Off-target selectivity assay

The possibility of KDS12025 on kinase off-target, the KINOMEscan panel of scanEDGE assay was conducted in DiscoverX (CA, USA) using a site-directed competition binding with the test compounds. The assay results are given in Supplementary Table 4 and experimental information is available on the DiscoverX web page ([www.discoverx.com](http://www.discoverx.com)). Moreover, the Delta SafetyScreen87 panel assay was conducted in Eurofins Discovery (Cerep, France). The assay results are given in Supplementary Table 5 and experimental information is available on the DiscoverX web page (<https://www.eurofinsdiscoveryservices.com>).

General Synthetic Method

Reaction progression was checked using analytical thin-layer chromatography (TLC) plates (#1.05715, Merck) and analyzed with 254 nm and 365 nm ultraviolet light. The reaction mixtures were purified by flash column chromatography using silica gel (#1.09385, Merck). Melting points were determined in open capillary tubes using a Standford Research Systems melting point apparatus and were uncorrected. Nuclear magnetic resonance (NMR) spectral data were obtained at 400MHz (^1^H) and at 100MHz (^13^C) using a BRUKER apparatus. Chemical shits (*δ*) were expressed in parts per million (ppm) from tetramethylsilane (TMS), the internal standard and coupling constants (*J*) were expressed in hertz and assigned as follows: s, singlet; d, doublet; t, triplet; q, quartet; AB_q_, AB quartet; br, broad; m, multiplet. All chemical reagents and solvents were of reagent grade, used without further purification and were purchased from commercial sources. Analytical HPLC was performed using a Waters E2695 system equipped with a YMC-Triart C18 column/S-5μM/12nm/Lot no. 17452 (150mm x 4.6 mm diameter). HPLC data were recorded using the following parameters: mobile phase 0.1% acetic acid in H_2_O(A) and MeCN(B), initial gradient 10% B to 100% B in 10 min, then 100% B isocratic hold maintained for 10 min, flow rate of 1.0 mL/min, λ = 254 and 280 nm. All HRMS experiments were conducted on Shimadzu LC-20AD XR UFLC system with an ACE Excel 2 C18-AR column (150 x 2.1 mm inner diameter) from Advanced Chromatography Technology which was connected to a Thermo Q Exactive-quadrupole orbitrap mass spectrometer with an ESI source. The mobile phase consisted of H_2_O(A) and MeCN(B), both of which contained 0.05% formic acid. The initial gradient composed of 2% B was increased to 95% B over 4.9 min. The 95% B gradient was maintained for 3.3 min before being decreased to 2% B for 0.1 min. Column conditioning was performed for 3.6 min (total running time: 12 min). The flow rate and column temperature were set to 0.4 mL/min and 35°C, respectively. The ESI spray was operated in positive ionization mode with a spray voltage of 3.5 kV. Capillary and vaporizer temperatures were 425°C and 320°C, respectively. Compound analysis was performed using full scan mode (150–650 m/z). Compounds were validated by using TLC, ^1^H and ^13^C NMR. TLC, NMR, and analytic data confirmed that the purity of the products was ≥ 95%.

Laser Speckle Contrast Imaging (LSCI)

To assess cerebral perfusion, mice were anesthetized with isoflurane (2-3% in 100% O_2_, maintained at 1.0%) and placed on a heating pad to maintain body temperature. Following scalp incision, the skull surface was exposed without thinning or craniotomy. LSCI was performed using a laser speckle imaging system (RWD Life Science), acquiring frames at 30 Hz. KDS12025 (0.1, 1, or 10 mg/kg/day) or saline was administered via retro-orbital injection immediately prior to imaging. Cerebral blood perfusion and vessel diameter (pipe diameter) were quantified using RWD’s proprietary LSCI analysis software. All imaging was conducted under identical illumination and acquisition parameters.

Hydrogen Peroxide Sensor Specificity Assay

oROS-G was expressed in the NEB BL21(DE3) E. coli strain using the pET29b vector. An overnight LB culture started from a single colony was diluted 1:100 in TBII (MpBio) medium supplemented with 50 μg/mL kanamycin. A total of 1500 mL of expression culture was grown at 37 °C for 4 h, followed by induction with isopropyl-β-D-thiogalactopyranoside (IPTG) and continued culturing at 18 °C for 24 h with shaking at 225 rpm. Cells were then collected by centrifugation at 4,000 x g and resuspended in 50 mM Tris (pH 8.0), 300 mM NaCl, 20 mM imidazole, 1 mM PMSF, 100 μg/mL lysozyme (Sigma-Aldrich), and 10 μg/mL DNase (Sigma-Aldrich). Cells were lysed by sonication and centrifuged at approximately 18,000 x g for 30 min. The soluble fractions were purified using immobilized metal affinity chromatography with gravity columns packed with Ni-NTA agarose resin (Qiagen). Columns were washed with a buffer containing 20 mM imidazole, and proteins were eluted with a buffer containing 300 mM imidazole and 1.5 mM DTT. Proteins were further purified by size-exclusion chromatography (SEC) using an ÄKTA FPLC instrument equipped with a Superdex 200 Increase 10/300 GL column (GE Healthcare Life Sciences) equilibrated with 25 mM Tris (pH 8.0), 150 mM NaCl and 1.5 mM DTT. During experimentation, the protein was diluted to 0.5 µM into cuvettes containing blank (30 mM MOPS, 100 mM KCl, and pH 7.2) with H_2_O_2_ (100 µM, ThermoFisher H325500), NO (100µM, MAHMA NONOate NO donor, Enzo ALX-430-015), or ONOO^-^ (10 µM, Cayman 81565). The concentration of the ONOO^-^ stock was determined spectrophotometrically, using ε302nm = 1670 M^-1^ cm^−1^. Catalase (100nM, Sigma-Aldrich, C1345) was added to the ONOO^−^ sample to remove any H_2_O_2_. Fluorescence intensity was obtained by each fluorescence cuvette read on a spectrophotometer (SpectraMax M5; Molecular Devices). Excitation and emission wavelengths for oROS-G fluorescence were set to 485 nm and 538 nm, respectively.

CIA mouse model and histological analysis

CIA was induced in DBA1 mice by intradermal injection of type II collagen emulsified in Complete Freund's Adjuvant (CFA) on day 0, followed by a booster injection of type II collagen in Incomplete Freund's Adjuvant (IFA) on day 21. Starting from day 23, mice were administered KDS12025 in drinking *ad libitum* at doses of 0, 0.1, or 1 mg/kg/day until day 42. To assess joint inflammation and cartilage thickness in CIA mice, joint samples were collected and fixed in 10% formalin. The samples were then decalcified, embedded in paraffin, and sectioned. For histological evaluation, sections were stained with H&E to assess inflammation and with Toluidine Blue to evaluate cartilage thickness. The stained sections were examined under a light microscope, and images were captured for quantitative analysis of inflammatory cell infiltration and cartilage integrity.

Blood H_2_O_2_ measurement

C57BL/6 mice were intraperitoneally (i.p.) injected with lipopolysaccharide (LPS) and sacrificed at 2, 6, and 24 h post-injection. Blood was collected from the heart using a syringe pre-treated with 0.5 M EGTA to prevent coagulation. Plasma was separated by centrifuging the collected blood at 1,500 × g for 10 min at 4 °C. H_2_O_2_ levels in the plasma were quantified using the Amplex Red assay according to the manufacturer’s instructions. The H_2_O_2_ levels peaked at 6 h post-LPS injection. Subsequently, KDS12025 (3 mg/kg/day) was intraperitoneally administered concurrently with LPS at the 6 h time point, and plasma H_2_O_2_ levels were measured to assess the effect of the compound.

Isolated Red Blood Cell (RBC) assay

RBCs were isolated following a modified version of the protocol described by Hanson et al. (2008). Whole blood was collected from male ICR mice (5–6 weeks old) via retro-orbital bleeding under isoflurane anesthesia. Blood was transferred using heparin-coated capillaries into EDTA-coated microcentrifuge tubes and centrifuged at 500 x g for 10 min. Plasma and buffy coat were removed, and the RBC pellet was washed three times with phosphate-buffered saline (PBS) containing 2% fetal bovine serum (FBS) to remove leukocytes and plasma components. The final RBC suspension was adjusted to 5 x 10^3^ cells/µL, and cell concentration was verified using an automated hematology analyzer (Horiba Microsemi LC-662).

H_2_O_2_ was quantified using the ROS-Glo H_2_O_2_ Assay kit (Promega) according to the manufacturer’s protocol. RBCs were dispensed into white 96-well plates at a final volume 70 µL per well. KDS12025 was tested at final concentrations of 0.01, 0.1, and 1 µM, and co-treated with 1 µM H_2_O_2_. The ROS-Glo substrate was added simultaneously to all wells. Plates were incubated for 10 min at 37°C. After incubation, ROS-Glo detection reagent was added directly to each well. Plates were then incubated for 20 min at room temperature. Luminescence was measured using a microplate reader (SpectraMax) according to kit settings. Relative luminescence units (RLU) were background-subtracted and normalized to the RBC+ H_2_O_2_ only control group. All conditions were tested in triplicate. Results present the combined average of two independent experiments; each performed with the same experimental layout and controls.


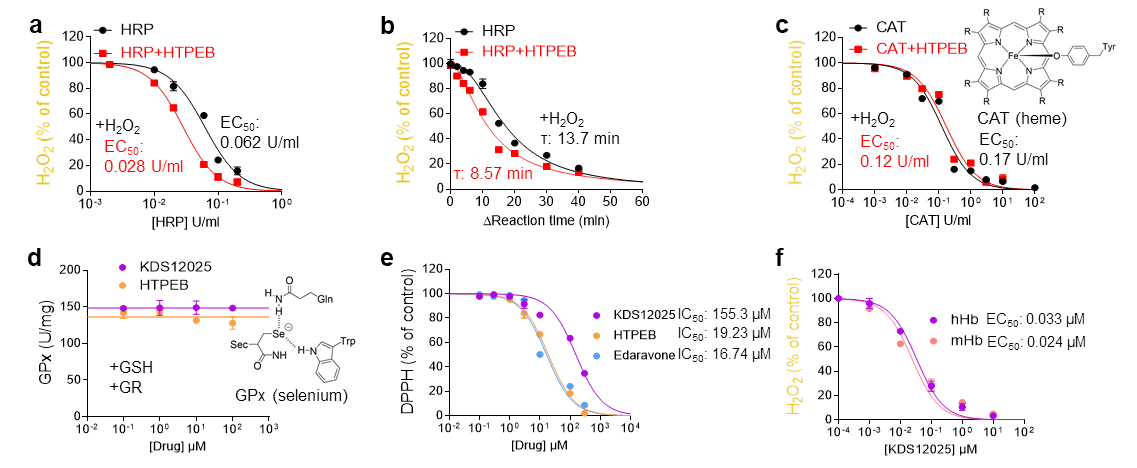


**Figure. S1. H_2_O_2_ assays using various peroxidase families.** To test whether HTPEB enhances H_2_O_2_-decomposition with HRP, CAT, and GPx, we used ROS-Glo H_2_O_2_ assay. **a,** Dose-response curve of HRP in ROS-Glo assay with (red square shapes, EC_50_ for H_2_O_2_ decomposition is 0.028 U/ml) or without (black circle shapes, EC_50_ is 0.062 U/ml) an HTPEB (10 μM). **b,** Reaction time-response curve for HRP with (red square shapes, tau value is 8.57 min) or without (black circle, tau value is 13.7 min) HTPEB. **c**, Dose-response curve of CAT (heme as a cofactor) in ROS-Glo assay with (black circle, EC_50_ for H_2_O_2_ decomposition is 0.12 U/ml) or without (red square, EC_50_ is 0.17 U/ml) an HTPEB. **d,** Dose-response curve of GPx (selenium as a cofactor) in GPx colorimetric assay kit to quantitate the GPx activity with HTPEB (orange circle) or KDS12025 (purple circle). **e,** DPPH (1,1-diphenyl-2-picrylhydrazyl) radical scavenging assay with HTPEB (IC_50_ for DPPH scavenging is 19.23 μM), KDS12025 (IC_50_ is 155.3 μM), and Edaravone (IC_50_ is 16.74 μM). These results indicate that HTPEB enhances the H_2_O_2_-decomposing activity of the heme-containing protein like HRP, not the selenium-containing protein. The dose-response curve and EC_50_ were calculated and determined by fitting data with GraphPad Prism software. **f,** Dose-response curves of KDS12025 in the ROS-Glo H₂O₂ decomposition assay using either human hemoglobin (hHb, EC_50_ is 0.033 μM) or mouse hemoglobin (mHb, EC_50_ is 0.024 μM), showing comparable enhancement of pseudoperoxidase activity across species. Data are presented as the mean ± s.e.m.

**Figure. S2. General procedure for the preparation of KDS compounds.** Synthetic procedures for the preparation of intermediates and KDS compounds.


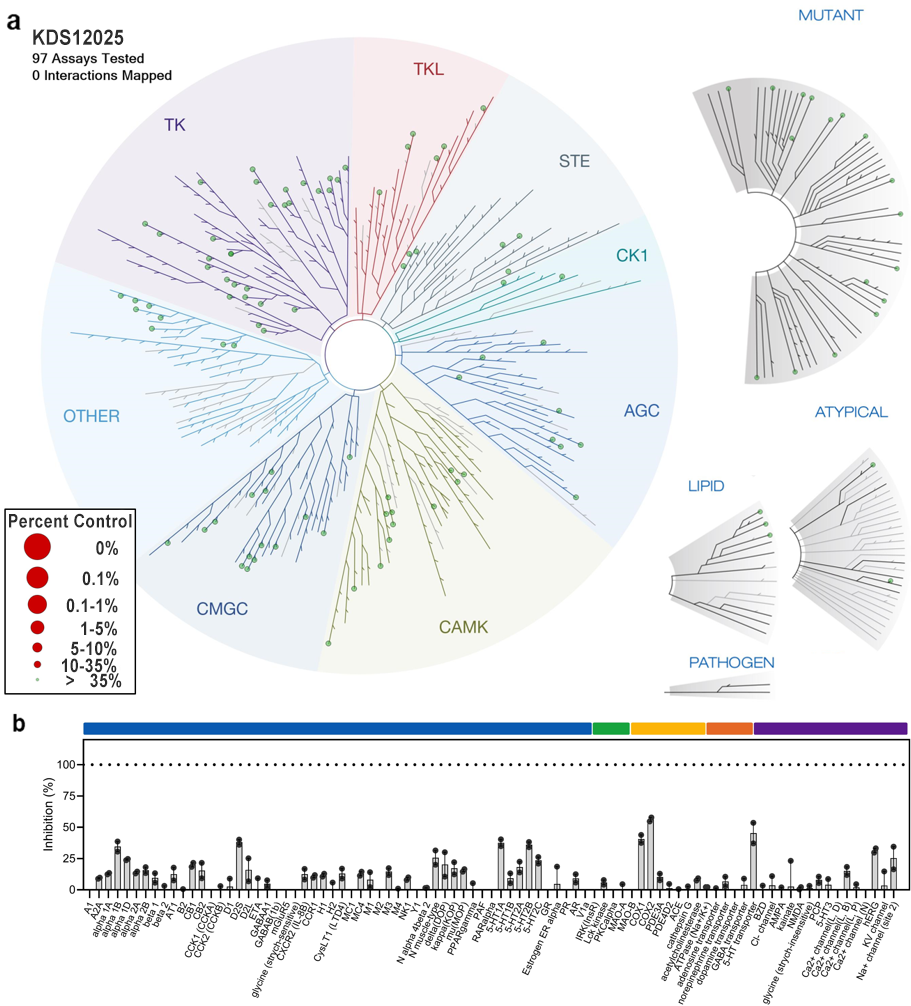


**Figure. S3. KINOMEscan and Off-target Selectivity Screening Results for KDS12025 at 1000 nM.** To test the off-target of KDS12025, we employed screening assays. **a,** Schematic diagram of KINOME*scan*^TM^ screening results categorizing human kinases and disease-associated mutant variants. Competitive binding assays for 97 human kinases were performed at 1000nM KDS12025, and the amount of inhibition through the control ligand reaction is expressed in the size of the colored circle (green/red). Zero interactions mapped means no meaningful responses with ≥ 50% inhibition. TK, Tyrosine Kinase; TKL, Tyrosine Kinase Like; STE, Yeast STE-MAPK family; CK1, Casein Kinase 1; AGC, PKA, PKG, PKC family; CAMK, Calmodulin/Calcium regulated kinases; CMGC, CDK, MAPK, GSK3 and CLK; see Supplementary Table 4 for detailed results. **b,** The off-target selectivity for 87 primary molecular targets at 1000 nM of KDS12025, including G protein-coupled receptors (blue line), kinases (green line), non-kinase enzymes (yellow line), transporters (orange line), and various channels (purple line); see Supplem Table 5 for detailed results. These results indicate that KDS12025 shows favorable drug-like properties.


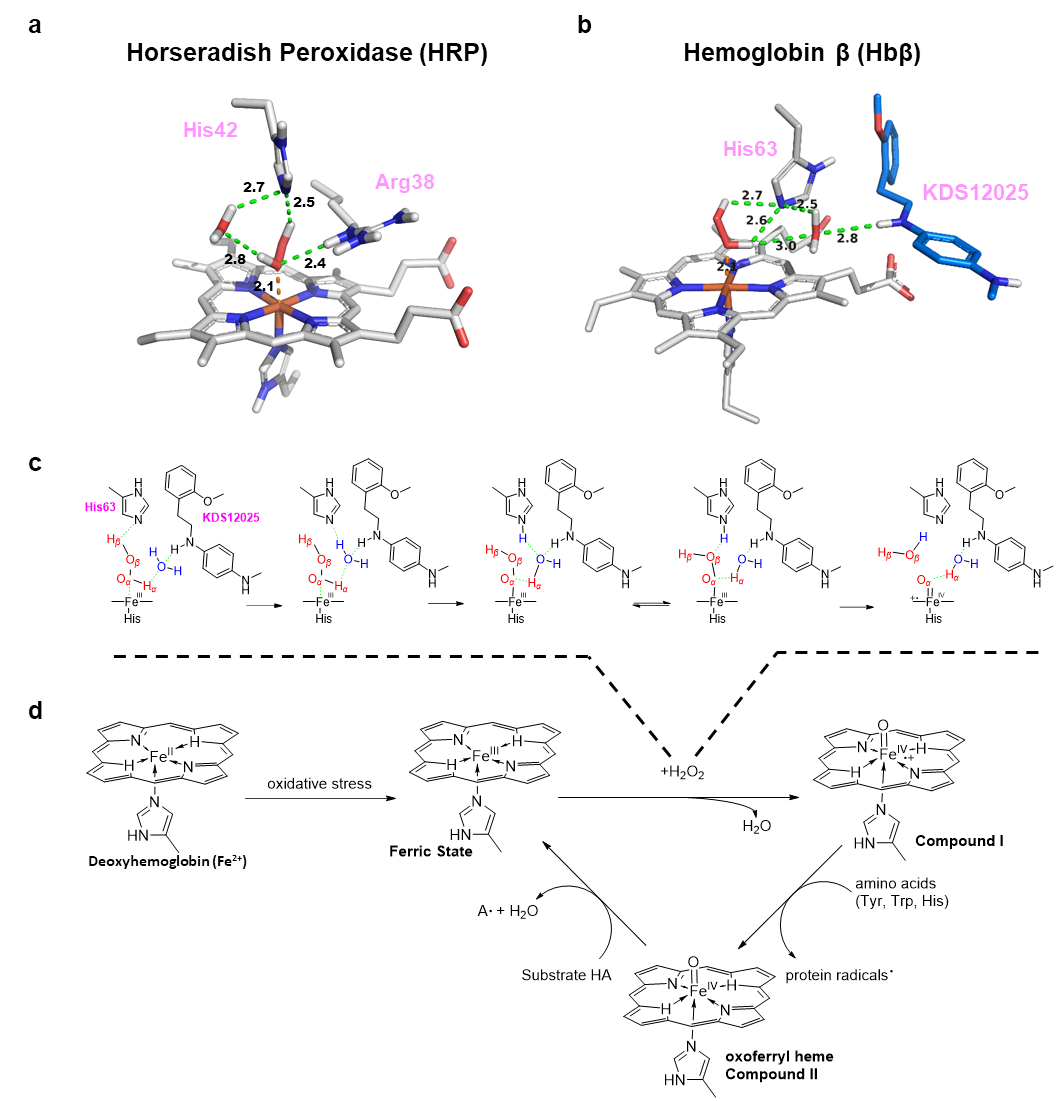


**Figure. S4. Molecular mechanisms of KDS12025 enhancement of hemoglobin’s peroxidase activity. a,** Representative image of HRP and H_2_O - H_2_O_2_ – His42 – Arg38 reactive conformation^26^ for H_2_O_2_ decomposition and subsequent activation to Por°±Fe^IV^=O (PDB ID: 7ATJ)^65^. **b,** Predicted reactive conformation of H_2_O – H_2_O_2_ – His63 – KDS12025 for H_2_O_2_ composition and subsequent activation to Por°±Fe^IV^=O (Compound I). KDS12025 acts as a critical electron donor and coordinates H_2_O and H_2_O_2_ with the heme iron, compensating for the absence of Arg38 in Hb. **c,** Proposed reactive conformation mechanism for the decomposition of H_2_O_2_ (red color) for proton transfer and release of H_2_O (blue color) with KDS12025 assisting in coordinating H_2_O and subsequently H_2_O_2_ with the iron atom. **d,** General Hb peroxidase cycle. KDS12025 facilitates the decomposition of H_2_O_2_ and the formation of Por°±Fe^IV^=O (Compound I). Importantly, this mechanism allows KDS12025 to enhance Hb’s thermodynamic and kinetic efficiency in decomposing aberrant H₂O₂, even at low Hb levels. These results suggest the KDS12025’s novel mode-of-action, which can act like Arg38 in HRP.


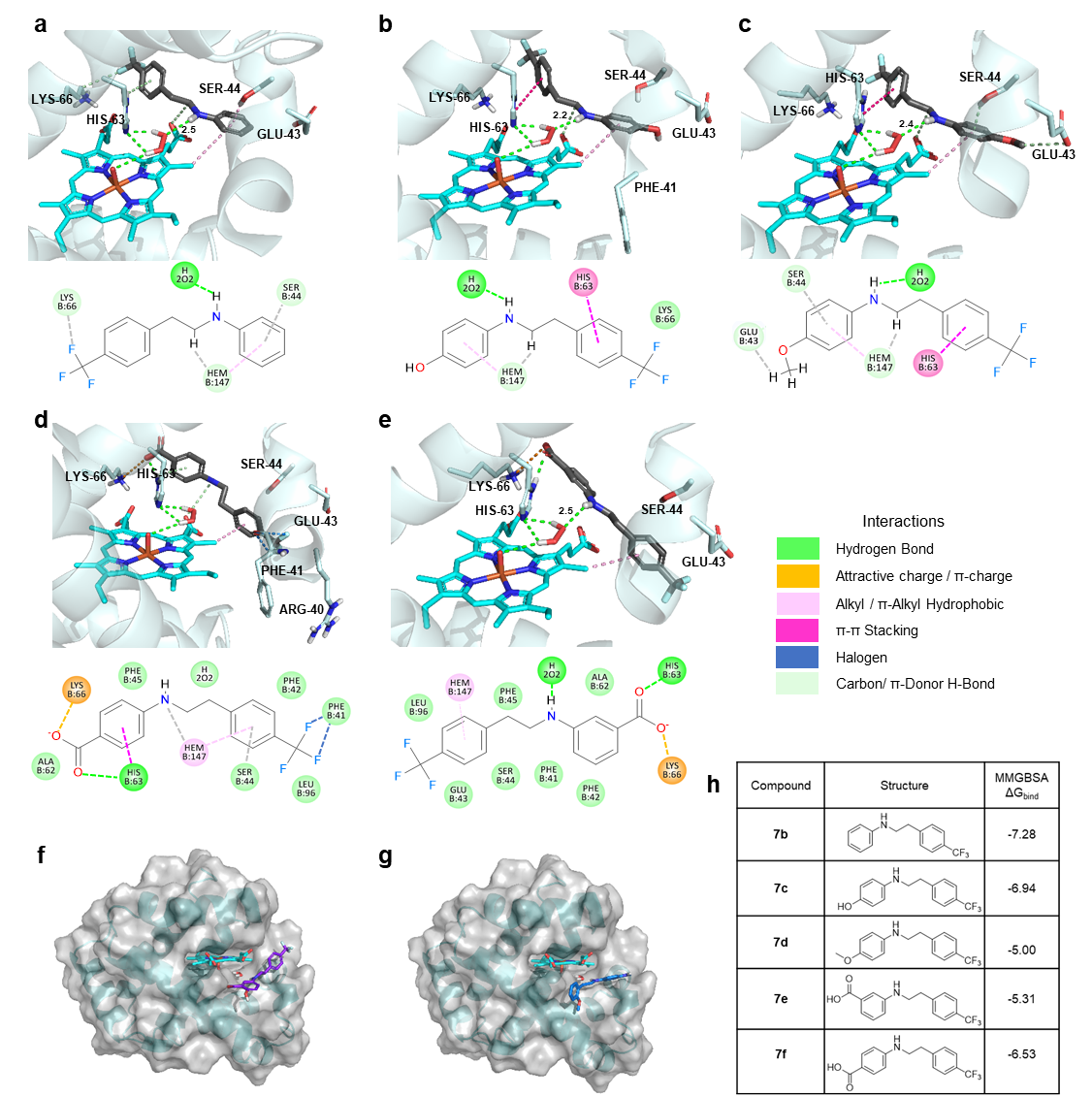


**Figure. S5. Binding modes of selected *N*-phenethylaniline compounds in Hbβ chain.** Estimated binding energies (ΔG_bind_) values correlate with experimentally determined compound activity, therefore validating the proposed binding site. **a–e,** Predicted 3D binding mode and 2D interaction maps of compounds **7b (a)**, **7c (b)**, **7d (c)**, **7f (d)** and **7e (e)** binding to the Hbβ-subunit. **f–g,** General view of the predicted 3D binding mode of **HTPEB (f)** and **KDS12025 (g)** to the Hbβ-subunit. Hemoglobin β-subunit shown in surface view and heme cofactors/ligands shown in stick form. **h,** Binding energy (ΔG_bind_) values for selected inactive compounds, calculated by the MMGBSA method. The corresponding enzyme assay results are displayed in Supplementary Table 7.


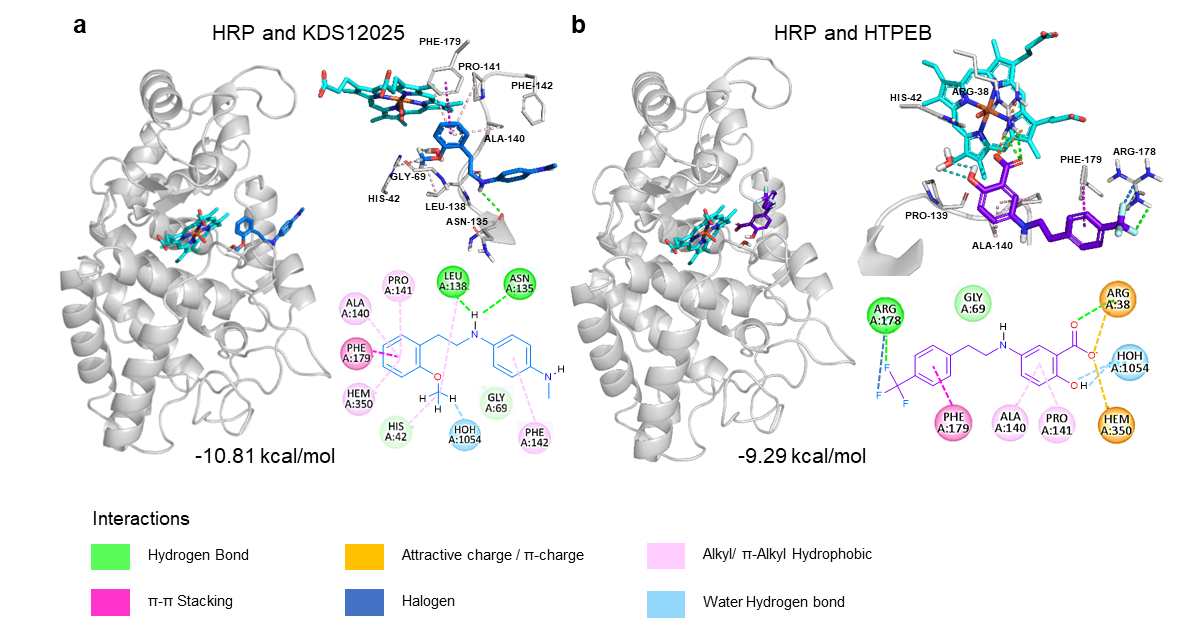


**Figure. S6. Binding modes of selected active compounds in HRP proposed by molecular docking. a,** General view of protein-ligand binding (left), 3D interaction scheme (top right), and 2D interaction map (bottom right) of KDS12025. **b,** General view of protein-ligand binding (left), 3D interaction scheme (top right), and 2D interaction map (bottom right) of HTPEB. The calculated binding energy (ΔG_bind_) values for each compound binding event are specified at the bottom center.


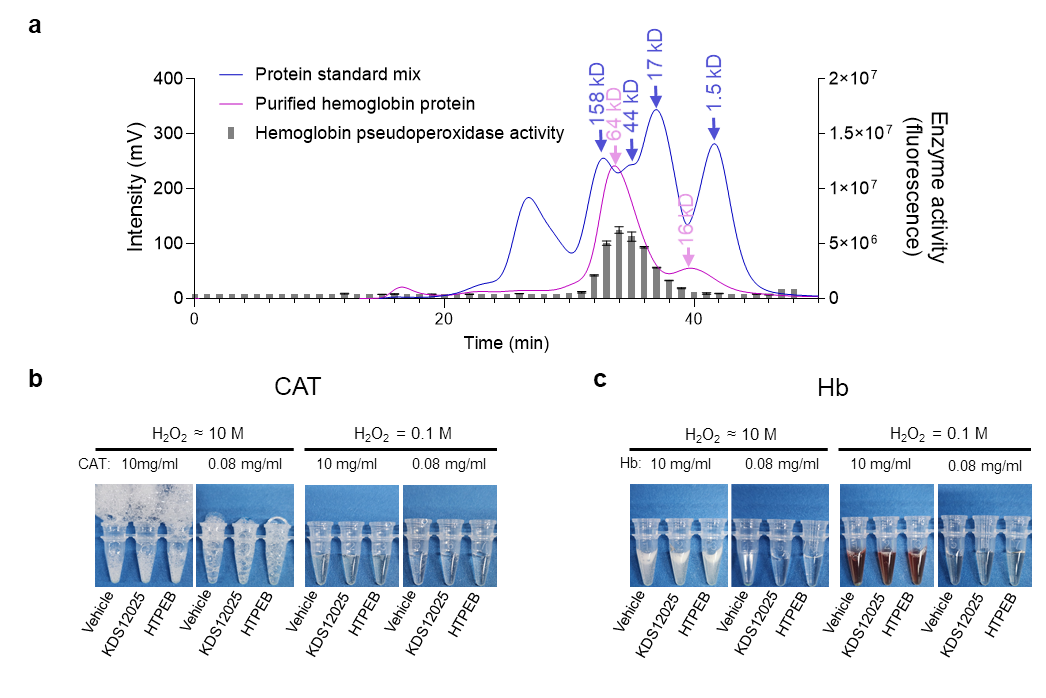


**Figure S7. Tetramer-dependent pseudoperoxidase activity of Hb and lack of oxygen liberation**. **a,** Recombinant Hb was size-separated by gel filtration chromatography, and elution profile was compared to a standard protein marker mix. The major Hb peak was detected between the 158 kD and 44 kD markers, corresponding to the ~64 kD tetramer/heterodimer form (pink trace). Hb’s pseudoperoxidase activity was measured in each elution fraction using a fluorescence-based H_2_O_2_ assay (gray bars). Activity peaked exclusively in the 64 kD fraction and declined in lower molecular weight fractions, including the ~16 kD monomeric Hb. These results indicate that tetrameric Hb structure is functionally optimal for KDS12025-mediated H_2_O_2_ decomposition. To test whether Hb might also function as CAT, which decomposes H_2_O_2_ into oxygen and water, while also evaluating the role of KDS12025 in this biochemical reaction. **b,** The assay shows oxygen release from CAT under varied conditions, with observable changes that indicate the degree of oxygen liberation. **c,** The assay shows oxygen release from Hb under varied conditions, with observable changes indicating oxygen liberation. These results demonstrate that Hb is not a catalase, and KDS12025 does not facilitate CAT activity/ Data are presented as the mean ± s.e.m. ns, not significant.


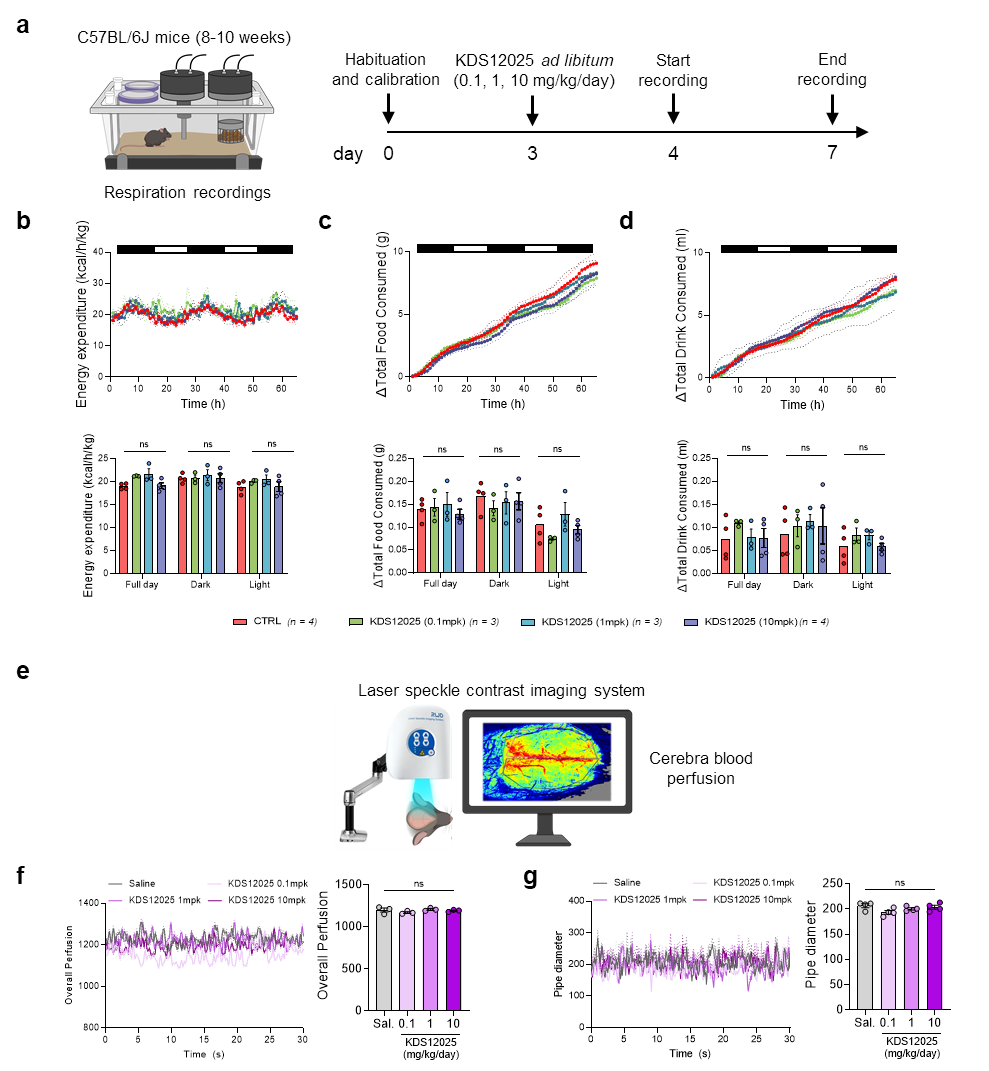


**Figure S8. KDS12025 does not alter metabolic processes and perfusion.** To assess whether KDS12025 affects mice's energy expenditure, food, or drink consumption, we employed the PhenoMaster to measure in the mouse chamber. **a,** Timeline of PhenoMaster experiments with the administration of KDS12025 (0.1, 1, 10 mg/kg/day). **b–d,** Measurement of energy expenditure (**b**), total food consumption (**c**), and total drink consumption (**d**) during the administration of KDS12025 at concentrations of 10, 1, and 0.1 mg/kg/day for both night (dark) and day (white) cycles, suggesting that KDS12025 enhances Hb’s peroxidase activity without overall metabolism in mice. **e,** Schematic illustration of the LSCI system for monitoring cerebral blood perfusion in anesthetized mice. Pseudocolor images represent cortical blood flow across the brain surface. Saline or KDS12025 (0.1, 1, or 10 mg/kg) was administered via retro-orbital injection, and perfusion was immediately recorded. **f,** Representative traces (left) and quantification (right) of overall cerebral perfusion measured by LSCI following saline or KDS12025 injection. **g,** Representative traces (left) and quantification (right) of vessel diameter (pipe diameter) extracted from LSCI recordings under each treatment condition. Data are presented as the mean ± s.e.m. ns, not significant. Additional statistics are provided in Supplementary Table 7.


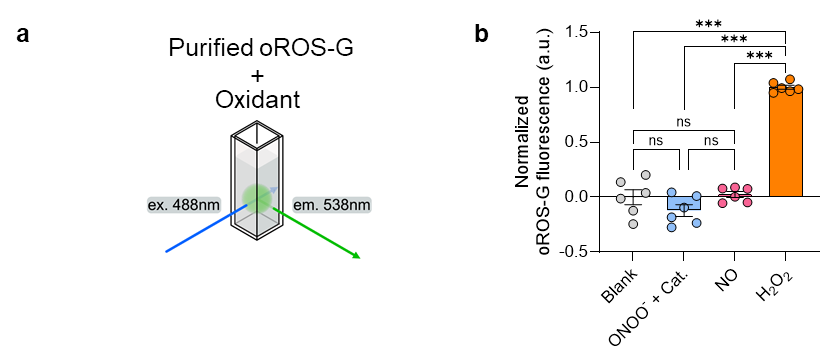


**Figure. S9. Specificity assessment of oROS-G with other oxidant molecules.** To examine the selectivity of oROS-G, we tested other oxidant molecules, including nitric oxide and peroxynitrite. **a,** schematic representation of the oxidant specificity assay conducted for oROS-G. Purified oROS-G protein, prepared in MOPS buffer, was contained in cuvettes and exposed to various oxidants. Fluorescence changes were recorded using a fluorescence spectrometer to assess the sensor’s specificity and response dynamics to different oxidizing agents. **b,** The normalized fluorescence changes before and after the addition of each oxidant are shown. Peroxynitrite, a potent H_2_O_2_ generator, was introduced alongside catalase to mitigate the production of H_2_O_2_ as a byproduct. Data are presented as the mean ± s.e.m. ****P* < 0.001; ns, not significant. Additional statistics are provided in Supplementary Table 7.


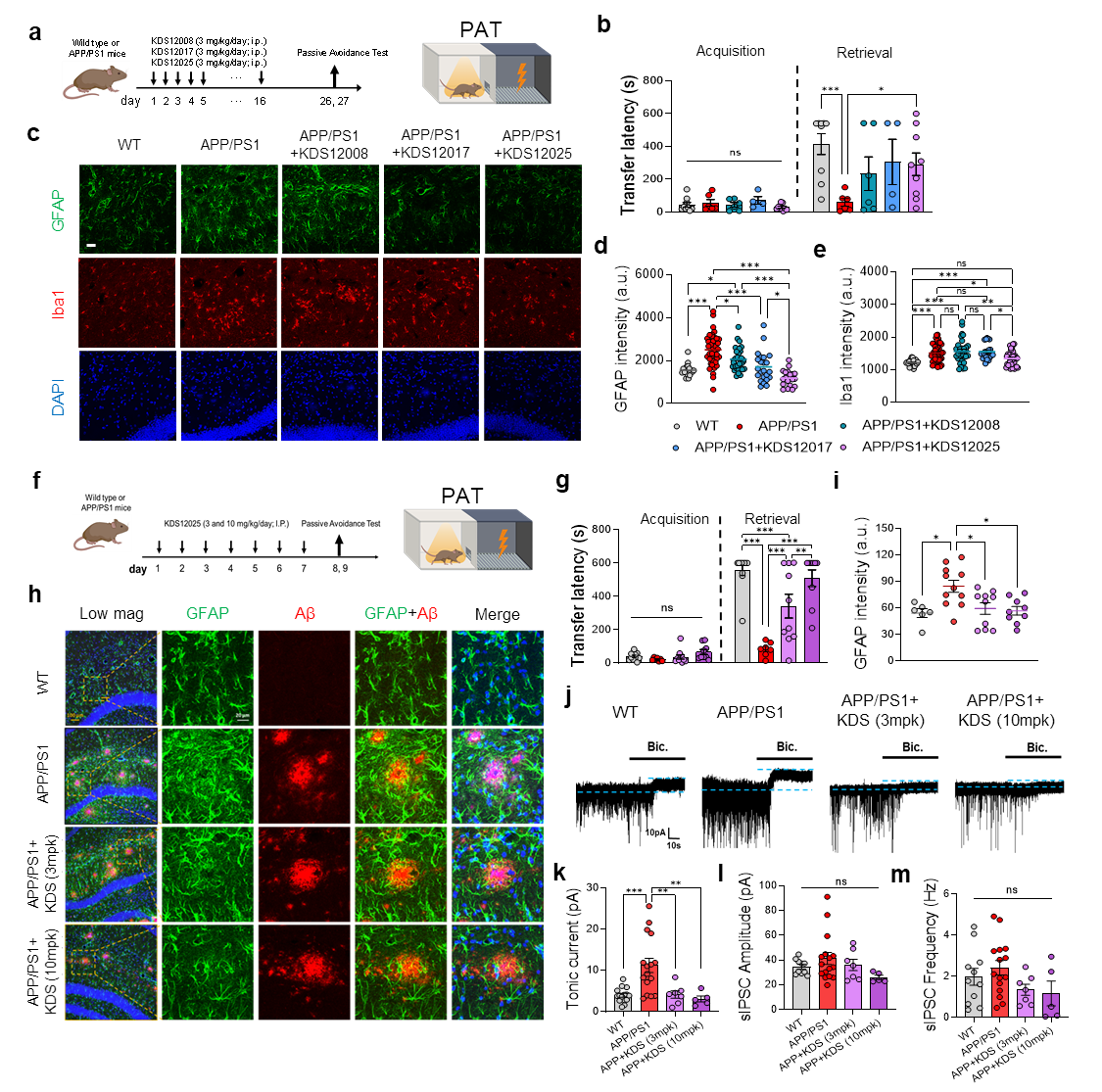


**Figure. S10. KDS12025 effectively reduces AD-like pathology in vivo*.*** To further investigate whether the KDS12008, KDS12017, and KDS12025 could alleviate AD symptoms like astrogliosis and memory impairment in the APP/PS1 mouse model. **a,** Schematic timeline of comparison KDS12008, KDS12017, and KDS12025 (3 mg/kg/day intraperitoneal; i.p. injection) in APP/PS1 mice. **b,** Transfer latency to enter the dark chamber where the day had a foot shock during the acquisition session in PAT. **c,** Representative image for GFAP, Iba1, and DAPI in each conditions. Scale bar, 20 μm. **d,e,** Mean intensity of GFAP (**d**) and Iba1 (**e**) in the hippocampus. **f,** Schematic timeline of comparison dose-dependent effect of KDS12025 (3 and 10 mg/kg/day i.p. injection). **g,** Transfer latency to enter the dark chamber in the PAT. **h,** Representative immunostaining image for GFAP and Aβ in each conditions. **i,** Mean intensity of GFAP in the hippocampus. **j,** Representative trace of GABA_A_ receptor-mediated tonic GABA current, revealed by the antagonist bicuculline from the dentate gyrus granule cells of the hippocampus. **k,** Tonic GABA current from the granule cells. **l,m,** Amplitude (**l**), and frequency (**m**) of sIPSC measured from granule cells. Data are presented as the mean ± s.e.m. **P* < 0.05, ***P* < 0.01, ****P* < 0.001; ns, not significant. Additional statistics are provided in Supplementary Table 7.


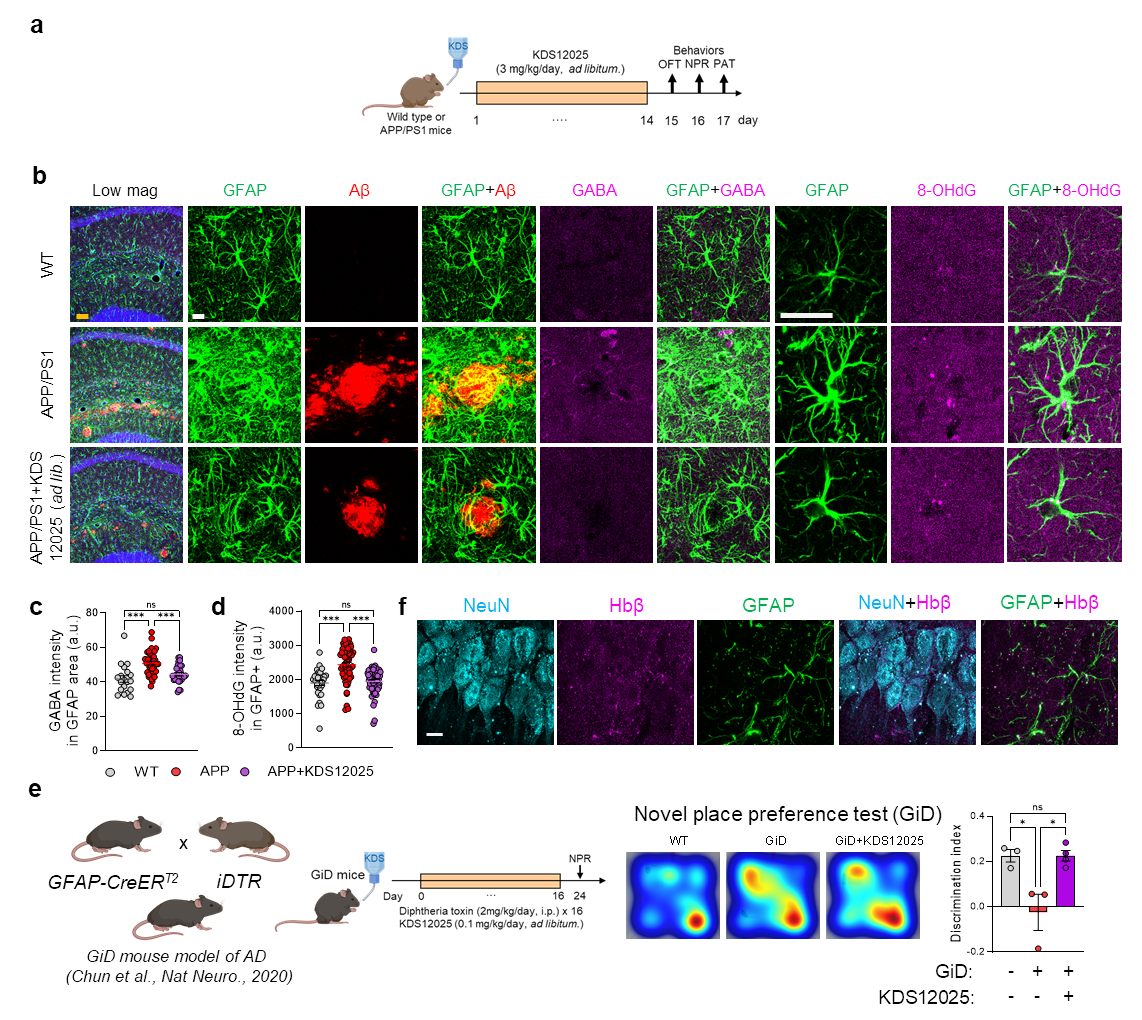


**Figure. S11. KDS12025 rescues astrocytic Hbβ, GABA, and 8-OHdG expression. a,** Schematic timeline of APP/PS1 mice and KDS12025 treatment (3 mg/kg/day; drinking *ad libitum*). **b,** Representative images of the hippocampal region for GFAP, Aβ, GABA, 8-OHdG (oxidative stress marker) of WT, APP/PS1, and APP/PS1+KDS12025 (3 mg/kg/day, drinking *ad libitum*). Scale bars, 100 μm (Low mag); 20 μm (High mag); 20 μm (8-OHdG). **c,d,** Mean intensity of GABA (**c**) and 8-OHdG (**d**) in GFAP-positive area. **e,** To assess the extremely low dose of KDS12025 in an AD mouse model, we used the severe AD mouse model. Left: GFAP-CreERT2 and iDTR crossbred GiD mouse model (Chun et al., Nature Neuroscience, 2020). Right: Results of NPR test following administration of KDS12025 (drinking ad libitum, 0.1 mg/kg/day) to the GiD mouse model. **f,** Representative Lattice-SIM images showing NeuN, Hbβ, and GFAP expression in the pyramidal layer of the hippocampus. Scale bar, 20 μm. Data are presented as the mean ± s.e.m. **P* < 0.05, ****P* < 0.001; ns, not significant. Additional statistics are provided in Supplementary Table 7.


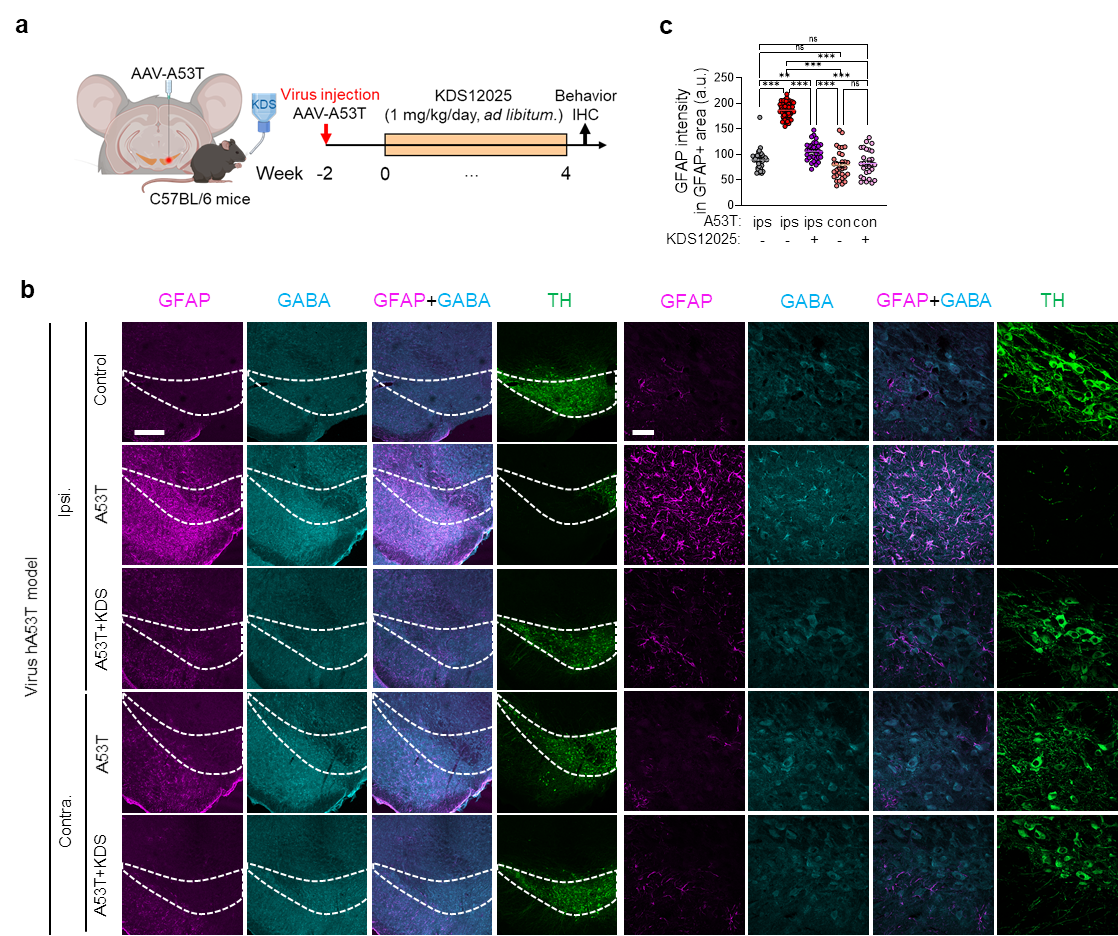


**Figure. S12. KDS12025 rescues PD pathologies in the A53T mouse model.** To examine the effects of KDS12025 in the PD, we used a human A53T overexpression PD mouse model. **a,** Timeline of human A53T-induced PD mouse model (unilaterally overexpression of A53T by injecting AAV carrying human A53T virus) with KDS12025 treatment (1 mg/kg/day for four weeks, drinking *ad libitum*). **b,** Representative images of the SN region with ipsilateral and contralateral side for GFAP, GABA, and TH in control, A53T, and A53T+KDS12025 mice. Scale bars, 150 µm (left); 10 µm (right). Related to Fig. 4i. **c,** Mean intensity of GFAP in GFAP-positive area. These results demonstrate that KDS12025 rescues the PD pathologies, including astrogliosis, TH-positive neuronal loss, and aberrant astrocytic GABA. Data are presented as the mean ± s.e.m. **P* < 0.05, ***P* < 0.01, ****P* < 0.001; ns, not significant. Additional statistics are provided in Supplementary Table 7.


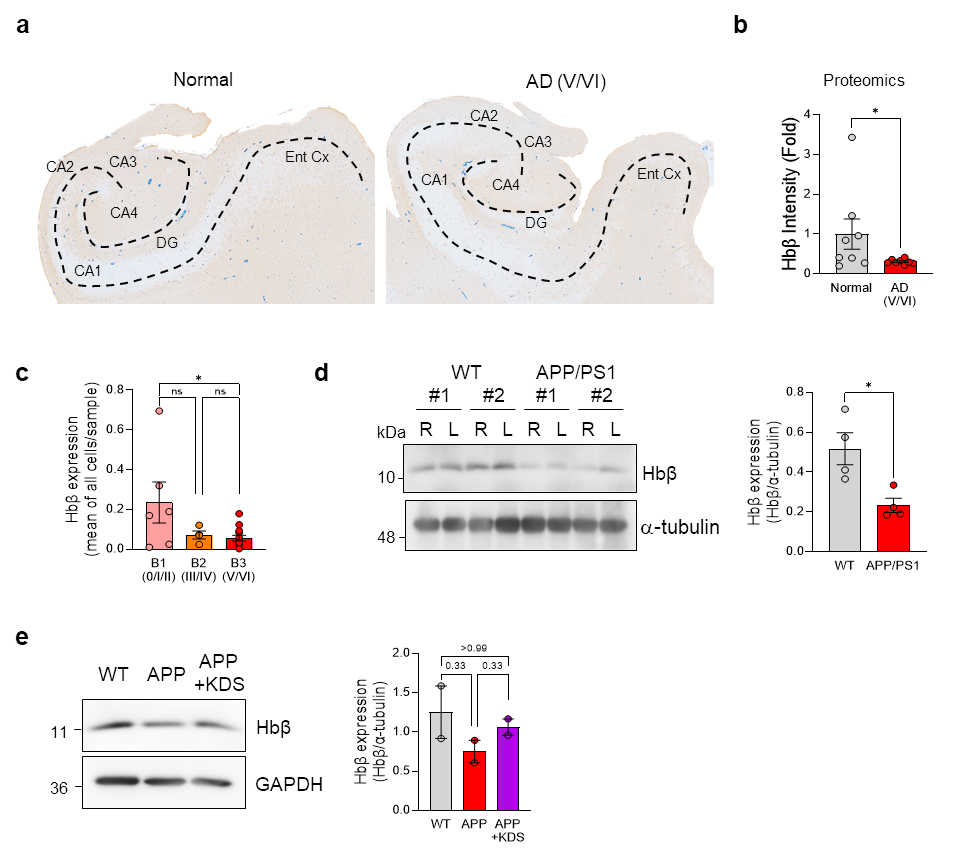


**Fig. S13. Reduced hippocampal Hbβ in AD patients and mouse model.** To assess the clinical significance of the astrocytic Hbβ in AD patients, we performed proteomics on postmortem hippocampal tissues from both normal subjects and AD patients *(*n = 3 patient per group; Braak stage V and VI). **a,** Representative images of postmortem hippocampal tissue from normal subjects and AD patients. **b,** Hbβ proteomic data of the hippocampal postmortem brain showed a significant decrease in Hbβ levels in AD postmortem brains. **c,** Hbβ expression levels in astrocytes depend on Braak stages from single cell astrocyte RNA sequence from publicly open database from [https://ad-progression-atlas.partners.org](https://ad-progression-atlas.partners.org/).) **d,** Protein quantification of Hbβ protein in the hippocampus of WT littermate and APP/PS1 mice (n = 2 mice per group). Parallel in human and mouse, reduced astrocytic Hbβ expression found in the AD patients and AD mouse model. **e,** Protein quantification of Hbβ protein in the hippocampus of WT littermate, APP/PS1, and APP+KDS12025 mice (n = 2 mice per group). Data are presented as the mean ± s.e.m. **P* < 0.05. Additional statistics are provided in Supplementary Table 7.


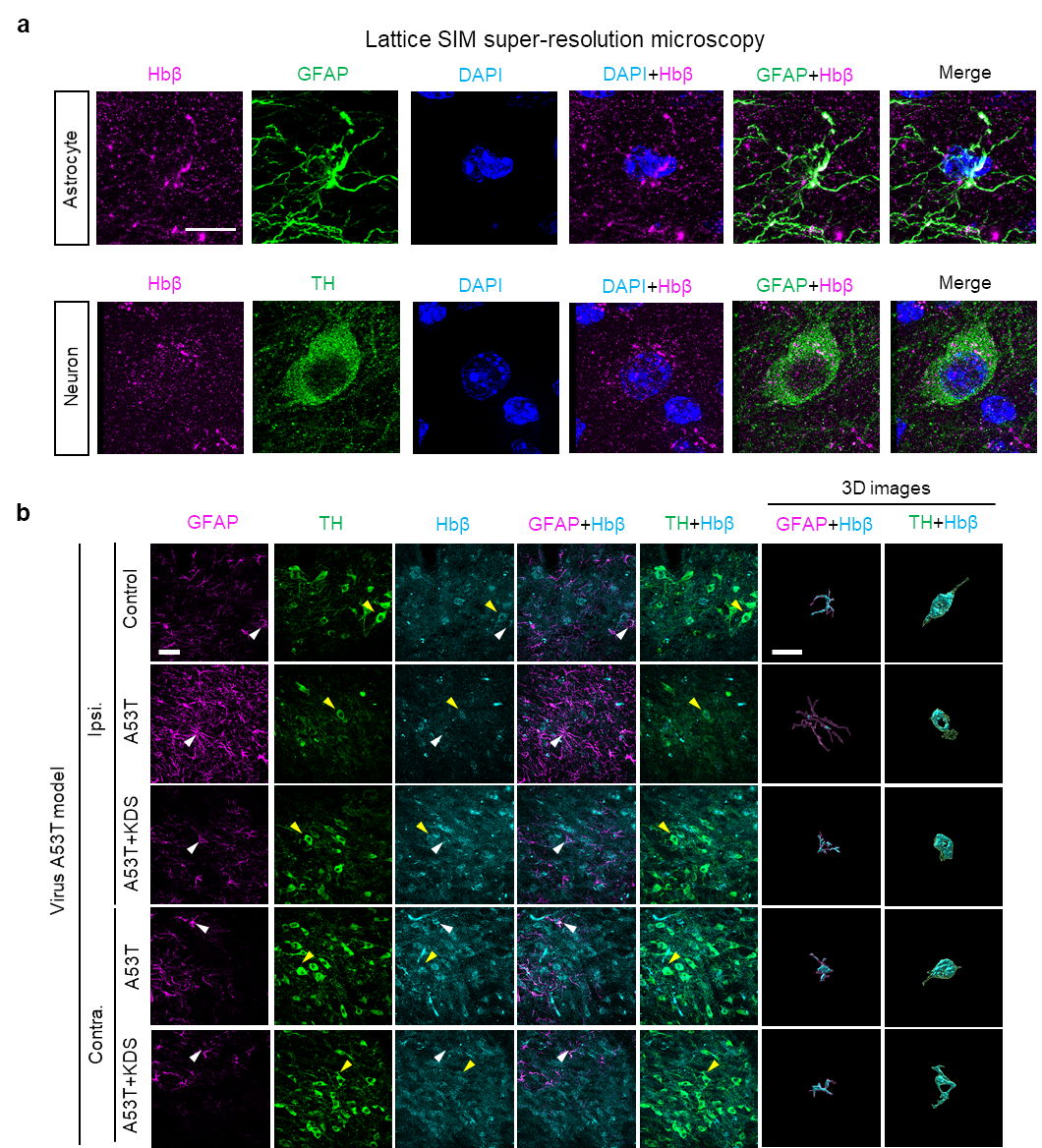


**Figure. S14. Expression of Hbβ in TH- and GFAP-positive cells in SNpc.** To investigate the expression pattern of Hbβ in the PD mouse model, we performed IHC. **a,** Representative super-resolution microscopy image showing Hbβ expression in GFAP-positive astrocytes and TH-positive dopaminergic neurons within the SNpc. The image highlights the distinct expression patterns of Hbβ across astrocytes and neurons, visualized using IHC. (Scale bars, 20 μm). **b,** Representative confocal images of the SNpc region with ipsilateral and contralateral side for GFAP, GABA, and TH in control, A53T, and A53T+KDS12025 mice. Representative 3D images from Imaris software (magenta, GFAP; green, TH; cyan, Hbβ). Related to Fig. 6o. Scale bars, 20 μm (left); 10 μm (Imaris).


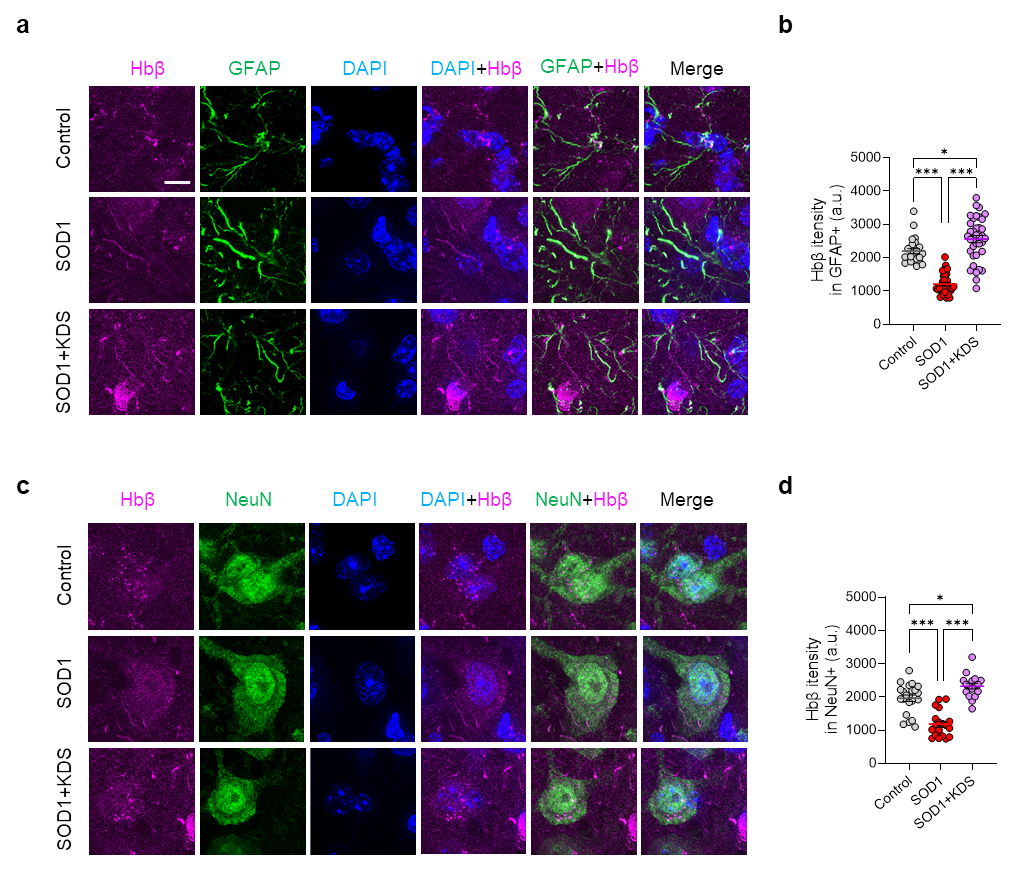


**Figure. S15. Expression of Hbβ in GFAP and NeuN-positive cells in the spinal cord.** To investigate the expression pattern of Hbβ in the ALS mouse model, we performed IHC. **a,** Representative super-resolution microscopy image showing Hbβ expression in GFAP-positive astrocytes within the ventral spinal cord (L2–L5). The image highlights the distinct expression patterns of Hbβ across astrocytes (Scale bars, 20 μm). **b,** Quantification of Hbβ in the GFAP-positive astrocytes across the group. **c,** Representative super-resolution microscopy image showing Hbβ expression in NeuN-positive neurons within the same section showin in panel a. This panel is presented separately to highlight neuronal localization of Hbβ. Quantification of Hbβ in the NeuN-positive neurons across the group. Data are presented as the mean ± s.e.m. **P* < 0.05, ***P* < 0.01, ****P* < 0.001; ns, not significant. Additional statistics are provided in Supplementary Table 7.


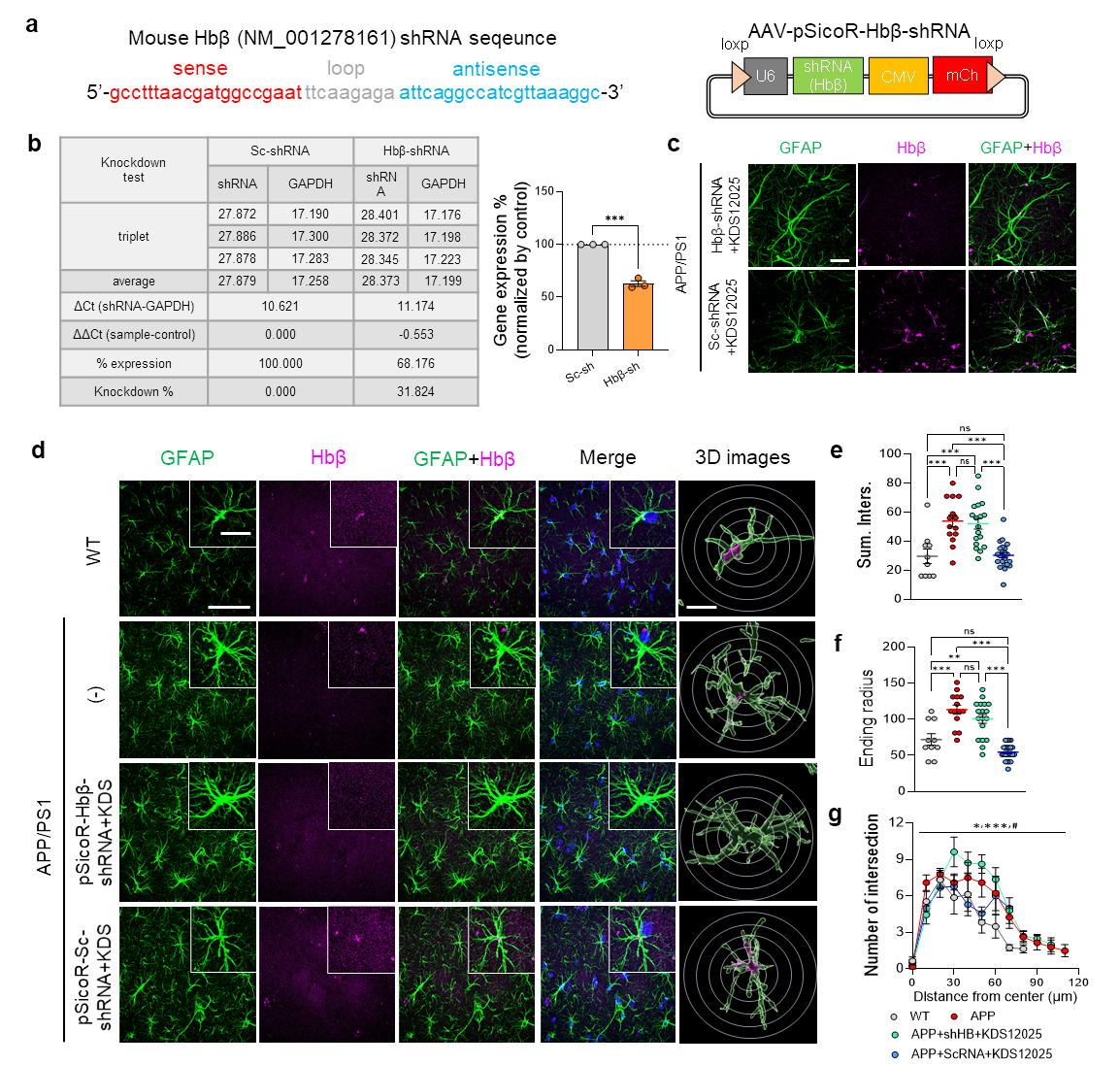


**Figure. S16. Validation of pSicoR-Hbβ-shRNA.** To silence the expression of Hbβ, we developed Hbβ-shRNA. **a,** The sequence and map of Hbβ-shRNA used in this study. **b,** Validation of Hbβ-shRNA efficiency in cultured astrocytes via qRT-PCR, comparing the knockdown results with Sc-shRNA (left), and the bar graph shows the relative expression level (right). **c,** Lattice SIM imaging demonstrates Hbβ levels in astrocytes after injecting control Sc-shRNA versus Hbβ-shRNA. Scale bar, 20 μm. **d,** Representative images of the hippocampus for GFAP and Hbβ in WT, APP/PS1, APP/PS1+Hbβ shRNA+KDS12025 (3 mg/kg/day), and APP/PS1+Sc-shRNA+KDS12025 (3 mg/kg/day). Representative 3D images from Imaris software. Scale bars, 50 μm (main); 10 μm (inset); 5 μm (Imaris). **e–g,** Summary graph showing the sum of intersections (**e**), ending radius (**f**), and number of intersections (**g**) in astrocytes by Sholl analysis. Data are presented as the mean ± s.e.m. **P* < 0.05, ***P* < 0.01, ****P* < 0.001; ns, not significant. Additional statistics are provided in Table S7.


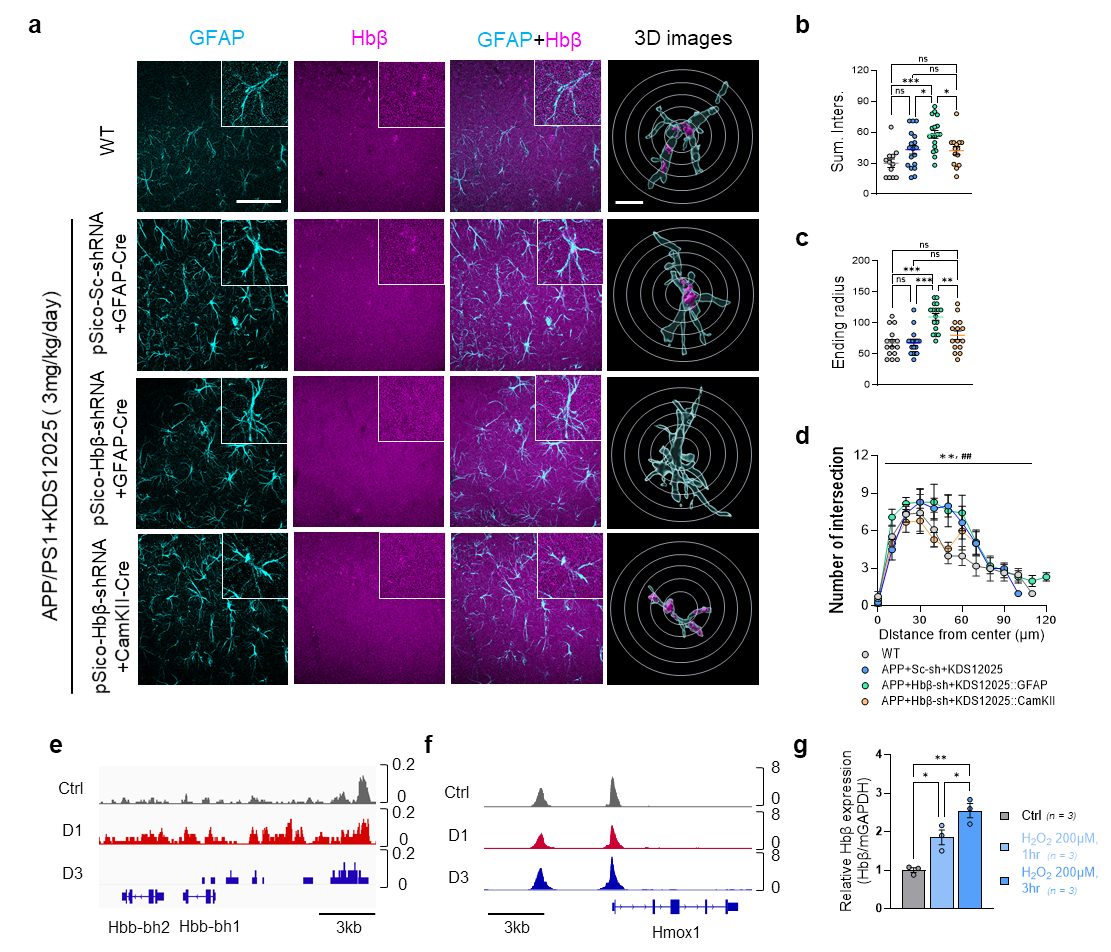


**Figure. S17. Astrocyte-specific knockdown and genetic changes in acute application of oxidative stress in cultured astrocytes. a,** Representative images of the hippocampus showing GFAP and Hbβ staining in WT and APP/PS1 mice treated with AAV-pSico-Hbβ-shRNA (or Sc-shRNA) in combination with AAV-GFAP-Cre or AAV-CaMKII-Cre, followed by KDS12025 (3 mg/kg/day, drinking ad libitum) for 2 weeks. The groups include APP/PS1 with GFAP-Cre and Hbβ-shRNA, APP/PS1 with GFAP-Cre and Sc-shRNA, and APP/PS1 with CaMKII-Cre and Hbβ-shRNA. Representative 3D reconstructions were generated using Imaris software. Scale bars: 50 μm (main), 10 μm (inset), and 5 μm (Imaris). **b–d,** Summary graph showing the sum of intersections (**b**), ending radius (**c**), and number of intersections (**d**) in astrocytes by Sholl analysis. **e,** Representative ATAC-seq tracks showing chromatin accessibility at the Hbβ locus accessibility profiles across control (Ctrl, gray) in cultured astrocytes one day after the H_2_O_2_ application (D1, red) and three day after (D3, blue). Chromatic accessibility was transiently increased at D1 but markedly closed at D3, suggesting a dynamic epigenetic regulation in response to oxidative stress. **f,** Representative ATAC-seq track for the Hmox1 locus in the same experimental conditions (D1, red and D3, blue). **g,** Quantitative qRT-PCR measurement of Hbβ mRNA in cultured astrocytes exposed to 200 µM H_2_O_2_ for 1 h and 3 h. Data are presented as the mean ± s.e.m. **P* < 0.05, ***P* < 0.01, ****P* < 0.001; ns, not significant. Additional statistics are provided in Supplementary Table 7.


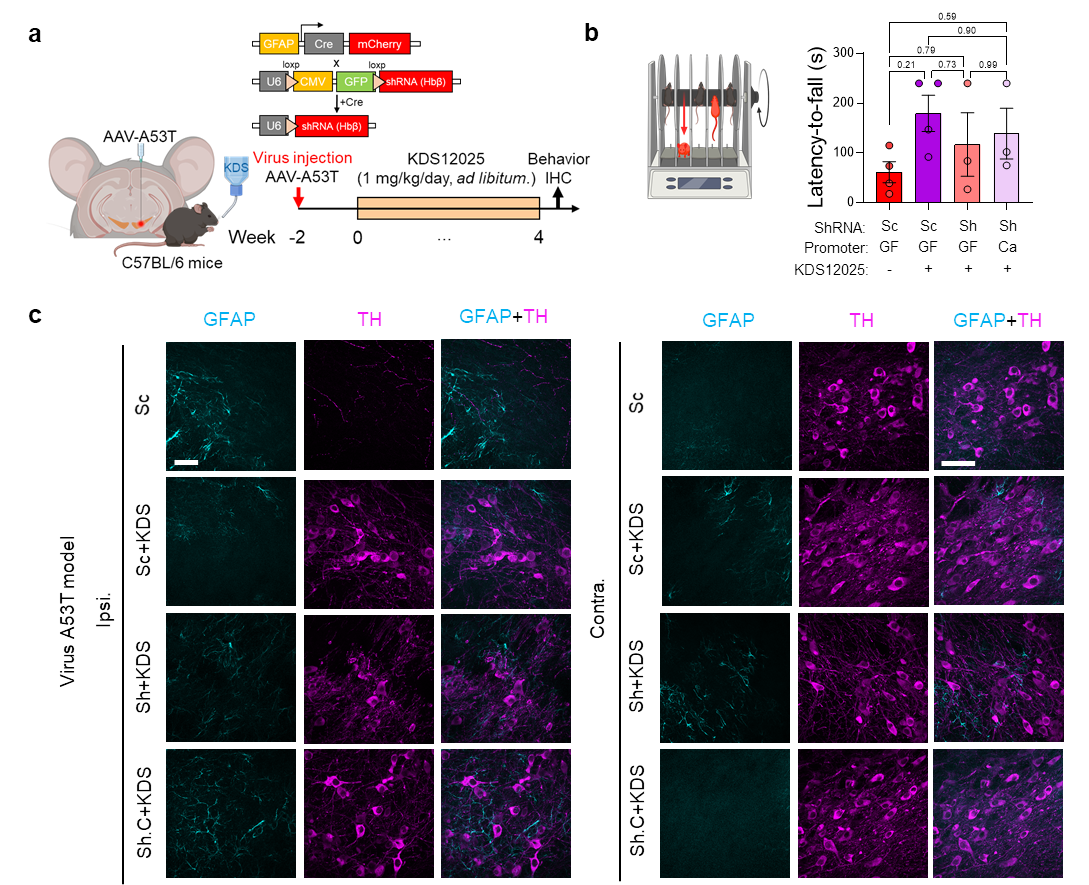


**Figure. S18. SNpc Hbβ is partially necessary for KDS12025 action. a,** Schematic timeline of injecting an AAV-pSico-Hbβ (or Sc)-shRNA-GFP with GFAP (or CaMKII) Cre virus into the SNpc in human α-synuclein A53T overexpression mouse model of PD. **b,** Schematic diagram of rotarod test and latency-to-fall of A53T+Sc::GFAP, A53T+Sc::GFAP+KDS12025 (1 mg/kg/day, *ad libitum* drinking water), A53T+Sh::GFAP+KDS12025, and A53T+Sh::CaMKII+KDS12025 mice. **c,** Representative images of the SNpc region with ipsilateral and contralateral side for GFAP, and TH of A53T+Sc, A53T+Sc+KDS, A53T+Sh::GFAP+KDS, and A53T+Sh::CaMKII+KDS mice (scale bar, 10 µm). Data are presented as the mean ± s.e.m. **P* < 0.05, ***P* < 0.01, ****P* < 0.001; ns, not significant. Additional statistics are provided in Supplementary Table 7.


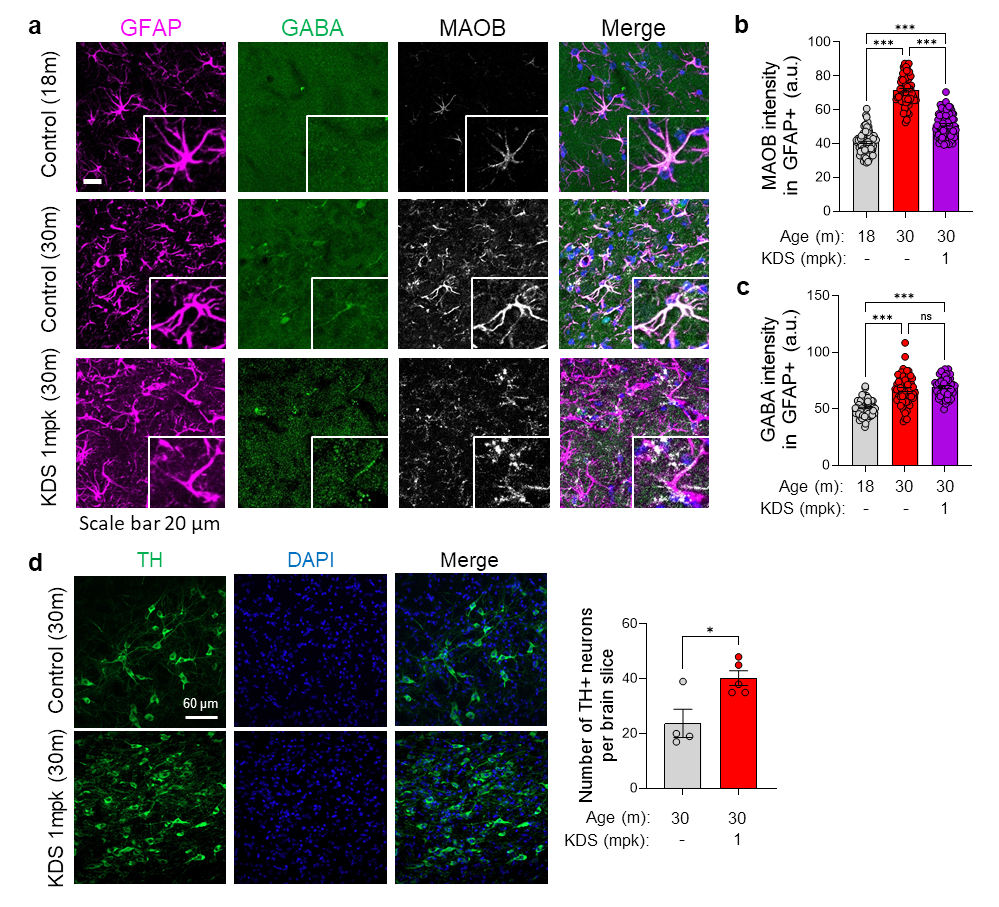


**Figure. S19. KDS12025 reverts astrogliosis and TH-neuronal loss on 30-month-old mice and its effect on 36-month-old mice. a,** Representative images showing GFAP, GABA, and MAOB staining in control mice at 18 months, 30 months, and KDS12025-treated mice (1 mg/kg/day) at 30 months of age. (Scale bar, 20 μm). **b,** Quantification of MAOB intensity in GFAP-positive areas of control (18 months), control (30 months), and KDS12025-treated mice (1 mg/kg/day, 30 months). **c,** Quantification of GABA intensity in GFAP-positive areas in control and KDS12025-treated groups (30 months). **d,** Prevention of TH-positive neuronal loss in the SNpc in 30-month-old mice treated with KDS12025 (1 mg/kg/day) and corresponding quantification bar graph. Data are presented as the mean ± s.e.m. **P* < 0.05, ***P* < 0.01, ****P* < 0.001; ns, not significant. Additional statistics are provided in Supplementary Table 7.


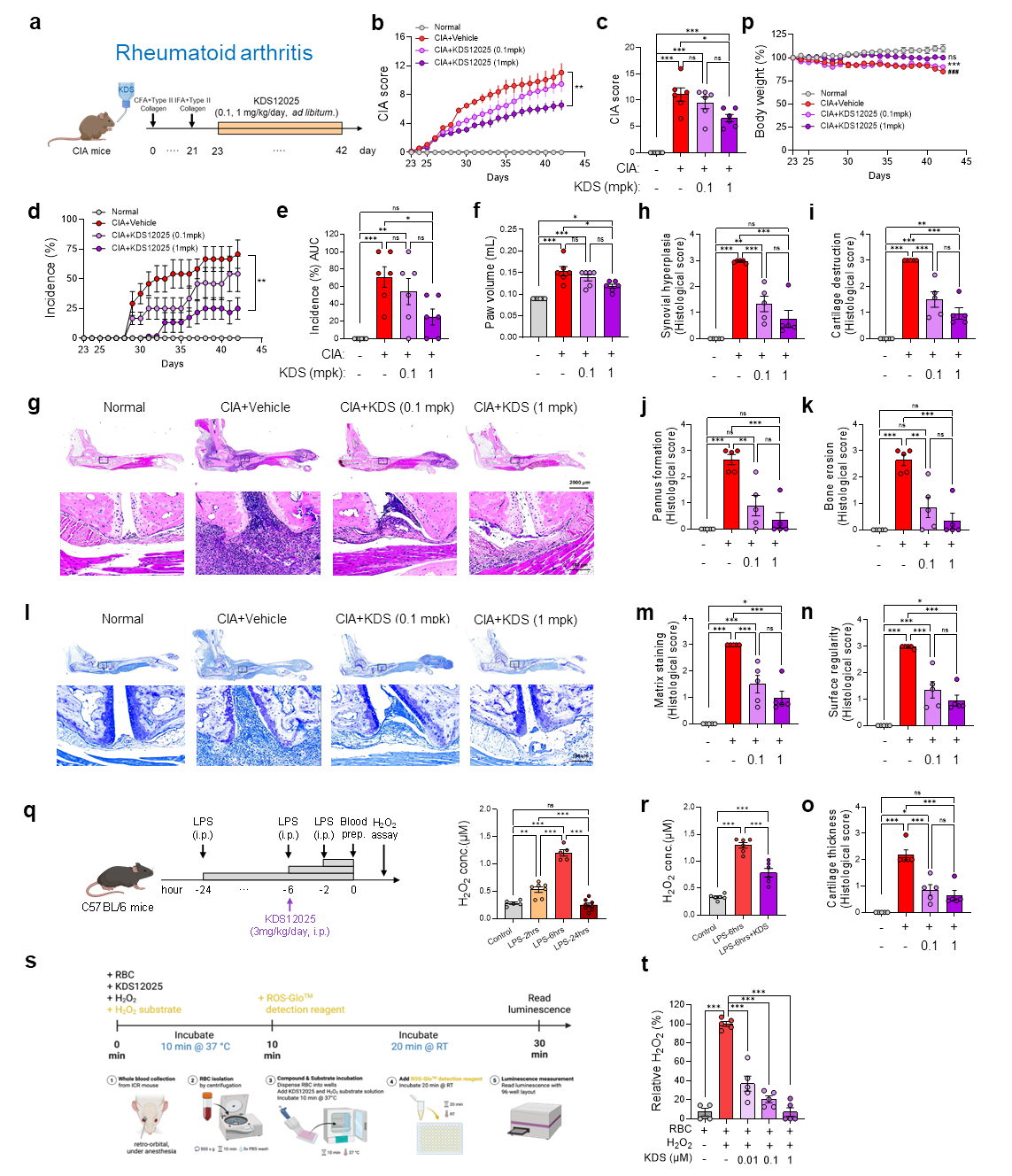


**Figure. S20. Mitigating RA symptoms by KDS12025.** To examine the effects of KDS12025 on the inflammatory disease, we used the RA mouse model. **a,** Schematic timeline of CIA mouse model and KDS12025 treatment (0.1, 1 mg/kg/day; drinking *ad libitum*). **b.** Measurement of arthritis score in normal, CIA (collagen-induced arthritis), CIA+KDS12025 (0.1 and 1 mg/kg/day, drinking *ad libitum*) mice. **c,** Summarized CIA score at day 42. **d,e,** A percentage of incidence (**d**) and area under the curve (AUC) (**e**) of symptom severity in normal, CIA, and CIA+KDS12025 mice. **f,** Paw volume (mL) across the groups. **g,** Representative images of H&E staining in normal, CIA, CIA+KDS12025 (n = 6 mice per group). **h–k,** Quantification synovial hyperplasia (**h**), cartilage destruction (**i**), pannus formation (**j**), and bone erosion (**k**) from H&E staining. **l,** Representative images of toluidine blue staining in normal, CIA, and CIA+KDS12025 mice (0.1 and 1 mg/kg/day). **m–o,** Quantification of matrix (**m**), surface regularity (**n**), and cartilage thickness (**o**) from toluidine blue-stained sections. **p,** Body weight difference (%) in normal, CIA, and CIA+KDS12025 mice (0.1 and 1 mg/kg/day). **q,** To measure the systemic level of H_2_O_2_, we injected an LPS to induce the inflammation acutely. Timeline of LPS (20 mg/kg, i.p. injection)-induced H_2_O_2_ levels of blood from the left ventricle of the heart in mice and measured by an Amplex red assay (left). Time-dependent LPS (20 mg/kg, i.p. injection, 2, 6, and 24 h)-induced H_2_O_2_ levels in the blood (right). **r,** KDS12025 (3 mg/kg, i.p. injection) treatment at 6 h of LPS and measurement of H_2_O_2_ from plasma. **s,** Timeline of the H₂O₂ assay using purified mouse RBCs treated with KDS12025. **t,** KDS12025 dose-dependently reduced H₂O₂ levels in purified RBCs (0.01–1 μM). Data are presented as the mean ± s.e.m. **P* < 0.05, ***P* < 0.01, ****P* < 0.001; ns, not significant. Additional statistics are provided in Supplementary Table 7.


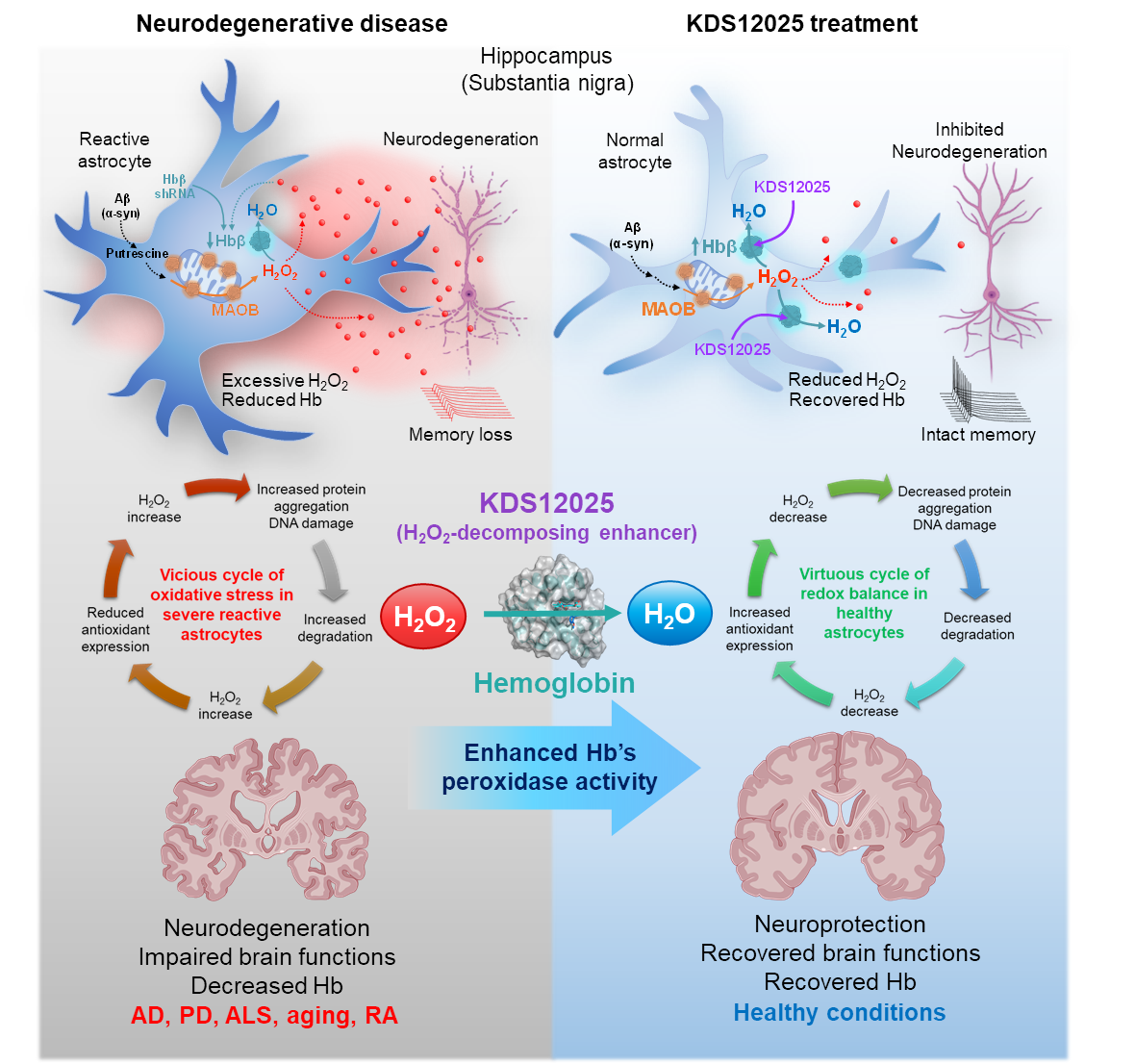


**Figure. S21. Graphical abstract.** (**Top left**) Neurodegenerative diseases like AD and PD involve amyloid beta or α-synuclein toxins triggering astrocytes to produce putrescine, which MAOB converts to aberrant H_2_O_2_. Excessive astrocytic H_2_O_2_ causes neurodegeneration in hippocampal pyramidal neurons or substantia nigra dopaminergic neurons, inhibiting neuronal firing and leading to cognitive and motor impairments. In a chronic H_2_O_2_ exposure environment, a reduction in Hbβ leads to a vicious cycle, vulnerable to oxidative stress and decreasing Hbβ levels, as seen with Hbβ-shRNA experiments. Conversely, the H_2_O_2_-decomposing enhancer KDS12025 promotes brain Hb’s pseudoperoxidase activity, reducing astrocytic H_2_O_2_ levels and normalizing reactive astrocytes. This prevents neurodegeneration, maintains memory and motor functions, and restores Hbβ to normal levels, indicating a return to a virtuous cycle. (**Middle**) In the context of neurodegenerative diseases, severe reactive astrocytes initiate a vicious cycle of oxidative stress characterized by increased H_2_O_2_ levels. This aberrant H_2_O_2_ induces protein aggregation and DNA damage, further exacerbating degradation processes leading to even higher H_2_O_2_ levels. Concurrently, antioxidant levels diminish, perpetuating the vicious cycle. Conversely, KDS12025 treatment breaks this vicious cycle by reducing H_2_O_2_ by enhancing Hb’s H_2_O_2_-decomposing pseudoperoxidase activity. As a result, H_2_O_2_ levels decrease, protein aggregation and DNA damage are diminished, and antioxidant levels are maintained, thereby establishing a virtuous cycle. (**Bottom**) In conclusion, aberrant H_2_O_2_ causes neurodegeneration and brain function impairment in AD, PD, ALS, and aging with reduced astrocytic Hb. Notably, KDS12025 also demonstrates antioxidative effects in non-neurodegenerative conditions such as RA, highlighting its broad therapeutic potential against oxidative stress-related pathologies. To conclude, KDS12025 enhances Hb's H_2_O_2_-decomposing pseudoperoxidase activity, mitigating oxidative stress even at extremely low Hb levels and restoring Hb expression. By breaking the vicious cycle of oxidative damage, KDS12025 facilitates the recovery of astrocytic and neuronal health, ultimately supporting a healthy brain and restoring its functions.

**Table S1. Parallel artificial membrane permeability assay (PAMPA) of HTPEB and KDS12025**

| PAMPA^a^ BBB permeability | | | | | |
| --- | --- | --- | --- | --- | --- |
| Compound | Concentration | Incubation Time | P_e_  (10^-6^cm/sec) | BCS code^b^ | Method |
| Progesterone | 50 μM | 4 h | 42.18 | High (CNS+) | U.V |
| Ranitidine | 50 μM | 4 h | 0 | Low (CNS-) | U.V |
| KDS12025 | 50 μM | 4 h | 67.43 | High (CNS+) | U.V |
| HTPEB | 50 μM | 4 h | 3.56 | High (CNS-) | U.V |

^a^PAMPA; parallel artificial membrane permeability assay

| Permeability classification | | CNS +/- classification | |
| --- | --- | --- | --- |
| **P_e_ (10^-6^cm/sec)** | **Classification** | **P_e_ (10^-6^cm/sec)** | **Classification** |
| > 0.4 | High | > 10 | + |
| < 0.4 | Low | < 10 | - |

**Table S2. SAR analysis of HTPEB**

|  | | | | |
| --- | --- | --- | --- | --- |
| Cpd. | | **Structure** | **HB-EC_50_** | **HRP-EC_50_** |
| Salicylic Acid |  | | >10 | >10 |
| p-Cresotic acid |  | | >10 | >10 |
| Mesalazine |  | | 0.2129 | 3.604 |
| 2-(4-(Trifluoromethyl) phenyl)ethylamine |  | | >10 | >10 |

**Table S3. Effects synthesized compounds on hydrogen peroxide scavenging activity**

|  | | | | |
| --- | --- | --- | --- | --- |
| cmpd | **A** | **B** | **HB-EC_50_** | **HRP-EC_50_** |
| HPTEB |  |  | 0.1551 | 1.520 |
| 7b |  |  | 393.2 | >10 |
| 7c |  |  | 5.090 | >10 |
| 7d |  |  | 23.41 | 13.28 |
| 7e |  |  | >10 | >10 |
| 7f | ­ |  | >10 | 22.11 |
| 8a (KDS12017) |  |  | 0.08622 | 0.8276 |
| 8b (KDS12008) |  |  | 0.06704 | 0.7953 |
| 8c  (KDS12025) |  |  | 0.04489 | 0.5289 |
| 8d |  |  | 0.08551 | 0.5163 |
| 8e |  |  | 0.06946 | 0.7896 |
| 8f |  |  | 0.07806 | 0.8973 |
| 8g |  |  | 0.08838 | 0.3782 |
| 8h |  |  | 0.06911 | 0.4999 |

**Table S4. In vivo pharmacokinetic parameters of KDS12025**

| Cmpd. | PK^a^ | | | | | | | | | |
| --- | --- | --- | --- | --- | --- | --- | --- | --- | --- | --- |
|  | IV | | | | | | PO | | | *F* (%) |
|  | *AUC_all_* (ng*h/mL) | *Cl* (mL/h/kg) | *V_ss_* (L/kg) | | *t_1/2_* (h) | *C_max_* (ng/mL) | | | *AUC_all_* (ng*h/mL) |  |
| KDS12025 | 686.60±118 | 74442.14±1493 | 5033.68±1407 | | 0.96±0.09 | 2305.40±344 | | | 3601.96±662 | 87.43 |
| Cmpd. | **PK-BBB^b^** | | | | | | | | | |
|  | *plasma* | | | | | *brain* | | | | |
|  | *AUC_all_* (ng*h/mL) | *Cl* (mL/h/kg) | *V_ss_* (L/kg) | | *t_1/2_* (h) | *C_max_* (ng/mL) | | | *AUC_all_* (ng*h/mL) | *t_1/2_* (h) |
| KDS12025 | 720.75 | 6910.53 | 2382.40 | | 0.65 | 1680.64 | | | 437.01 | 0.24 |
| Time (h) | Plasma concentration (ng/mL) | | | Brain concentration^c^ (ng/g) | | | | Brain to plasma ratio^d^ (B/P) | | |
| 0.083 | 1906.78±322.66 | | | 1680.64±34.23 | | | | 0.90±0.17 | | |
| 0.25 | 781.93±104.44 | | | 589.20±44.15 | | | | 0.76±0.04 | | |
| 0.5 | 283.70±45.52 | | | 233.82±31.73 | | | | 0.84±0.19 | | |
| 1 | 84.65±10.29 | | | 65.59±12.33 | | | | 0.79±0.25 | | |
| 2 | 15.51±7.09 | | | BLQ | | | | ND | | |
| 4 | 2.99±0.52 | | | BLQ | | | | ND | | |
| 8 | BLQ | | | BLQ | | | | ND | | |

^a^Mice (n=4) were dosed with 5 mg/kg for IV and 30 mg/kg for PO. Parameters were calculated from composite plasma concentration-time data. Data are expressed as the mean ± SD %.

^b^Mice (n=3) were dosed with 5 mg/kg by IV. Parameters were calculated from composite plasma concentration-time data.

^c^Brain concentration was determined by the amount of KDS12025 in total brain homogenate from mice (n=3/time point) after IV administration with 5 mg/kg)

^d^B/P: brain concentration/plasma concentration (IV administration with 5 mg/kg) calculated using 1 g/mL brain density

**Abbreviations**: *Cmpd*, compound; *AUC*, area under the plasma concentration-time curve; *Cl*, time-averaged total body clearance; *V_ss_*, apparent volume distribution at steady state; *t_1/2_*, elimination half-life; *C_max_*, maximum concentration of the drug; *F*, bioavailability; BLQ, below the lower limit of quantification (LLOQ); ND, not determined (Parameters not determined due to inadequately defined terminal elimination phase).

**Table S5. KINOMEscan screening results for KDS12025 interactions with 97 kinases**

| **Compound Name** | **Selectivity Score Type** | **Number of Hits** | **Number of Non-Mutant Kinases** | **Screening Concentration (nM)** | **Selectivity Score** |
| --- | --- | --- | --- | --- | --- |
| KDS12025 | S(35) | 0 | 90 | 10000 | 0 |
| KDS12025 | S(10) | 0 | 90 | 10000 | 0 |
| KDS12025 | S(1) | 0 | 90 | 10000 | 0 |

| **Compound Name** | **DiscoveRx Gene Symbol** | **Entrez Gene Symbol** | **Percent Control** | **Compound Concentration (nM)** |
| --- | --- | --- | --- | --- |
| KDS12025 | ABL1(E255K)-phosphorylated | ABL1 | 100 | 10000 |
| KDS12025 | ABL1(T315I)-phosphorylated | ABL1 | 84 | 10000 |
| KDS12025 | ABL1-nonphosphorylated | ABL1 | 72 | 10000 |
| KDS12025 | ABL1-phosphorylated | ABL1 | 100 | 10000 |
| KDS12025 | ACVR1B | ACVR1B | 100 | 10000 |
| KDS12025 | ADCK3 | CABC1 | 93 | 10000 |
| KDS12025 | AKT1 | AKT1 | 75 | 10000 |
| KDS12025 | AKT2 | AKT2 | 94 | 10000 |
| KDS12025 | ALK | ALK | 98 | 10000 |
| KDS12025 | AURKA | AURKA | 86 | 10000 |
| KDS12025 | AURKB | AURKB | 78 | 10000 |
| KDS12025 | AXL | AXL | 95 | 10000 |
| KDS12025 | BMPR2 | BMPR2 | 92 | 10000 |
| KDS12025 | BRAF | BRAF | 100 | 10000 |
| KDS12025 | BRAF(V600E) | BRAF | 100 | 10000 |
| KDS12025 | BTK | BTK | 100 | 10000 |
| KDS12025 | CDK11 | CDK19 | 73 | 10000 |
| KDS12025 | CDK2 | CDK2 | 92 | 10000 |
| KDS12025 | CDK3 | CDK3 | 79 | 10000 |
| KDS12025 | CDK7 | CDK7 | 89 | 10000 |
| KDS12025 | CDK9 | CDK9 | 83 | 10000 |
| KDS12025 | CHEK1 | CHEK1 | 82 | 10000 |
| KDS12025 | CSF1R | CSF1R | 100 | 10000 |
| KDS12025 | CSNK1D | CSNK1D | 88 | 10000 |
| KDS12025 | CSNK1G2 | CSNK1G2 | 45 | 10000 |
| KDS12025 | DCAMKL1 | DCLK1 | 82 | 10000 |
| KDS12025 | DYRK1B | DYRK1B | 67 | 10000 |
| KDS12025 | EGFR | EGFR | 48 | 10000 |
| KDS12025 | EGFR(L858R) | EGFR | 45 | 10000 |
| KDS12025 | EPHA2 | EPHA2 | 89 | 10000 |

**Table S5. KINOMEscan screening results for KDS12025 interactions with 97 kinases *(continued)***

| **Compound Name** | **DiscoveRx Gene Symbol** | **Entrez Gene Symbol** | **Percent Control** | **Compound Concentration (nM)** |
| --- | --- | --- | --- | --- |
| KDS12025 | ERBB2 | ERBB2 | 100 | 10000 |
| KDS12025 | ERBB4 | ERBB4 | 92 | 10000 |
| KDS12025 | ERK1 | MAPK3 | 83 | 10000 |
| KDS12025 | FAK | PTK2 | 100 | 10000 |
| KDS12025 | FGFR2 | FGFR2 | 87 | 10000 |
| KDS12025 | FGFR3 | FGFR3 | 91 | 10000 |
| KDS12025 | FLT3 | FLT3 | 100 | 10000 |
| KDS12025 | GSK3B | GSK3B | 95 | 10000 |
| KDS12025 | IGF1R | IGF1R | 84 | 10000 |
| KDS12025 | IKK-alpha | CHUK | 100 | 10000 |
| KDS12025 | IKK-beta | IKBKB | 100 | 10000 |
| KDS12025 | INSR | INSR | 85 | 10000 |
| KDS12025 | JAK2(JH1domain-catalytic) | JAK2 | 100 | 10000 |
| KDS12025 | JAK3(JH1domain-catalytic) | JAK3 | 76 | 10000 |
| KDS12025 | JNK1 | MAPK8 | 75 | 10000 |
| KDS12025 | JNK2 | MAPK9 | 91 | 10000 |
| KDS12025 | JNK3 | MAPK10 | 68 | 10000 |
| KDS12025 | KIT | KIT | 90 | 10000 |
| KDS12025 | KIT(D816V) | KIT | 91 | 10000 |
| KDS12025 | KIT(V559D,T670I) | KIT | 94 | 10000 |
| KDS12025 | LKB1 | STK11 | 80 | 10000 |
| KDS12025 | MAP3K4 | MAP3K4 | 100 | 10000 |
| KDS12025 | MAPKAPK2 | MAPKAPK2 | 75 | 10000 |
| KDS12025 | MARK3 | MARK3 | 66 | 10000 |
| KDS12025 | MEK1 | MAP2K1 | 82 | 10000 |
| KDS12025 | MEK2 | MAP2K2 | 91 | 10000 |
| KDS12025 | MET | MET | 82 | 10000 |
| KDS12025 | MKNK1 | MKNK1 | 100 | 10000 |
| KDS12025 | MKNK2 | MKNK2 | 95 | 10000 |
| KDS12025 | MLK1 | MAP3K9 | 79 | 10000 |
| KDS12025 | p38-alpha | MAPK14 | 100 | 10000 |
| KDS12025 | p38-beta | MAPK11 | 100 | 10000 |
| KDS12025 | PAK1 | PAK1 | 75 | 10000 |
| KDS12025 | PAK2 | PAK2 | 94 | 10000 |
| KDS12025 | PAK4 | PAK4 | 100 | 10000 |

**Table S5. KINOMEscan screening results for KDS12025 interactions with 97 kinases *(continued)***

| **Compound Name** | **DiscoveRx Gene Symbol** | **Entrez Gene Symbol** | **Percent Control** | **Compound Concentration (nM)** |
| --- | --- | --- | --- | --- |
| KDS12025 | PCTK1 | CDK16 | 94 | 10000 |
| KDS12025 | PDGFRA | PDGFRA | 85 | 10000 |
| KDS12025 | PDGFRB | PDGFRB | 92 | 10000 |
| KDS12025 | PDPK1 | PDPK1 | 56 | 10000 |
| KDS12025 | PIK3C2B | PIK3C2B | 91 | 10000 |
| KDS12025 | PIK3CA | PIK3CA | 63 | 10000 |
| KDS12025 | PIK3CG | PIK3CG | 100 | 10000 |
| KDS12025 | PIM1 | PIM1 | 95 | 10000 |
| KDS12025 | PIM2 | PIM2 | 64 | 10000 |
| KDS12025 | PIM3 | PIM3 | 84 | 10000 |
| KDS12025 | PKAC-alpha | PRKACA | 74 | 10000 |
| KDS12025 | PLK1 | PLK1 | 100 | 10000 |
| KDS12025 | PLK3 | PLK3 | 75 | 10000 |
| KDS12025 | PLK4 | PLK4 | 77 | 10000 |
| KDS12025 | PRKCE | PRKCE | 100 | 10000 |
| KDS12025 | RAF1 | RAF1 | 95 | 10000 |
| KDS12025 | RET | RET | 100 | 10000 |
| KDS12025 | RIOK2 | RIOK2 | 98 | 10000 |
| KDS12025 | ROCK2 | ROCK2 | 85 | 10000 |
| KDS12025 | RSK2(Kin.Dom.1-N-terminal) | RPS6KA3 | 84 | 10000 |
| KDS12025 | SNARK | NUAK2 | 100 | 10000 |
| KDS12025 | SRC | SRC | 72 | 10000 |
| KDS12025 | SRPK3 | SRPK3 | 91 | 10000 |
| KDS12025 | TGFBR1 | TGFBR1 | 96 | 10000 |
| KDS12025 | TIE2 | TEK | 100 | 10000 |
| KDS12025 | TRKA | NTRK1 | 100 | 10000 |
| KDS12025 | TSSK1B | TSSK1B | 45 | 10000 |
| KDS12025 | TYK2(JH1domain-catalytic) | TYK2 | 93 | 10000 |
| KDS12025 | ULK2 | ULK2 | 95 | 10000 |
| KDS12025 | VEGFR2 | KDR | 100 | 10000 |
| KDS12025 | YANK3 | STK32C | 83 | 10000 |
| KDS12025 | ZAP70 | ZAP70 | 88 | 10000 |
| KDS12025 | PCTK1 | CDK16 | 94 | 10000 |
| KDS12025 | PDGFRA | PDGFRA | 85 | 10000 |
| KDS12025 | PDGFRB | PDGFRB | 92 | 10000 |

**Table S6. KDS12025 interactions with 87 primary molecular targets including G protein–coupled receptors (GPCRs), kinases, non-kinase enzymes, nuclear receptors, transporters, and various ion channels**

| **Assay** | **Compound Name** | **Test Concentration** | **% Inhibition of Control Specific Binding** | | |
| --- | --- | --- | --- | --- | --- |
|  |  |  | **1st** | **2nd** | **Mean** |
| A1 (h) (antagonist radioligand) | KDS12025 | 1.0E-06 M | 114.7 | 113.5 | 114.1 |
| A2A (h) (agonist radioligand) | KDS12025 | 1.0E-06 M | 90.3 | 91.2 | 90.7 |
| alpha 1A (h) (antagonist radioligand) | KDS12025 | 1.0E-06 M | 87.6 | 86.5 | 87.1 |
| alpha 1B (h) (antagonist radioligand) | KDS12025 | 1.0E-06 M | 61.4 | 69.4 | 65.4 |
| alpha 1D (h) (antagonist radioligand) | KDS12025 | 1.0E-06 M | 75.3 | 76.3 | 75.8 |
| alpha 2A (h) (antagonist radioligand) | KDS12025 | 1.0E-06 M | 86.9 | 85.4 | 86.1 |
| alpha 2B (h) (antagonist radioligand) | KDS12025 | 1.0E-06 M | 81.9 | 86.6 | 84.2 |
| beta 1 (h) (agonist radioligand) | KDS12025 | 1.0E-06 M | 86.7 | 94.3 | 90.5 |
| beta 2 (h) (antagonist radioligand) | KDS12025 | 1.0E-06 M | 96.8 | 103.9 | 100.3 |
| AT1 (h) (antagonist radioligand) | KDS12025 | 1.0E-06 M | 82.3 | 92.5 | 87.4 |
| BZD (central) (agonist radioligand) | KDS12025 | 1.0E-06 M | 102.5 | 96.6 | 99.5 |
| Cl- channel (GABA-gated) (TBOB site) (antagonist radioligand) | KDS12025 | 1.0E-06 M | 103.8 | 89 | 96.4 |
| B2 (h) (agonist radioligand) | KDS12025 | 1.0E-06 M | 104.5 | 99.4 | 102 |
| CB1 (h) (agonist radioligand) | KDS12025 | 1.0E-06 M | 81.7 | 78.4 | 80 |
| CB2 (h) (agonist radioligand) | KDS12025 | 1.0E-06 M | 78.4 | 90.8 | 84.6 |
| CCK1 (CCKA) (h) (agonist radioligand) | KDS12025 | 1.0E-06 M | 103.7 | 104.5 | 104.1 |
| CCK2 (CCKB) (h) (agonist radioligand) | KDS12025 | 1.0E-06 M | 107.1 | 97 | 102.1 |
| D1 (h) (antagonist radioligand) | KDS12025 | 1.0E-06 M | 91.1 | 103.8 | 97.5 |
| D2S (h) (agonist radioligand) | KDS12025 | 1.0E-06 M | 64 | 59.8 | 61.9 |
| D2L (h) (antagonist radioligand) | KDS12025 | 1.0E-06 M | 74.8 | 93.2 | 84 |
| ETA (h) (agonist radioligand) | KDS12025 | 1.0E-06 M | 108 | 90.8 | 99.4 |
| GABAA1 (h) (alpha 1,beta 2,gamma 2) (agonist radioligand) | KDS12025 | 1.0E-06 M | 92.4 | 97.8 | 95.1 |
| GABAB(1b) (h) (antagonist radioligand) | KDS12025 | 1.0E-06 M | 106.5 | 100.7 | 103.6 |
| AMPA (agonist radioligand) | KDS12025 | 1.0E-06 M | 98 | 102.8 | 100.4 |
| kainate (agonist radioligand) | KDS12025 | 1.0E-06 M | 76.8 | 118.1 | 97.4 |
| NMDA (antagonist radioligand) | KDS12025 | 1.0E-06 M | 97.8 | 99.6 | 98.7 |
| mGluR5 (h) (agonist radioligand) | KDS12025 | 1.0E-06 M | 113.6 | 115.8 | 114.7 |
| glycine (strychnine-sensitive) (antagonist radioligand) | KDS12025 | 1.0E-06 M | 117.4 | 117.6 | 117.5 |
| glycine (strychnine-insensitive) (antagonist radioligand) | KDS12025 | 1.0E-06 M | 96.8 | 100.5 | 98.6 |
| CXCR2 (IL-8B) (h) (agonist radioligand) | KDS12025 | 1.0E-06 M | 91.8 | 83.3 | 87.6 |
| CCR1 (h) (agonist radioligand) | KDS12025 | 1.0E-06 M | 90.4 | 88.2 | 89.3 |

**Table S6. KDS12025 interactions with 87 primary molecular targets including G protein–coupled receptors (GPCRs), kinases, non-kinase enzymes, nuclear receptors, transporters, and various ion channels *(continued)***

| **Assay** | **Compound Name** | **Test Concentration** | **% Inhibition of Control Specific Binding** | | |
| --- | --- | --- | --- | --- | --- |
|  |  |  | **1st** | **2nd** | **Mean** |
| H1 (h) (antagonist radioligand) | KDS12025 | 1.0E-06 M | 87.1 | 89.4 | 88.2 |
| H2 (h) (antagonist radioligand) | KDS12025 | 1.0E-06 M | 112.8 | 94.2 | 103.5 |
| CysLT1 (LTD4) (h) (agonist radioligand) | KDS12025 | 1.0E-06 M | 83.1 | 91 | 87.1 |
| MC1 (agonist radioligand) | KDS12025 | 1.0E-06 M | 116.6 | 101.8 | 109.2 |
| MC4 (h) (agonist radioligand) | KDS12025 | 1.0E-06 M | 88.7 | 85.5 | 87.1 |
| MAO-A (antagonist radioligand) | KDS12025 | 1.0E-06 M | 95.5 | 105.1 | 100.3 |
| M1 (h) (antagonist radioligand) | KDS12025 | 1.0E-06 M | 97.8 | 85.9 | 91.8 |
| M2 (h) (antagonist radioligand) | KDS12025 | 1.0E-06 M | 104.1 | 110.2 | 107.1 |
| M3 (h) (antagonist radioligand) | KDS12025 | 1.0E-06 M | 88.2 | 82.7 | 85.5 |
| M4 (h) (antagonist radioligand) | KDS12025 | 1.0E-06 M | 99 | 104.5 | 101.7 |
| NK1 (h) (agonist radioligand) | KDS12025 | 1.0E-06 M | 92.2 | 90.1 | 91.2 |
| Y1 (h) (agonist radioligand) | KDS12025 | 1.0E-06 M | 105.5 | 102.1 | 103.8 |
| N neuronal alpha 4beta 2 (h) (agonist radioligand) | KDS12025 | 1.0E-06 M | 98.5 | 98.3 | 98.4 |
| N muscle-type (h) (antagonist radioligand) | KDS12025 | 1.0E-06 M | 68.5 | 80 | 74.3 |
| delta (DOP) (h) (agonist radioligand) | KDS12025 | 1.0E-06 M | 89.4 | 70 | 79.7 |
| kappa (h) (KOP) (agonist radioligand) | KDS12025 | 1.0E-06 M | 77.9 | 87.6 | 82.7 |
| mu (MOP) (h) (agonist radioligand) | KDS12025 | 1.0E-06 M | 85.5 | 83.5 | 84.5 |
| PPARgamma (h) (agonist radioligand) | KDS12025 | 1.0E-06 M | 94.7 | 104.6 | 99.7 |
| PAF (h) (agonist radioligand) | KDS12025 | 1.0E-06 M | 106 | 106.1 | 106 |
| PCP (antagonist radioligand) | KDS12025 | 1.0E-06 M | 94.6 | 89.4 | 92 |
| RARalpha (h) (agonist radioligand) | KDS12025 | 1.0E-06 M | 105.4 | 105 | 105.2 |
| 5-HT1A (h) (agonist radioligand) | KDS12025 | 1.0E-06 M | 65.1 | 59.6 | 62.4 |
| 5-HT1B (h) (antagonist radioligand) | KDS12025 | 1.0E-06 M | 86.8 | 94.7 | 90.8 |
| 5-HT2A (h) (agonist radioligand) | KDS12025 | 1.0E-06 M | 77.5 | 86.1 | 81.8 |
| 5-HT2B (h) (agonist radioligand) | KDS12025 | 1.0E-06 M | 65.9 | 61.8 | 63.9 |
| 5-HT2C (h) (antagonist radioligand) | KDS12025 | 1.0E-06 M | 79 | 73.8 | 76.4 |
| 5-HT3 (h) (antagonist radioligand) | KDS12025 | 1.0E-06 M | 91.4 | 100.5 | 96 |
| GR (h) (agonist radioligand) | KDS12025 | 1.0E-06 M | 104.3 | 103.4 | 103.9 |
| Estrogen ER alpha (h) (agonist radioligand) | KDS12025 | 1.0E-06 M | 109.2 | 81.5 | 95.3 |
| PR (h) (agonist radioligand) | KDS12025 | 1.0E-06 M | 105.3 | 102.8 | 104.1 |
| AR(h) (agonist radioligand) | KDS12025 | 1.0E-06 M | 94.2 | 87.5 | 90.9 |
| V1a (h) (agonist radioligand) | KDS12025 | 1.0E-06 M | 112.5 | 107.3 | 109.9 |

**Table S6. KDS12025 interactions with 87 primary molecular targets including G protein–coupled receptors (GPCRs), kinases, non-kinase enzymes, nuclear receptors, transporters, and various ion channels *(continued)***

| **Assay** | **Compound Name** | **Test Concentration** | **% Inhibition of Control Specific Binding** | | |
| --- | --- | --- | --- | --- | --- |
|  |  |  | **1st** | **2nd** | **Mean** |
| Ca2+ channel (L, dihydropyridine site) (antagonist radioligand) | KDS12025 | 1.0E-06 M | 137.5 | 121 | 129.3 |
| Ca2+ channel (L, diltiazem site) (benzothiazepines) (antagonist radioligand) | KDS12025 | 1.0E-06 M | 88.1 | 81.7 | 84.9 |
| Ca2+ channel (L, verapamil site) (phenylalkylamine) (antagonist radioligand) | KDS12025 | 1.0E-06 M | 100.6 | 95.5 | 98 |
| Ca2+ channel (N) (antagonist radioligand) | KDS12025 | 1.0E-06 M | 103.6 | 117.7 | 110.7 |
| Potassium Channel hERG (human)- [3H] Dofetilide | KDS12025 | 1.0E-06 M | 70.3 | 66.8 | 68.5 |
| KV channel (antagonist radioligand) | KDS12025 | 1.0E-06 M | 85.3 | 107.8 | 96.5 |
| Na+ channel (site 2) (antagonist radioligand) | KDS12025 | 1.0E-06 M | 83.6 | 65.5 | 74.5 |
| adenosine transporter (antagonist radioligand) | KDS12025 | 1.0E-06 M | 98.6 | 101.4 | 100 |
| norepinephrine transporter (h) (antagonist radioligand) | KDS12025 | 1.0E-06 M | 89.5 | 97 | 93.3 |
| dopamine transporter (h) (antagonist radioligand) | KDS12025 | 1.0E-06 M | 101.9 | 111.4 | 106.6 |
| GABA transporter (antagonist radioligand) | KDS12025 | 1.0E-06 M | 101 | 91.1 | 96 |
| 5-HT transporter (h) (antagonist radioligand) | KDS12025 | 1.0E-06 M | 62.9 | 46.4 | 54.6 |
| COX1(h) | KDS12025 | 1.00E-06 | 62.5 | 56.1 | 59.3 |
| COX2(h) | KDS12025 | 1.00E-06 | 42.2 | 45.3 | 43.8 |
| PDE3A (h) | KDS12025 | 1.00E-06 | 87.1 | 92.4 | 89.8 |
| PDE4D2 (h) | KDS12025 | 1.00E-06 | 95.3 | 102 | 98.7 |
| ACE (h) | KDS12025 | 1.00E-06 | 101.4 | 99.4 | 100.4 |
| cathepsin G (h) | KDS12025 | 1.00E-06 | 97.2 | 102.8 | 100 |
| IRK (h) (InsR) | KDS12025 | 1.00E-06 | 100.5 | 109.3 | 104.9 |
| Lck kinase (h) | KDS12025 | 1.00E-06 | 95.9 | 92.2 | 94.1 |
| PKCalpha (h) | KDS12025 | 1.00E-06 | 105.1 | 103 | 104 |
| acetylcholinesterase (h) | KDS12025 | 1.00E-06 | 92.6 | 90.8 | 91.7 |
| MAO-B (h) recombinant enzyme | KDS12025 | 1.00E-06 | 116.4 | 120.8 | 118.6 |
| ATPase (Na+/K+) | KDS12025 | 1.00E-06 | 97.8 | 97.8 | 97.8 |

**Table S7. Demographic information about human postmortem brain samples from normal subjects and AD patients**

| **Number** | **Case** | **Age** | **Sex** | **Braak Stage** |
| --- | --- | --- | --- | --- |
| **1** | Normal | 88 | M | I |
| **2** | Normal | 82 | M | I |
| **3** | Normal | 70 | M | I |
| **1** | AD | 89 | M | V |
| **2** | AD | 83 | M | VI |
| **3** | AD | 70 | M | VI |

**Table S8. Detailed statistics table**

| Figure No. | Detailed information for statistical analysis |
| --- | --- |
| 2b | Two-way ANOVA with Tukey's multiple comparison test, F (1,21) = 0.6535, p = 0.43 HRP: Vehicle (1.001±0.02269, n=3), HTPEB (3.769±0.7911, n=3), KDS12025 (5.642±0.5747, n=3) Vehicle vs. HTPEB, p = 0.03; Vehicle vs. KSD12025, p < 0.001; HTPEB vs. KDS12025, p = 0.17 Hb: Vehicle (1.001±0.01076, n=6), HTPEB (2.529±0.6645, n=6), KDS12025 (5.676±0.6374, n=6) Vehicle vs. HTPEB, p = 0.10; Vehicle vs. KSD12025, p < 0.001; HTPEB vs. KDS12025, p < 0.001 |
| 2d | Hb (0.01 U/ml): (-) (100.0±1.860, n=3), Hb+DMSO (97.77±0.9081, n=3),  Hb+HTPEB (84.92±3.034, n=3), Hb+KDS12025 (82.57±2.025, n=3)  One-way ANOVA with Tukey's multiple comparisons test, F(3,8) = 17.77, p < 0.001 (-) vs. Hb+DMSO, p = 0.87; (-) vs. Hb+HTPEB, p = 0.004; (-) vs. Hb+KDS12025, p = 0.002;  Hb+DMSO vs. Hb+HTPEB, p = 0.01; Hb+DMSO vs. Hb+KDS12025, p = 0.004; Hb+HTPEB vs. Hb+KDS12025, p = 0.86 Hb (0.1 U/ml): (-) (100.0±1.860, n=3), Hb+DMSO (95.19±1.581, n=3),  Hb+HTPEB (81.24±5.607, n=3), Hb+KDS12025 (75.36±1.113, n=3)  One-way ANOVA with Tukey's multiple comparisons test, F(3,8) = 13.84, p = 0.002 (-) vs. Hb+DMSO, p = 0.7; (-) vs. Hb+HTPEB, p = 0.01; (-) vs. Hb+KDS12025, p = 0.002;  Hb+DMSO vs. Hb+HTPEB, p = 0.05; Hb+DMSO vs. Hb+KDS12025, p = 0.009; Hb+HTPEB vs. Hb+KDS12025, p = 0.57 Hb (1 U/ml): (-) (100.0±5.136, n=3), Hb+DMSO (91.00±3.892, n=6),  Hb+HTPEB (70.82±3.83, n=6), Hb+KDS12025 (62.38±2.923, n=6)  One-way ANOVA with Tukey's multiple comparisons test, F(3,17) = 18.34, p < 0.001 (-) vs. Hb+DMSO, p = 0.49; (-) vs. Hb+HTPEB, p = 0.001; (-) vs. Hb+KDS12025, p < 0.001;  Hb+DMSO vs. Hb+HTPEB, p = 0.005; Hb+DMSO vs. Hb+KDS12025, p < 0.001; Hb+HTPEB vs. Hb+KDS12025, p = 0.37 Hb (11 U/ml): (-) (100.0±5.009, n=6), Hb+DMSO (64.68±6.780, n=9),  Hb+HTPEB (48.10±5.928, n=9), Hb+KDS12025 (30.17±2.742, n=9)  One-way ANOVA with Tukey's multiple comparisons test, F(3,29) = 25.49, p < 0.001 (-) vs. Hb+DMSO, p = 0.001; (-) vs. Hb+HTPEB, p < 0.001; (-) vs. Hb+KDS12025, p < 0.001;  Hb+DMSO vs. Hb+HTPEB, p = 0.14; Hb+DMSO vs. Hb+KDS12025, p < 0.001; Hb+HTPEB vs. Hb+KDS12025, p = 0.09 Hb (112 U/ml): (-) (100.0±5.968, n=3), Hb+DMSO (22.45±2.044, n=6),  Hb+HTPEB (13.83±1.029, n=6), Hb+KDS12025 (8.191±0.4716, n=6)  One-way ANOVA with Tukey's multiple comparisons test, F(3,17) = 289.3, p < 0.001 (-) vs. Hb+DMSO, p < 0.001; (-) vs. Hb+HTPEB, p < 0.001; (-) vs. Hb+KDS12025, p < 0.001;  Hb+DMSO vs. Hb+HTPEB, p = 0.03; Hb+DMSO vs. Hb+KDS12025, p < 0.001; Hb+HTPEB vs. Hb+KDS12025, p = 0.20 |
| 2g | CAT: Vehicle (93.8±11.09, n=8), HTPEB (84.5±9.957, n=8), KDS12025 (92.0±6.622, n=8)  One-way ANOVA with Tukey's multiple comparisons test, F(2,21) = 0.2723, p = 0.76 Vehicle vs. HTPEB, p = 0.77; Vehicle vs. KDS12025, p > 0.99; HTPEB vs. KDS12025, p = 0.84  HRP: Vehicle (7.25±0.479, n=4), HTPEB (9.0±1.0, n=4), KDS12025 (7.3±0.947, n=4)  One-way ANOVA with Tukey's multiple comparisons test, F(2,9) = 1.441, p = 0.29 Vehicle vs. HTPEB, p = 0.35; Vehicle vs. KDS12025, p > 0.99; HTPEB vs. KDS12025, p = 0.35  Hb: Vehicle (13.0±1.643, n=5), HTPEB (12.5±3.969, n=4), KDS12025 (13.5±2.533, n=4)  One-way ANOVA with Tukey's multiple comparisons test, F(2,10) = 0.03125, p = 0.96 Vehicle vs. HTPEB, p > 0.99; Vehicle vs. KDS12025, p > 0.99; HTPEB vs. KDS12025, p = 0.97 |
| 2j | Two-way ANOVA with Sidak's multiple comparison test, F (6,24) = 0.1095, p > 0.99 Full day: CTRL (3927, n=4), KDS12025 (10mpk) (3593, n=4), KDS12025 (1mpk) (3871, n=4), KDS12025 (0.1mpk) (4161, n=4) CTRL vs. KDS12025 (10mpk), p > 0.99; CTRL vs. KDS12025 (1mpk), p > 0.99; CTRL vs. KDS12025 (0.1mpk), p > 0.99; KDS12025 (10mpk) vs. KDS12025 (1mpk), p > 0.99; KDS12025 (10mpk) vs. KDS12025 (0.1mpk), p > 0.99; KDS12025 (1mpk) vs. KDS12025 (0.1mpk), p > 0.99 Dark: CTRL (4162, n=4), KDS12025 (10mpk) (4216, n=4), KDS12025 (1mpk) (4063, n=4), KDS12025 (0.1mpk) (4228, n=4) CTRL vs. KDS12025 (10mpk), p > 0.99; CTRL vs. KDS12025 (1mpk), p > 0.99; CTRL vs. KDS12025 (0.1mpk), p > 0.99; KDS12025 (10mpk) vs. KDS12025 (1mpk), p > 0.99; KDS12025 (10mpk) vs. KDS12025 (0.1mpk), p > 0.99; KDS12025 (1mpk) vs. KDS12025 (0.1mpk), p > 0.99 Light: CTRL (3752, n=4), KDS12025 (10mpk) (3753, n=4), KDS12025 (1mpk) (3584, n=4), KDS12025 (0.1mpk) (3923, n=4) CTRL vs. KDS12025 (10mpk), p > 0.99; CTRL vs. KDS12025 (1mpk), p > 0.99; CTRL vs. KDS12025 (0.1mpk), p > 0.99; KDS12025 (10mpk) vs. KDS12025 (1mpk), p > 0.99; KDS12025 (10mpk) vs. KDS12025 (0.1mpk), p > 0.99; KDS12025 (1mpk) vs. KDS12025 (0.1mpk), p > 0.99 |
| 2k | Two-way ANOVA with Sidak's multiple comparison test, F (6,24) = 0.1095, p > 0.99 Full day: CTRL (3072, n=4), KDS12025 (10mpk) (3120, n=4), KDS12025 (1mpk) (3075, n=4), KDS12025 (0.1mpk) (3292, n=4) CTRL vs. KDS12025 (10mpk), p > 0.99; CTRL vs. KDS12025 (1mpk), p > 0.99; CTRL vs. KDS12025 (0.1mpk), p > 0.99; KDS12025 (10mpk) vs. KDS12025 (1mpk), p > 0.99; KDS12025 (10mpk) vs. KDS12025 (0.1mpk), p > 0.99; KDS12025 (1mpk) vs. KDS12025 (0.1mpk), p > 0.99 Dark: CTRL (3299, n=4), KDS12025 (10mpk) (3344, n=4), KDS12025 (1mpk) (3167, n=4), KDS12025 (0.1mpk) (3345, n=4) CTRL vs. KDS12025 (10mpk), p > 0.99; CTRL vs. KDS12025 (1mpk), p > 0.99; CTRL vs. KDS12025 (0.1mpk), p > 0.99; KDS12025 (10mpk) vs. KDS12025 (1mpk), p > 0.99; KDS12025 (10mpk) vs. KDS12025 (0.1mpk), p > 0.99; KDS12025 (1mpk) vs. KDS12025 (0.1mpk), p > 0.99 Light: CTRL (2850, n=4), KDS12025 (10mpk) (2900, n=4), KDS12025 (1mpk) (2916, n=4), KDS12025 (0.1mpk) (3065, n=4) CTRL vs. KDS12025 (10mpk), p > 0.99; CTRL vs. KDS12025 (1mpk), p > 0.99; CTRL vs. KDS12025 (0.1mpk), p > 0.99; KDS12025 (10mpk) vs. KDS12025 (1mpk), p > 0.99; KDS12025 (10mpk) vs. KDS12025 (0.1mpk), p > 0.99; KDS12025 (1mpk) vs. KDS12025 (0.1mpk), p > 0.99 |
| 2l | Two-way ANOVA with Sidak's multiple comparison test, F (6,24) = 0.1095, p > 0.99 Full day: CTRL (0.834, n=4), KDS12025 (10mpk) (0.857, n=4), KDS12025 (1mpk) (0.883, n=4), KDS12025 (0.1mpk) (0.857, n=4) CTRL vs. KDS12025 (10mpk), p > 0.99; CTRL vs. KDS12025 (1mpk), p > 0.99; CTRL vs. KDS12025 (0.1mpk), p > 0.99; KDS12025 (10mpk) vs. KDS12025 (1mpk), p > 0.99; KDS12025 (10mpk) vs. KDS12025 (0.1mpk), p > 0.99; KDS12025 (1mpk) vs. KDS12025 (0.1mpk), p > 0.99 Dark: CTRL (0.858, n=4), KDS12025 (10mpk) (0.877, n=4), KDS12025 (1mpk) (0.891, n=4), KDS12025 (0.1mpk) (0.849, n=4) CTRL vs. KDS12025 (10mpk), p > 0.99; CTRL vs. KDS12025 (1mpk), p > 0.99; CTRL vs. KDS12025 (0.1mpk), p > 0.99; KDS12025 (10mpk) vs. KDS12025 (1mpk), p > 0.99; KDS12025 (10mpk) vs. KDS12025 (0.1mpk), p > 0.99; KDS12025 (1mpk) vs. KDS12025 (0.1mpk), p > 0.99 Light: CTRL (0.8033, n=4), KDS12025 (10mpk) (0.831, n=4), KDS12025 (1mpk) (0.860, n=4), KDS12025 (0.1mpk) (0.833, n=4) CTRL vs. KDS12025 (10mpk), p > 0.99; CTRL vs. KDS12025 (1mpk), p > 0.99; CTRL vs. KDS12025 (0.1mpk), p > 0.99; KDS12025 (10mpk) vs. KDS12025 (1mpk), p > 0.99; KDS12025 (10mpk) vs. KDS12025 (0.1mpk), p > 0.99; KDS12025 (1mpk) vs. KDS12025 (0.1mpk), p > 0.99 |
| 3d | Control (100.0±4.136, n=6), Aβ 0μM (89.89±4.975, n=3), Aβ 1μM (101.9±6.217, n=6),  Aβ 5μM (137.3±8.218, n=6), Aβ 10μM (155.2±9.442, n=6)  One-way ANOVA with Tukey's multiple comparisons test, F(4,22) = 13.74, p < 0.0001 Control vs. Aβ 0μM, p = 0.9182; Control vs. Aβ 1μM, p = 0.9997; Control vs. Aβ 5μM, p = 0.0088; Control vs. Aβ 10μM, p = 0.0001; Aβ 0μM vs. Aβ 1μM, p = 0.8587; Aβ 0μM vs. Aβ 5μM, p = 0.0064; Aβ 0μM vs. Aβ 10μM, p = 0.0002; Aβ 1μM vs. Aβ 5μM, p = 0.0137; Aβ 1μM vs. Aβ 10μM, p = 0.0002; Aβ 5μM vs. Aβ 10μM, p = 0.3971 |
| 3e | Control (100.0±3.409, n=12), Aβ (5μM) (138.5±4.632, n=10), Aβ+KDS12008 (10μM) (96.78±7.989, n=10),  Aβ+KDS12017 (10μM) (109.6±6.466, n=10), Aβ+KDS12025 (10μM) (110.3±5.804, n=11), Aβ+HTPEB (10μM) (112.0±8.321, n=8), Aβ+Sodium pyruvate (1mM) (149.1±6.194, n=5)  One-way ANOVA with Tukey's multiple comparisons test, F(6,59) = 8.225, p < 0.0001 CTRL vs. Aβ (5μM), p = 0.0003; CTRL vs. Aβ+KDS12008 (10μM), p = 0.99976; CTRL vs. Aβ+KDS12017 (10μM), p = 0.8993; CTRL vs. Aβ+KDS12025 (10μM), p = 0.8492; Aβ+HTPEB (10μM), p = 0.8079; Aβ+Sodium pyruvate (1mM), p = 0.0002; Aβ (5μM) vs. Aβ+KDS12008 (10μM), p = 0.0002; Aβ (5μM) vs. Aβ+KDS12017 (10μM), p = 0.0198; Aβ (5μM) vs. Aβ+KDS12025 (10μM), p = 0.0202; Aβ (5μM) vs. Aβ+HTPEB (10μM), p = 0.0672; Aβ (5μM) vs. Aβ+Sodium pyruvate (1mM), p = 0.9471; Aβ +KDS12008 (10μM) vs. Aβ+KDS12017 (10μM), p = 0.7391; Aβ+KDS12008 (10μM) vs. Aβ+KDS12025 (10μM), p = 0.6638; Aβ+KDS12008 (10μM) vs. Aβ+HTPEB (10μM), p = 0.6257; Aβ+KDS12008 (10μM) vs. Aβ+Sodium pyruvate (1mM), p < 0.0001; Aβ+KDS12017 (10μM) vs. Aβ+KDS12025 (10μM), p > 0.9999; Aβ+KDS12017 (10μM) vs. Aβ+HTPEB (10μM), p > 0.9999; Aβ+KDS12017 (10μM) vs. Aβ+Sodium pyruvate (1mM), p = 0.0061; Aβ+KDS12025 (10μM) vs. Aβ+HTPEB (10μM), p = 0.0064; Aβ+KDS12025 (10μM) vs. Aβ+Sodium pyruvate (10μM), p = 0.0184 |
| 3f | Control (100.0±3.367, n=4), Aβ 0μM (109.8±5.776, n=4), Aβ 1μM (109.4±4.271, n=4),  Aβ 5μM (140.5±4.407, n=4), Aβ 10μM (208.1±3.662, n=4)  One-way ANOVA with Tukey's multiple comparisons test, F(4,15) = 102.9, p < 0.0001 Control vs. Aβ 0μM, p = 0.5290; Control vs. Aβ 1μM, p = 0.5641; Control vs. Aβ 5μM, p < 0.0001; Control vs. Aβ 10μM, p < 0.0001; Aβ 0μM vs. Aβ 1μM, p > 0.9999; Aβ 0μM vs. Aβ 5μM, p = 0.0014; Aβ 0μM vs. Aβ 10μM, p < 0.0001; Aβ 1μM vs. Aβ 5μM, p = 0.0012; Aβ 1μM vs. Aβ 10μM, p < 0.0001; Aβ 5μM vs. Aβ 10μM, p < 0.0001 |
| 3h | Vehicle (91.88±9.525, n=12), Aβ (203.1±11.93, n=11), Aβ+KDS12025 (111.8±4.968, n=12),  Aβ+Sodium Pyruvate (137.5±9.751, n=10)  One-way ANOVA with Tukey's multiple comparisons test, F(3,41) = 27.64, p < 0.001 Vehicle vs. Aβ, p < 0.001; Vehicle vs. Aβ+KDS12025, p = 0.40; Vehicle vs. Aβ+Sodium Pyruvate, p = 0.007; Aβ vs. Aβ+KDS12025, p < 0.001; Aβ vs. Aβ+Sodium Pyruvate, p < 0.001; Aβ+KDS12025 vs. Aβ+Sodium Pyruvate, p = 0.23 |
| 3i | Vehicle (104.8±6.634, n=10), Putrescine (163.6±15.84, n=16), Put.+KDS12025 (96.12±6.634, n=28),  Put.+Sodium Pyruvate (189.0±23.55, n=10)  One-way ANOVA with Tukey's multiple comparisons test, F(3,60) = 13.61, p < 0.001 Vehicle vs. Putrescine, p = 0.02; Vehicle vs. Put.+KDS12025, p = 0.96; Vehicle vs. Put.+Sodium Pyruvate, p = 0.001; Putrescine vs. Put.+KDS12025, p < 0.001; Putrescine vs. Put.+Sodium Pyruvate, p = 0.55; Put.+KDS12025 vs. Put.+Sodium Pyruvate, p < 0.001 |
| 3j | 6-OHDA 0μM (120.2±14.1, n=5), 6-OHDA 10μM (153.8±24.7, n=5), 6-OHDA 30μM (211.9±18.2, n=5),  6-OHDA 50μM (271.2±14.8, n=5) One-way ANOVA with Tukey's multiple comparisons test, F(3,16) = 13.01, p = 0.0001 6-OHDA 0μM vs. 6-OHDA 10μM, p = 0.583; 6-OHDA 0μM vs. 6-OHDA 30μM, p = 0.014; 6-OHDA 0μM vs. 6-OHDA 50μM, p = 0.0001; 6-OHDA 10μM vs. 6-OHDA 30μM, p = 0.158; 6-OHDA 10μM vs. 6-OHDA 50μM, p = 0.002; 6-OHDA 30μM vs. 6-OHDA 50μM, p = 0.15 |
| 3k | Vehicle (117.0±9.2, n=9), 6-OHDA (205.3±12.3, n=14), 6-OHDA+KDS12025 (123.4±9.7, n=10), 6-OHDA+Sodium Pyruvate (177.0±17.5, n=7) One-way ANOVA with Tukey's multiple comparisons test, F(3,36) = 13.29, p < 0.001 Vehicle vs. 6-OHDA, p < 0.001; Vehicle vs. 6-OHDA+KDS12025, p = 0.98; Vehicle vs. 6-OHDA+Sodium Pyruvate, p = 0.02; 6-OHDA vs. 6-OHDA+KDS12025, p < 0.001; 6-OHDA vs. 6-OHDA+Sodium Pyruvate, p = 0.41; 6-OHDA+KDS12025 vs. 6-OHDA+Sodium Pyruvate, p = 0.04 |
| 4b | Two-way ANOVA with Tukey's multiple comparison test, F (2,378) = 37.72, p < 0.001 Acquisition: WT (18.68±8.178, n=6), APP (19.40±5.736, n=5), APP+KDS12025 (ad libitum) (21.17±3.843, n=6) WT vs. APP, p > 0.99; WT vs. APP+KDS12025 (ad libitum), p > 0.99; APP vs. APP+KDS12025 (ad libitum), p > 0.99 Retrieval: WT (271.5±76.57, n=6), APP (47.64±16.04, n=5), APP+KDS12025 (ad libitum) (254.3±56.96, n=6) WT vs. APP, p = 0.003; WT vs. APP+KDS12025 (ad libitum), p = 0.95; APP vs. APP+KDS12025 (ad libitum), p = 0.006 |
| 4c | WT (0.2560±0.07604, n=6), APP (-0.09560±0.05233, n=5), APP+KDS12025 (ad libitum) (0.2057±0.04843, n=6) One-way ANOVA with Tukey's multiple comparisons test, F(2,14) = 8.997, p = 0.003 WT vs. APP, p = 0.004; WT vs. APP+KDS12025 (ad libitum), p = 0.82; APP vs. APP+KDS12025 (ad libitum), p = 0.01 |
| 4e | fGcon (49.2±2.7, n=9), fGiD (82.5±3.6, n=9), fGiD+KDS (59.0±2.5, n=9) One-way ANOVA with Tukey's multiple comparisons test, F(2,24) = 33.23, p < 0.001 fGcon vs. fGiD, p < 0.01; fGcon vs. fGiD+KDS, p = 0.07; fGiD vs. fGiD+KDS12025 (0.1mpk), p = 0.01 |
| 4f | WT (0.96±0.04, n=6), APP (0.08±0.08, n=5), APP+KDS12025 (ad libitum) (0.733±0.1085, n=6) One-way ANOVA with Tukey's multiple comparisons test, F(2,13) = 26.68, p < 0.001 WT vs. APP, p < 0.001; WT vs. APP+KDS12025 (ad libitum), p = 0.18; APP vs. APP+KDS12025 (ad libitum), p < 0.001 |
| 4h | Control (225.3±30.6, n=6), A53T (93.8±19.6, n=7), A53T+KDS12025 (223.8±23.5, n=8) One-way ANOVA with Tukey's multiple comparisons test, F(2,18) = 9.52, p = 0.002 Control vs. A53T, p = 0.005; Control vs. A53T+KDS12025, p > 0.99; A53T vs. A53T+KDS12025, p = 0.003 |
| 4j | Control (26.81±3.748, n=35), A53T ipsi (57.94±4.409, n=91), A53T ipsi+KDS (34.18±3.214, n=53),  A53T contra (36±6.065, n=31), A53T contra+KDS (25.28±2.892, n=39) One-way ANOVA with Tukey's multiple comparisons test, F(4,244) = 11.18, p < 0.001 Control vs. A53T ipsi, p < 0.001; Control vs. A53T ipsi+KDS, p = 0.83; Control vs. A53T contra, p = 0.77; Control vs. A53T contra+KDS, p > 0.99; A53T ipsi vs. A53T ipsi+KDS, p < 0.001; A53T ipsi vs. A53T contra, p = 0.001; A53T ipsi vs. A53T contra+KDS, p < 0.001; A53T ipsi+KDS vs. A53T contra, p >0.99; A53T ipsi+KDS vs. A53T contra+KDS, p = 0.68; A53T contra vs. A53T contra+KDS, p = 0.63 |
| 4k | Control (12.16±1.171.59, n=35), A53T ipsi (73.73±10.13, n=34), A53T ipsi+KDS (5.531±0.9318, n=16),  A53T contra (4.673±1.833, n=8), A53T contra+KDS (10.18±1.460, n=18) One-way ANOVA with Tukey's multiple comparisons test, F(4,106) = 22.01, p < 0.001 Control vs. A53T ipsi, p < 0.001; Control vs. A53T ipsi+KDS, p = 0.96; Control vs. A53T contra, p = 0.98; Control vs. A53T contra+KDS, p > 0.99; A53T ipsi vs. A53T ipsi+KDS, p < 0.001; A53T ipsi vs. A53T contra, p < 0.001; A53T ipsi vs. A53T contra+KDS, p < 0.001; A53T ipsi+KDS vs. A53T contra, p >0.99; A53T ipsi+KDS vs. A53T contra+KDS, p > 0.99; A53T contra vs. A53T contra+KDS, p > 0.99 |
| 4l | Control (30.24±4.6, n=5), A53T ipsi (7.112±2.209, n=5), A53T ipsi+KDS (20.96±2.68, n=7),  A53T contra (23.62±2.751, n=5), A53T contra+KDS (23.20±2.685, n=6) One-way ANOVA with Tukey's multiple comparisons test, F(4,23) = 7.027, p < 0.001 Control vs. A53T ipsi, p < 0.001; Control vs. A53T ipsi+KDS, p = 0.21; Control vs. A53T contra, p = 0.60; Control vs. A53T contra+KDS, p = 0.50; A53T ipsi vs. A53T ipsi+KDS, p = 0.02; A53T ipsi vs. A53T contra, p = 0.01; A53T ipsi vs. A53T contra+KDS, p = 0.01; A53T ipsi+KDS vs. A53T contra, p = 0.97; A53T ipsi+KDS vs. A53T contra+KDS, p = 0.98; A53T contra vs. A53T contra+KDS, p > 0.99 |
| 4n | Two-way ANOVA with Tukey's multiple comparison test, F (42,303) = 3.645, p < 0.001 15wk: Control (n=5), SOD1 (n=6), SOD1+KDS12025 1mpk (n=7), SOD1+KDS12025 10mpk (n=7) Control vs. SOD1, p = 0.0014; Control vs. SOD1+KDS12025 1mpk, p = 0.9366; Control vs. SOD1+KDS12025 10mpk, p = 0.306 10wk: Control vs. SOD1, p > 0.9999; Control vs. SOD1+KDS12025 1mpk, p > 0.9999; Control vs. SOD1+KDS12025 10mpk, p = 0.7843 11wk: Control vs. SOD1, p = 0.4748; Control vs. SOD1+KDS12025 1mpk, p = 0.1726; Control vs. SOD1+KDS12025 10mpk, p > 0.9999 12wk: Control vs. SOD1, p = 0.0727; Control vs. SOD1+KDS12025 1mpk, p > 0.9999; Control vs. SOD1+KDS12025 10mpk, p = 0.3824 13wk: Control vs. SOD1, p = 0.5437; Control vs. SOD1+KDS12025 1mpk, p = 0.9744; Control vs. SOD1+KDS12025 10mpk, p > 0.9999 14wk: Control vs. SOD1, p = 0.3696; Control vs. SOD1+KDS12025 1mpk, p > 0.9999; Control vs. SOD1+KDS12025 10mpk, p = 0.8831 15wk: Control vs. SOD1, p = 0.0014; Control vs. SOD1+KDS12025 1mpk, p = 0.9366; Control vs. SOD1+KDS12025 10mpk, p = 0.3060 16wk: Control vs. SOD1, p = 0.0201; Control vs. SOD1+KDS12025 1mpk, p = 0.9991; Control vs. SOD1+KDS12025 10mpk, p = 0.2009 17wk: Control vs. SOD1, p = 0.0007; Control vs. SOD1+KDS12025 1mpk, p = 0.9962; Control vs. SOD1+KDS12025 10mpk, p > 0.9999 18wk: Control vs. SOD1, p = 0.0201; Control vs. SOD1+KDS12025 1mpk, p = 0.9991; Control vs. SOD1+KDS12025 10mpk, p = 0.2009 19wk: Control vs. SOD1, p = 0.0007; Control vs. SOD1+KDS12025 1mpk, p = 0.9962; Control vs. SOD1+KDS12025 10mpk, p > 0.9999 20wk: Control vs. SOD1, p < 0.0001; Control vs. SOD1+KDS12025 1mpk, p = 0.7111; Control vs. SOD1+KDS12025 10mpk, p = 0.3060 21wk: Control vs. SOD1, p < 0.0001; Control vs. SOD1+KDS12025 1mpk, p = 0.2127; Control vs. SOD1+KDS12025 10mpk, p = 0.1706 22wk: Control vs. SOD1, p < 0.0001; Control vs. SOD1+KDS12025 1mpk, p < 0.0001; Control vs. SOD1+KDS12025 10mpk, p = 0.0002 23wk: Control vs. SOD1, p < 0.0001; Control vs. SOD1+KDS12025 1mpk, p < 0.0001; Control vs. SOD1+KDS12025 10mpk, p < 0.0001 24wk: Control vs. SOD1, p = 0.0002; Control vs. SOD1+KDS12025 1mpk, p < 0.0001; Control vs. SOD1+KDS12025 10mpk, p < 0.0001 |
| 4o | Log-rank (Mantel-Cox) text; Chi square: 28.14, df=3, p <0.001 Control (median survival: undefined, n = 4), SOD1 (median survival: 140, n = 7), SOD1+KDS12025 1mpk (median survival: 168, n = 7), SOD1+KDS12025 10mpk (median survival: 168, n = 7) |
| 5b | Two-way ANOVA with Tukey's multiple comparison test, F (1,350) = 600.9, p < 0.001 CA1: Normal (1.0±0.1025, n=30), AD (0.1101±0.01655, n=30); Normal vs. AD, p < 0.001 CA2: Normal (1.0±0.0761, n=30), AD (0.1514±0.02163, n=30); Normal vs. AD, p < 0.001 CA3: Normal (1.0±0.09249, n=30), AD (0.0888±0.01051, n=30); Normal vs. AD, p < 0.001 CA4: Normal (1.0±0.09124, n=30), AD (0.1165±0.01455, n=30); Normal vs. AD, p < 0.001 DG: Normal (1.0±0.07573, n=30), AD (0.07965±0.01313, n=30); Normal vs. AD, p < 0.001 Ent Cx: Normal (1.0±0.08698, n=30), AD (0.1148±0.01699, n=30); Normal vs. AD, p < 0.001 |
| 5e | WT (70.03±1.523, n=55), APP (62.3±1.219, n=65), APP+KDS12025 (ad libitum) (68.67±2.358, n=54) One-way ANOVA with Tukey's multiple comparisons test, F(2,171) = 6.123, p = 0.003 WT vs. APP, p = 0.004; WT vs. APP+KDS12025 (ad libitum), p = 0.85; APP vs. APP+KDS12025 (ad libitum), p = 0.02 |
| 5f | WT (61.01±2.28, n=6), APP (114.3±9.536, n=6), APP+KDS12025 (ad libitum) (64.48±6.98, n=6) One-way ANOVA with Tukey's multiple comparisons test, F(2,14) = 17.24, p < 0.001 WT vs. APP, p < 0.001; WT vs. APP+KDS12025 (ad libitum), p = 0.89; APP vs. APP+KDS12025 (ad libitum), p < 0.001 |
| 5g | WT (31.45±3.606, n=11), APP (63.19±5.017, n=16), APP+KDS12025 (ad libitum) (33.69±3.3, n=13) One-way ANOVA with Tukey's multiple comparisons test, F(2,37) = 18.10, p < 0.001 WT vs. APP, p < 0.001; WT vs. APP+KDS12025 (ad libitum), p = 0.94; APP vs. APP+KDS12025 (ad libitum), p < 0.001 |
| 5h | WT (1.617±0.2177, n=11), APP (7.134±1.523, n=16), APP+KDS12025 (ad libitum) (2.808±0.6584, n=13) One-way ANOVA with Tukey's multiple comparisons test, F(2,37) = 6.945, p = 0.003 WT vs. APP, p = 0.004; WT vs. APP+KDS12025 (ad libitum), p = 0.76; APP vs. APP+KDS12025 (ad libitum), p = 0.02 |
| 5i | WT (110.0±6.606, n=11), APP (135.6±5.625, n=16), APP+KDS12025 (ad libitum) (103.1±7.794, n=13) One-way ANOVA with Tukey's multiple comparisons test, F(2,37) = 7.249, p = 0.002 WT vs. APP, p = 0.03; WT vs. APP+KDS12025 (ad libitum), p = 0.77; APP vs. APP+KDS12025 (ad libitum), p = 0.003 |
| 5j | Two-way ANOVA with Tukey's multiple comparison test, F (2,378) = 37.72, p < 0.001 WT (2.028, n=12), APP (4.318, n=17), APP+KDS12025 (ad libitum) (2.392, n=12) WT vs. APP, p < 0.001 (***); WT vs. APP+KDS12025 (ad libitum), p = 0.52; APP vs. APP+KDS12025 (ad libitum), p < 0.001 (^###^) |
| 5l | Left: Two-way ANOVA with Tukey's multiple comparison test, F (2,378) = 37.72, p < 0.001 WT (0.8145±0.09924, n=11), APP (0.4109±0.1036, n=11), APP+KDS12025 (ad libitum) (0.7455±0.1216, n=11) WT vs. APP, p < 0.001; WT vs. APP+KDS12025 (ad libitum), p = 0.16; APP vs. APP+KDS12025 (ad libitum), p < 0.001 Right: WT (0.96±0.04, n=6), APP (0.08±0.08, n=5), APP+KDS12025 (ad libitum) (0.733±0.1085, n=6) One-way ANOVA with Tukey's multiple comparisons test, F(2,13) = 26.68, p < 0.001 WT vs. APP, p < 0.001; WT vs. APP+KDS12025 (ad libitum), p = 0.18; APP vs. APP+KDS12025 (ad libitum), p < 0.001 |
| 5m | Two-way ANOVA with Tukey's multiple comparison test, F (2,378) = 37.72, p < 0.001 Acquisition: WT (18.68±8.178, n=6), APP (19.40±5.736, n=5), APP+KDS12025 (ad libitum) (21.17±3.843, n=6) WT vs. APP, p > 0.99; WT vs. APP+KDS12025 (ad libitum), p > 0.99; APP vs. APP+KDS12025 (ad libitum), p > 0.99 Retrieval: WT (271.5±76.57, n=6), APP (47.64±16.04, n=5), APP+KDS12025 (ad libitum) (254.3±56.96, n=6) WT vs. APP, p = 0.003; WT vs. APP+KDS12025 (ad libitum), p = 0.95; APP vs. APP+KDS12025 (ad libitum), p = 0.006 |
| 5n | WT (0.2560±0.07604, n=6), APP (-0.09560±0.05233, n=5), APP+KDS12025 (ad libitum) (0.2057±0.04843, n=6) One-way ANOVA with Tukey's multiple comparisons test, F(2,14) = 8.997, p = 0.003 WT vs. APP, p = 0.004; WT vs. APP+KDS12025 (ad libitum), p = 0.82; APP vs. APP+KDS12025 (ad libitum), p = 0.01 |
| 5p | Control (26.40±1.082, n=61), A53T ipsi (44.57±2.459, n=31), A53T ipsi+KDS12025 (34.98±2.564, n=20), A53T contra (45.24±4.064, n=19), A53T contra+KDS12025 (46.92±4.141, n=12) One-way ANOVA with Tukey's multiple comparisons test, F(4,138) = 18.62, p < 0.001 Control vs. A53T ipsi, p < 0.001; Control vs. A53T ipsi+KDS12025, p = 0.98; Control vs. A53T contra, p = 0.06; Control vs. A53T contra+KDS12025, p > 0.99; A53T ipsi vs. A53T ipsi+KDS12025, p < 0.001; A53T ipsi vs. A53T contra, p = 0.05; A53T ipsi vs. A53T contra+KDS12025, p < 0.0001; A53T ipsi+KDS12025 vs. A53T contra, p = 0.05; A53T ipsi+KDS12025 vs. A53T contra+KDS12025, p > 0.99; A53T contra vs. A53T contra+KDS12025, p = 0.07 |
| 5q | Control (65.22±6.586, n=14), A53T ipsi (60.57±4.01, n=13), A53T ipsi+KDS12025 (55.65±3.378, n=28), A53T contra (60.30±3.44, n=39), A53T contra+KDS12025 (61.58±2.401, n=38) One-way ANOVA with Tukey's multiple comparisons test, F(4,127) = 0.8159, p = 0.58 Control vs. A53T ipsi, p = 0.97; Control vs. A53T ipsi+KDS12025, p = 0.53; Control vs. A53T contra, p = 0.92; Control vs. A53T contra+KDS12025, p = 0.97; A53T ipsi vs. A53T ipsi+KDS12025, p = 0.94; A53T ipsi vs. A53T contra, p > 0.99; A53T ipsi vs. A53T contra+KDS12025, p > 0.99; A53T ipsi+KDS12025 vs. A53T contra, p = 0.85; A53T ipsi+KDS12025 vs. A53T contra+KDS12025, p = 0.71; A53T contra vs. A53T contra+KDS12025, p > 0.99 |
| 6b | Unpaired t-test, two-tailed  Sc-shRNA (4386±265.6, n=20) vs. Hbβ (1665±134.9, n=18), p<0.001, t=8.83, df=36 |
| 6c | Two-way ANOVA with Tukey's multiple comparison test, F (1,42) = 37.70, p < 0.001 Sc-sh: Vehicle (100.0±0.9850, n=8), Aβ42 (125.7±2.521, n=8), Aβ42+KDS12025 (105.8±2.173, n=8) Vehicle vs. Aβ42, p < 0.001; Vehicle vs. Aβ42+KDS12025, p = 0.40; Aβ42 vs. Aβ42+KDS12025, p < 0.001 Hbβ-Sh: Vehicle (100.0±2.506, n=8), Aβ42 (144.7.7±3.744, n=8), Aβ42+KDS12025 (134.1±5.185, n=8) Vehicle vs. Aβ42, p < 0.001; Vehicle vs. Aβ42+KDS12025, p < 0.001; Aβ42vs. Aβ42+KDS12025, p = 0.05 |
| 6e | Two-way ANOVA with Tukey's multiple comparison test, F (4,90) = 8.83, p < 0.001 Sc-sh: Control (86.8±16.8, n=8), Aβ (217.6±18.6, n=11), Aβ+KDS12025 0.1μM (100.5±13.5, n=9), Aβ+KDS12025 1μM (112.3±24.1, n=11), Aβ+KDS12025 10μM (83.4±13.5, n=8)  Hbβ-Sh: Control (107.8±, n=8), Aβ (232.0±, n=11), Aβ+KDS12025 0.1μM (199.3±, n=9), Aβ+KDS12025 1μM (204.7±, n=11), Aβ+KDS12025 10μM (182.4±, n=8)  Control, p = 0.99; Aβ, p > 0.99; Aβ+KDS12025 0.1μM, p = 0.02; Aβ+KDS12025 1μM, p = 0.01; Aβ+KDS12025 10μM, p = 0.01 |
| 6g | WT (0.33±0.057, n=6), APP (-0.097.±0.052, n=6), APP+shHbβ+KDS12025 (-0.06±0.082, n=7), APP+ScRNA+KDS12025 (0.46±0.09, n=6) One-way ANOVA with Tukey's multiple comparisons test, F(3,20) = 13.30, p < 0.001 WT vs. APP, p = 0.004; WT vs. APP+shHbβ+KDS12025, p = 0.004; WT vs. APP+ScRNA+KDS12025, p = 0.46; APP vs. APP+shHbβ+KDS12025, p = 0.75; APP vs. APP+ScRNA+KDS12025, p < 0.001; APP+shHbβ+KDS12025 vs. APP+ScRNA+KDS12025, p < 0.001 |
| 6h | WT (11.25±0.6627, n=47), APP (8.798±0.5302, n=54),  APP+shHbβ+KDS12025 (6.275±0.4473, n=83), APP+ScRNA+KDS12025 (10.10±0.4531, n=96) One-way ANOVA with Tukey's multiple comparisons test, F(3,276) = 17.89, p < 0.001 WT vs. APP, p = 0.02; WT vs. APP+shHbβ+KDS12025, p < 0.001; WT vs. APP+ScRNA+KDS12025, p = 0.42; APP vs. APP+shHbβ+KDS12025, p = 0.004; APP vs. APP+ScRNA+KDS12025, p = 0.28; APP+shHbβ+KDS12025 vs. APP+ScRNA+KDS12025, p < 0.001 |
| 6i | WT (117.1±19.54, n=21), APP (203.7±22.90, n=18),  APP+shHbβ+KDS12025 (234.3±16.93, n=26), APP+ScRNA+KDS12025 (142.4±8.715, n=35) One-way ANOVA with Tukey's multiple comparisons test, F(3,99) = 61.21, p < 0.001 WT vs. APP, p = 0.005; WT vs. APP+shHbβ+KDS12025, p < 0.001; WT vs. APP+ScRNA+KDS12025, p = 0.65; APP vs. APP+shHbβ+KDS12025, p = 0.59; APP vs. APP+ScRNA+KDS12025, p = 0.04; APP+shHbβ+KDS12025 vs. APP+ScRNA+KDS12025, p < 0.001 |
| 6j | WT (117.8±2.617, n=21), APP (156.1±2.347, n=20),  APP+shHbβ+KDS12025 (146.8±2.262, n=27), APP+ScRNA+KDS12025 (124.3±1.773, n=35) One-way ANOVA with Tukey's multiple comparisons test, F(3,99) = 61.21, p < 0.001 WT vs. APP, p < 0.001; WT vs. APP+shHbβ+KDS12025, p < 0.001; WT vs. APP+ScRNA+KDS12025, p = 0.16; APP vs. APP+shHbβ+KDS12025, p = 0.03; APP vs. APP+ScRNA+KDS12025, p < 0.001; APP+shHbβ+KDS12025 vs. APP+ScRNA+KDS12025, p < 0.001 |
| 6l | WT (0.24±0.04, n=4), APP+Sc-shRNA (-0.34±0.064, n=4), APP+shHbβ::GFAP+KDS12025 (-0.04±0.059, n=4), APP+shHbβ::CaMKII+KDS12025 (0.24±0.08, n=3) One-way ANOVA with Tukey's multiple comparisons test, F(3,20) = 13.30, p < 0.001 WT vs. APP+Sc-shRNA, p = 0.57; WT vs. APP+shHbβ::GFAP+KDS12025, p = 0.04; WT vs. APP+shHbβ::CaMKII+KDS12025, p = 0.96; APP+Sc-shRNA vs. APP+shHbβ::GFAP+KDS12025, p = 0.005; APP+Sc-shRNA vs. APP+shHbβ::CaMKII+KDS12025, p = 0.57; APP+shHbβ::GFAP+KDS12025 vs. APP+shHbβ::CaMKII+KDS12025, p = 0.04 |
| 6m | WT (101.6±2.91, n=28), APP+Sc-shRNA (99.82±1.97, n=63), APP+shHbβ::GFAP+KDS12025 (87.46±1.26, n=34), APP+shHbβ::CaMKII+KDS12025 (110.5±1.59, n=32) One-way ANOVA with Tukey's multiple comparisons test, F(3,153) = 17.66, p < 0.001 WT vs. APP+Sc-shRNA, p = 0.56; WT vs. APP+shHbβ::GFAP+KDS12025, p < 0.001; WT vs. APP+shHbβ::CaMKII+KDS12025, p = 0.02; APP+Sc-shRNA vs. APP+shHbβ::GFAP+KDS12025, p < 0.001; APP+Sc-shRNA vs. APP+shHbβ::CaMKII+KDS12025, p < 0.001; APP+shHbβ::GFAP+KDS12025 vs. APP+shHbβ::CaMKII+KDS12025, p < 0.001 |
| 6n | WT (59.47±5,16, n=28), APP+Sc-shRNA (86.49±5.59, n=63), APP+shHbβ::GFAP+KDS12025 (134.3±10.91, n=34), APP+shHbβ::CaMKII+KDS12025 (55.98±5.45, n=32) One-way ANOVA with Tukey's multiple comparisons test, F(3,153) = 21.40, p < 0.001 WT vs. APP+Sc-shRNA, p = 0.56; WT vs. APP+shHbβ::GFAP+KDS12025, p < 0.001; WT vs. APP+shHbβ::CaMKII+KDS12025, p = 0.02; APP+Sc-shRNA vs. APP+shHbβ::GFAP+KDS12025, p < 0.001; APP+Sc-shRNA vs. APP+shHbβ::CaMKII+KDS12025, p < 0.001; APP+shHbβ::GFAP+KDS12025 vs. APP+shHbβ::CaMKII+KDS12025, p < 0.001 |
| 6o | WT (114.2±4.70, n=28), APP+Sc-shRNA (90.67±3.32, n=63), APP+shHbβ::GFAP+KDS12025 (152.1±5.50, n=34), APP+shHbβ::CaMKII+KDS12025 (131.4±3.56, n=32) One-way ANOVA with Tukey's multiple comparisons test, F(3,153) = 44.37, p < 0.001 WT vs. APP+Sc-shRNA, p < 0.001; WT vs. APP+shHbβ::GFAP+KDS12025, p < 0.001; WT vs. APP+shHbβ::CaMKII+KDS12025, p = 0.01; APP+Sc-shRNA vs. APP+shHbβ::GFAP+KDS12025, p < 0.001; APP+Sc-shRNA vs. APP+shHbβ::CaMKII+KDS12025, p < 0.001; APP+shHbβ::GFAP+KDS12025 vs. APP+shHbβ::CaMKII+KDS12025, p = 0.003 |
| 6q | WT (61.5±21.25, n=4), A53T+Sc-shRNA (179.8±36.5, n=4), A53T+shHbβ::GFAP+KDS12025 (117.0±63.6, n=3), A53T+shHbβ::CaMKII+KDS12025 (139.0±51.1, n=3) One-way ANOVA with Tukey's multiple comparisons test, F(3,10) = 1.57, p = 0.26 WT vs. A53T+Sc-shRNA, p < 0.001; WT vs. A53T+shHbβ::GFAP+KDS12025, p < 0.001; WT vs. A53T+shHbβ::CaMKII+KDS12025, p = 0.01; A53T+Sc-shRNA vs. A53T+shHbβ::GFAP+KDS12025, p < 0.001; A53T+Sc-shRNA vs. A53T+shHbβ::CaMKII+KDS12025, p < 0.001; A53T+shHbβ::GFAP+KDS12025 vs. A53T+shHbβ::CaMKII+KDS12025, p = 0.003 |
| 6r | WT (49.3±4.91, n=33), A53T+Sc-shRNA (24.12±2.99, n=30), A53T+shHbβ::GFAP+KDS12025 (42.7±5.05, n=27), A53T+shHbβ::CaMKII+KDS12025 (37.16±3.6, n=45) One-way ANOVA with Tukey's multiple comparisons test, F(3,131) = 5.99, p < 0.001 WT vs. A53T+Sc-shRNA, p < 0.001; WT vs. A53T+shHbβ::GFAP+KDS12025, p = 0.72; WT vs. A53T+shHbβ::CaMKII+KDS12025, p = 0.13; A53T+Sc-shRNA vs. A53T+shHbβ::GFAP+KDS12025, p = 0.02; A53T+Sc-shRNA vs. A53T+shHbβ::CaMKII+KDS12025, p = 0.11; A53T+shHbβ::GFAP+KDS12025 vs. A53T+shHbβ::CaMKII+KDS12025, p = 0.79 |
| 6s | WT (3.6±1.7, n=5), A53T+Sc-shRNA (23.25±1.9, n=4), A53T+shHbβ::GFAP+KDS12025 (16.75±2.75, n=4), A53T+shHbβ::CaMKII+KDS12025 (18.5±1.3, n=4) One-way ANOVA with Tukey's multiple comparisons test, F(3,13) = 20.12, p < 0.001 WT vs. A53T+Sc-shRNA, p < 0.001; WT vs. A53T+shHbβ::GFAP+KDS12025, p = 0.002; WT vs. A53T+shHbβ::CaMKII+KDS12025, p < 0.001; A53T+Sc-shRNA vs. A53T+shHbβ::GFAP+KDS12025, p = 0.15; A53T+Sc-shRNA vs. A53T+shHbβ::CaMKII+KDS12025, p = 0.38; A53T+shHbβ::GFAP+KDS12025 vs. A53T+shHbβ::CaMKII+KDS12025, p = 0.93 |
| 7c | 12 month (6.5±0.25, n=10), 26 month (4.64±0.5, n=6), 26 month+KDS 0.1mpk (5.42±0.34, n=5), 26 month+KDS1mpk (6.24±0.6, n=5) One-way ANOVA with Tukey's multiple comparisons test, F(3,22) = 5.04, p = 0.008 12 month vs. 26 month, p = 0.007; 12 month vs. 26 month+KDS 0.1mpk, p = 0.21; 12 month vs. 26 month+KDS 1mpk, p = 0.96; 26 month vs. 26 month+KDS 0.1mpk, p = 0.57; 26 month vs. 26 month+KDS 1mpk, p = 0.06; 26 month+KDS 0.1mpk vs. 26 month+KDS 1mpk, p = 0.56 |
| 7d | 12 month (3905±148, n=10), 26 month (2793±269, n=6), 26 month+KDS 0.1mpk (3262±203, n=5), 26 month+KDS1mpk (3600±561, n=5) One-way ANOVA with Tukey's multiple comparisons test, F(3,22) = 3.2, p = 0.04 12 month vs. 26 month, p = 0.03; 12 month vs. 26 month+KDS 0.1mpk, p = 0.38; 12 month vs. 26 month+KDS 1mpk, p = 0.86; 26 month vs. 26 month+KDS 0.1mpk, p = 0.71; 26 month vs. 26 month+KDS 1mpk, p = 0.27; 26 month+KDS 0.1mpk vs. 26 month+KDS 1mpk, p = 0.88 |
| 7f | 18 month (1694642±125526, n=6), 30 month (1149296±45876, n=4), 30 month+KDS (1820376±163596, n=6) One-way ANOVA with Tukey's multiple comparisons test, F(2,13) = 5.78, p = 0.02 18 month vs. 30 month, p = 0.05; 18 month vs. 30 month+KDS, p = 0.77; 30 month vs. 30 month+KDS, p = 0.02 |
| 7g | 18 month (272.5±20.8, n=44), 30 month (167.6±12, n=60), 30 month+KDS (212.0±12.4, n=76) One-way ANOVA with Tukey's multiple comparisons test, F(2,177) = 11.24, p < 0.001 18 month vs. 30 month, p < 0.001; 18 month vs. 30 month+KDS, p = 0.01; 30 month vs. 30 month+KDS, p = 0.06 |
| 7h | 18 month (84.3±5.19, n=8), 30 month (44.4±5.44, n=9), 30 month+KDS (63.6±5.78, n=14) One-way ANOVA with Tukey's multiple comparisons test, F(2,28) = 9.65, p < 0.001 18 month vs. 30 month, p < 0.001; 18 month vs. 30 month+KDS, p = 0.05; 30 month vs. 30 month+KDS, p = 0.06 |
| 7j | Two-way ANOVA with Dunnett's multiple comparison test, F (2, 1050) = 38.44, p < 0.001  18 month vs. 30 month, p < 0.001, 18 month vs 30 month+KDS, p < 0.001 |
| 7l | 18 month (56.4±1.04, n=48), 30 month (49.5±1.7, n=40), 30 month+KDS (62.8±1.32, n=32) One-way ANOVA with Tukey's multiple comparisons test, F(2,117) = 21.5, p < 0.001 18 month vs. 30 month, p < 0.001; 18 month vs. 30 month+KDS, p = 0.004; 30 month vs. 30 month+KDS, p < 0.001 |
| S8b | Two-way ANOVA with Sidak's multiple comparison test, F (6,24) = 0.2512, p = 0.95 Full day: CTRL (18.99±0.3193, n=4), KDS12025 (10mpk) (19.14±0.6082, n=4), KDS12025 (1mpk) (21.26±0.07088, n=4), KDS12025 (0.1mpk) (22.21±1.751, n=4) CTRL vs. KDS12025 (10mpk), p > 0.99; CTRL vs. KDS12025 (1mpk), p = 0.83; CTRL vs. KDS12025 (0.1mpk), p = 0.31; KDS12025 (10mpk) vs. KDS12025 (1mpk), p = 0.89; KDS12025 (10mpk) vs. KDS12025 (0.1mpk), p =0.38; KDS12025 (1mpk) vs. KDS12025 (0.1mpk), p > 0.99 Dark: CTRL (20.68±0.4930, n=4), KDS12025 (10mpk) (20.73±0.9735, n=4), KDS12025 (1mpk) (21.29±0.7258, n=4), KDS12025 (0.1mpk) (22.37±1.215, n=4) CTRL vs. KDS12025 (10mpk), p > 0.99; CTRL vs. KDS12025 (1mpk), p > 0.99; CTRL vs. KDS12025 (0.1mpk), p = 0.98; KDS12025 (10mpk) vs. KDS12025 (1mpk), p > 0.99; KDS12025 (10mpk) vs. KDS12025 (0.1mpk), p = 0.99; KDS12025 (1mpk) vs. KDS12025 (0.1mpk), p > 0.99 Light: CTRL (18.81±0.6740, n=4), KDS12025 (10mpk) (18.91±1.057, n=4), KDS12025 (1mpk) (19.95±0.3562, n=4), KDS12025 (0.1mpk) (20.90±1.556, n=4) CTRL vs. KDS12025 (10mpk), p > 0.99; CTRL vs. KDS12025 (1mpk), p > 0.99; CTRL vs. KDS12025 (0.1mpk), p = 0.90; KDS12025 (10mpk) vs. KDS12025 (1mpk), p > 0.99; KDS12025 (10mpk) vs. KDS12025 (0.1mpk), p =0.93; KDS12025 (1mpk) vs. KDS12025 (0.1mpk), p > 0.99 |
| S8c | Two-way ANOVA with Sidak's multiple comparison test, F (6,24) = 0.2059, p = 0.97 Full day: CTRL (0.1391±0.0116, n=4), KDS12025 (10mpk) (0.1288±0.0108, n=4), KDS12025 (1mpk) (0.166±0.03508, n=4), KDS12025 (0.1mpk) (0.126±0.0123, n=4) CTRL vs. KDS12025 (10mpk), p > 0.99; CTRL vs. KDS12025 (1mpk), p > 0.99; CTRL vs. KDS12025 (0.1mpk), p > 0.99; KDS12025 (10mpk) vs. KDS12025 (1mpk), p = 0.93; KDS12025 (10mpk) vs. KDS12025 (0.1mpk), p > 0.99; KDS12025 (1mpk) vs. KDS12025 (0.1mpk), p = 0.96 Dark: CTRL (0.1680±0.0158, n=4), KDS12025 (10mpk) (0.1572±0.01881, n=4), KDS12025 (1mpk) (0.1761±0.0164, n=4), KDS12025 (0.1mpk) (0.1543±0.018, n=4) CTRL vs. KDS12025 (10mpk), p > 0.99; CTRL vs. KDS12025 (1mpk), p > 0.99; CTRL vs. KDS12025 (0.1mpk), p > 0.99; KDS12025 (10mpk) vs. KDS12025 (1mpk), p > 0.99; KDS12025 (10mpk) vs. KDS12025 (0.1mpk), p > 0.99; KDS12025 (1mpk) vs. KDS12025 (0.1mpk), p > 0.99 Light: CTRL (0.1056±0.018, n=4), KDS12025 (10mpk) (0.09464±0.0095, n=4), KDS12025 (1mpk) (0.1032±0.009, n=4), KDS12025 (0.1mpk) (0.07281±0.003, n=4) CTRL vs. KDS12025 (10mpk), p > 0.99; CTRL vs. KDS12025 (1mpk), p > 0.99; CTRL vs. KDS12025 (0.1mpk), p = 0.98; KDS12025 (10mpk) vs. KDS12025 (1mpk), p > 0.99; KDS12025 (10mpk) vs. KDS12025 (0.1mpk), p > 0.99; KDS12025 (1mpk) vs. KDS12025 (0.1mpk), p > 0.99 |
| S8d | Two-way ANOVA with Sidak's multiple comparison test, F (6,16) = 0.3547, p = 0.90 Full day: CTRL (0.07±0.0116, n=4), KDS12025 (10mpk) (0.07760±0.01206, n=4), KDS12025 (1mpk) (0.0902±0.0248, n=4), KDS12025 (0.1mpk) (0.108±0.005, n=4) CTRL vs. KDS12025 (10mpk), p > 0.99; CTRL vs. KDS12025 (1mpk), p > 0.99; CTRL vs. KDS12025 (0.1mpk), p > 0.99; KDS12025 (10mpk) vs. KDS12025 (1mpk), p > 0.99; KDS12025 (10mpk) vs. KDS12025 (0.1mpk), p > 0.99; KDS12025 (1mpk) vs. KDS12025 (0.1mpk), p > 0.99 Dark: CTRL (0.0862±0.0258, n=4), KDS12025 (10mpk) (0.1041±0.0394, n=4), KDS12025 (1mpk) (0.1220±0.0208, n=4), KDS12025 (0.1mpk) (0.1272±0.009, n=4) CTRL vs. KDS12025 (10mpk), p > 0.99; CTRL vs. KDS12025 (1mpk), p > 0.99; CTRL vs. KDS12025 (0.1mpk), p = 0.99; KDS12025 (10mpk) vs. KDS12025 (1mpk), p > 0.99; KDS12025 (10mpk) vs. KDS12025 (0.1mpk), p > 0.99; KDS12025 (1mpk) vs. KDS12025 (0.1mpk), p > 0.99 Light: CTRL (0.0599±0.0177, n=4), KDS12025 (10mpk) (0.05856±0.0075, n=4), KDS12025 (1mpk) (0.0790±0.0149, n=4), KDS12025 (0.1mpk) (0.0693±0.0004, n=4 CTRL vs. KDS12025 (10mpk), p > 0.99; CTRL vs. KDS12025 (1mpk), p > 0.99; CTRL vs. KDS12025 (0.1mpk), p > 0.99; KDS12025 (10mpk) vs. KDS12025 (1mpk), p > 0.99; KDS12025 (10mpk) vs. KDS12025 (0.1mpk), p > 0.99; KDS12025 (1mpk) vs. KDS12025 (0.1mpk), p > 0.99 |
| S8f | Sal. (1198±24.6, n=3), 0.1 mpk (1169±10.7, n=3), 1 mpk (1205±10.4, n=3), 10 mpk (1190±7.4, n=3) One-way ANOVA with Tukey's multiple comparisons test, F(3,8) = 1.12, p = 0.40 Sal. vs. 0.1 mpk, p = 0.54; Sal. vs. 1 mpk, p = 0.99; Sal. vs. 10 mpk, p = 0.98; 0.1 mpk vs. 1 mpk, p = 0.37; 0.1 mpk vs. 10 mpk, p = 0.75; 1 mpk vs. 10 mpk, p = 0.88 |
| S8g | Sal. (206±3.9, n=4), 0.1 mpk (193±3.8, n=4), 1 mpk (200±2.3, n=4), 10 mpk (203±3.9, n=4) One-way ANOVA with Tukey's multiple comparisons test, F(3,12) = 2.53, p = 0.11 Sal. vs. 0.1 mpk, p = 0.09; Sal. vs. 1 mpk, p = 0.62; Sal. vs. 10 mpk, p = 0.94; 0.1 mpk vs. 1 mpk, p = 0.53; 0.1 mpk vs. 10 mpk, p = 0.23; 1 mpk vs. 10 mpk, p = 0.91 |
| S9b | Blank (-1.000e-008±0.07, n=8), H2O2 (1.0±0.02, n=6), NO (0.025±0.026, n=6), ONOO+Cat. (-0.12±0.05, n=6) One-way ANOVA with Tukey's multiple comparisons test, F(3,20) = 127.4, p < 0.001 Blank vs. H2O2, p < 0.001; Blank vs. NO, p = 0.98; Blank vs. ONOO+Cat., p = 0.27, H2O2 vs. NO, p < 0.001, H2O2 vs. ONOO+Cat., p < 0.001, NO vs. ONOO+Cat., p = 0.15 |
| S10b | Two-way ANOVA with Sidak's multiple comparison test, F (4,58) = 2.950, p = 0.0275 Acquisition: WT (45.24±14.0, n=9), APP/PS1 (56.45±20.33, n=6), APP/PS1+KDS12008 (44.42±13.85, n=6),  APP/PS1+KDS12017 (71.53±23.24, n=4), APP/PS1+KDS12025 (29.03±6.431, n=9) WT vs. APP/PS1, p > 0.9999; WT vs APP/PS1+KDS12008, p > 0.9999; WT vs APP/PS1+KDS12017, p > 0.9999; WT vs APP/PS1+KDS12025, p > 0.9999; APP/PS1 vs APP/PS1+KDS12008, p > 0.9999; APP/PS1 vs APP/PS1+KDS12017, p > 0.9999; APP/PS1 vs APP/PS1+KDS12025, p > 0.9999; APP/PS1+KDS12008 vs APP/PS1+KDS12017, p > 0.9999; APP/PS1+KDS12008 vs APP/PS1+KDS12025, p > 0.9999; APP/PS1+KDS12017 vs APP/PS1+KDS12025, p > 0.9999 Retrieval: WT (414.1±63.65, n=9), APP/PS1 (59.87±22.89, n=6), APP/PS1+KDS12008 (233.6±102.3, n=6),  APP/PS1+KDS12017 (305.6±137.5, n=4), APP/PS1+KDS12025 (291.0±68.80, n=9) WT vs. APP/PS1, p = 0.0002; WT vs APP/PS1+KDS12008, p = 0.2005; WT vs APP/PS1+KDS12017, p = 0.9166; WT vs APP/PS1+KDS12025, p = 0.5572; APP/PS1 vs APP/PS1+KDS12008, p = 0.3581; APP/PS1 vs APP/PS1+KDS12017, p = 0.1085; APP/PS1 vs APP/PS1+KDS12025, p = 0.0379; APP/PS1+KDS12008 vs APP/PS1+KDS12017, p = 0.9973; APP/PS1+KDS12008 vs APP/PS1+KDS12025, p = 0.9978; APP/PS1+KDS12017 vs APP/PS1+KDS12025, p > 0.9999 |
| S10d | WT (1557±63.18, n=19), APP/PS1 (2513±115.0, n=41), APP/PS1+KDS12008 (2063±93.76, n=32),  APP/PS1+KDS12017 (1711±145.9, n=23), APP/PS1+KDS12025 (1190±87.29, n=19)  One-way ANOVA with Tukey's multiple comparisons test, F(4,129) = 20.43, p < 0.001 WT vs. APP/PS1, p < 0.001; WT vs APP/PS1+KDS12008, p = 0.03; WT vs APP/PS1+KDS12017, p = 0.92; WT vs APP/PS1+KDS12025, p = 0.32; APP/PS1 vs APP/PS1+KDS12008, p = 0.01; APP/PS1 vs APP/PS1+KDS12017, p <0.001; APP/PS1 vs APP/PS1+KDS12025, p < 0.001; APP/PS1+KDS12008 vs APP/PS1+KDS12017, p = 0.19; APP/PS1+KDS12008 vs APP/PS1+KDS12025, p < 0.001; APP/PS1+KDS12017 vs APP/PS1+KDS12025, p = 0.04 |
| S10e | WT (1213±22.64, n=20), APP/PS1 (1512±43.96, n=43), APP/PS1+KDS12008 (1562±64.27, n=32),  APP/PS1+KDS12017 (1556±53.65, n=21), APP/PS1+KDS12025 (1343±38.44, n=36)  One-way ANOVA with Tukey's multiple comparisons test, F(4,147) = 7.937, p < 0.001 WT vs. APP/PS1, p < 0.001; WT vs APP/PS1+KDS12008, p < 0.001; WT vs APP/PS1+KDS12017, p < 0.001; WT vs APP/PS1+KDS12025, p = 0.43; APP/PS1 vs APP/PS1+KDS12008, p = 0.94; APP/PS1 vs APP/PS1+KDS12017, p =0.97; APP/PS1 vs APP/PS1+KDS12025, p = 0.05; APP/PS1+KDS12008 vs APP/PS1+KDS12017, p > 0.99; APP/PS1+KDS12008 vs APP/PS1+KDS12025, p = 0.01; APP/PS1+KDS12017 vs APP/PS1+KDS12025, p = 0.04 |
| S10g | Two-way ANOVA with Tukey's multiple comparison test, F (3,66) = 13.58, p < 0.001 Acquisition: WT+Saline (40.67±6.90, n=11), APP/PS1+Saline (22.17±3.617, n=7),  APP/PS1+KDS12025 (3mpk) (36.47±13.39, n=10), APP/PS1+KDS12025 (10mpk) (66.00±15.30, n=9) WT+Saline vs. APP/PS1+Saline, p = 0.99; WT+Saline vs. APP/PS1+KDS12025 (3mpk), p > 0.99; WT+Saline vs. APP/PS1+KDS12025 (10mpk), p = 0.96; APP/PS1+Saline vs. APP/PS1+KDS12025 (3mpk), p > 0.99; APP/PS1+Saline vs. APP/PS1+KDS12025 (10mpk), p = 0.86; APP/PS1+KDS12025 (3mpk) vs. APP/PS1+KDS12025 (10mpk), p = 0.94 Retrieval: WT+Saline (556.0±31.47, n=11), APP/PS1+Saline (83.69±15.91, n=7),  APP/PS1+KDS12025 (3mpk) (341.7±71.16, n=10), APP/PS1+KDS12025 (10mpk) (508.8±49.93, n=9) WT+Saline vs. APP/PS1+Saline, p < 0.001; WT+Saline vs. APP/PS1+KDS12025 (3mpk), p < 0.001; WT+Saline vs. APP/PS1+KDS12025 (10mpk), p = 0.77; APP/PS1+Saline vs. APP/PS1+KDS12025 (3mpk), p < 0.001; APP/PS1+Saline vs. APP/PS1+KDS12025 (10mpk), p < 0.001; APP/PS1+KDS12025 (3mpk) vs. APP/PS1+KDS12025 (10mpk), p = 0.008 |
| S10i | WT (54.04±4.850, n=6), APP/PS1 (84.53±6.827, n=11),  APP/PS1+KDS (3mpk) (59.33±6.459, n=10), APP/PS1+KDS (10mpk) (56.71±4.775, n=9) One-way ANOVA with Tukey's multiple comparisons test, F(3,39) = 8.841, p < 0.001 WT vs. APP/PS1, p = 0.02; WT vs. APP/PS1+KDS (3mpk), p = 0.95; WT vs. APP/PS1+KDS (10mpk), p > 0.99; APP/PS1 vs. APP/PS1+KDS (3mpk), p = 0.02; APP/PS1 vs. APP/PS1+KDS (10mpk), p = 0.01; APP/PS1+KDS (3mpk) vs. APP/PS1+KDS (10mpk), p = 0.99 |
| S10k | WT (4.001±0.4549, n=15), APP/PS1 (11.24±1.731, n=16), APP/PS1+KDS (3mpk) (4.160±0.8926, n=7). APP/PS1+KDS (10mpk) (3.113±0.7279, n=5) One-way ANOVA with Tukey's multiple comparisons test, F(3,39) = 8.841, p < 0.001 WT vs. APP/PS1, p < 0.001; WT vs. APP/PS1+KDS (3mpk), p > 0.99; WT vs. APP/PS1+KDS (10mpk), p = 0.98; APP/PS1 vs. APP/PS1+KDS (3mpk), p = 0.007; APP/PS1 vs. APP/PS1+KDS (10mpk), p = 0.006; APP/PS1+KDS (3mpk) vs. APP/PS1+KDS (10mpk), p = 0.98 |
| S10l | WT (34.57±1.908, n=10), APP/PS1 (41.39±4.835, n=16),  APP/PS1+KDS (3mpk) (36.09±4.601, n=7), APP/PS1+KDS (10mpk) (26.02±1.734, n=5) One-way ANOVA with Tukey's multiple comparisons test, F(3,34) = 1.596, p = 0.21 WT vs. APP/PS1, p = 0.64; WT vs. APP/PS1+KDS (3mpk), p > 0.99; WT vs. APP/PS1+KDS (10mpk), p = 0.60; APP/PS1 vs. APP/PS1+KDS (3mpk), p = 0.84; APP/PS1 vs. APP/PS1+KDS (10mpk), p = 0.17; APP/PS1+KDS (3mpk) vs. APP/PS1+KDS (10mpk), p = 0.63 |
| S10m | WT (1.983±0.4121, n=11), APP/PS1 (2.428±0.3485, n=15),  APP/PS1+KDS (3mpk) (1.355±0.2694, n=7), APP/PS1+KDS (10mpk) (1.177±0.5985, n=5) One-way ANOVA with Tukey's multiple comparisons test, F(3,34) = 1.596, p = 0.21 WT vs. APP/PS1, p = 0.81; WT vs. APP/PS1+KDS (3mpk), p = 0.73; WT vs. APP/PS1+KDS (10mpk), p = 0.64; APP/PS1 vs. APP/PS1+KDS (3mpk), p = 0.27; APP/PS1 vs. APP/PS1+KDS (10mpk), p = 0.24; APP/PS1+KDS (3mpk) vs. APP/PS1+KDS (10mpk), p > 0.99 |
| S11c | WT (41.73±1.1951, n=19), APP (50.90±1.074, n=41), APP+KDS12025 (ad libitum) (44.45±1.015, n=28) One-way ANOVA with Tukey's multiple comparisons test, F(2,85) = 14.24, p < 0.001 WT vs. APP, p < 0.001; WT vs. APP+KDS12025 (ad libitum), p = 0.38; APP vs. APP+KDS12025 (ad libitum), p < 0.001 |
| S11d | WT (1903±72.86, n=32), APP (2435±66.11, n=58), APP+KDS12025 (ad libitum) (1951±48.61, n=57) One-way ANOVA with Tukey's multiple comparisons test, F(2,144) = 23.42, p < 0.001 WT vs. APP, p < 0.001; WT vs. APP+KDS12025 (ad libitum), p = 0.88; APP vs. APP+KDS12025 (ad libitum), p < 0.001 |
| S11e | WT (0.2256±0.02750, n=3), GiD (-0.02438±0.07989, n=3), GiD+KDS12025 (0.1mpk) (0.2255±0.02347, n=4) One-way ANOVA with Tukey's multiple comparisons test, F(2,7) = 9.285, p = 0.01 WT vs. GiD, p = 0.02; WT vs. GiD+KDS12025 (0.1mpk), p > 0.99; GiD vs. GiD+KDS12025 (0.1mpk), p = 0.01 |
| S12c | Control (89.59±3.31, n=35), A53T ipsi (186.7±1.985, n=57), A53T ipsi+KDS12025 (106.7±3.202, n=32), A53T contra (79.40±5.141, n=31),  A53T contra+KDS12025 (82.69±4.999, n=28) One-way ANOVA with Tukey's multiple comparisons test, F(4,178) = 214.4, p < 0.001 Control vs. A53T ipsi, p < 0.001; Control vs. A53T ipsi+KDS12025, p = 0.009; Control vs. A53T contra, p = 0.29; Control vs. A53T contra+KDS12025, p = 0.70; A53T ipsi vs. A53T ipsi+KDS12025, p < 0.001; A53T ipsi vs. A53T contra, p < 0.001; A53T ipsi vs. A53T contra+KDS12025, p < 0.0001; A53T ipsi+KDS12025 vs. A53T contra, p < 0.001; A53T ipsi+KDS12025 vs. A53T contra+KDS12025, p < 0.001; A53T contra vs. A53T contra+KDS12025, p = 0.98 |
| S13b | Unpaired t-test, one-tailed Normal (1.0±0.3789, n=8) vs. AD (0.3084±0.02120, n=8), p = 0.04, t = 1.822, df = 14 |
| S13c | B1 (0.23±0.10, n=6), B2 (0.072±0.019, n=4), B3 (0.056±0.014, n=13) One-way ANOVA with Tukey's multiple comparisons test, F(2,20) = 3.836, p = 0.04 B1 vs. B2, p = 0.17; B1 vs. B3, p = 0.03; B2 vs. B3, p = 0.98 |
| S13d | Unpaired t-test, two-tailed WT (0.5166±0.08026, n=4) vs. APP/PS1 (0.2328±0.03508, n=4), p = 0.02, t = 3.241, df = 6 |
| S15b | Control (2194±76.5, n=25), SOD1 (1209±54.8, n=31), SOD1+KDS (2557±108.6, n=37) One-way ANOVA with Tukey's multiple comparisons test, F(2,90) = 65.01, p < 0.001 Control vs. SOD1, p < 0.001; Control vs. SOD1+KDS, p = 0.02; SOD1 vs. SOD1+KDS, p < 0.001 |
| S15d | Control (1961±96.1, n=22), SOD1 (1183±89.9, n=19), SOD1+KDS (2326±85.3, n=17) One-way ANOVA with Tukey's multiple comparisons test, F(2,55) = 38.14, p < 0.001 Control vs. SOD1, p < 0.001; Control vs. SOD1+KDS, p = 0.02; SOD1 vs. SOD1+KDS, p < 0.001 |
| S16e | WT (117.1±19.54, n=21), APP (203.7±22.90, n=18),  APP+shHbβ+KDS12025 (234.3±16.93, n=26), APP+ScRNA+KDS12025 (142.4±8.715, n=35) One-way ANOVA with Tukey's multiple comparisons test, F(3,99) = 61.21, p < 0.001 WT vs. APP, p = 0.005; WT vs. APP+shHbβ+KDS12025, p < 0.001; WT vs. APP+ScRNA+KDS12025, p = 0.65; APP vs. APP+shHbβ+KDS12025, p = 0.59; APP vs. APP+ScRNA+KDS12025, p = 0.04; APP+shHbβ+KDS12025 vs. APP+ScRNA+KDS12025, p < 0.001 |
| S16f | WT (72.00±7.810, n=10), APP (113.1.±6.305, n=14),  APP+shHbβ+KDS12025 (100.4±6.075, n=28), APP+ScRNA+KDS12025 (54.48±2.319, n=23) One-way ANOVA with Tukey's multiple comparisons test, F(3,61) = 28.97, p < 0.001 WT vs. APP, p < 0.001; WT vs. APP+shHbβ+KDS12025, p = 0.006; WT vs. APP+ScRNA+KDS12025, p = 0.13; APP vs. APP+shHbβ+KDS12025, p = 0.33; APP vs. APP+ScRNA+KDS12025, p < 0.001; APP+shHbβ+KDS12025 vs. APP+ScRNA+KDS12025, p < 0.001 |
| S16g | Two-way ANOVA with Tukey's multiple comparison test, F (3,310) = 8.024, p < 0.001 WT (4.037±0.7779, n=9), APP (4.614±0.8108, n=12),  APP+shHbβ+KDS12025 (5.314±0.9626, n=11), APP+ScRNA+KDS12025 (4.984±0.7027, n=8) WT vs. APP, p = 0.01; WT vs. APP+shHbβ+KDS12025, p < 0.001; WT vs. APP+ScRNA+KDS12025, p > 0.99; APP vs. APP+shHbβ+KDS12025, p = 0.64; APP vs. APP+ScRNA+KDS12025, p = 0.03; APP+shHbβ+KDS12025 vs. APP+ScRNA+KDS12025, p = 0.001 |
| S17b | WT (30.0±4.1, n=12), APP+Sc-shRNA (431±4.2, n=18), APP+shHbβ::GFAP+KDS12025 (57.9±3.7, n=18), APP+shHbβ::CaMKII+KDS12025 (42.1±3.9, n=14) One-way ANOVA with Tukey's multiple comparisons test, F(3,58) = 7.67, p < 0.001 WT vs. APP+Sc-shRNA, p = 0.09; WT vs. APP+shHbβ::GFAP+KDS12025, p < 0.001; WT vs. APP+shHbβ::CaMKII+KDS12025, p = 0.11; APP+Sc-shRNA vs. APP+shHbβ::GFAP+KDS12025, p = 0.03; APP+Sc-shRNA vs. APP+shHbβ::CaMKII+KDS12025, p = 0.87; APP+shHbβ::GFAP+KDS12025 vs. APP+shHbβ::CaMKII+KDS12025, p = 0.03 |
| S17c | WT (67.7±5.8, n=12), APP+Sc-shRNA (68.1±4.8, n=18), APP+shHbβ::GFAP+KDS12025 (109.8±5.4, n=18), APP+shHbβ::CaMKII+KDS12025 (80.3±6.8, n=14) One-way ANOVA with Tukey's multiple comparisons test, F(3,58) = 12.66, p < 0.001 WT vs. APP+Sc-shRNA, p = 0.96; WT vs. APP+shHbβ::GFAP+KDS12025, p < 0.001; WT vs. APP+shHbβ::CaMKII+KDS12025, p = 0.35; APP+Sc-shRNA vs. APP+shHbβ::GFAP+KDS12025, p < 0.001; APP+Sc-shRNA vs. APP+shHbβ::CaMKII+KDS12025, p = 0.35; APP+shHbβ::GFAP+KDS12025 vs. APP+shHbβ::CaMKII+KDS12025, p = 0.002 |
| S17d | Two-way ANOVA with Dunnett's multiple comparison test, F (3, 299) = 6.74, p < 0.001  WT vs. APP+shHbβ::GFAP+KDS12025, p = 0.003 in *, APP+shHbβ::GFAP+KDS12025 vs. APP+shHbβ::CaMKII+KDS12025, p = 0.001 in ^#^ |
| S17f | Unaired t-test, two tailed Ctrl (1.191±0.3225, n=6), D1 (4.588±0.9107, n=6), p = 0.006; t = 3.517, df = 10 |
| S17g | Ctrl (1.0±0.1, n=3), H_2_O_2_ 200 μM, 1hr (1.853±0.2, n=3), H_2_O_2_ 200 μM, 3hr (2.544±0.2, n=3) One-way ANOVA with Tukey's multiple comparisons test, F(2,6) = 19.94, p = 0.002 Ctrl vs. H_2_O_2_ 200 μM, 1hr, p = 0.03; Ctrl vs. H_2_O_2_ 200 μM, 3hr, p = 0.002; H_2_O_2_ 200 μM, 1hr vs. H_2_O_2_ 200 μM, 3hr, p = 0.07 |
| S19b | 18 month (40.7±0.7, n=95), 30 month (71.5±1.04, n=66), 30 month+KDS (51.1±0.8, n=83) One-way ANOVA with Tukey's multiple comparisons test, F(2,241) = 354.7, p < 0.001 18 month vs. 30 month, p < 0.001; 18 month vs. 30 month+KDS, p < 0.001; 30 month vs. 30 month+KDS, p < 0.001 |
| S19c | 18 month (50.7±0.7, n=95), 30 month (67.03±1.7, n=66), 30 month+KDS (69.2±1.3, n=83) One-way ANOVA with Tukey's multiple comparisons test, F(2,198) = 84.9, p < 0.001 18 month vs. 30 month, p < 0.001; 18 month vs. 30 month+KDS, p < 0.001; 30 month vs. 30 month+KDS, p = 0.47 |
| S19d | Unaired t-test, two tailed 30 month (23.75±5.12, n=4), 30 month+KDS (40.2±2.67, n=5), p = 0.02 t = 3.03, df = 7 |
| S20c | Normal (0.0±0, n=6), CIA+Vehicle (11.08±1.274, n=6), CIA+KDS12025 0.1 MPK (9.5±1.133, n=6),  CIA+KDS12025 1 MPK (6.583±0.7002, n=6) One-way ANOVA with Tukey's multiple comparisons test, F(3,20) = 28.23, p < 0.001 Normal vs. CIA+Vehicle, p < 0.001; Normal vs. CIA+KDS12025 0.1 MPK, p < 0.001; Normal vs. CIA+KDS12025 1 MPK, p < 0.001; CIA+Vehicle vs. CIA+KDS12025 0.1 MPK, p = 0.62; CIA+Vehicle vs. CIA+KDS12025 1 MPK, p = 0.01; CIA+KDS12025 0.1 MPK vs. CIA+KDS12025 1 MPK, p = 0.15 |
| S20e | Normal (0.0±0, n=6), CIA+Vehicle (70.83±11.93, n=6), CIA+KDS12025 0.1 MPK (54.17±15.02, n=6),  CIA+KDS12025 1 MPK (25.0±9.129, n=6) One-way ANOVA with Tukey's multiple comparisons test, F(3,20) = 8.718, p < 0.001 Normal vs. CIA+Vehicle, p < 0.001; Normal vs. CIA+KDS12025 0.1 MPK, p = 0.009; Normal vs. CIA+KDS12025 1 MPK, p = 0.37; CIA+Vehicle vs. CIA+KDS12025 0.1 MPK, p = 0.69; CIA+Vehicle vs. CIA+KDS12025 1 MPK, p = 0.03; CIA+KDS12025 0.1 MPK vs. CIA+KDS12025 1 MPK, p = 0.24 |
| S20f | Normal (0.09±0.0, n=6), CIA+Vehicle (0.1533±0.01085, n=6), CIA+KDS12025 0.1 MPK (0.1383±0.0074, n=6),  CIA+KDS12025 1 MPK (0.1183±0.0047, n=6) One-way ANOVA with Tukey's multiple comparisons test, F(3,20) = 15.25, p < 0.001 Normal vs. CIA+Vehicle, p < 0.001; Normal vs. CIA+KDS12025 0.1 MPK, p < 0.001; Normal vs. CIA+KDS12025 1 MPK, p = 0.04; CIA+Vehicle vs. CIA+KDS12025 0.1 MPK, p = 0.45; CIA+Vehicle vs. CIA+KDS12025 1 MPK, p = 0.01; CIA+KDS12025 0.1 MPK vs. CIA+KDS12025 1 MPK, p = 0.22 |
| S20h | Normal (0.0±0, n=5), CIA+Vehicle (2.964±0.024, n=5), CIA+KDS12025 0.1 MPK (1.338±0.2985, n=5),  CIA+KDS12025 1 MPK (0.7620±0.3263, n=5) One-way ANOVA with Tukey's multiple comparisons test, F(3,16) = 32.25, p < 0.001 Normal vs. CIA+Vehicle, p < 0.001; Normal vs. CIA+KDS12025 0.1 MPK, p = 0.003; Normal vs. CIA+KDS12025 1 MPK, p = 0.11; CIA+Vehicle vs. CIA+KDS12025 0.1 MPK, p < 0.001; CIA+Vehicle vs. CIA+KDS12025 1 MPK, p < 0.001; CIA+KDS12025 0.1 MPK vs. CIA+KDS12025 1 MPK, p = 0.29 |
| S20i | Normal (0.0±0, n=5), CIA+Vehicle (3.0±0, n=5), CIA+KDS12025 0.1 MPK (1.5±0.2949, n=5),  CIA+KDS12025 1 MPK (0.966±0.2183, n=5) One-way ANOVA with Tukey's multiple comparisons test, F(3,16) = 46.69, p < 0.001 Normal vs. CIA+Vehicle, p < 0.001; Normal vs. CIA+KDS12025 0.1 MPK, p < 0.001; Normal vs. CIA+KDS12025 1 MPK, p = 0.009; CIA+Vehicle vs. CIA+KDS12025 0.1 MPK, p < 0.001; CIA+Vehicle vs. CIA+KDS12025 1 MPK, p < 0.001; CIA+KDS12025 0.1 MPK vs. CIA+KDS12025 1 MPK, p = 0.21 |
| S20j | Normal (0.0±0, n=5), CIA+Vehicle (2.664±0.1938, n=5), CIA+KDS12025 0.1 MPK (0.9±0.3929, n=5),  CIA+KDS12025 1 MPK (0.338±0.2928, n=5) One-way ANOVA with Tukey's multiple comparisons test, F(3,16) = 20.24, p < 0.001 Normal vs. CIA+Vehicle, p < 0.001; Normal vs. CIA+KDS12025 0.1 MPK, p = 0.11; Normal vs. CIA+KDS12025 1 MPK, p = 0.80; CIA+Vehicle vs. CIA+KDS12025 0.1 MPK, p = 0.001; CIA+Vehicle vs. CIA+KDS12025 1 MPK, p < 0.001; CIA+KDS12025 0.1 MPK vs. CIA+KDS12025 1 MPK, p = 0.46 |
| S20k | Normal (0.0±0, n=5), CIA+Vehicle (2.64±0.1974, n=5), CIA+KDS12025 0.1 MPK (0.852±0.3905, n=5),  CIA+KDS12025 1 MPK (0.338±0.2928, n=5) One-way ANOVA with Tukey's multiple comparisons test, F(3,16) = 19.93, p < 0.001 Normal vs. CIA+Vehicle, p < 0.001; Normal vs. CIA+KDS12025 0.1 MPK, p = 0.14; Normal vs. CIA+KDS12025 1 MPK, p = 0.80; CIA+Vehicle vs. CIA+KDS12025 0.1 MPK, p = 0.001; CIA+Vehicle vs. CIA+KDS12025 1 MPK, p < 0.001; CIA+KDS12025 0.1 MPK vs. CIA+KDS12025 1 MPK, p = 0.53 |
| S20m | Normal (0.0±0, n=5), CIA+Vehicle (3.0±0, n=5), CIA+KDS12025 0.1 MPK (1.504±0.3303, n=5),  CIA+KDS12025 1 MPK (0.988±0.2547, n=5) One-way ANOVA with Tukey's multiple comparisons test, F(3,16) = 19.93, p < 0.001 Normal vs. CIA+Vehicle, p < 0.001; Normal vs. CIA+KDS12025 0.1 MPK, p = 0.14; Normal vs. CIA+KDS12025 1 MPK, p = 0.80; CIA+Vehicle vs. CIA+KDS12025 0.1 MPK, p = 0.001; CIA+Vehicle vs. CIA+KDS12025 1 MPK, p < 0.001; CIA+KDS12025 0.1 MPK vs. CIA+KDS12025 1 MPK, p = 0.53 |
| S20n | Normal (0.0±0, n=5), CIA+Vehicle (2.976±0.024, n=5), CIA+KDS12025 0.1 MPK (1.352±0.3083, n=5),  CIA+KDS12025 1 MPK (0.95±0.2009, n=5) One-way ANOVA with Tukey's multiple comparisons test, F(3,16) = 45.32, p < 0.001 Normal vs. CIA+Vehicle, p < 0.001; Normal vs. CIA+KDS12025 0.1 MPK, p < 0.001; Normal vs. CIA+KDS12025 1 MPK, p = 0.01; CIA+Vehicle vs. CIA+KDS12025 0.1 MPK, p < 0.001; CIA+Vehicle vs. CIA+KDS12025 1 MPK, p < 0.001; CIA+KDS12025 0.1 MPK vs. CIA+KDS12025 1 MPK, p = 0.44 |
| S20o | Normal (0.0±0, n=5), CIA+Vehicle (2.188±0.1666, n=5), CIA+KDS12025 0.1 MPK (0.85±0.2018, n=5),  CIA+KDS12025 1 MPK (0.652±0.1825, n=5) One-way ANOVA with Tukey's multiple comparisons test, F(3,16) = 30.86, p < 0.001 Normal vs. CIA+Vehicle, p < 0.001; Normal vs. CIA+KDS12025 0.1 MPK, p = 0.01; Normal vs. CIA+KDS12025 1 MPK, p = 0.06; CIA+Vehicle vs. CIA+KDS12025 0.1 MPK, p < 0.001; CIA+Vehicle vs. CIA+KDS12025 1 MPK, p < 0.001; CIA+KDS12025 0.1 MPK vs. CIA+KDS12025 1 MPK, p = 0.83 |
| S20p | Normal (105.2±0.7, n=5), CIA+Vehicle (93.1±0.8, n=5), CIA+KDS12025 0.1 MPK (93±0.5, n=5),  CIA+KDS12025 1 MPK (100.7±0.4, n=5) One-way ANOVA with Tukey's multiple comparisons test, F(3,16) = 100.86, p < 0.001 Normal vs. CIA+Vehicle, p < 0.001; Normal vs. CIA+KDS12025 0.1 MPK, p < 0.01; Normal vs. CIA+KDS12025 1 MPK, p = 0.20; CIA+Vehicle vs. CIA+KDS12025 0.1 MPK, p > 0.99; CIA+Vehicle vs. CIA+KDS12025 1 MPK, p < 0.001; CIA+KDS12025 0.1 MPK vs. CIA+KDS12025 1 MPK, p < 0.001 |
| S20q | Control (0.2808±0.02283, n=5), LPS-2hrs (0.5301±0.04939, n=7), LPS-6hrs (1.204±0.05891, n=5),  LPS-24hrs (0.2503±0.03503, n=8) One-way ANOVA with Tukey's multiple comparisons test, F(3,21) = 89.41, p < 0.001 Control vs. LPS-2hrs, p = 0.004; Control vs. LPS-6hrs, p < 0.001; Control vs. LPS-24hrs, p = 0.96; LPS-2hrs vs. LPS-6hrs, p < 0.001; LPS-2hrs vs. LPS-24hrs, p < 0.001; LPS-6hrs vs. LPS-24hrs, p < 0.001 |
| S20r | Control (0.3325±0.02161, n=6), LPS-6hrs (1.295±0.05323, n=6), LPS-6hrs+KDS (0.7847±0.08078, n=6) One-way ANOVA with Tukey's multiple comparisons test, F(2,15) = 70.74, p < 0.001 Control vs. LPS-6hrs, p < 0.001; Control vs. LPS-6hrs+KDS, p < 0.001; LPS-6hrs vs. LPS-6hrs+KDS, p < 0.001 |
| S20t | RBC only (7.153±4.130, n=4), No KDS (100±2.5, n=5), KDS 0.01 (37.08±7.98, n=5), KSD 0.1 (20.61±3.586, n=5), KDS 1 (7.116±4.5, n=5) Onew-way ANOVA with Tukey’s multiple comparison test, F(4,19) = 61.06, p < 0.001 RBC only vs. No KDS, p < 0.001; RBC only vs. KDS 0.01, p = 0.005; RBC only vs. KDS 0.1, p = 0.38; RBC only vs. KDS 1, p > 0.99; No KDS vs. KDS 0.01, p < 0.001; No KDS vs. KDS 0.1, p < 0.001; No KDS vs. KDS 1, p < 0.001; KDS 0.01 vs. KDS 0.1, p = 16; |

**Supplementary Chemical Information**

**List of Contents**

1. Experimental procedure for synthesized small molecules
2. ^1^H and ^13^C NMR spectra of synthesized products
3. HPLC purities of final compounds
4. HRMS data of final compounds

**1. Experimental procedure for synthesized small molecules**

1. Preparation of *N*­methyl-4-nitroaniline (1)

A solution of fluoro-4-nitrobenzene (1.0 g, 7.1 mmol) and methylamine (3.41 g, 109.9 mmol) in 10 mL ethanol was heated at reflux for 24 h. The reaction was cooled and concentrated in vacuo. The resulting residue was diluted with ethyl acetate and washed with brine, dried (Na_2_SO_4_), and the solvent removed in vacuo to give 913.3 mg (85%) of the title compound as a yellow solid. R*_f_* = 0.70 (*n-*Hex 1: EtOAc 1); ^1^H NMR (400 MHz, DMSO-*d_6_*) *δ* 8.01 (d, 2H, *J* = 9.28 Hz), 7.31 (d, 1H, *J* = 4.28 Hz), 6.61 (d, 2H, *J* = 9.36 Hz), 2.80 (d, 2H, *J* = 5.00 Hz), 2.33 (s, 1H).

2. Preparation of *tert*-butyl methyl(4-nitrophenyl)carbamate (2)

To a solution of *N-*methyl-4-nitroaniline (800 mg, 5.26 mmol) in 10 mL THF were added di-*tert*-butyl dicarbonate (1.72 g, 7.89 mmol) and 4-dimethylaminopyridine (32.1 mg, 0.26 mmol). The reaction mixture was heated at reflux for 12 h. The reaction was cooled and concentrated in vacuo. The resulting residue was diluted with ethyl acetate and washed with brine, dried (Na_2_SO_4_), and the solvent was removed in vacuo to give 1.29 g (97%) of the title compound as a yellow oil. R*_f_* = 0.80 (*n-*Hex 3: EtOAc 1); ^1^H NMR (400 MHz, DMSO-*d_6_*) *δ* 8.20 (d, 2H, *J* = 9.20 Hz), 7.60 (d, 2H, *J* = 9.16 Hz), 3.29 (s, 3H), 1.45 (s, 9H).

3. Preparation of *tert*-butyl (4-aminophenyl)(methyl)carbamate (3)

To a solution of *tert*-butyl methyl(4-nitrophenyl)carbamate (6.67 g, 26.45 mmol) in 25 mL methanol, was added palladium on carbon (667 mg). The reaction mixture was stirred under hydrogen for 2 h at room temperature. The reaction was filtered through a pad of celite. The filtrate was concentrated in vacuo, to give 5.18 g (88%) of the title compound as a yellow solid. R*_f_* = 0.05 (*n-*Hex 1: EtOAc 1); ^1^H NMR (400 MHz, DMSO-*d_6_*) *δ* 6.86 (d, 2H, *J* = 8.52 Hz), 6.50 (d, 2H, *J* = 8.60 Hz), 5.01 (s, 2H), 3.06 (s, 3H), 1.35 (s, 9H).

4. Preparation of 1-(2-bromoethyl)-2-methoxybenzene (5a)

To a solution of 2-(2-methoxyphenyl)ethan-1-ol (5.0 g, 32.85 mmol) in anhydrous dichloromethane (75 mL) was added carbon tetrabromide (15.25 g, 45.99 mmol) and stirred at 0 °C under nitrogen atmosphere. Triphenylphospine (10.35 g, 39.42 mmol) was added potionwise and stirred under same condition for 1 h. The reaction mixture was concentrated in vacuo and the residue was purified by column chromatography on silica gel, eluting with *n-*hexane only and then a mixture of *n-*hexane and ethyl acetate (20:1) to afford 5.81 g (82%) of title compound as a colorless oil. R*_f_* = 0.20 (*n-*Hex); ^1^H NMR (400 MHz, DMSO-*d_6_*) *δ* 7.24 (t, 1H, *J* = 7.98 Hz), 6.99 (d, 1H, *J* = 8.16 Hz), 6.89 (t, 1H, *J* = 7.36 Hz), 3.79 (s, 3H), 3.64 (t, 2H, *J* = 7.48 Hz), 3.09 (t, 2H, *J* = 7.44 Hz).

5. Preparation of 1-(2-bromoethyl)-3-methoxybenzene (5b)

To a solution of 2-(3-methoxyphenyl)ethan-1-ol (1.0 g, 6.57 mmol) in anhydrous dichloromethane (20 mL) was added carbon tetrabromide (3.05 g, 9.20 mmol) and stirred at 0 °C under nitrogen atmosphere. Triphenylphospine (2.06 g, 7.88 mmol) was added portionwise and stirred under same condition for 1 h. The reaction mixture was concentrated in vacuo and the residue was purified by column chromatography on silica gel, eluting with *n-*hexane only and then a mixture of *n-*hexane and ethyl acetate (20:1) to afford 1.37 g (97%) of title compound as a colorless oil. R*_f_* = 0.20 (*n-*Hex); ^1^H NMR (400 MHz, DMSO-*d_6_*) *δ* 7.23 (t, 1H, *J* = 7.60 Hz), 6.88 (m, 3H), 3.75 (s, 3H), 3.74 (t, 2H, *J* = 7.2 Hz), 3.11 (t, 2H, *J* = 7.2 Hz).

6. General procedure for alkylated compounds (7a–7m) (Method A)

To a solution of aniline (3, 4a–4h) (1.0–3.0 equiv) in acetonitrile, were added potassium carbonate/cesium carbonate (1.0–1.2 equiv), potassium iodide (0.1 equiv), and phenethyl bromide (6a–6g) (1.0 equiv) in a sealed tube. The reaction mixture was heated at 110 °C for 36 h. The reaction mixture was cooled and diluted with ethyl acetate and washed with brine, dried with anhydrous Na_2_SO_4_, and concentrated in vacuo. Purification by column chromatography afforded the desired compound.

6.1. Preparation of *tert*-butyl methy­­­­­l ­­(4-((4-(trifluoromethyl)phenethyl)amino)phenyl) carbamate (7a)

Using method A, *tert*-butyl (4-aminophenyl)(methyl)carbamate (440 mg, 1.98 mmol), 1-(2-bromoethyl)-4-(trifluoromethyl)benzene (500 mg, 1.98 mmol), potassium carbonate (821 mg, 5.94 mmol), and potassium iodide (33 mg, 0.20 mmol) in acetonitrile (10 mL) to give 7a as an off white solid (319 mg, 41%); R*_f_* = 0.45 (*n-*Hex 3: EtOAC 1); ^1^H NMR (400 MHz, DMSO-*d_6_*) *δ* 7.68 (d, 2H, *J* = 8.00 Hz) 7.53 (d, 2H, *J* = 8.00 Hz), 6.96 (d, 2H, *J* = 8.80 Hz), 6.57 (d, 2H, *J* = 8.80 Hz), 5.70 (t, 1H, *J* = 5.60 Hz), 3.28 (t, 2H, *J* = 7.20 Hz), 3.08 (s, 3H), 2.94 (d, 2H, *J* = 7.20 Hz).

6.2. Preparation of *N*-(4-(trifluoromethyl)phenethyl)aniline (7b)

Using method A, aniline (552 mg, 5.93 mmol), 1-(2-bromoethyl)-4-(trifluoromethyl)benzene (500 mg, 1.98 mmol), potassium carbonate (273 mg, 1.98 mmol), and potassium iodide (33 mg, 0.20 mmol) in acetonitrile (10 mL) gave 7b as a white solid (50.4 mg, 8%); R*_f_* = 0.50 (*n-*Hex 9: EtOAC 1); mp 20–30 °C; ^1^H NMR (400 MHz, DMSO-*d_6_*) *δ* 7.65 (d, 2H, *J* = 8.12 Hz), 7.50 (d, 2H, *J* = 8.04 Hz), 7.07 (t, 2H, *J* = 8.16 Hz), 6.59 (d, 2H, *J* = 8.12 Hz), 6.53 (t, 1H, *J* = 7.24 Hz), 5.65 (t, 1H, *J* = 5.56 Hz), 3.30–3.25 (m, 2H), 2.92 (t, 2H, *J* = 7.28 Hz); ^13^C NMR (100 MHz, DMSO-*d_6_*) *δ* 149.1, 145.5, 130.0, 129.4, 127.7, 127.4, 127.1, 126.8, 126.3, 125.5 (q, *J*_C-F_ = 3.57 Hz), 116.2, 112.5, 44.5 (**C**H_2_), 35.1 (**C**H_2_); HPLC purity: 7.7 min, 99.2%; HRMS (M+H) (ESI^+^) 266.11575 [M + H]^+^ (calcd for C_15_H_14_F_3_NH^+^ 266.115109).

6.3. Preparation of 4-((4-(trifluoromethyl)phenethyl)amino)phenol (7c)

Using method A, 4-aminophenol (258.6 mg, 2.37 mmol), 1-(2-bromoethyl)-4-(trifluoromethyl)benzene (200 mg, 0.79 mmol), potassium carbonate (200 mg, 0.79 mmol), and potassium iodide (13 mg, 0.08 mmol) in acetonitrile (4 mL) gave 7c as a white solid (111 mg, 53%); R*_f_* = 0.30 (*n-*Hex 3: EtOAC 1); mp 110–120 °C; ^1^H NMR (400 MHz, DMSO-*d_6_*) *δ* 8.39 (s, 1H), 7.64 (d, 2H, *J* = 8.00 Hz), 7.49 (d, 2H, *J* = 8.00 Hz), 6.55 (d, 2H, *J* = 8.56 Hz), 6.45 (d, 2H, *J* = 8.64 Hz), 4.99 (s, 1H), 3.21–3.17 (m, 2H), 2.89 (t, 2H, *J* = 7.20 Hz); ^13^C NMR (100 MHz, DMSO-*d_6_*) *δ* 148.8, 145.7, 134.6, 130.0, 125.5 (q, *J*_C-F_ = 4.06 Hz), 116.2, 113.9, 103.1 45.7 (**C**H_2_), 35.3 (**C**H_2_); HPLC purity: 9.3 min, >99.9%; HRMS (M+H) (ESI^+^) 282.10986 [M + H]^+^ (calcd for C_15_H_14_F_3_NOH^+^ 282.110024).

6.4. Preparation of 4-methoxy-N-(4-(trifluoromethyl)phenethyl)aniline (7d)

Using method A, 4-methoxyaniline (292 mg, 2.37 mmol), 1-2-bromoethyl)-4-(trifluoromethyl)benzene (200 mg, 0.79 mmol), potassium carbonate (109 mg, 0.79 mmol), and potassium iodide (13 mg, 0.08 mmol) in acetonitrile (4 mL) gave 7d as a yellow oil (159 mg, 68%); R*_f_* = 0.31 (*n*-Hex 3: EtOAC 1); ^1^H NMR (400 MHz, DMSO-*d_6_*) *δ* 7.64 (d, 2H, *J* = 8.08 Hz), 7.49 (d, 2H, J = 8.00 Hz), 6.72 (d, 2H, *J* = 8.88 Hz), 6.55 (d, 2H, *J* = 8.92 Hz), 5.22 (t, 1H, *J* = 5.84 Hz), 3.64 (s, 3H), 3.22 (q, 2H, *J* = 6.88 Hz), 2.91 (t, 2H, *J* = 7.24 Hz); ^13^C NMR (100 MHz, DMSO-*d_6_*) 151.2, 145.6, 143.3, 130.0, 127.2 (q, *J*_C-F_ = 31.57 Hz), 124.9 (q, *J*_C-F_ = 270.32 Hz) 115.1, 113.6, 55.8 (**C**H_2_), 45.4 (**C**H_2_), 35.2 (**C**H_3_); HPLC purity: 12.2 min, >99.9%; HRMS (M+H) (ESI^+^) 296.12634 [M + H]^+^ (calcd for C_16_H_16_F_3_NOH^+^ 296.125674).

6.5. Preparation of 3-((4-(trifluoromethyl)phenethyl)amino)benzoic acid (7e)

Using method A, 3-aminobenzoic acid (813 mg, 5.93 mmol), 1-(2-bromoethyl)-4-(trifluoromethyl)benzene (500 mg, 1.98 mmol), potassium carbonate (273 mg, 1.98 mmol), and potassium iodide (33 mg, 0.20 mmol) in acetonitrile (10 mL) gave 7e as a white solid (49 mg, 8%); R*_f_* = 0.30 (*n-*Hex 3: EtOAC 1); mp 82–86 °C; ^1^H NMR (400 MHz, DMSO-*d_6_*) *δ* 12.65, (s, 1H), 7.66 (d, 2H, *J* = 8.08 Hz), 7.51 (d, 2H, *J* = 7.92 Hz), 7.21–7.18 (m, 2H), 7.14 (d, 1H, *J* = 7.40 Hz), 6.83 (d, 1H, *J* = 7.08 Hz), 5.98 (s, 1H), 3.32 (t, 2H, *J* = 7.04 Hz), 2.94 (t, 2H, *J* = 6.88 Hz); ^13^C NMR (100 MHz, DMSO-*d_6_*) *δ* 166.6 (**C**(O)), 149.5, 143.8, 130.7, 130.2, 129.5, 127.6 (q, *J*_C-F_ = 31.56 Hz), 125.7 (q, *J*_C-F_ = 3.71 Hz), 124.9 (q, *J*_C-F_ = 270.27 Hz), 118.9, 116.7, 114.5, 64.9 (**C**H_2_), 34.6 (**C**H_2_); HPLC purity: 12.6 min, 98.4%; HRMS (M+H) (ESI^+^) 310.10513 [M + H]^+^ (calcd for C_16_H_14_F_3_NO_2_H^+^ 310.104939).

6.6. Preparation of 4-((4-(trifluoromethyl)phenethyl)amino)benzoic acid (7f)

Using method a, 4-aminobenzoic acid (268.8 mg, 1.96 mmol), 1-(2-(bromoethyl)-4-(trifluoromethyl)benzene (496 mg, 1.96 mmol), cesium carbonate (765.7 mg, 2.35 mmol), and potassium iodide (32.5 mg, 0.20 mmol) in acetonitrile (5 mL) gave 7f as a white solid (43 mg, 5.6%); R*_f_* = 0.17 (*n-*Hex 1: EtOAC 1); mp 84–86 °C; ^1^H NMR *δ* 11.97 (m, 4H), 7.50 (d, 2H, *J* = 8.04 Hz), 6.60 (d, 2H, *J* = 8.80 Hz), 7.53 (t, 1H, *J* = 5.44 Hz), 3.36 (q, 2H, *J* = 7.00 Hz), 2.94 (t, 2H, *J* = 7.24); ^13^C NMR (100 MHz, DMSO-*d_6_*) *δ* 166.6 (**C**(O)), 149.5, 143.8, 130.7, 130.2, 129.5, 127.6 (q, *J*_C-F_ = 31.56 Hz), 125.7 (q, *J*_C-F_ = 3.71 Hz), 124.9 (q, *J*_C-F_ = 270.27 Hz), 118.9, 116.7, 114.5, 64.9 (**C**H_2_), 34.6 (**C**H_2_); HPLC purity: 10.3 min, >99.9%; HRMS (M+H) (ESI^+^) 310.10565 [M + H]^+^ (calcd for C_16_H_14_F_3_NO_2_H^+^ 310.104939).

6.7. Preparation of *N*^1^*,N*^1^-dimethyl-*N*^4^-(4-(trifluoromethyl)phenethyl)benzene-1,4-diamine (7g)

Using method A, *N^1^,N^1^*-dimethylbenzene-1,4-diamine (646 mg, 4.74 mmol), 1-(2-bromoethyl)-4-(trifluoromethyl)benzene (1.0 g, 3.96 mmol), potassium carbonate (1.64 g, 11.88 mmol), and potassium iodide (66 mg, 0.40 mmol) in acetonitrile (20 mL) gave 7g as a dark brown oil (300 mg, 25%); R*_f_* = 0.37 (*n-*Hex 3: EtOAC 1); ^1^H NMR (400 MHz, DMSO-*d_6_*) *δ* 7.66 (d, 2H, *J* = 8.00 Hz), 7.50 (d, 2H, *J* = 8.00 Hz), 6.65 (d, 2H, *J* = 8.96 Hz), 6.54 (d, 2H, *J* = 8.96 Hz), 5.02 (t, 1H, *J* = 6.00 Hz), 3.22 (q, 2H, *J* = 6.44 Hz), 2.91 (t, 2H, *J* = 7.12 Hz), 2.72 (s, 6H).

6.8. Preparation of *tert*-butyl (4-((2-methoxyphenethyl)amino)phenyl)(methyl)carbamate (7h)

Using method A, *tert*-butyl (4-aminophenyl)(methyl)carbamate (2.10 g, 9.45 mmol), 1-(2-bromoethyl)-2-methoxybenzene (2.03 g, 9.45 mmol), cesium carbonate (3.69 g, 11.34 mmol), and potassium iodide (157.7 mg, 0.95 mmol) in acetonitrile (20 mL) gave 7h as a yellow oil (1.21 g, 36%); R*_f_* = 0.42 (*n-*Hex 3: EtOAC 1); ^1^H NMR (400 MHz, DMSO-*d_6_*) *δ* 7.23–7.18 (m, 2H), 6.97 (d, 1H, *J* = 7.92 Hz), 6.93 (d, 2H, *J* = 8.64 Hz), 6.88 (t, 1H, *J* = 7.36 Hz), 6.54 (d, 2H, *J* = 8.76 Hz), 5.69 (t, 1H, *J* = 5.68 Hz), 3.81 (s, 3H), 3.18–3.13 (m, 2H), 3.07 (s, 3H), 2.80 (t, 2H, *J* = 6.84 Hz), 1.35 (s, 9H).

6.9. Preparation of *tert*-butyl (4-((3-methoxyphenethyl)amino)phenyl)(methyl)carbamate (7i)

Using method A, *tert*-butyl (4-aminophenyl)(methyl)carbamate (455 mg, 2.05 mmol), 1-(2-bromoethyl)-3-methoxybenzene (400 mg, 1.86 mmol), potassium carbonate (771 mg, 5.58 mmol), and potassium iodide (32 mg, 0.19 mmol) in acetonitrile (5 mL) gave 7i as a yellow oil (244 mg, 37%); R*_f_* = 0.41 (*n-*Hex 3: EtOAC 1); ^1^H NMR (400 MHz, DMSO-*d_6_*) *δ* 7.22 (t, 1H, *J* = 8.04 Hz), 6.95 (d, 2H, *J* = 8.60 Hz), 6.86–6.85 (m, 2H), 6.80-6.77 (m, 1H), 6.55 (d, 2H, *J* = 8.72 Hz), 5.65 (t, 1H, *J* = 5.64 Hz), 3.72 (s, 3H), 3.23 (q, 2H, *J* = 6.80 Hz), 3.08 (s, 3H), 2.81 (t, 2H, *J* = 7.60 Hz), 1.36 (s, 9H).

6.10. Preparation of *tert*-butyl (4-((4-methoxyphenethyl)amino)phenyl)(methyl)carbamate (7j)

Using method A, *tert*-butyl (4-aminophenyl)(methyl)carbamate (340 mg, 1.53 mmol), 1-(2-bromoethyl)-4-methoxybenzene (300 mg, 1.39 mmol), potassium carbonate (578 mg, 4.18 mmol), and potassium iodide (23 mg, 0.14 mmol) in acetonitrile (5 mL) gave 7j as a yellow oil (244 mg, 37%); R*_f_* = 0.41 (*n-*Hex 3: EtOAC 1); ^1^H NMR (400 MHz, DMSO-*d_6_*) *δ* 7.20 (d, 2H, *J* = 8.52 Hz), 6.94 (d, 2H, *J* = 8.60 Hz), 6.87 (d, 2H, *J* = 8.52 Hz), 6.54 (d, 2H, *J* = 8.72 Hz), 5.63 (t, 1H, *J* = 5.60 Hz), 3.73 (s, 3H), 3.21–3.16 (m, 2H), 3.08 (s, 3H), 2.77 (t, 2H, *J* = 7.24), 1.36 (s, 9H).

6.11. Preparation of *tert*-butyl methyl(4-(phenethylamino)phenyl)carbamate (7k)

Using method A, *tert*-butyl (4-aminophenyl)(methyl)carbamate (500 mg, 2.25 mmol), (2-bromoethyl)benzene (416 mg, 2.25 mmol), cesium carbonate (879.4 mg, 2.70 mmol), and potassium iodide (37 mg, 0.23 mmol) in acetonitrile (10 mL) gave 7k as a yellow oil (160.4 mg, 20%); R*_f_* = 0.32 (*n-*Hex 9: EtOAC 1); ^1^H NMR (400 MHz, DMSO-*d_6_*) *δ* 7.32-7.27 (m, 4H), 7.22-7.18 (m, 1H), 6.93 (d, 2H, *J* = 8.68 Hz), 6.54 (d, 2H, *J* = 8.80 Hz), 5.65 (t, 1H, *J* = 5.72 Hz), 3.25–3.19 (m, 2H), 3.07 (s, 3H), 2.83 (t, 2H, *J* = 7.76 Hz), 1.35 (s, 9H).

6.12. Preparation of *tert*-butyl methyl (4-((3-(trifluoromethyl)phenethyl)amino)phenyl) carbamate (7l)

Using method A, *tert*-butyl (4-aminophenyl)(methyl)carbamate (440 mg, 1.98 mmol), 1-(2-bromoethyl)-3-(trifluoromethyl)benzene (500 mg, 1.98 mmol), potassium carbonate (821 mg, 5.94 mmol), and potassium iodide (33 mg, 0.20 mmol) in acetonitrile (7 mL) to give 7l as an off white solid (395 mg, 51%); R*_f_* = 0.44 (*n-*Hex 3: EtOAc 1); ^1^H NMR (400 MHz, DMSO-*d_6_*) *δ* 7.64 (s, 1H), 7.61-7.52 (m, 3H), 6.95 (d, 2H, *J* = 8.72 Hz), 6.56 (d, 2H, *J* = 8.80 Hz), 5.69 (t, 1H, *J* = 5.68 Hz), 3.30–3.25 (m, 2H), 3.08 (s, 3H), 2.94 (t, 2H, *J* = 7.16 Hz).

6.13. Preparation of *tert*-butyl methyl (4-((2-(trifluoromethyl)phenethyl)amino)phenyl) carbamate (7m)

Using method A, *tert*-butyl (4-aminophenyl)(methyl)carbamate (295 mg, 1.33 mmol), 1-(2-bromoethyl)-2-(trifluoromethyl)benzene (337 mg, 1.33 mmol), potassium carbonate (552 mg, 3.99 mmol), and potassium iodide (22 mg, 0.13 mmol) in acetonitrile (7 mL) to give 7m as an off white solid (319 mg, 41%); R*_f_* = 0.45 (*n-*Hex 3: EtOAc 1); ^1^H NMR (400 MHz, DMSO-*d_6_*) *δ* 7.73 (d, 1H, *J* = 7.72 Hz), 7.65 (t, 1H, *J* = 7.36 Hz), 7.58 (d, 1H, *J* = 7.60 Hz), 7.46 (t, 1H, *J* = 7.48 Hz), 6.97 (d, 2H, *J* = 8.64 Hz), 6.58 (d, 2H, *J* = 8.76 Hz), 5.85 (t, 1H, *J* = 5.84 Hz), 3.29–3.24 (m, 2H), 3.09 (s, 3H), 3.01 (t, 2H, *J* = 7.96 Hz), 1.37 (s, 9H).

7. General procedure for final KDS compounds (8a–8h) (Method B)

To a solution of amine containing compounds (7g–7m) (1.0 equiv) was dissolved in anhydrous dichloromethane was added 4.0 M hydrochloric acid in dioxane (4.0–6.0 equiv). The reaction mixture was stirred at room temperature for 48 h. The precipitate was filtered and the filtercake was obtained to afford the desired compound.

7.1. Preparation of *N*^1^-methyl-*N*^4^-(4-(trifluoromethyl)phenethyl)benzene-1,4-diamine hydrochloride salt (8a, KDS12017)

Using method B, *tert*-butyl methyl(4-((4-(trifluoromethyl)phenethyl)amino)phenyl) carbamate (316 mg, 0.80 mmol), 4.0 M hydrochloric acid in dioxane (1.20 mL, 4.80 mmol) in dichloromethane (5 mL) to give 8a as an off white solid (270 mg, 92%); mp 246–250 °C; ^1^H NMR (400 MHz, DMSO-*d_6_*) *δ* 10.58 (br, 1H), 7.66 (d, 2H, *J* = 7.96 Hz), 7.50 (d, 2H, *J* = 7.92 Hz), 7.13 (s, 2H), 6.82 (s, 2H), 3.34 (t, 2H, *J* = 7.04 Hz), 2.96 (t, 2H, *J* = 7.40 Hz), 2.79 (s, 3H); ^13^C NMR (100 MHz, DMSO-*d_6_*) *δ* 144.51, 130.07, 129.81, 127.50 (q, *J*_C-F_ = 31.63 Hz), 126.24, 125.62 (q, *J*_C-F_ = 3.81 Hz), 123.54, 46.40 (**C**H_2_), 35.74 (**C**H_2_), 33.92 (**C**H_3_); HPLC purity: 8.1 min, >99.9%; HRMS (M+H) (ESI^+^) 295.14157 [M + H]^+^ (calcd for C_16_H_17_F_3_N_2_H^+^ 295.141658).

7.2. Preparation of *N*^1^*,N*^1^-dimethyl-*N*^4^-(4-(trifluoromethyl)phenethyl)benzene-1,4-diamine hydrochloride salt (8b, KDS12008)

Using method B, *N*^1^,*N*^1^-dimethyl-*N*^4^-(4-(trifluoromethyl)phenethyl)benzene-1,4-diamine (300 mg, 0.97 mmol), 4.0 M hydrochloric acid in dioxane (0.73 mL) in dichloromethane (7 mL) to give 8b as an off white solid; mp 216–241 °C ^1^H NMR (400 MHz, DMSO-*d_6_*) *δ* 7.89 (br, 2H) 7.66 (d, 2H, *J* = 8.00 Hz), 7.51 (d, 2H, *J* = 7.96 Hz), 7.40 (s, 2H), 6.89 (s, 2H), 3.36 (t, 2H, *J* = 7.28 Hz), 3.01 (s, 6H), 2.98 (t, 3H, *J* = 7.04 Hz); ^13^C NMR (100 MHz, DMSO-*d_6_*) *δ* 144.47, 130.07, 128.94, 127.50 (q, *J*_C-F_ = 31.48 Hz), 126.24, 125.62 (q, *J*_C-F_ = 3.45 Hz), 123.54, 46.21 (**C**H_2_), 45.19 (**C**H_3_), 33.90 (**C**H_2_); HPLC purity: 8.0 min, 99.7%; HRMS (M+H) (ESI^+^) 309.15714 [M + H]^+^ (calcd for C_17_H_19_F_3_N_2_H^+^ 309.157308).

7.3. Preparation of *N*^1^-(2-methoxyphenethyl)-*N*^4^-methylbenzene-1,4-diamine hydrochloride salt (8c, KDS12025)

Using method B, *tert*-butyl (4-((2-methoxyphenethyl)amino)phenyl)(methyl) carbamate (586 mg, 1.64 mmol), 4.0 M hydrochloric acid in dioxane (1.65 mL, 6.58 mmol) in dichloromethane (10 mL) to give 8c as a white solid (436 mg, 81%); mp 210–212 °C; ^1^H NMR (400 MHz, DMSO-*d_6_*) *δ* 7.25-7.17 (m, 3H), 7.09 (s, 2H), 6.98 (d, 1H, *J* = 8.04 Hz), 6.89 (t, 3H, *J* = 7.36 Hz), 3.80 (s, 3H), 3.24 (t, 2H, *J* = 7.12 Hz), 2.84 (t, 2H, *J* = 8.16 Hz), 2.79 (s, 3H); ^13^C NMR (100 MHz, DMSO-*d_6_*) *δ* 157.60, 130.42, 128.49, 126.16, 124.94, 124.79, 122.13, 120.86, 119.99, 111.19, 55.80 (**C**H_3_), 47.45 (**C**H_2_), 35.50 (**C**H_2_), 27.96 (**C**H_3_); HPLC purity: 6.8 min, 99.7%; HRMS (M+H) (ESI^+^) 257.16479 [M + H]^+^ (calcd for C_16_H_20_N_2_OH^+^ 257.164839).

7.4. Preparation of *N*^1^-(3-methoxyphenethyl)-*N*^4^-methylbenzene-1,4-diamine hydrochloride salt (8d)

Using method B, *tert*-butyl (4-((3-methoxyphenethyl)amino)phenyl)(methyl) carbamate (240 mg, 0.67 mmol), 4.0 M hydrochloric acid in dioxane (1.01 mL, 4.02 mmol) in dichloromethane (10 mL) to give 8d as a white solid (193 mg, 87%); mp 245–249 °C; ^1^H NMR (400 MHz, DMSO-*d_6_*) *δ* 9.18 (br, 4H), 7.29 (d, 2H, *J* = 7.29 Hz), 7.22 (t, 1H, *J* = 7.92 Hz), 7.16 (d, 2H, *J* = 7.84 Hz), 6.84-6.78 (m, 3H), 3.74 (s, 3H), 3.37 (t, 2H, *J* = 8.24 Hz), 2.92 (t, 2H, *J* = 7.44 Hz), 2.80 (s, 3H); ^13^C NMR (100 MHz, DMSO-*d_6_*) *δ* 159.82, 140.34, 129.92, 124.58, 124.46, 121.48, 121.33, 118.82, 114.75, 112.41, 55.44 (**C**H_3_), 48.30 (**C**H_2_), 35.34 (**C**H_2_), 33.46 (**C**H_3_); HPLC purity: 6.7 min, >99.9%; HRMS (M+H) (ESI^+^) 257.16434 [M + H]^+^ (calcd for C_16_H_20_F_3_N_2_OH^+^ 257.164839).

7.5. Preparation of *N*^1^-(4-methoxyphenethyl)-*N*^4^-methylbenzene-1,4-diamine (8e)

Using method B, *tert*-butyl (4-((4-methoxyphenethyl)amino)phenyl)(methyl) carbamate (586 mg, 1.64 mmol), 4.0 M hydrochloric acid in dioxane (1.65 mL, 6.58 mmol) in dichloromethane (10 mL) to give 8e as a white solid (436 mg, 81%); mp 220–245 °C; ^1^H NMR (400 MHz, DMSO-*d_6_*) *δ* 10.10 (br, 4H), 7.34 (d, 2H, *J* = 8.56 Hz), 7.25 (d, 2H, *J* = 8.12 Hz), 7.18 (d, 2H, *J* = 8.48 Hz), 6.87 (d, 2H, *J* = 8.48 Hz), 3.72 (s, 3H), 3.34 (t, 2H, *J* = 8.28 Hz), 2.90 (t, 2H, *J* = 7.48 Hz), 2.81 (s, 3H); ^13^C NMR (100 MHz, DMSO-*d_6_*) *δ* 158.38, 13036, 130.12, 124.77, 124.64, 121.72, 119.86, 114.36, 55.50 (**C**H_3_), 49.24(**C**H_2_), 35.33(**C**H_2_), 32.28 (**C**H_3_); HPLC purity: 6.6 min, 99.4%; HRMS (M+H) (ESI^+^) 257.16418 [M + H]^+^ (calcd for C_16_H_20_N_2_OH^+^ 257.164839).

7.6. Preparation of *N*^1^-methyl-*N*^4^-phenethylbenzene-1,4-diamine hydrochloride salt (8f)

Using method B, *tert*-butyl methyl(4-(phenethylamino)phenyl)carbamate (160 mg, 0.49 mmol), 4.0 M hydrochloric acid in dioxane (0.73 mL) in dichloromethane (5 mL) to give 8f as an off white solid (111 mg, 76%); mp 246-247 °C; ^1^H NMR (400 MHz, DMSO-*d_6_*) *δ* 10.32 (br, 1H), 7.33-7.26 (m, 4H), 7.24-7.20 (m, 1H), 7.08 (s, 2H), 6.88 (s, 2H), 3.31 (t, 2H, *J* = 7.40 Hz), 2.87 (t, 2H, *J* = 7.92 Hz), 2.78 (s, 3H); ^13^C NMR (100 MHz, DMSO-*d_6_*) *δ* 139.1, 129.1, 128.9, 126.8, 121.0, 117.6, 47.7 (**C**H_2_), 35.2 (**C**H_2_), 33.8 (**C**H_3_); HPLC purity: 7.0 min, >99.9%; HRMS (M+H) (ESI^+^) 228.15521 [M + H]^+^ (calcd for C_15_H_18_N_2_H^+^ 228.15482).

7.7. Preparation of *N*^1^-methyl-*N*^4^-(3-(trifluoromethyl)phenethyl)benzene-1,4-diamine hydrochloride salt (8g)

Using method B, *tert*-butyl methyl(4-((3-(trifluoromethyl)phenethyl)amino)phenyl) carbamate (395 mg, 0.34 mmol), 4.0 M hydrochloric acid in dioxane (1.5 mL, 6.00 mmol) in dichloromethane (7 mL) to give 8g as an off white solid (351 mg, 96%); mp 210–246 °C; ^1^H NMR (400 MHz, MeOD-*d_4_*) *δ* 7.59-7.7.50 (m, 4H), 7.43 (d, 2H, *J* = 8.92 Hz), 7.30 (d, 2H, *J* = 6.84 Hz), 3.62–3.58 (m, 2H), 3.12 (t, 2H, *J* = 8.00 Hz), 3.30 (s, 3H); ^13^C NMR (100 MHz, DMSO-*d_6_*) *δ* 140.66, 133.55, 129.84, 125.84, 123.60, 122.06, 117.98, 47.55 (**C**H_2_), 35.80 (**C**H_2_), 33.43 (**C**H_3_); HPLC purity: 7.8 min, >99.9%; HRMS (M+H) (ESI^+^) 295.14127 [M + H]^+^ (calcd for C_16_H_17_F_3_N_2_H^+^ 295.141658).

7.8. Preparation of *N*^1^-methyl-*N*^4^-(2-(trifluoromethyl)phenethyl)benzene-1,4-diamine hydrochloride salt (8h)

Using method B, *tert*-butyl methyl(4-((2-(trifluoromethyl)phenethyl)amino)phenyl) carbamate (80 mg, 0.20 mmol), 4.0 M hydrochloric acid in dioxane (0.30 mL, 1.22 mmol) in dichloromethane (5 mL) to give 8h as an off white solid (53.1 mg, 72%); mp 221–246 °C; ^1^H NMR (400 MHz, DMSO-*d_6_*) *δ* 9.93 (br, 4H), 7.71 (d, 1H, *J* = 7.88 Hz), 7.65 (t, 1H, *J* = 7.48 Hz), 7.58 (d, 1H, *J* = 7.56 Hz), 7.46 (t, 1H, *J* = 7.48 Hz), 7.35 (d, 2H, *J* = 8.44 Hz), 7.08 (d, 2H, *J* = 7.96 Hz), 3.36 (t, 2H, *J* = 7.24 Hz), 3.11 (t, 2H, *J* = 7.20 Hz), 2.82 (s, 3H); ^13^C NMR (100 MHz, DMSO-*d_6_*) *δ* 137.36, 133.13, 132.21, 127.69 (q, *J*_C-F_ = 32.55 Hz), 127.55, 126.27 (q, *J*_C-F_ = 5.63 Hz), 124.91 (q, *J*_C-F_ = 255.06 Hz), 122.50, 117.23, 47.13 (**C**H_2_), 35.94 (**C**H_2_), 30.65 (**C**H_3_); HPLC purity: 7.7 min, 99.2%; HRMS (M+H) (ESI^+^) 295.14130 [M + H]^+^ (calcd for C_16_H_17_F_3_N_2_H^+^ 295.141658).

**II. ^1^H and ^13^C-NMR spectra of synthesized products**

**Chemical Information, CI 1a.** ^1^H and ^13^C NMR spectra of **7b**


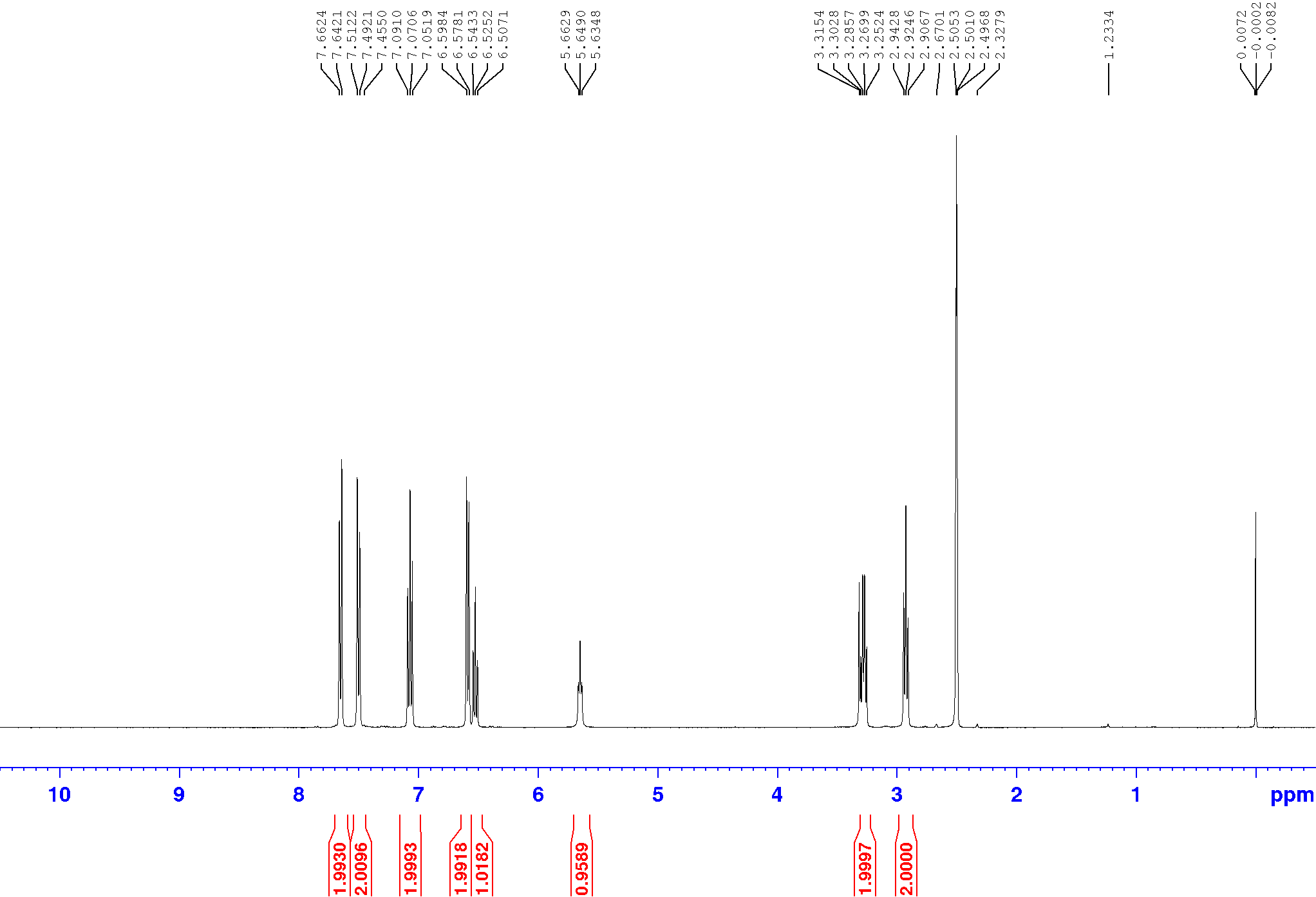


W

S

S : solvent

W : water

I : impurity

**DMSO-*d_6_***

**7b**


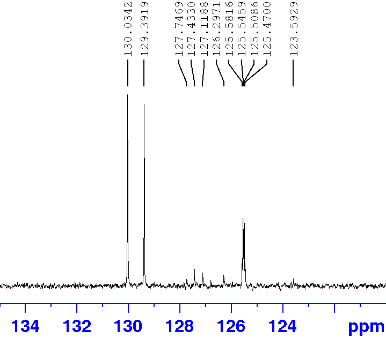

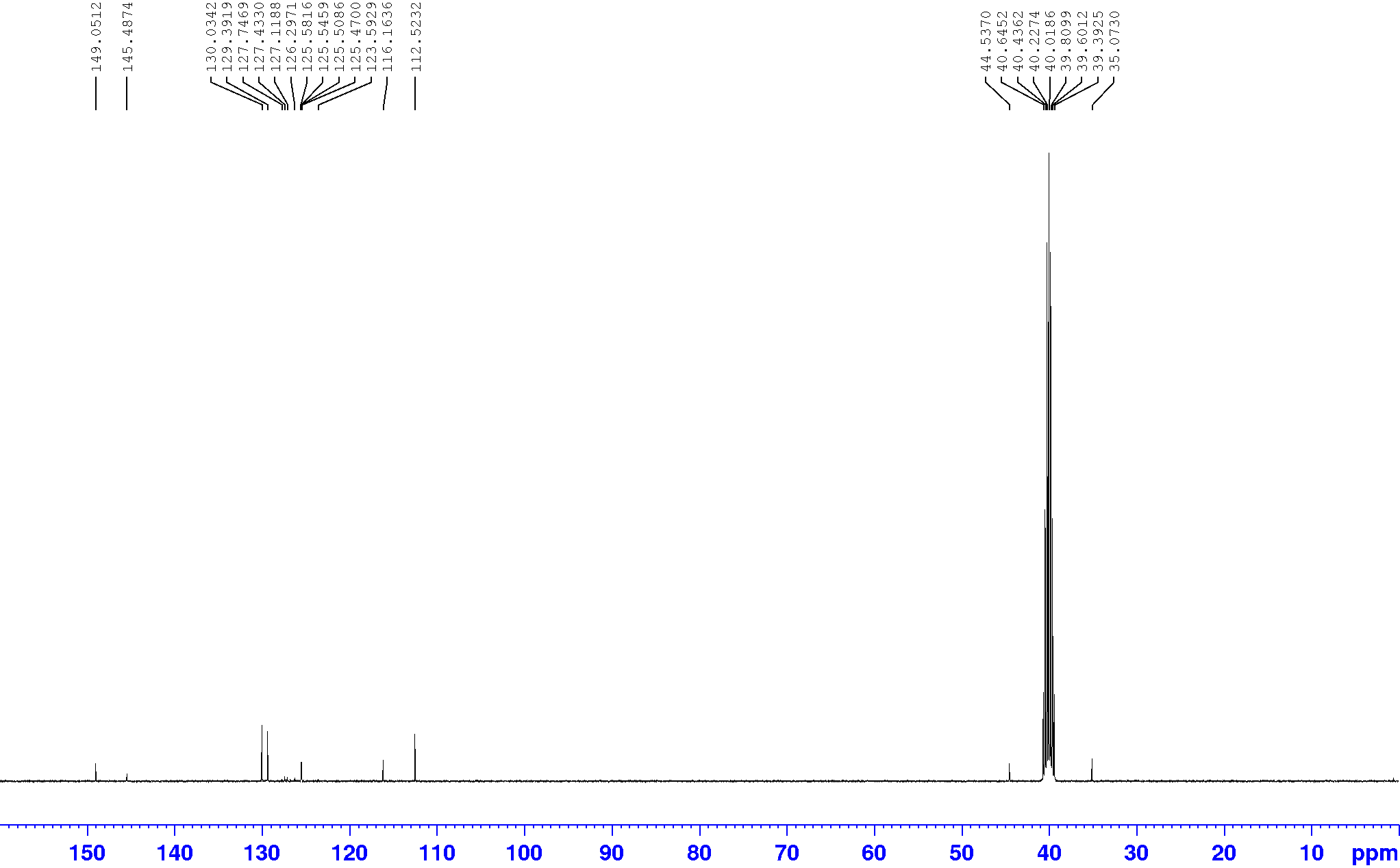


S

S : solvent

W : water

I : impurity

**DMSO-*d_6_***

**7b**

**CI 1b.** ^1^H and ^13^C NMR spectra of **7c**


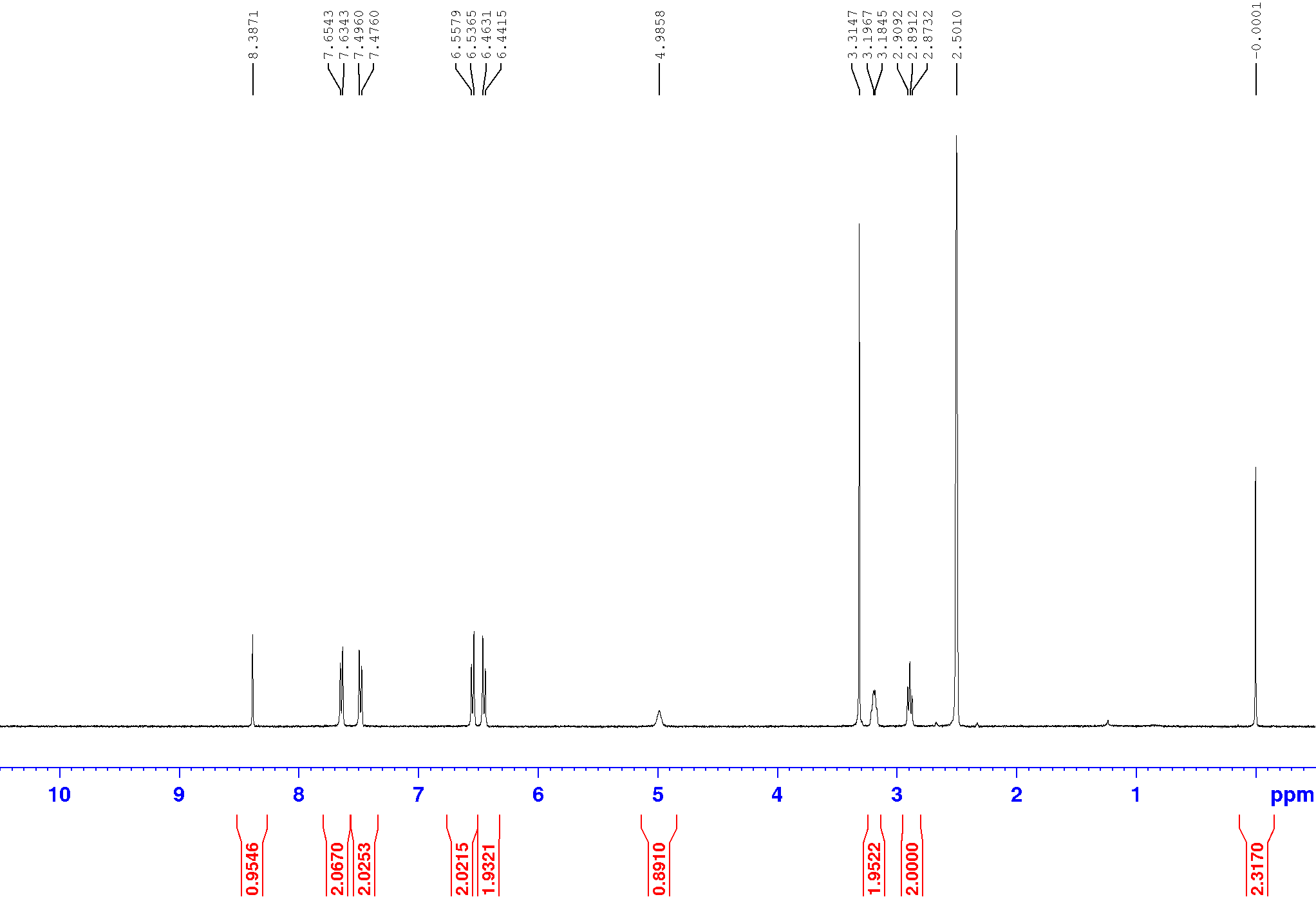


W

S : solvent

W : water

I : impurity

S

**DMSO-*d_6_***

**7c**


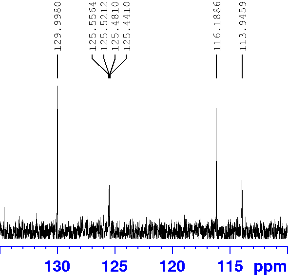

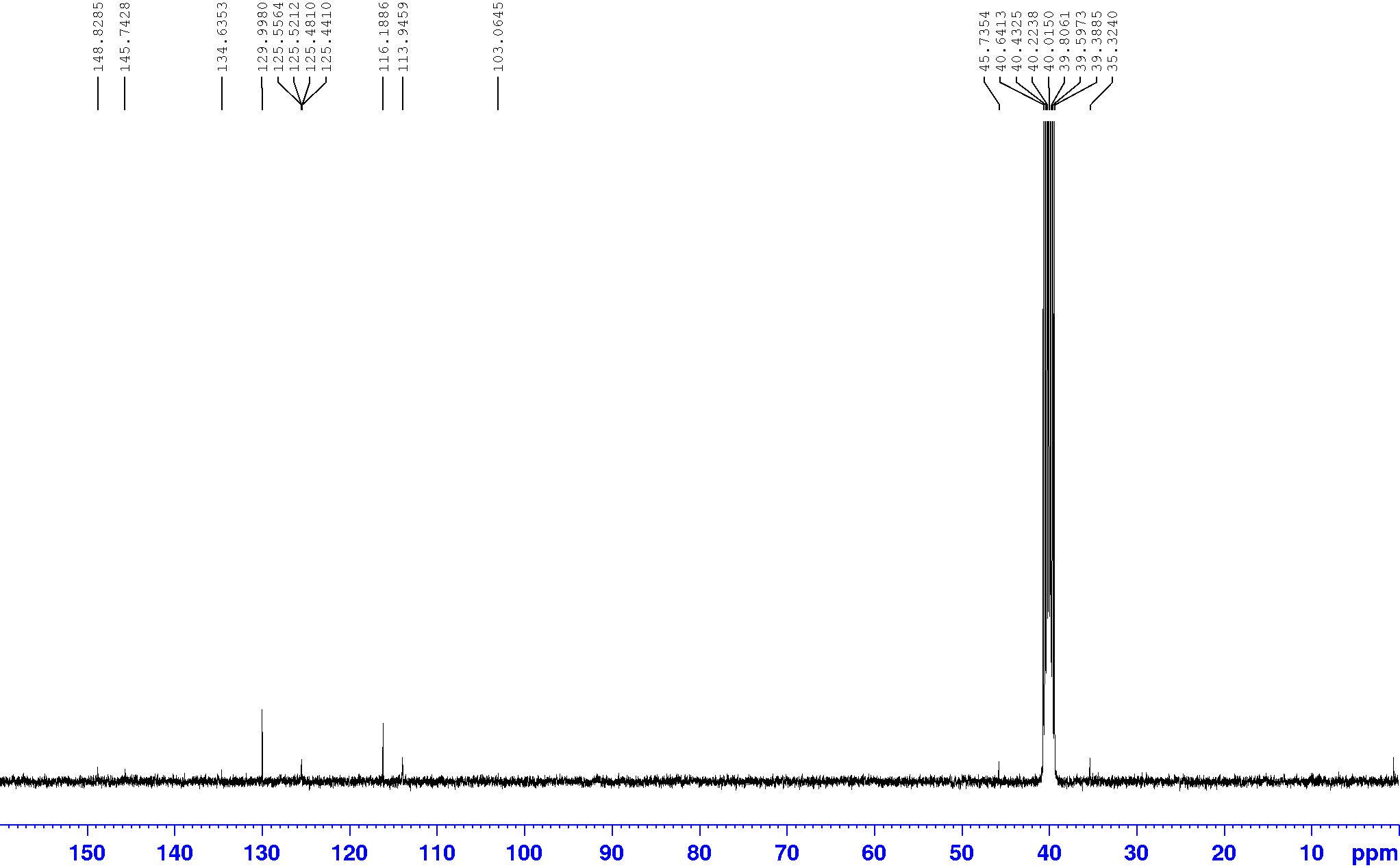


S

S : solvent

W : water

I : impurity

**DMSO-*d_6_***

**7c**

**CI 1c.** ^1^H and ^13^C NMR spectra of **7d**


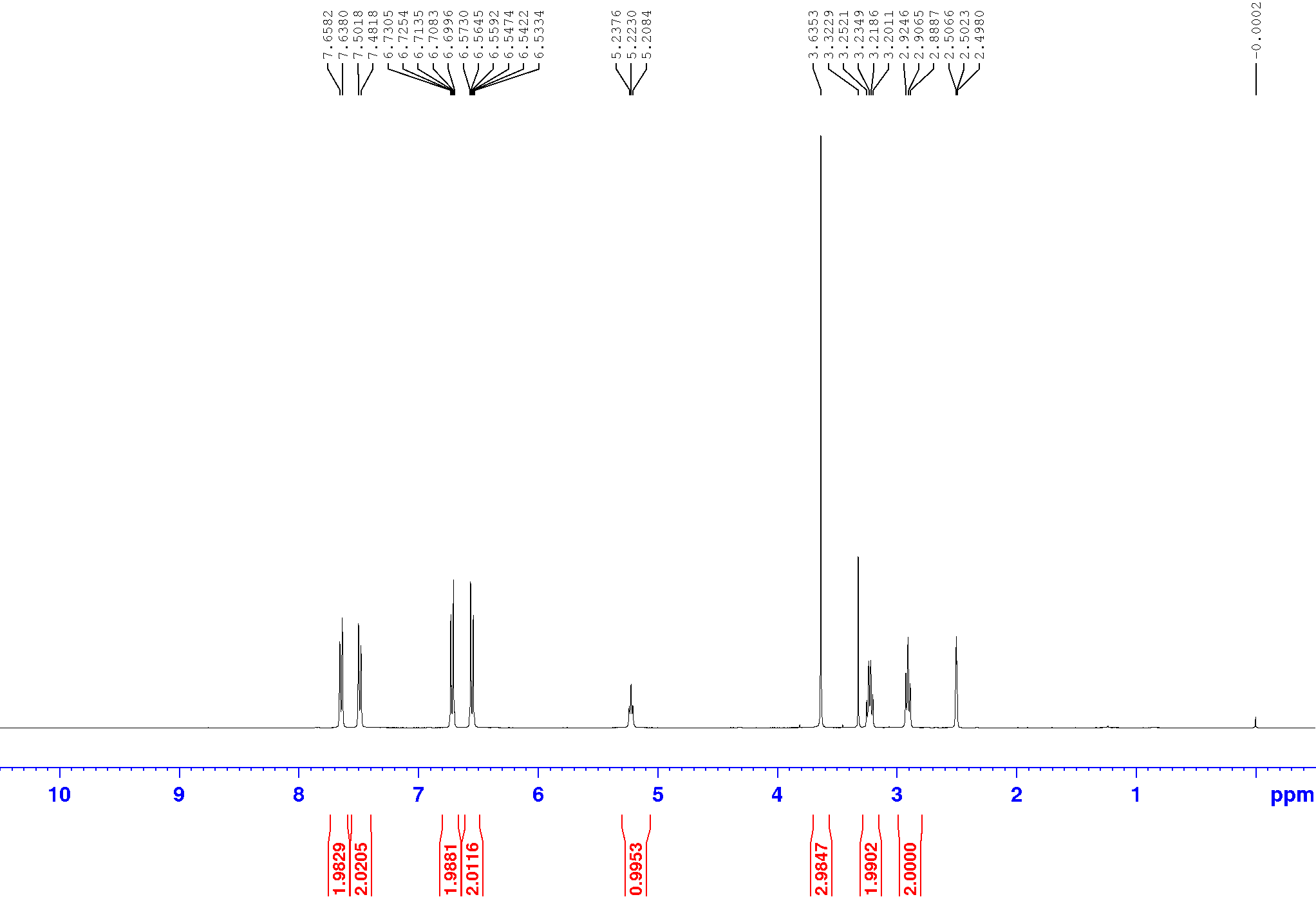


**7d**

**DMSO-*d_6_***

S

W

S : solvent

W : water

I : impurity


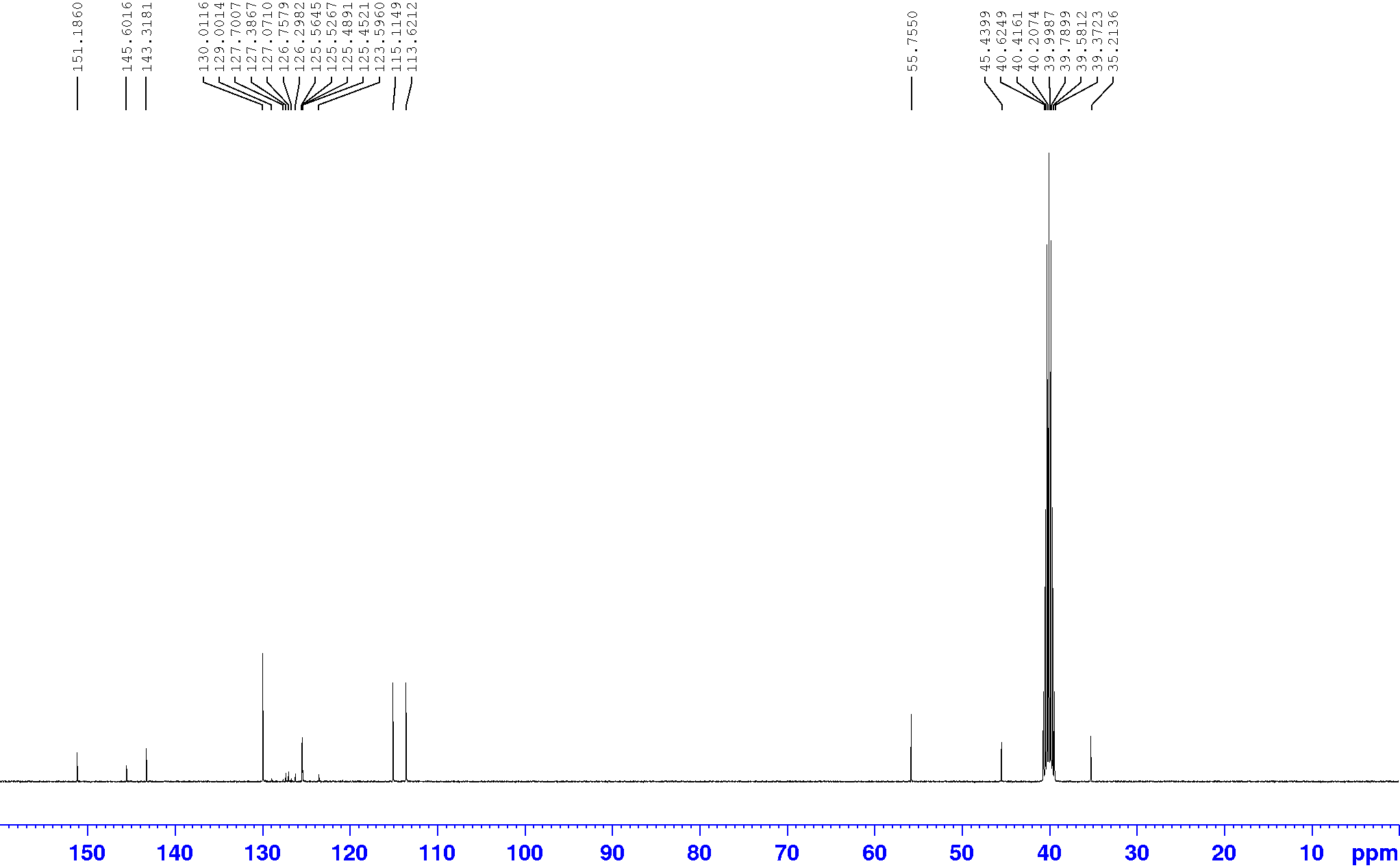


S

S : solvent

W : water

I : impurity

**7d**

**DMSO-*d_6_***

**CI 1d.** ^1^H and ^13^C NMR spectra of **7e**


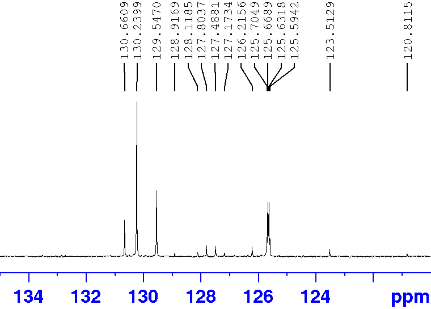

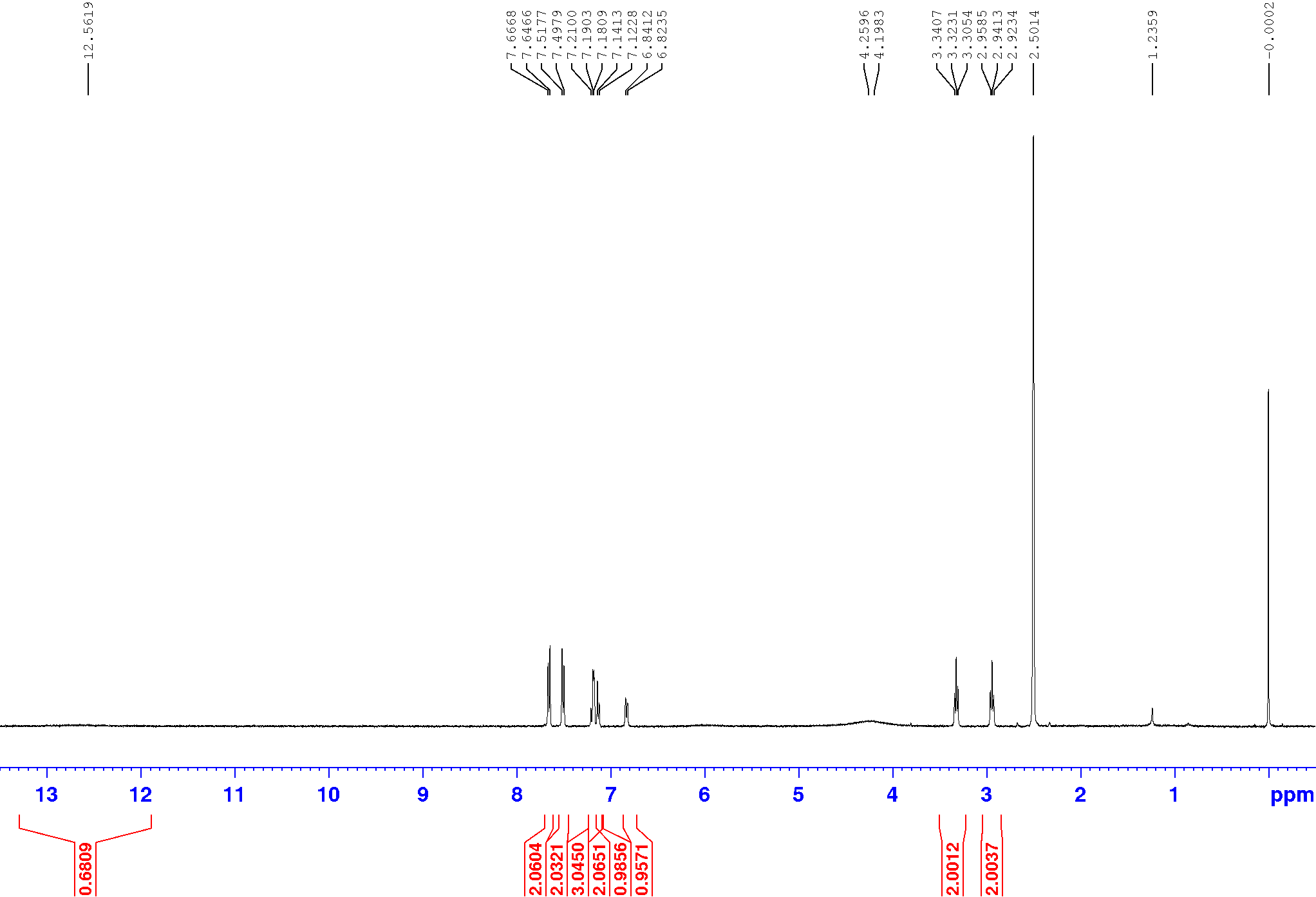

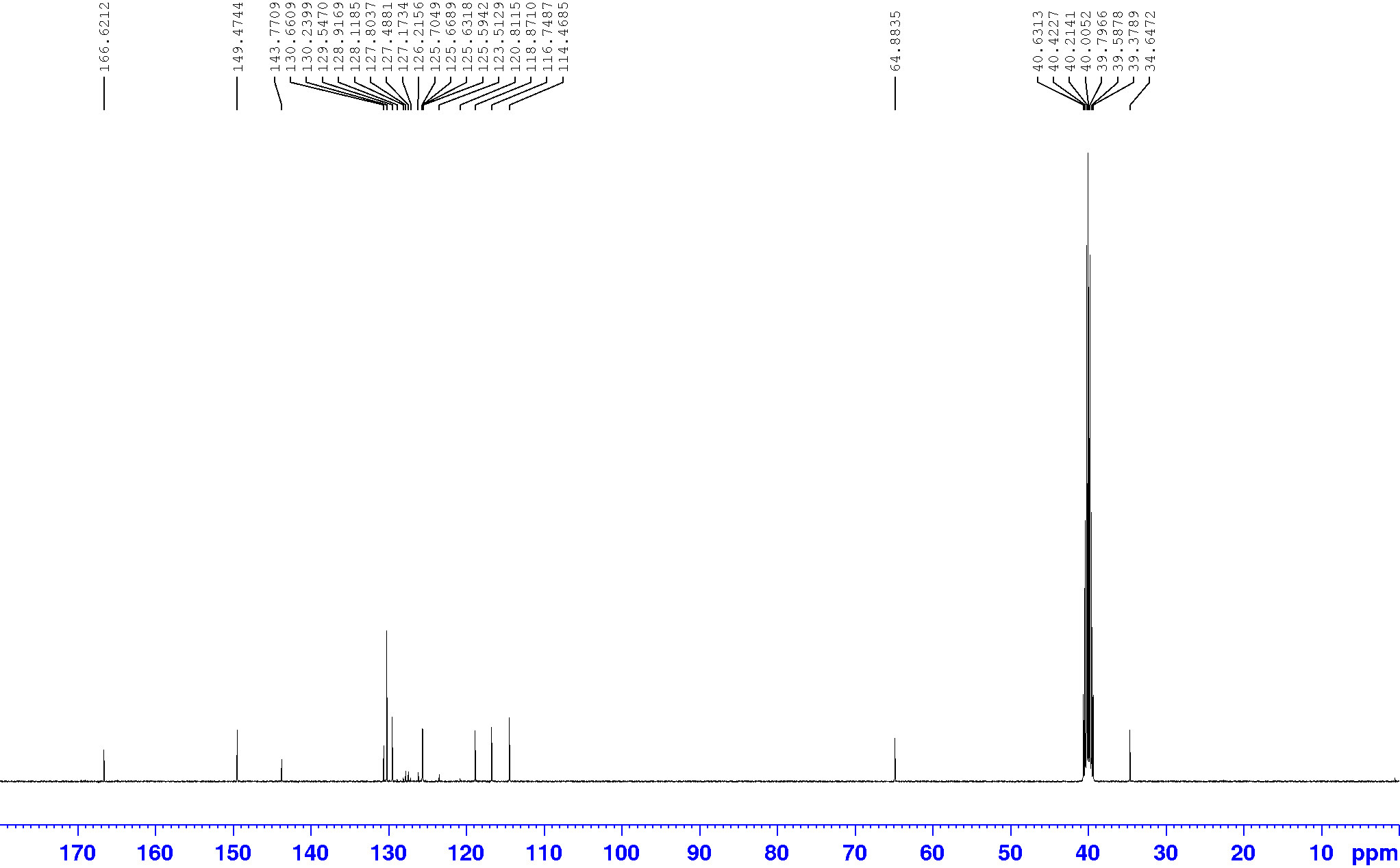


S

S : solvent

W : water

I : impurity

S

S : solvent

W : water

I : impurity

**7e**

**DMSO-*d_6_***

**DMSO-*d_6_***

**7e**

**CI 1e.** ^1^H and ^13^C NMR spectra of **7f**


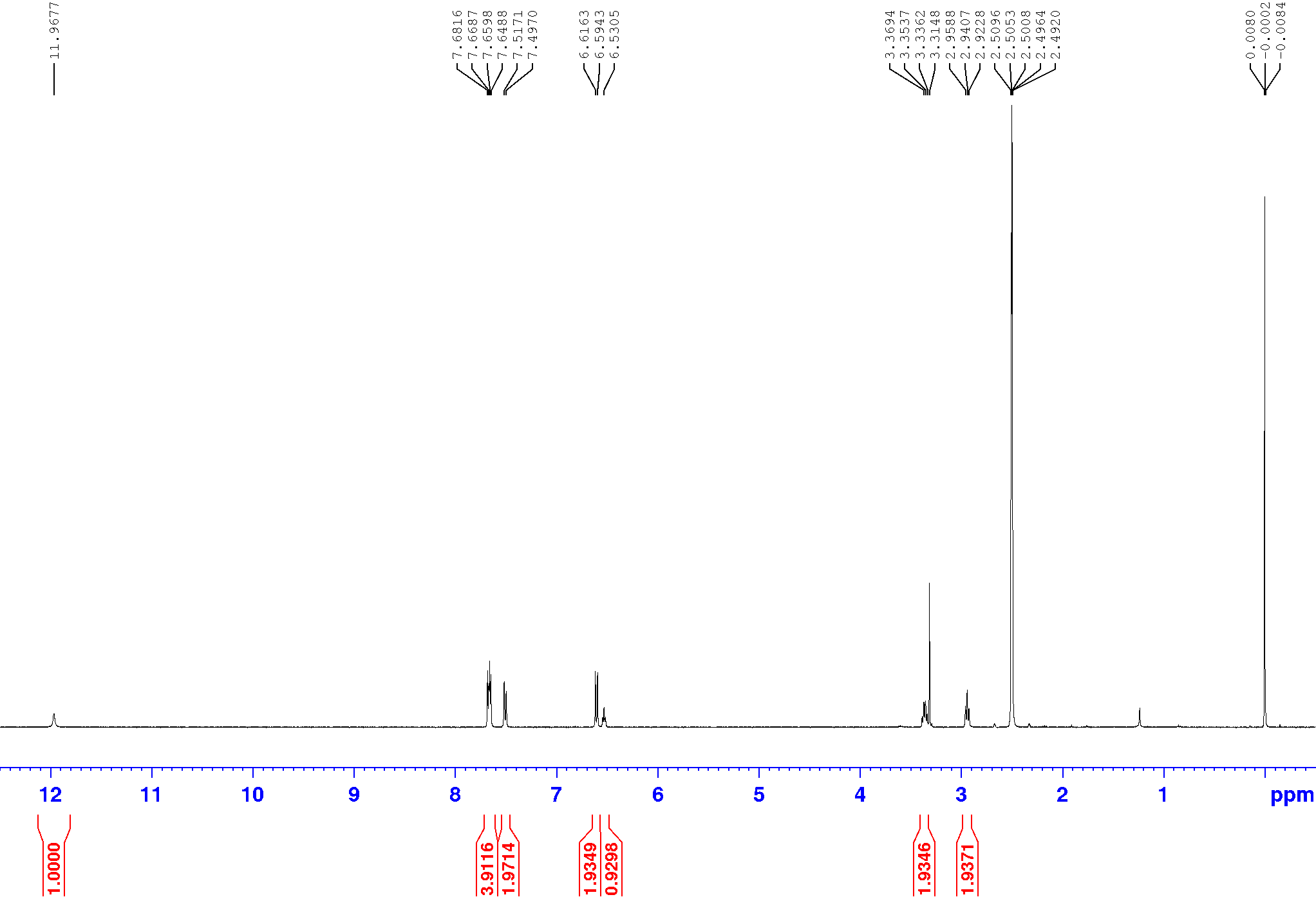


S : solvent

W : water

I : impurity

W

S

S

**DMSO-*d_6_***

**DMSO-*d_6_***

**7f**


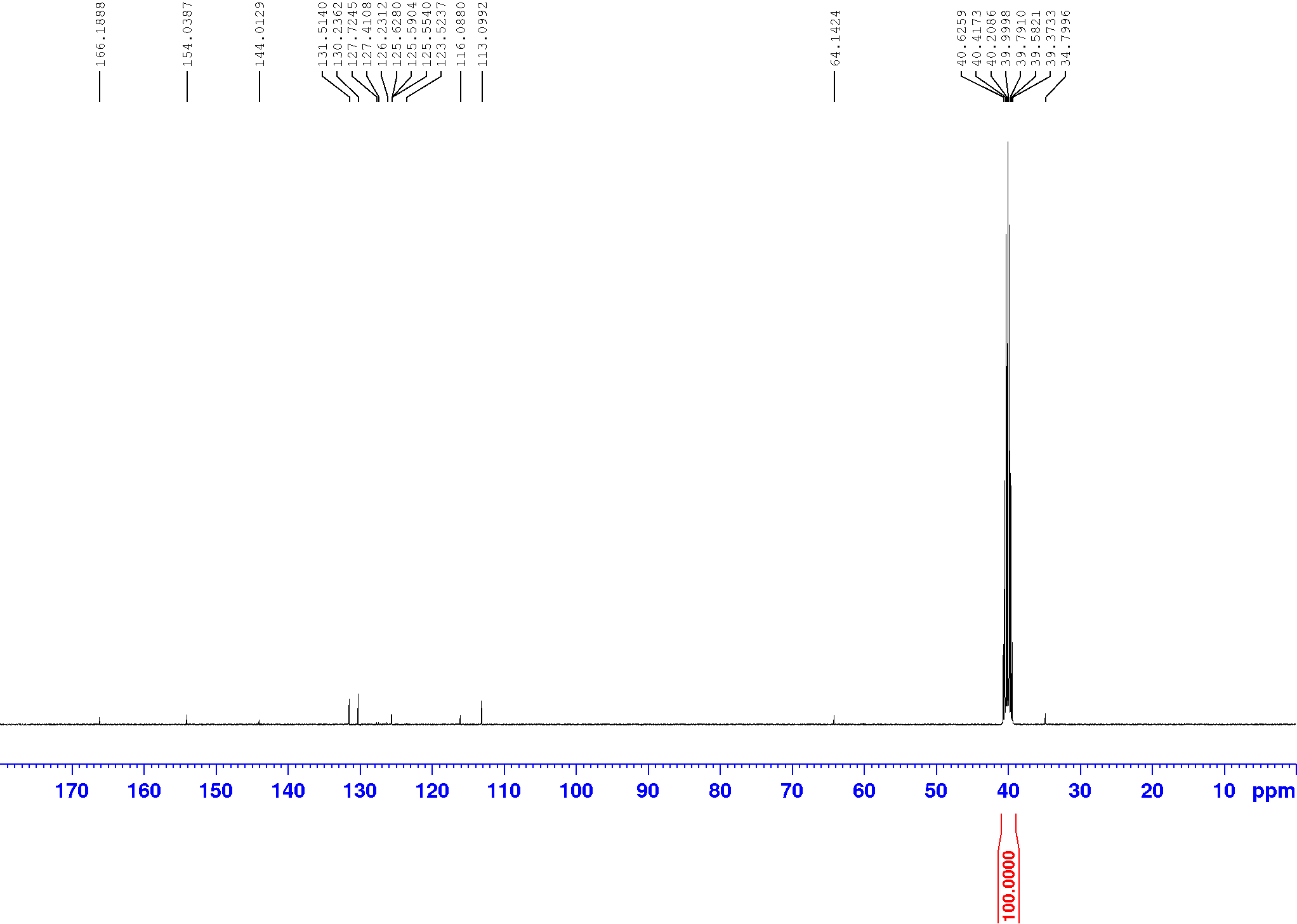


**7f**

S : solvent

W : water

I : impurity

**CI 1f.** ^1^H and ^13^C NMR spectra of **8a (KDS12017)**


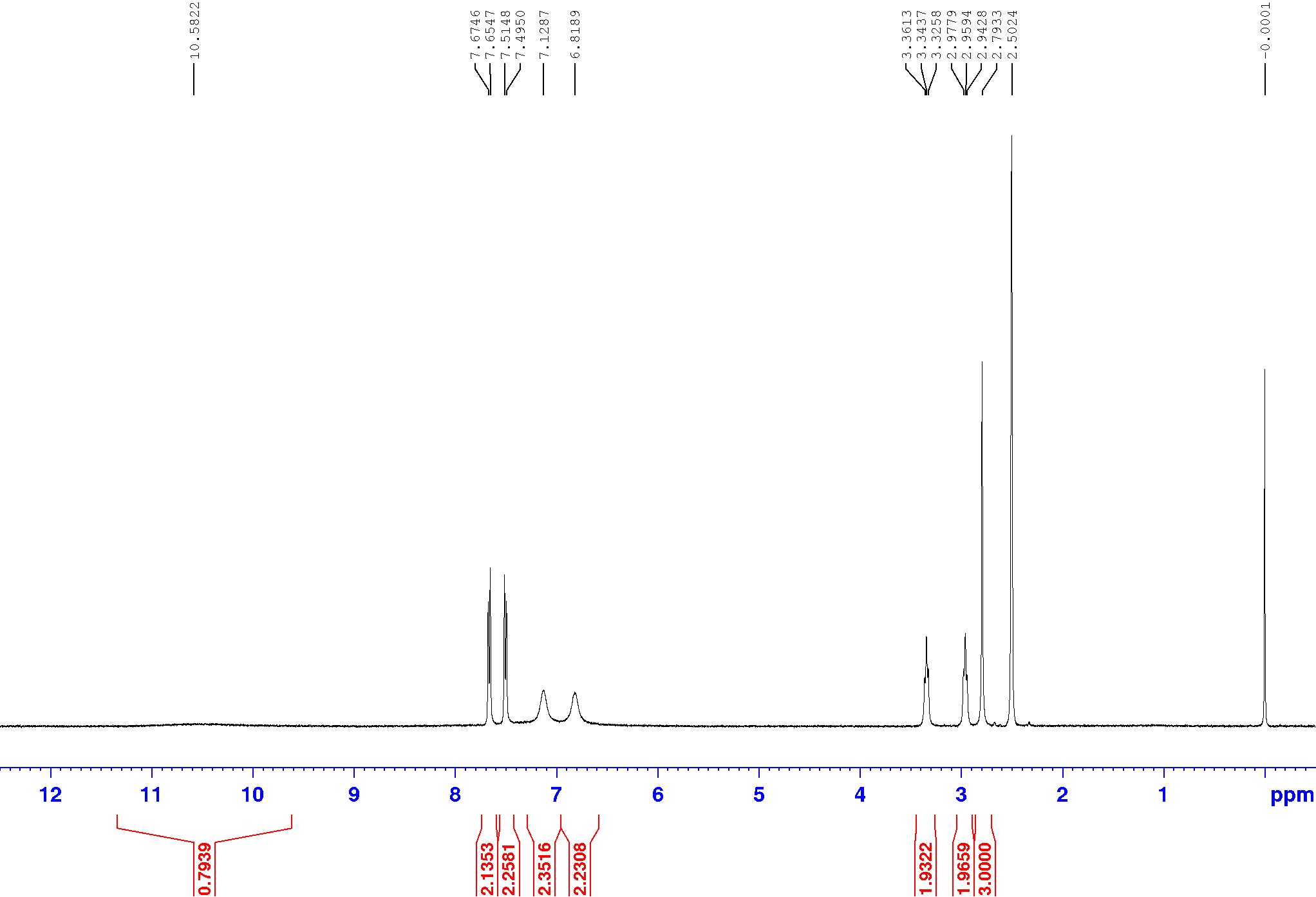


S

S : solvent

W : water

I : impurity

**DMSO-*d_6_***

**8a (KDS12017)**


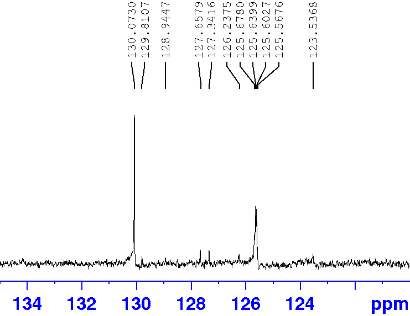

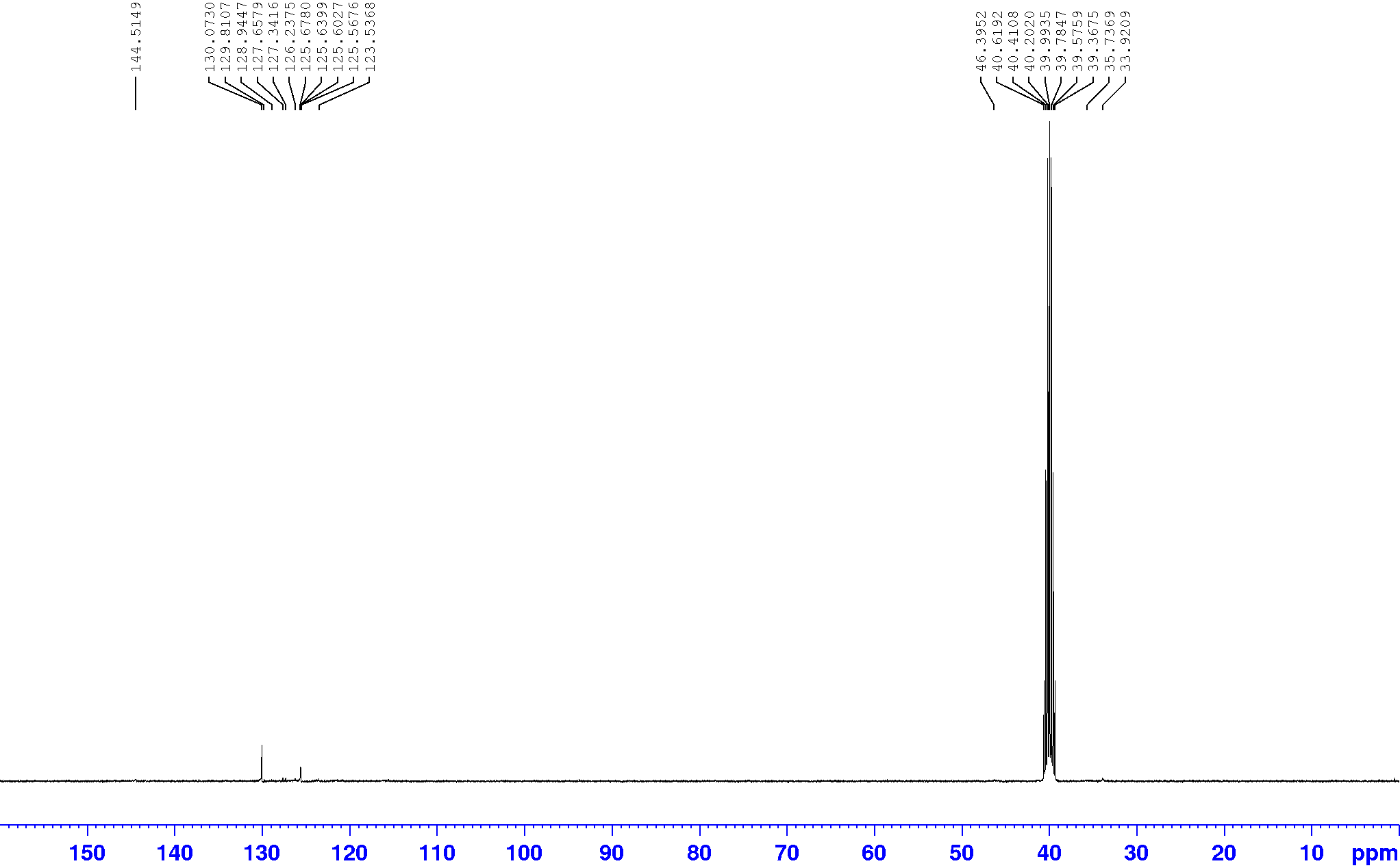


S

S : solvent

W : water

I : impurity

**8a (KDS12017)**

**DMSO-*d_6_***

**CI 1g.** ^1^H and ^13^C NMR spectra of **8b (KDS12008)**


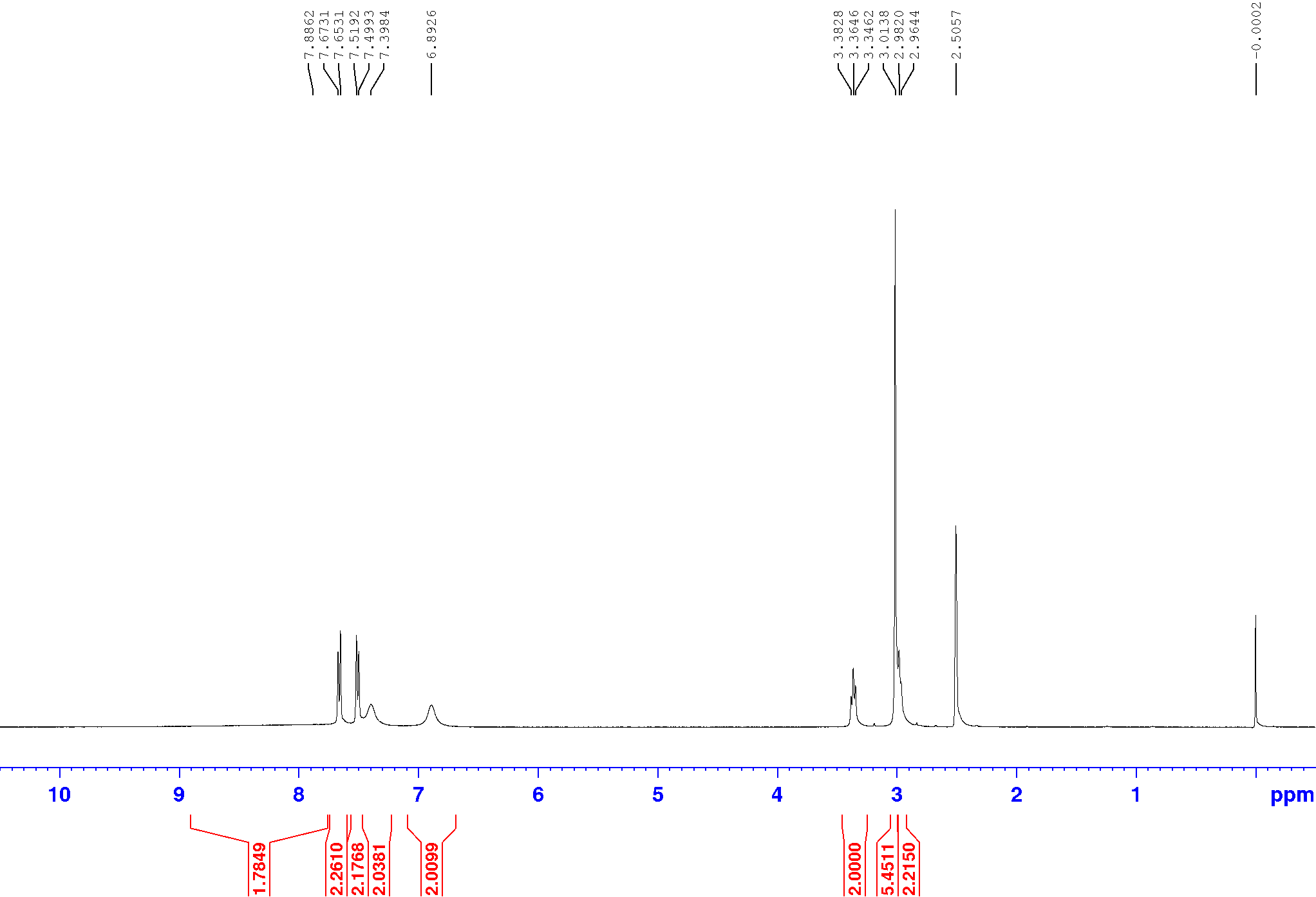


S

S : solvent

W : water

I : impurity

**8b (KDS12008)**

**DMSO-*d_6_***


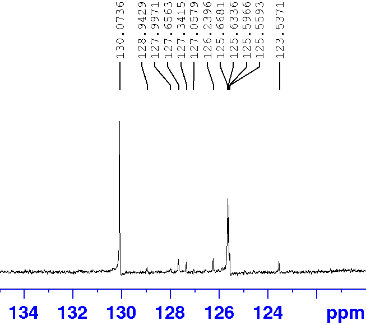

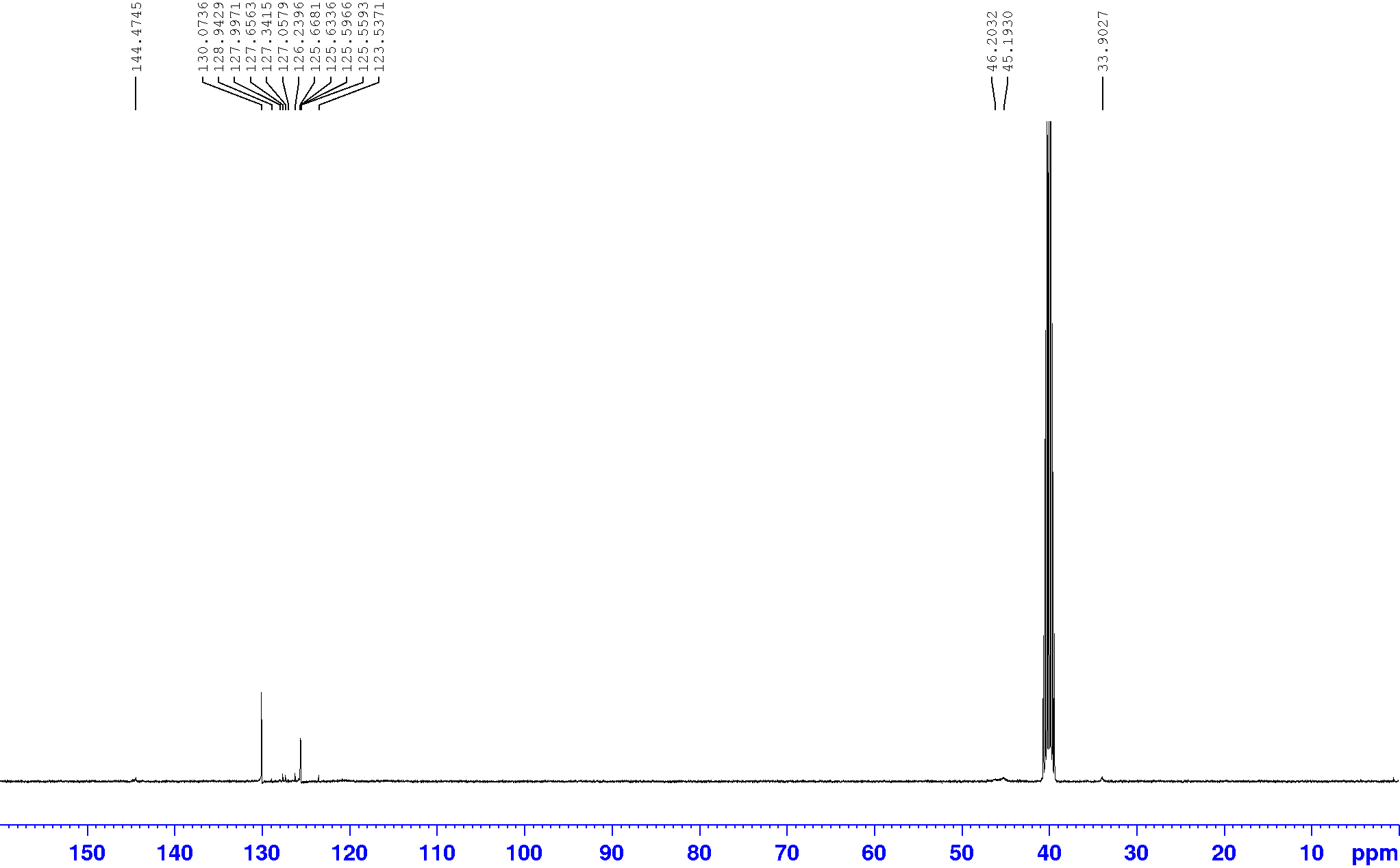


S

S : solvent

W : water

I : impurity

**8b (KDS12008)**

**DMSO-*d_6_***

**CI 1h.** ^1^H and ^13^C NMR spectra of **8c (KDS12025)**


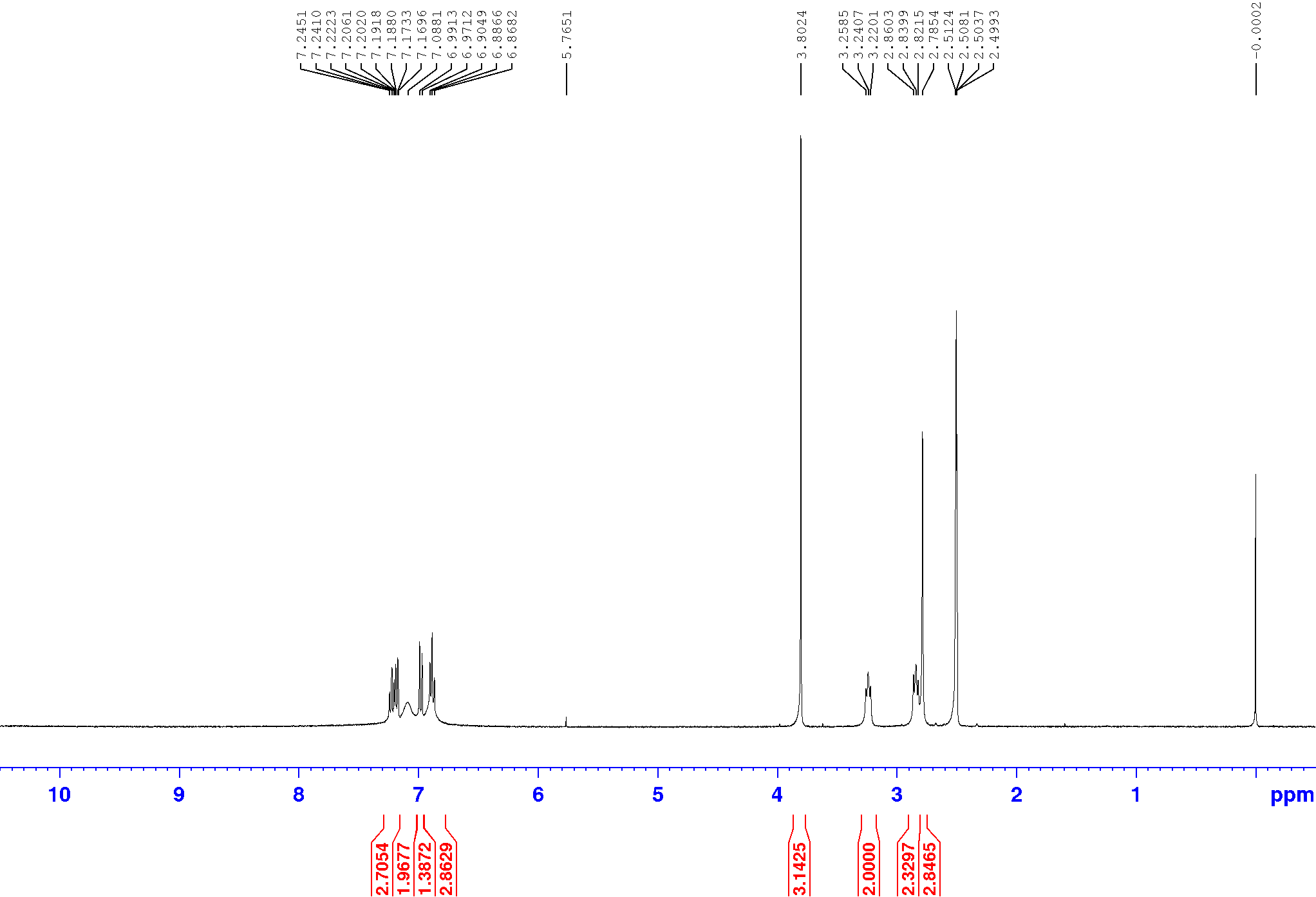


S

S : solvent

W : water

I : impurity

**8c (KDS12025)**

**DMSO-*d_6_***


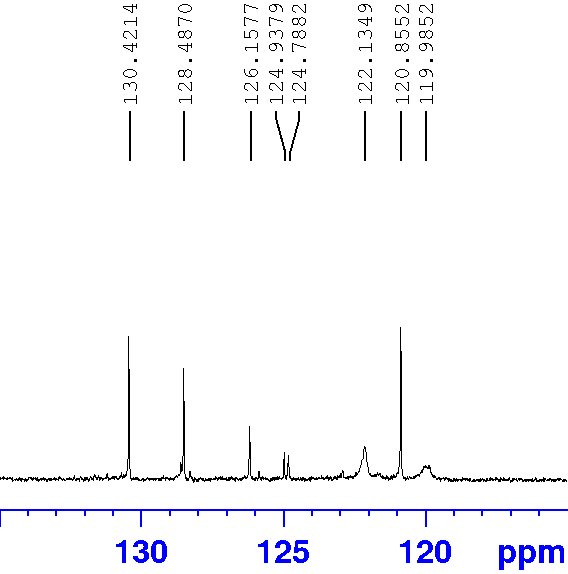

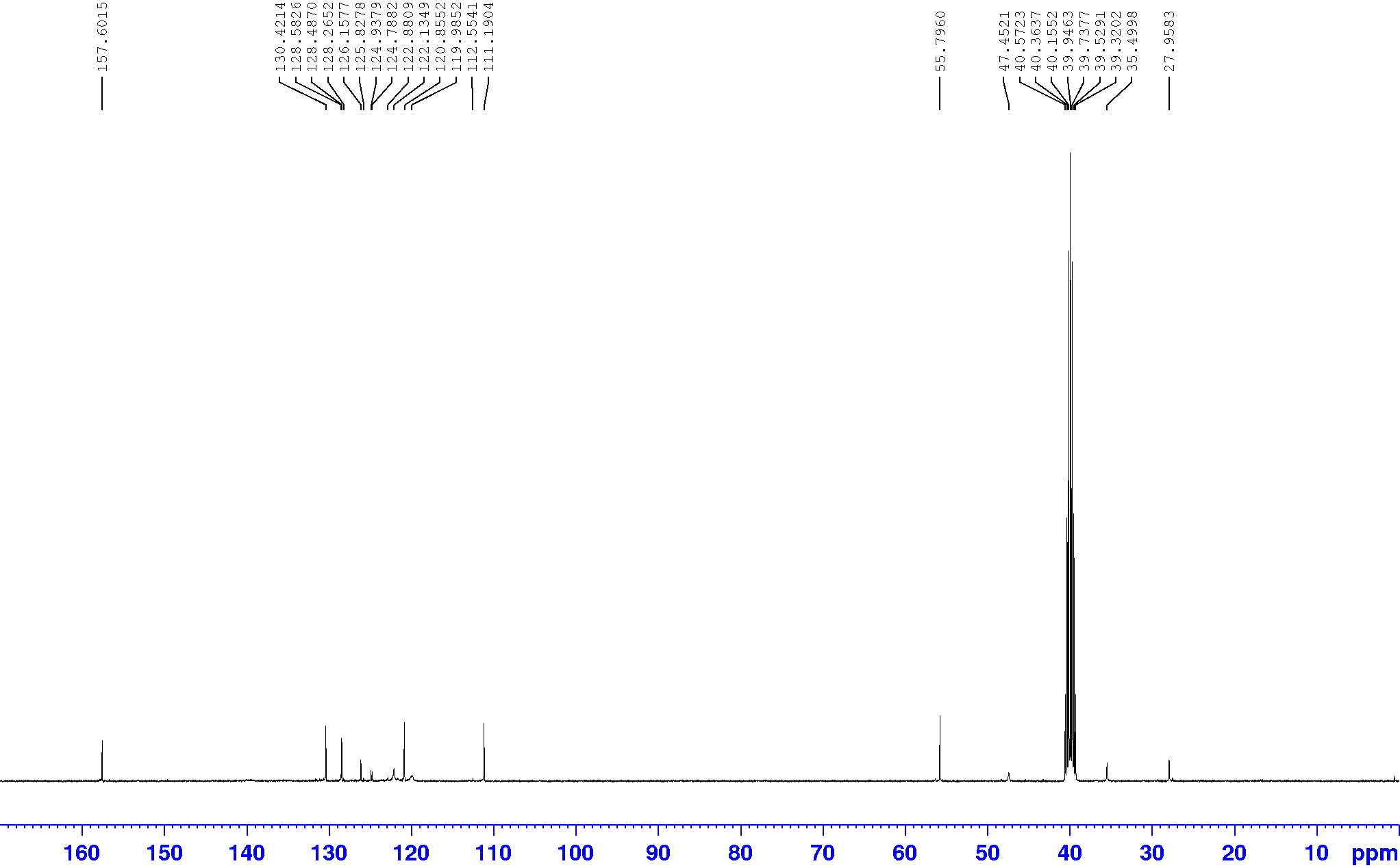


S : solvent

W : water

I : impurity

S

**8c (KDS12025)**

**DMSO-*d_6_***

**CI 1i.** ^1^H and ^13^C NMR spectra of **8d**


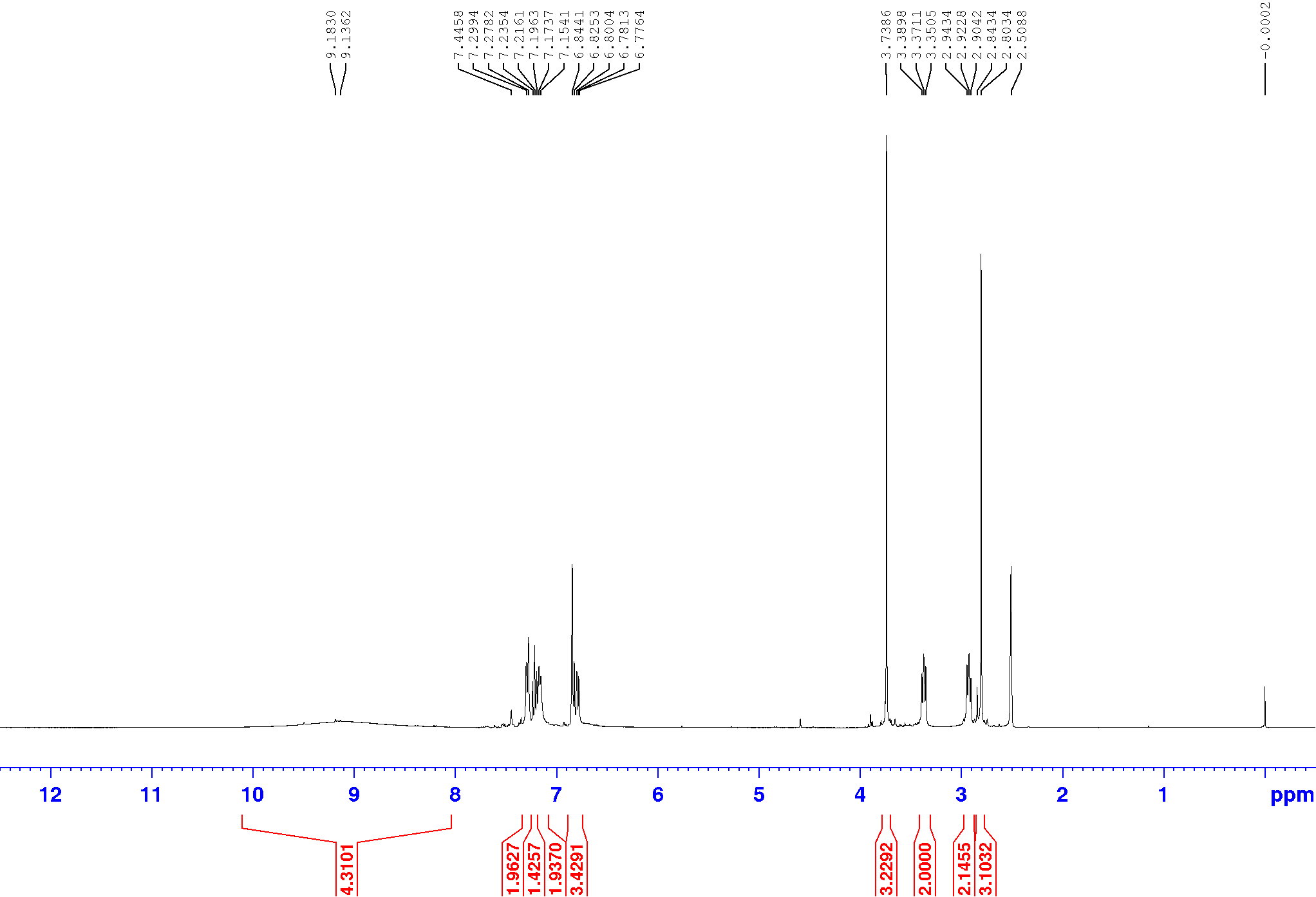


S

S : solvent

W : water

I : impurity

**DMSO-*d_6_***

**8d**


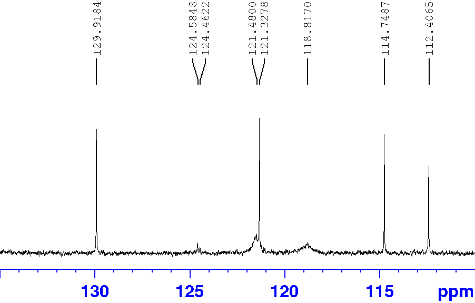

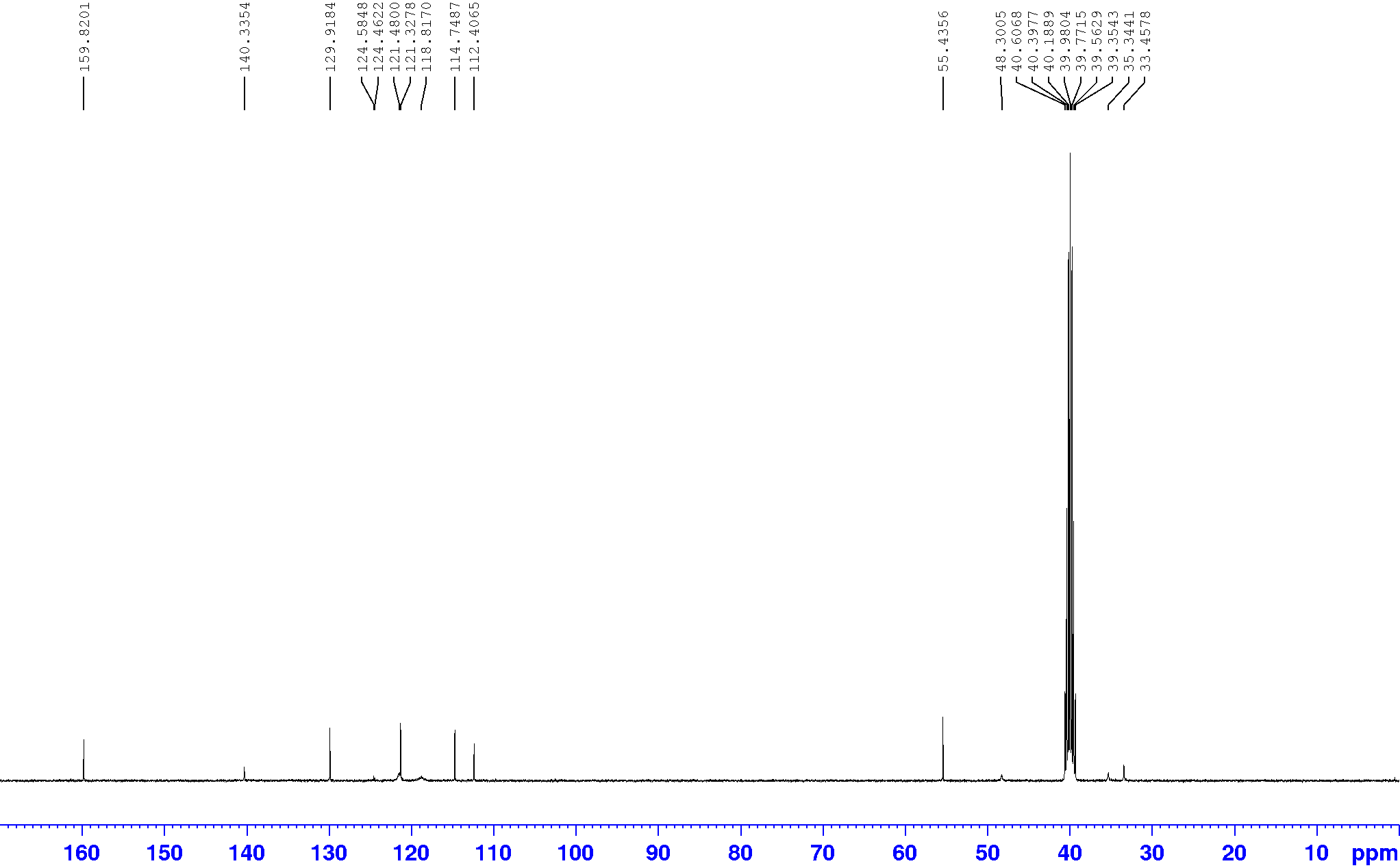


S

S : solvent

W : water

I : impurity

**DMSO-*d_6_***

**8d**

**CI 1j.** ^1^H and ^13^C NMR spectra of **8e**


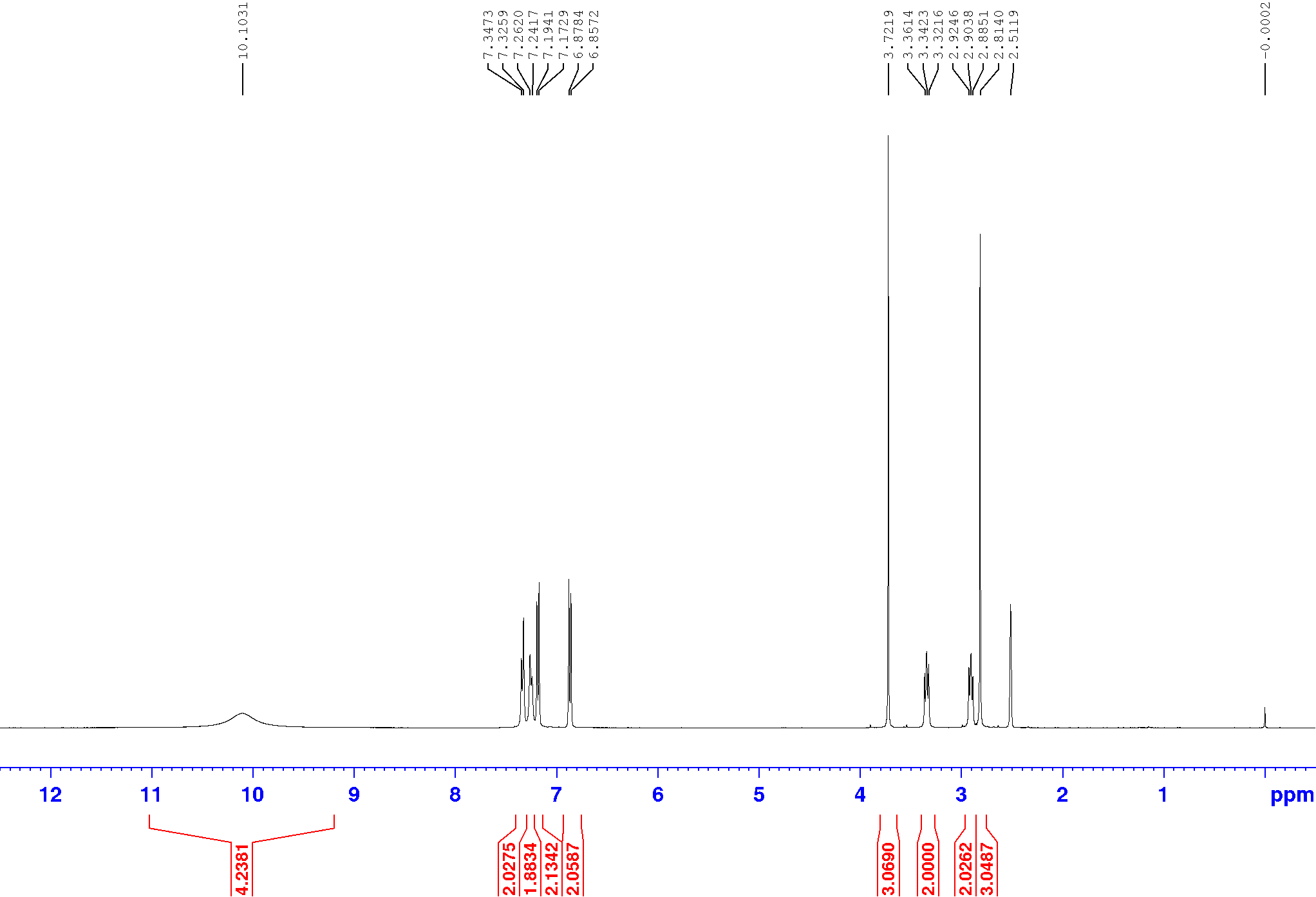


S : solvent

W : water

I : impurity

S

**8e**

**DMSO-*d_6_***


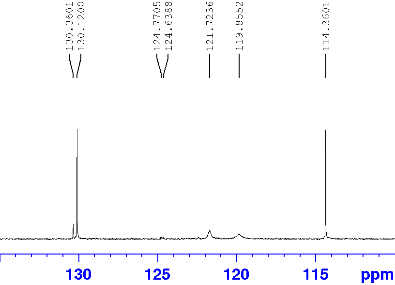

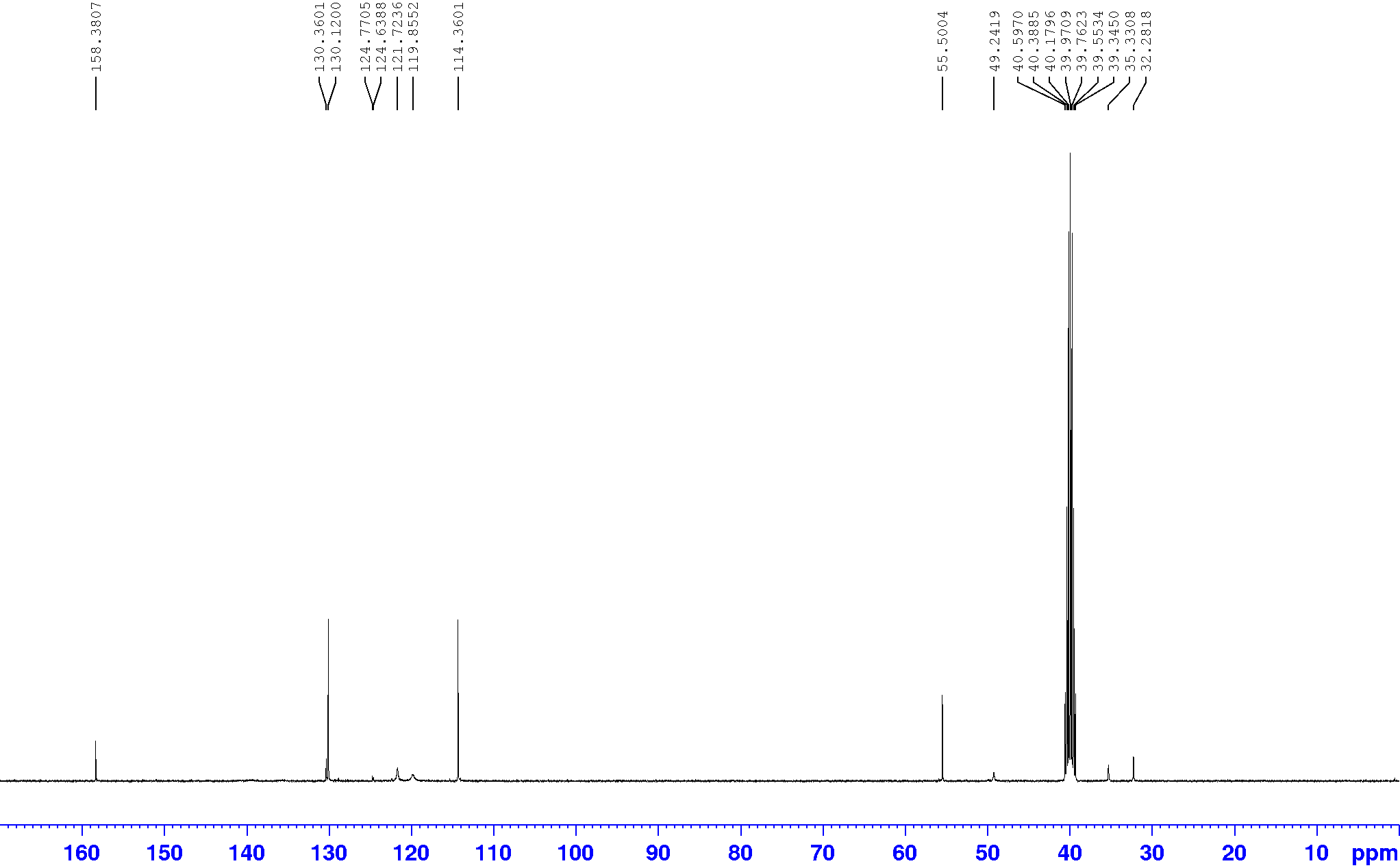


S

S : solvent

W : water

I : impurity

**DMSO-*d_6_***

**8e**

**CI 1k.** ^1^H and ^13^C NMR spectra of **8f**


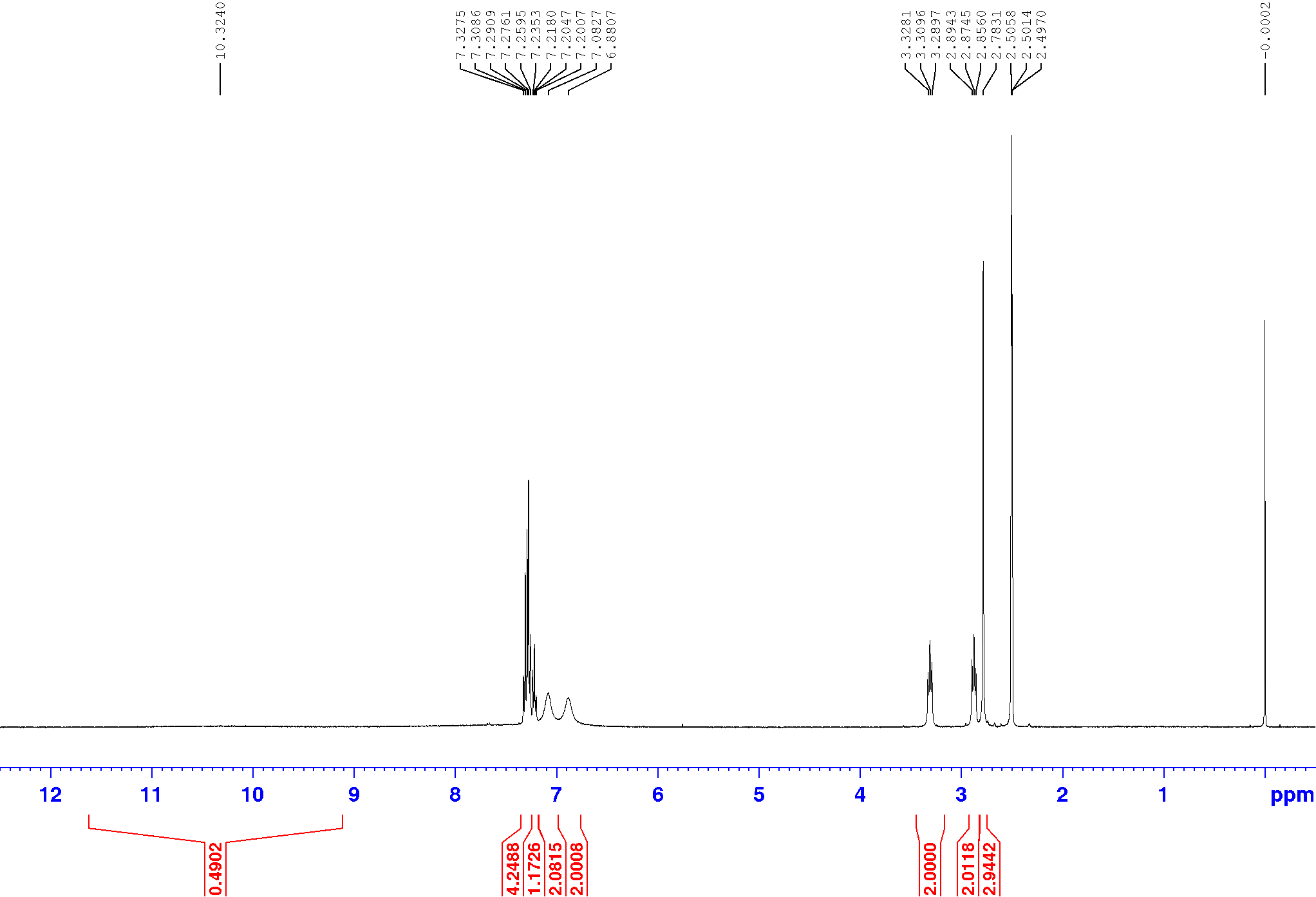


S

S : solvent

W : water

I : impurity

**DMSO-*d_6_***

**8f**


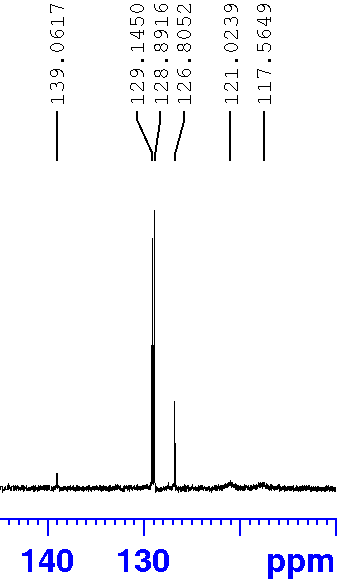

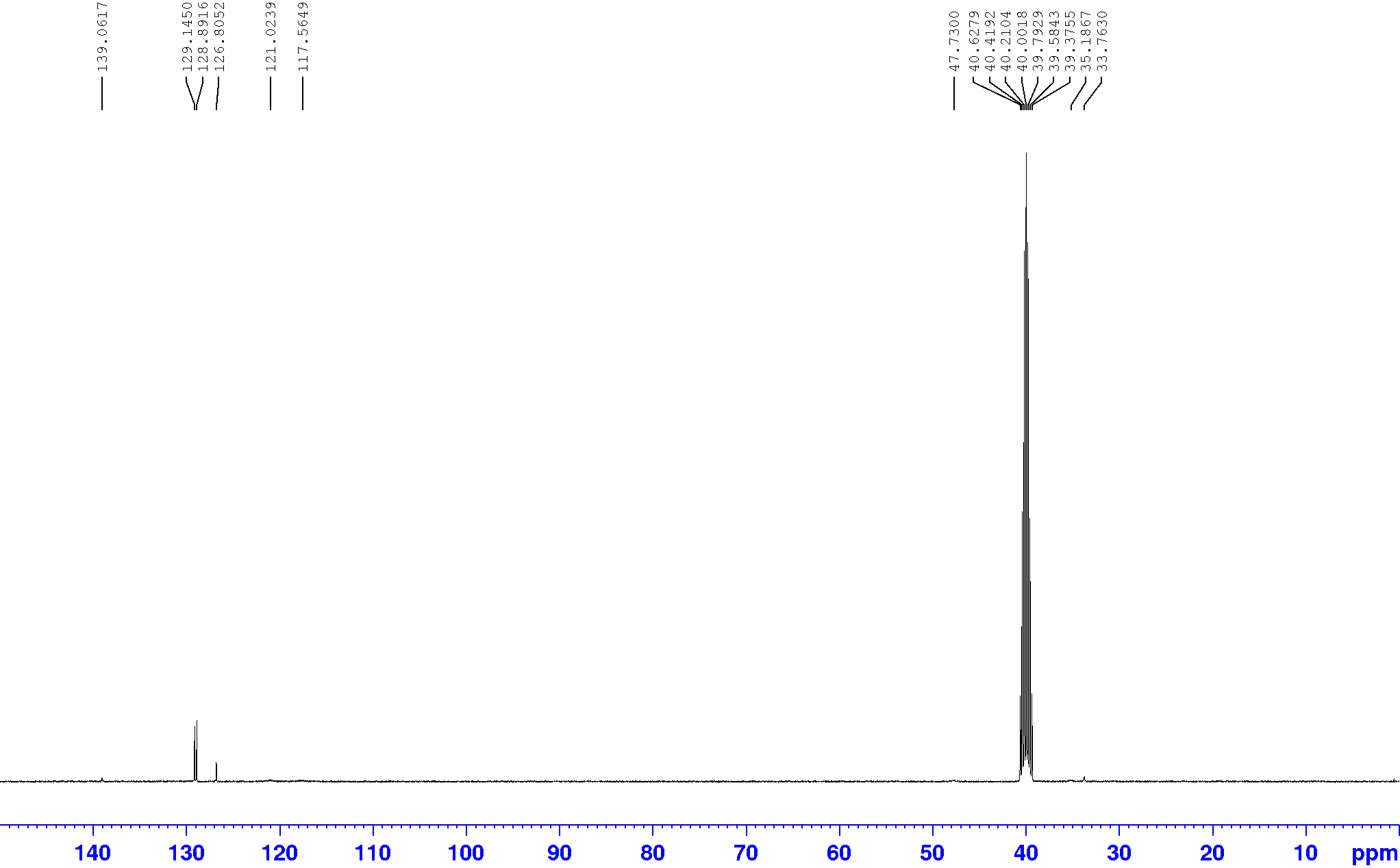


S

S : solvent

W : water

I : impurity

**8f**

**DMSO-*d_6_***

**CI 1l.** ^1^H and ^13^C NMR spectra of **8g**


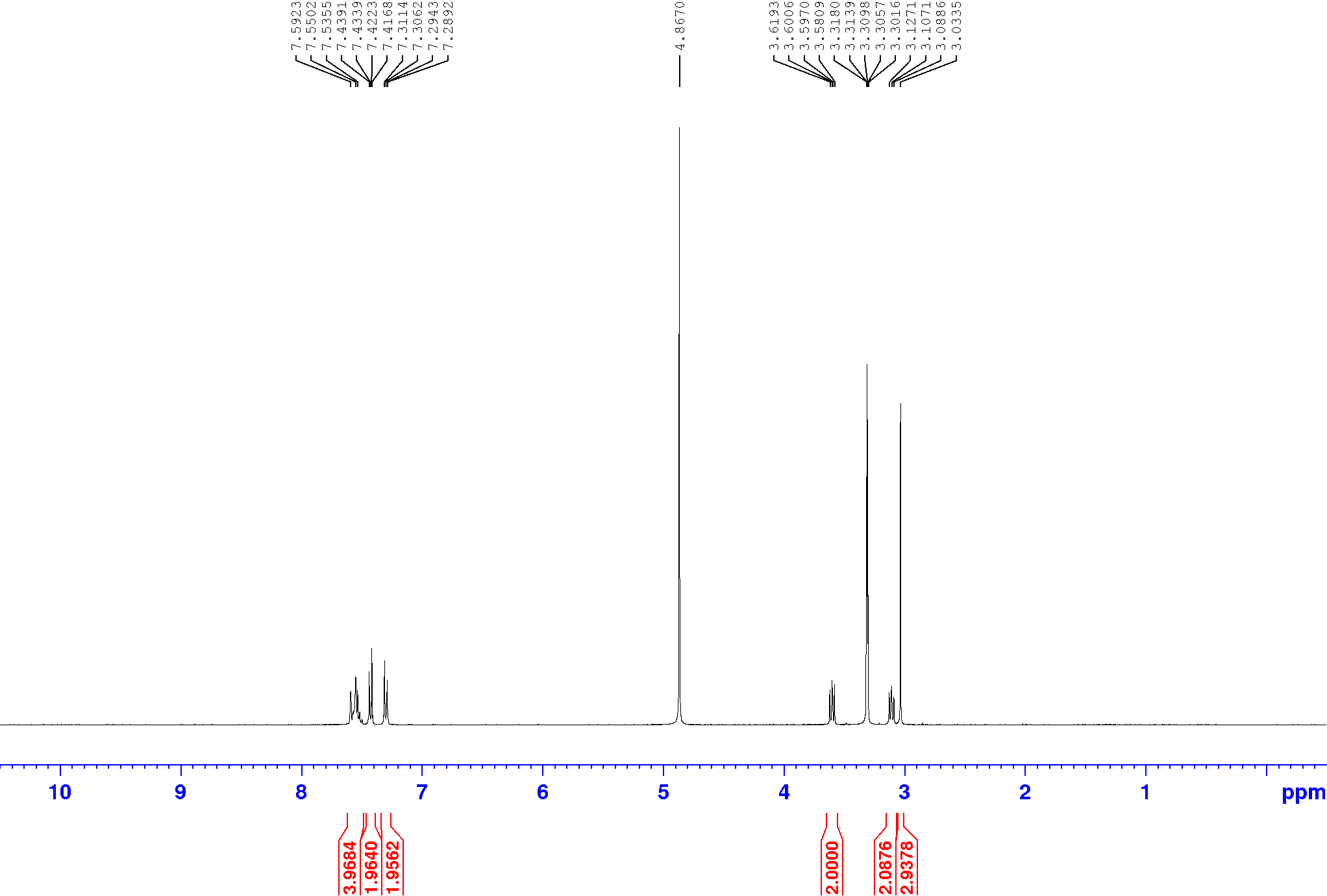


W

S

S : solvent

W : water

I : impurity

**8g**

**MeOD-*d_4_***


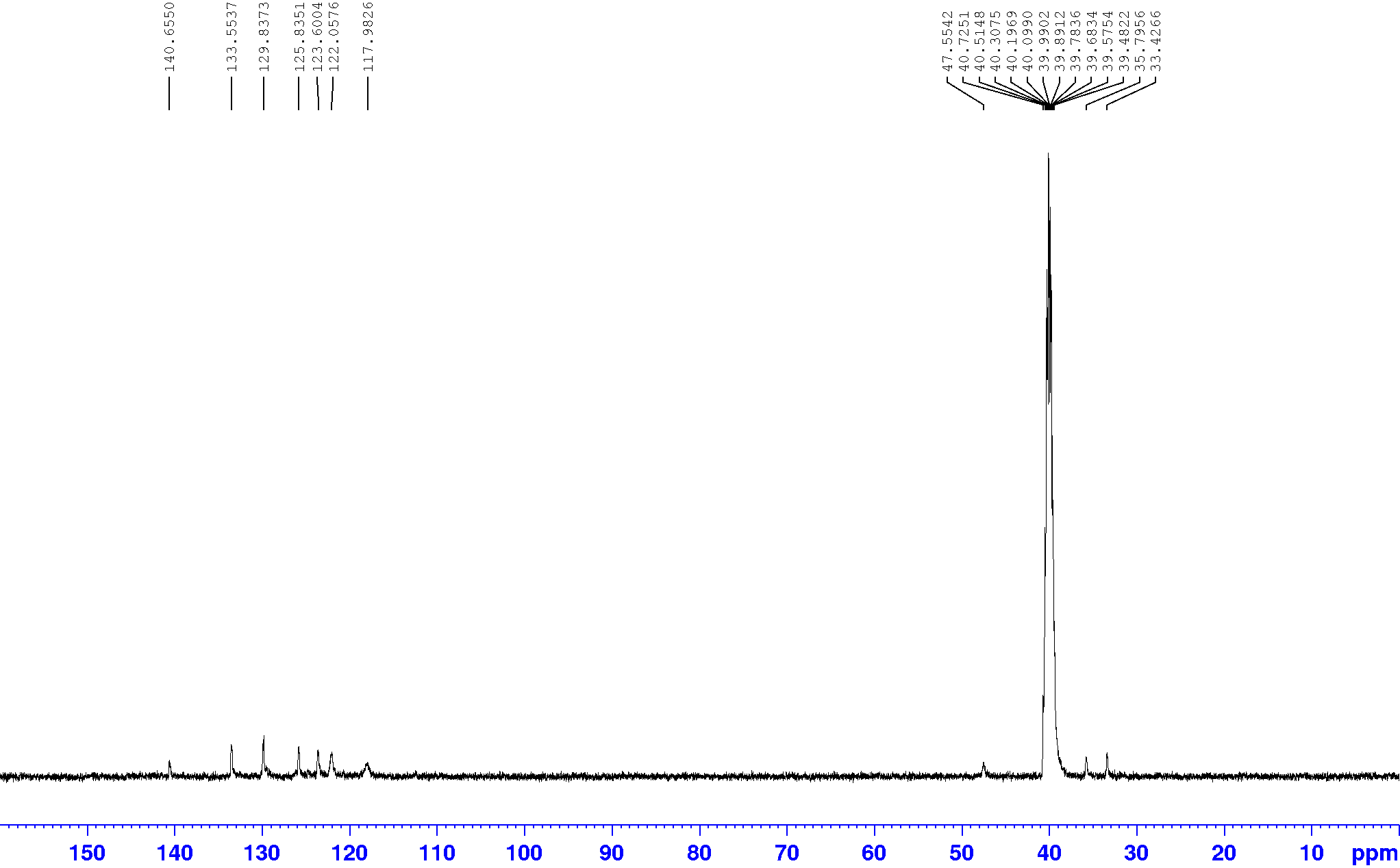


S

S : solvent

W : water

I : impurity

**DMSO-*d_6_***

**8g**

**CI 1m.** ^1^H and ^13^C NMR spectra of 8h


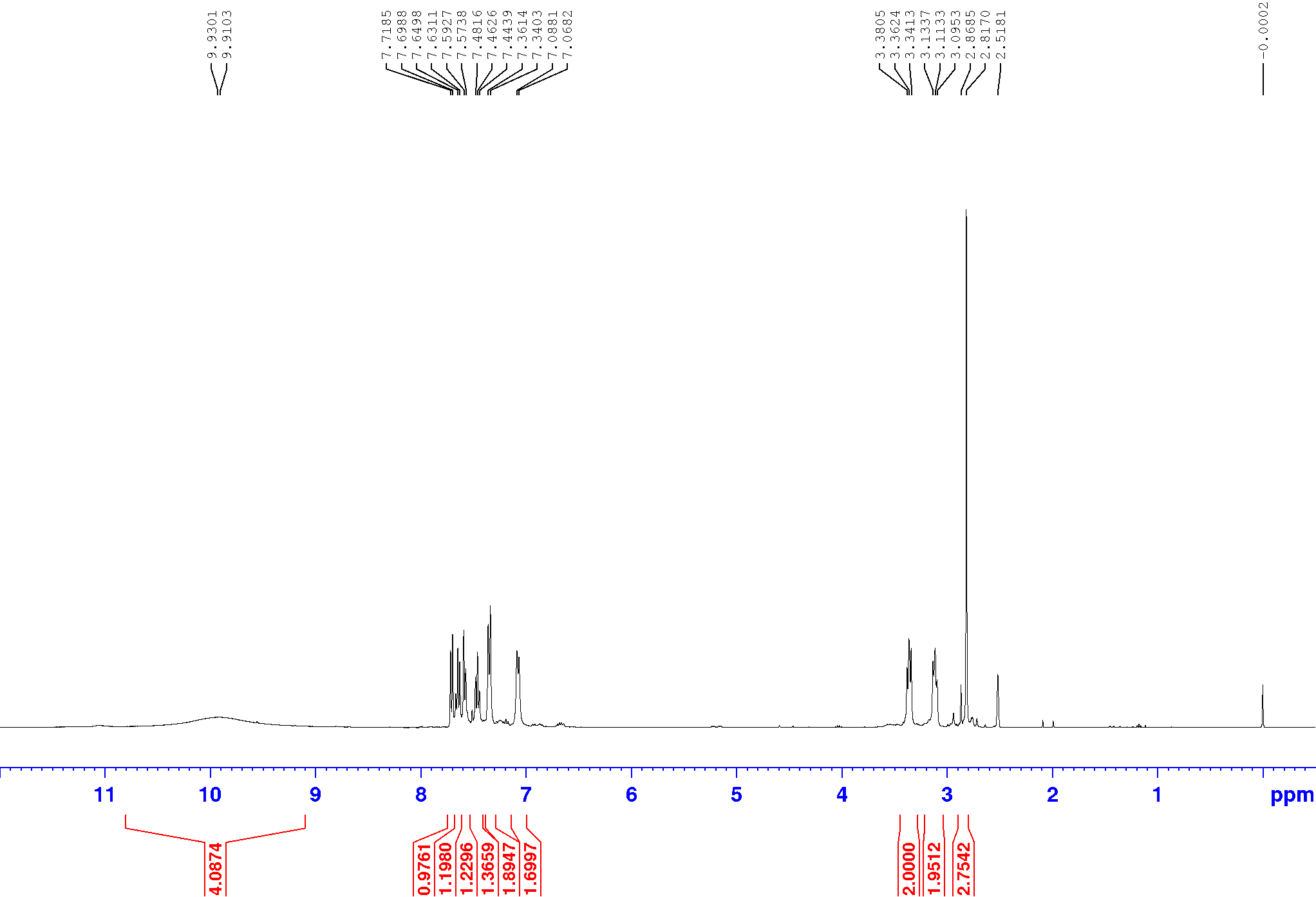


S

S : solvent

W : water

I : impurity

**8h**

**DMSO-*d_6_***


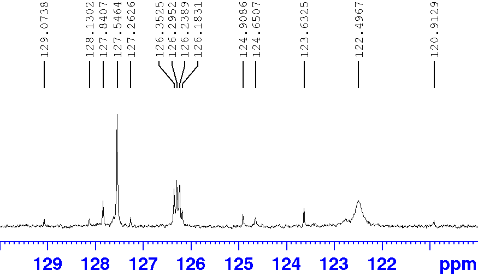

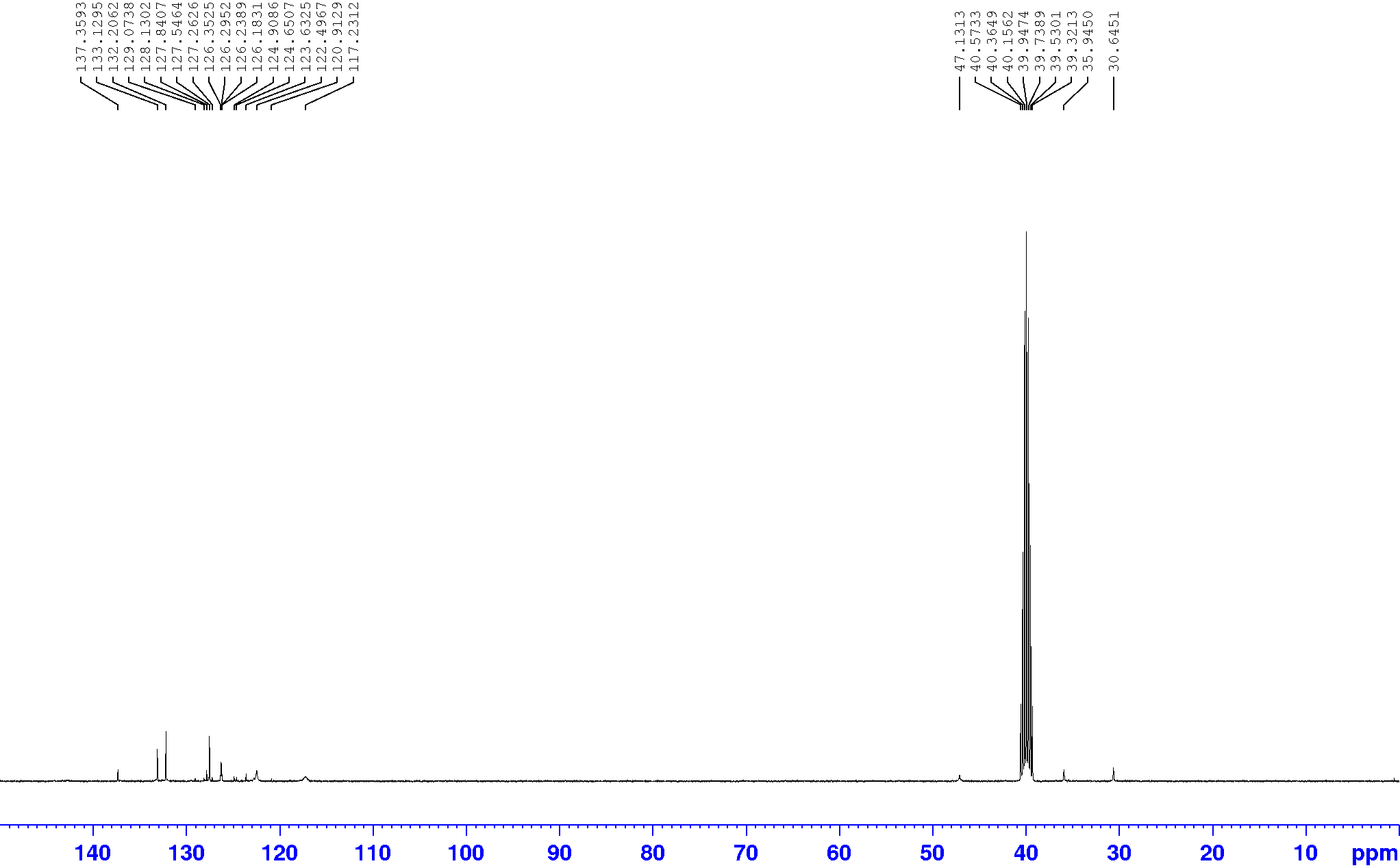


S

S : solvent

W : water

I : impurity

**DMSO-*d_6_***

**8h**

**III. HPLC purities of final compounds**

**CI 2a.** HPLC analayis report of **7b**

**
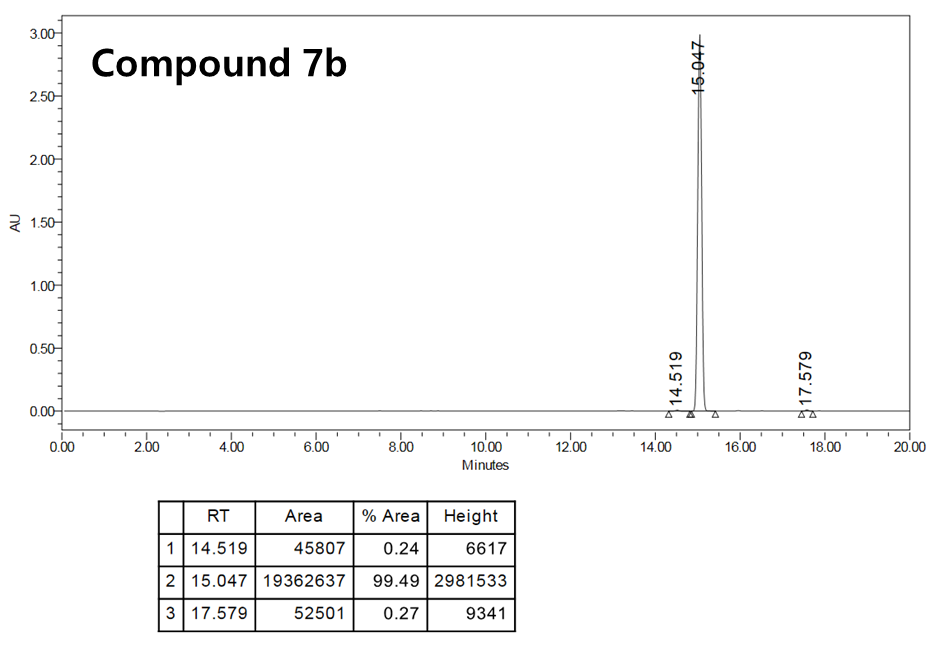
**

**CI 2b.** HPLC analayis report of **7c**

**
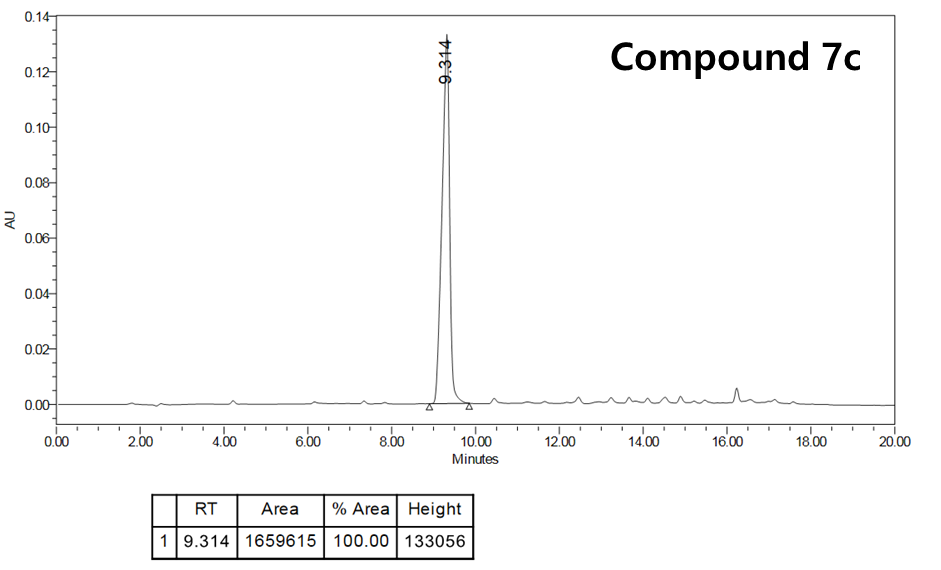
**

**CI 2c.** HPLC analayis report of **7d**

**
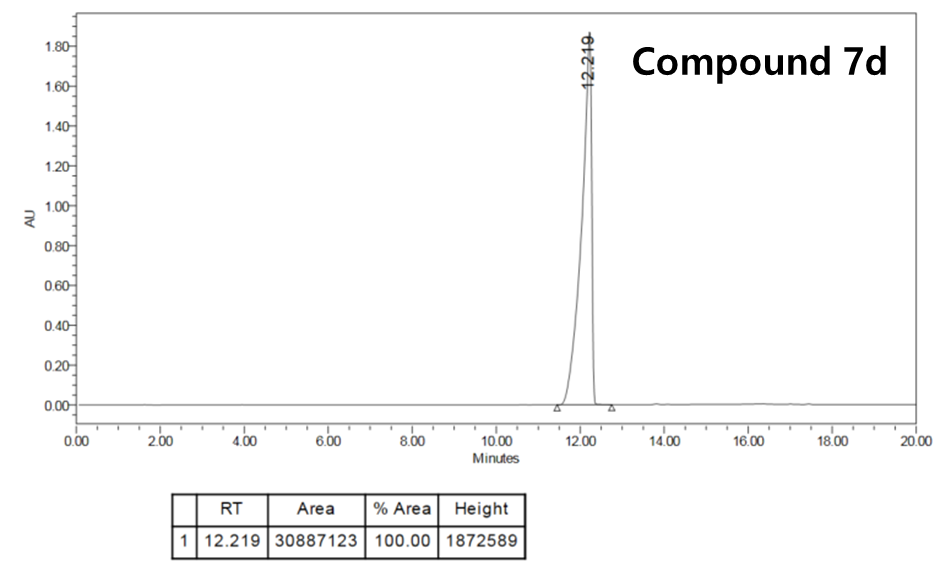
**

**CI 2d.** HPLC analayis report of **7e**

**
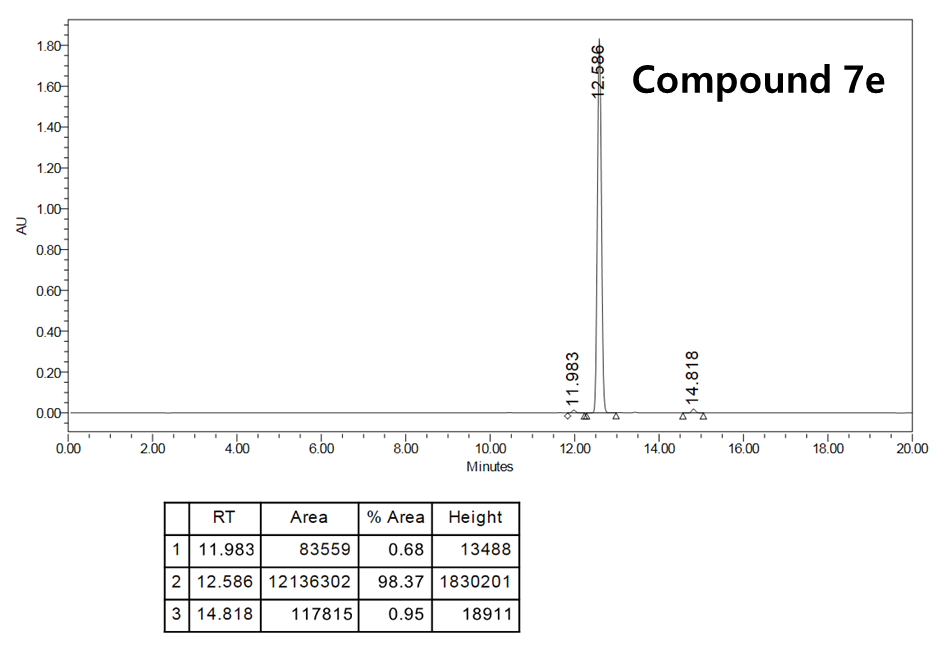
**

**CI 2e.** HPLC analayis report of **7f**

**
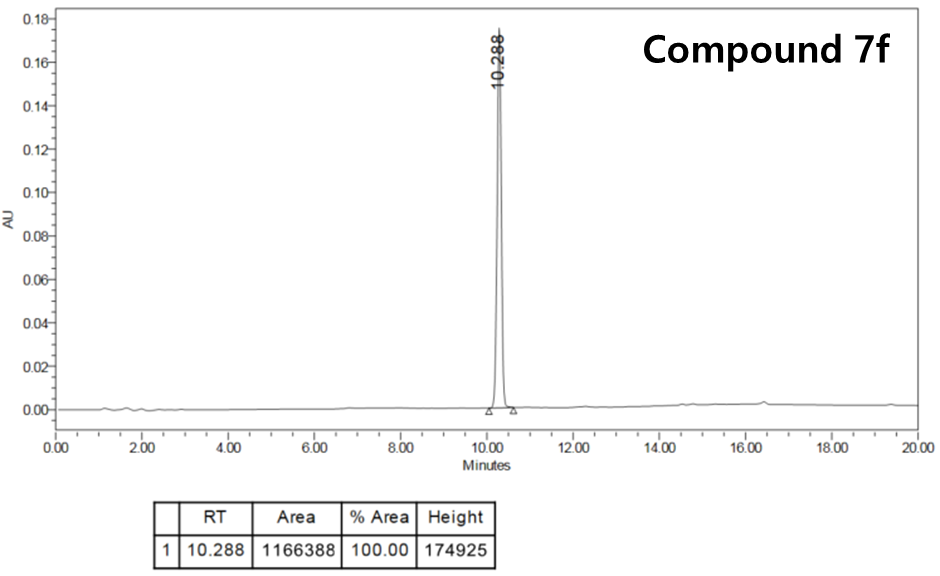
**

**CI 2f.** HPLC analayis report of **8a (KDS12017)**

**
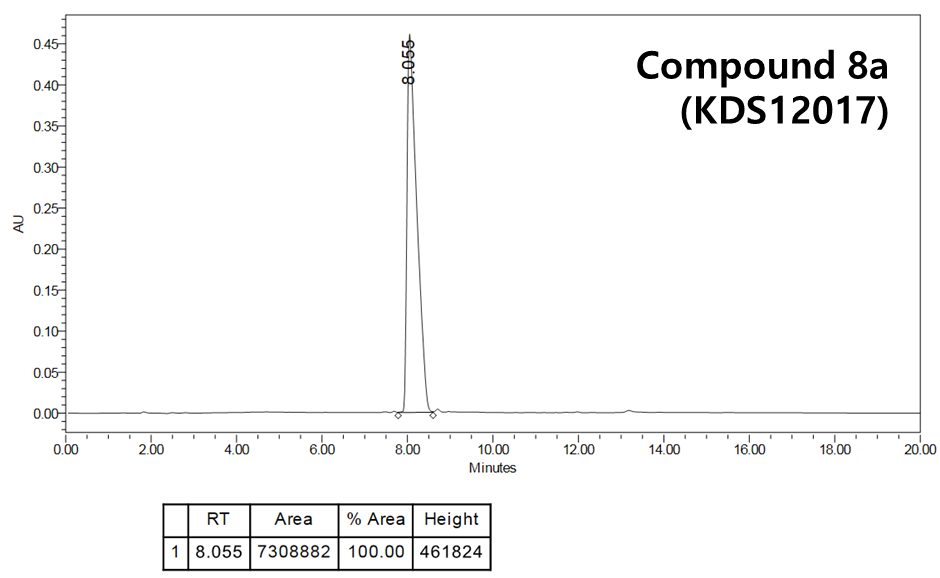
**

**CI 2g.** HPLC analayis report of **8b (KDS12008)
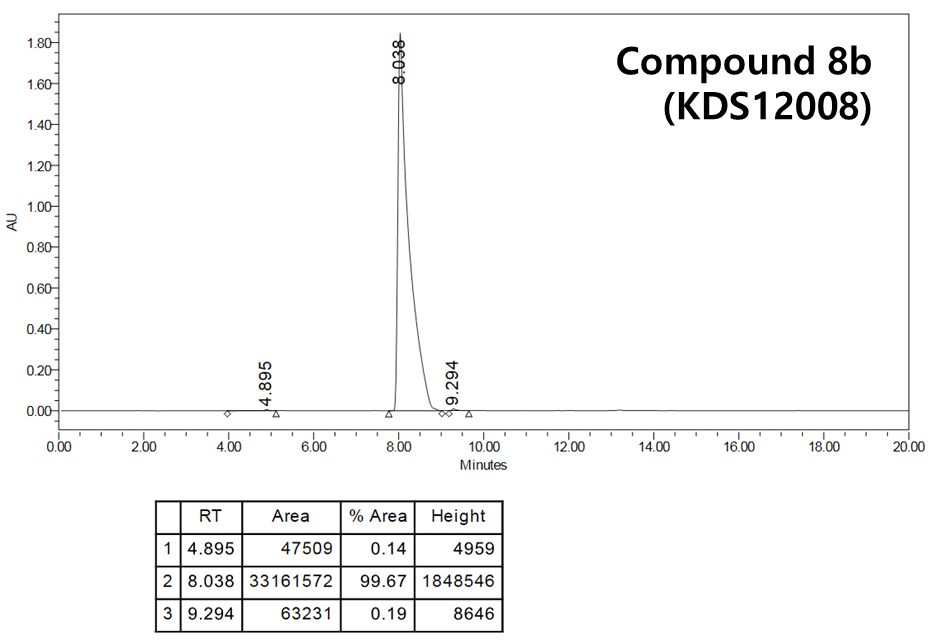
**

**CI 2h.** HPLC analayis report of **8c (KDS12025)**

**
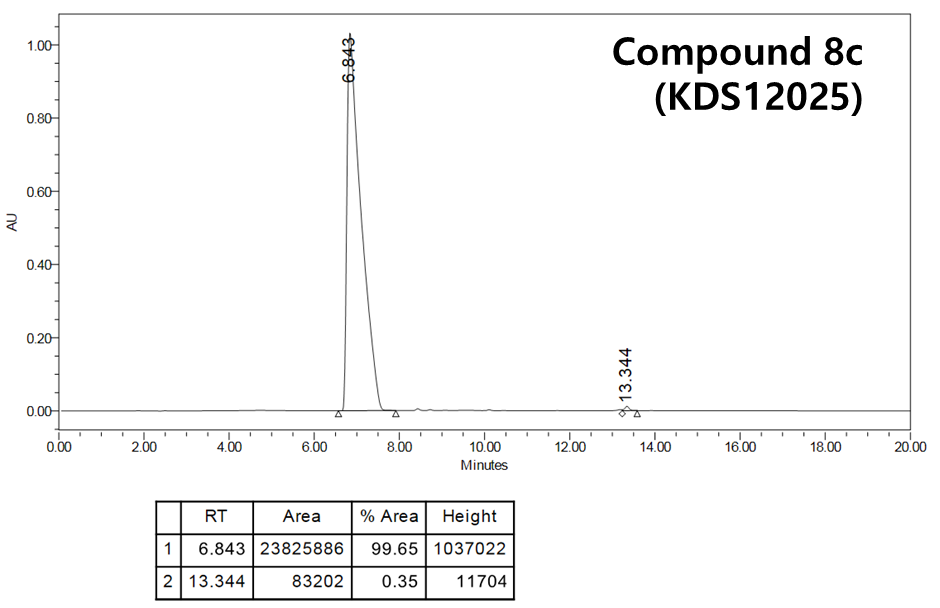
**

**CI 2i.** HPLC analayis report of **8d**

**
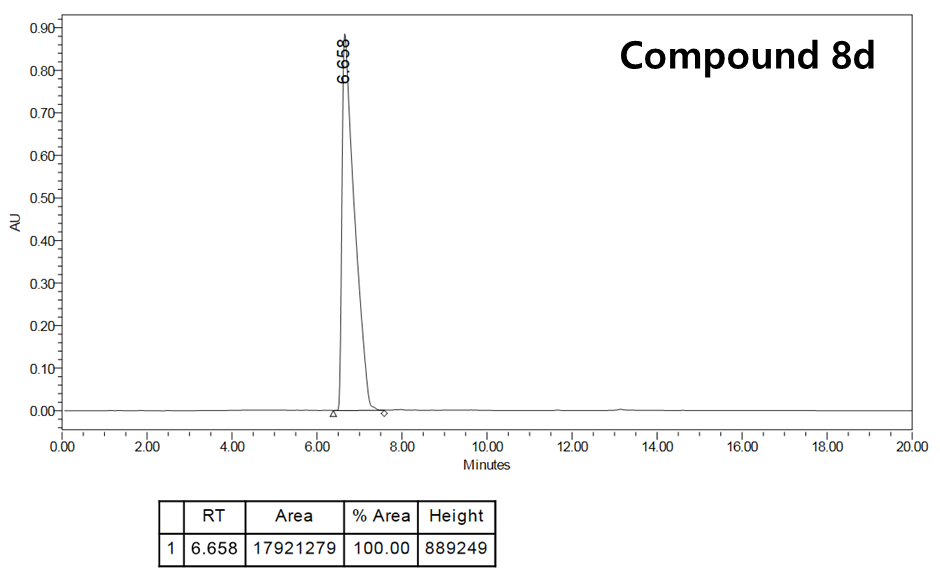
**

**CI 2j.** HPLC analayis report of **8e**

**
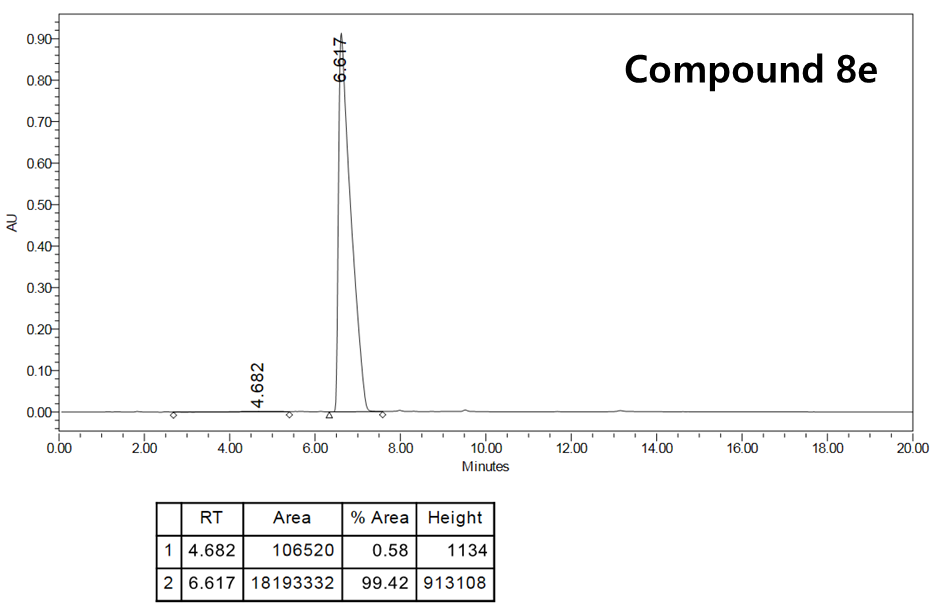
**

**CI 2k.** HPLC analayis report of **8f**

**
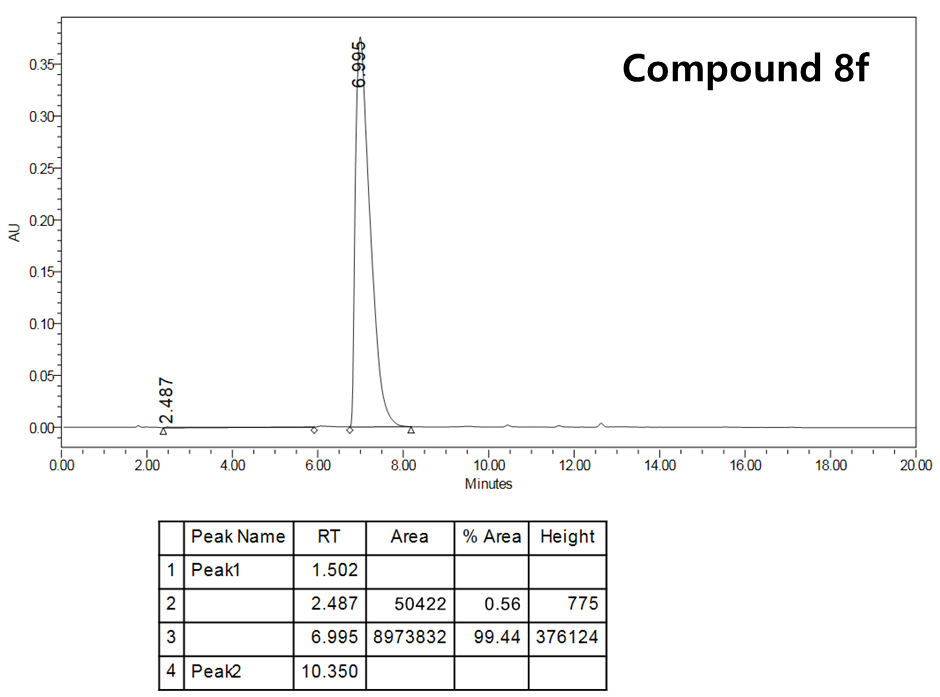
**

**CI 2l.** HPLC analayis report of **8g**

**
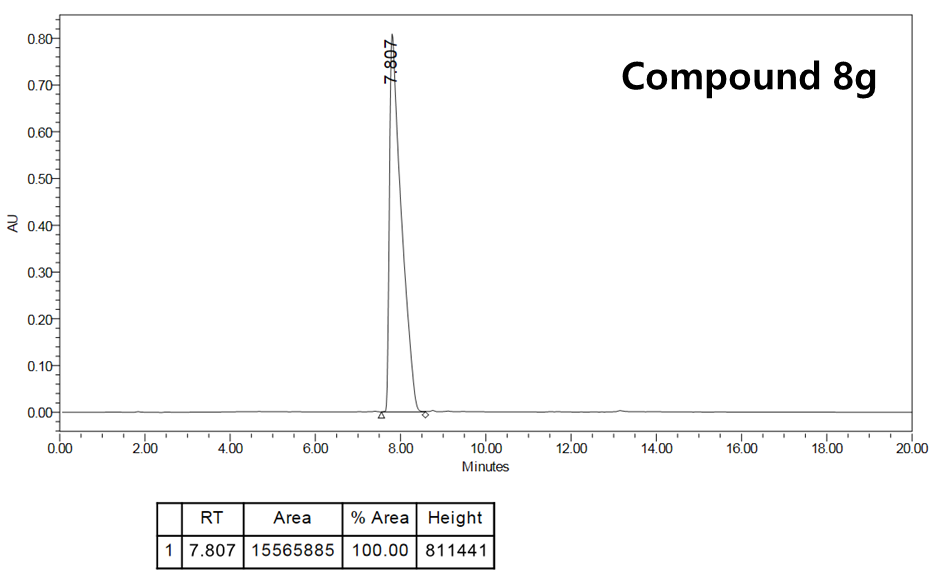
**

**CI 2m.** HPLC analayis report of **8h**

**
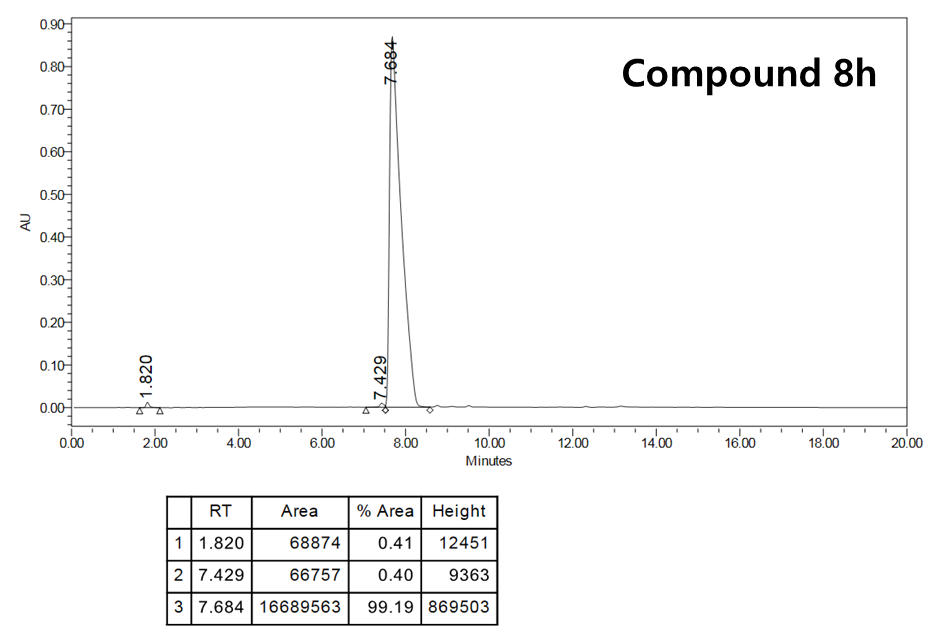
**

**IV. HRMS data of final compounds**

**CI 3a.** HRMS data of **7b**


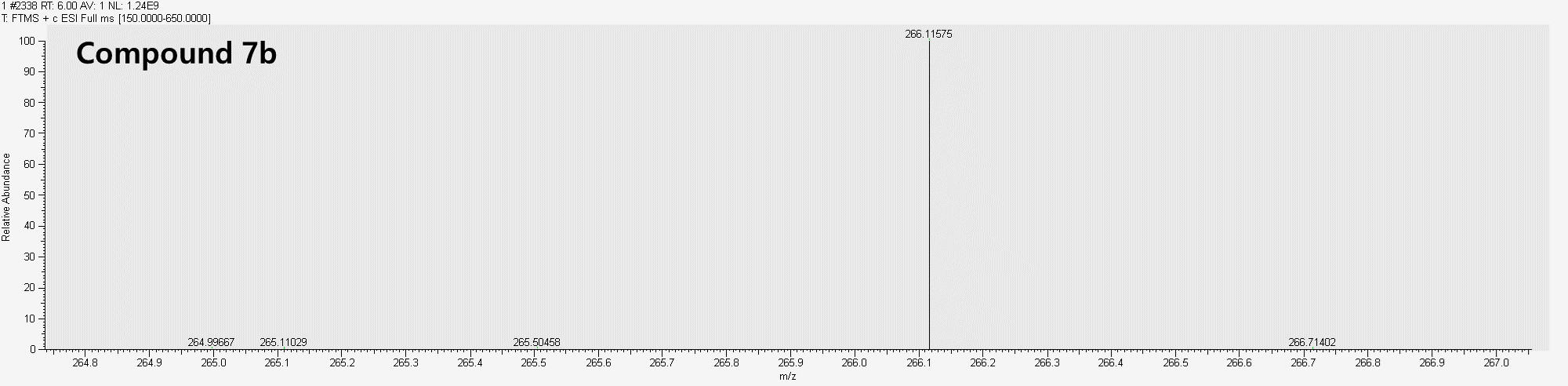


**CI 3b.** HRMS data of **7c**


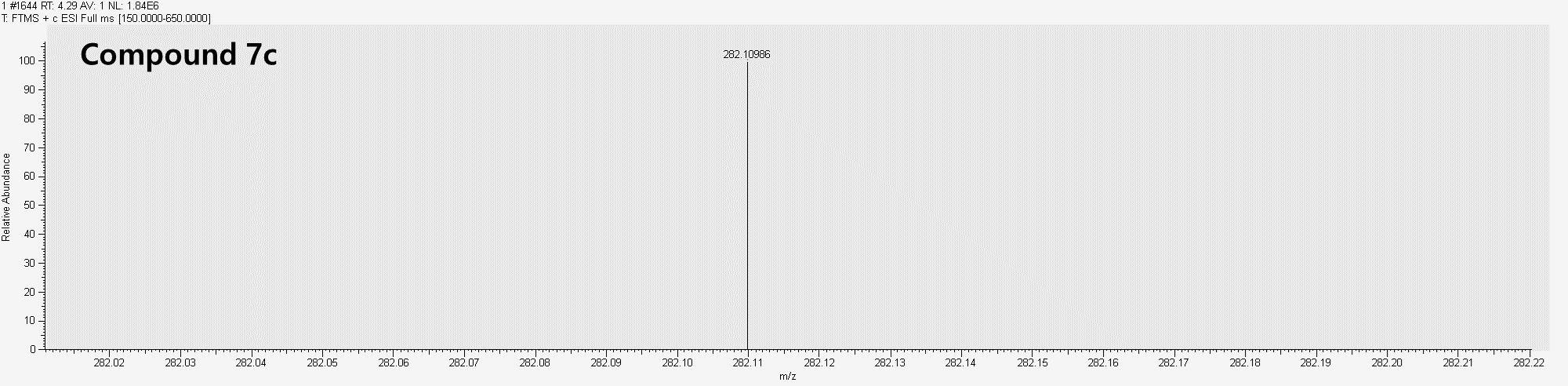


**CI 3c.** HRMS data of **7d**


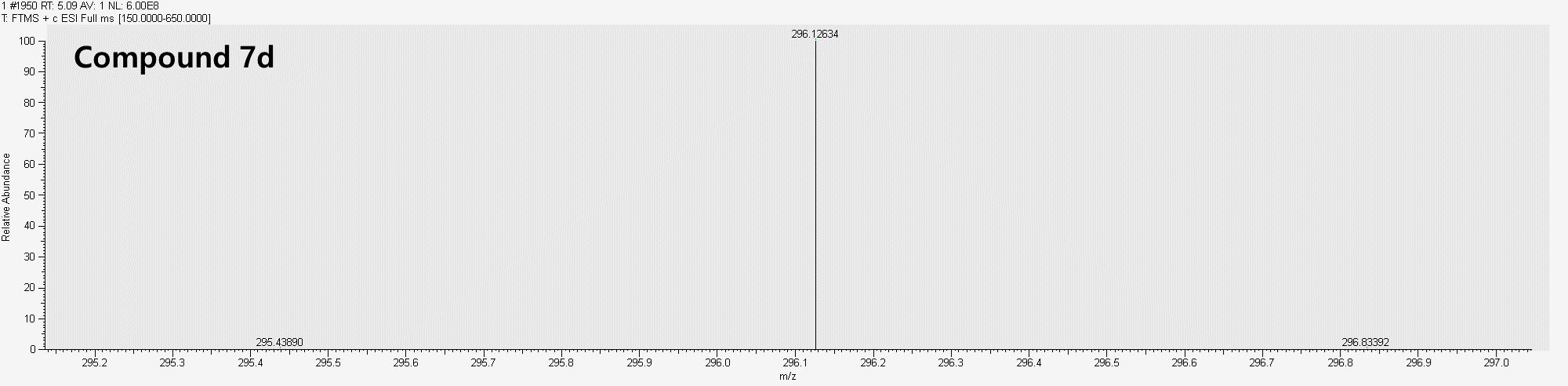


**CI 3d.** HRMS data of **7e**


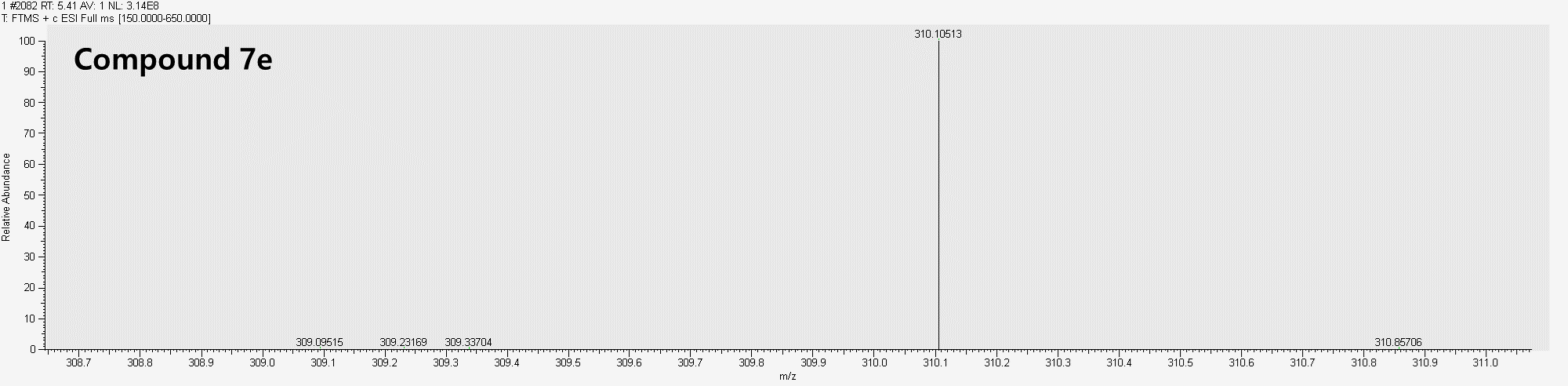


**CI 3e.** HRMS data of **7f**


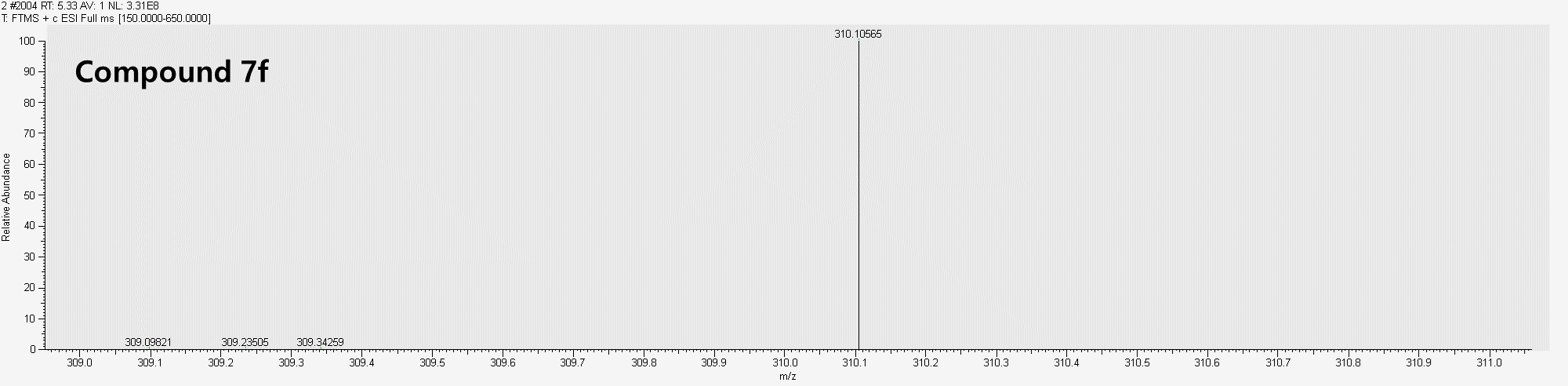


**CI 3f.** HRMS data of **8a (KDS12017)**


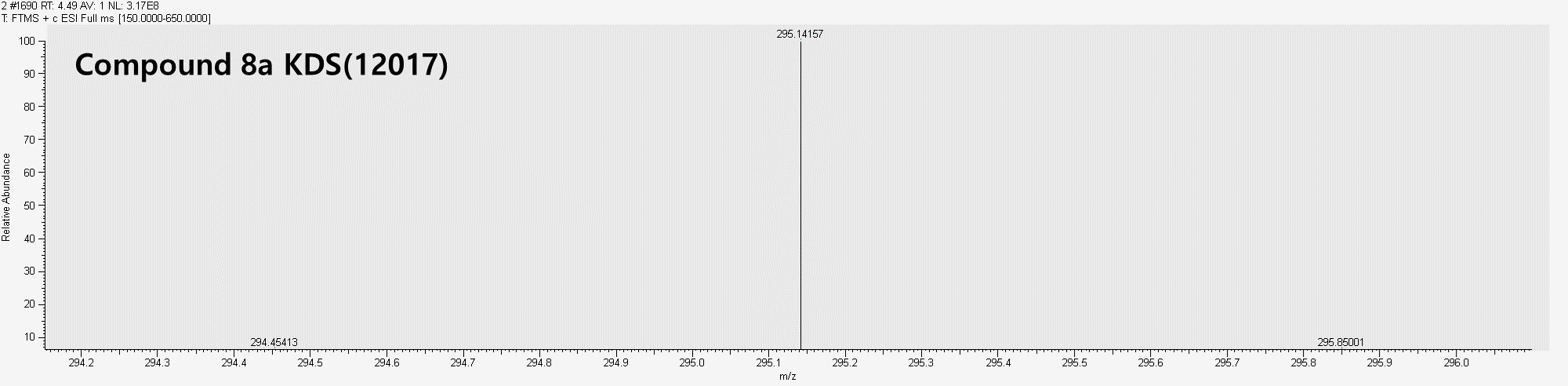


**CI 3g.** HRMS data of **8b (KDS12008)**


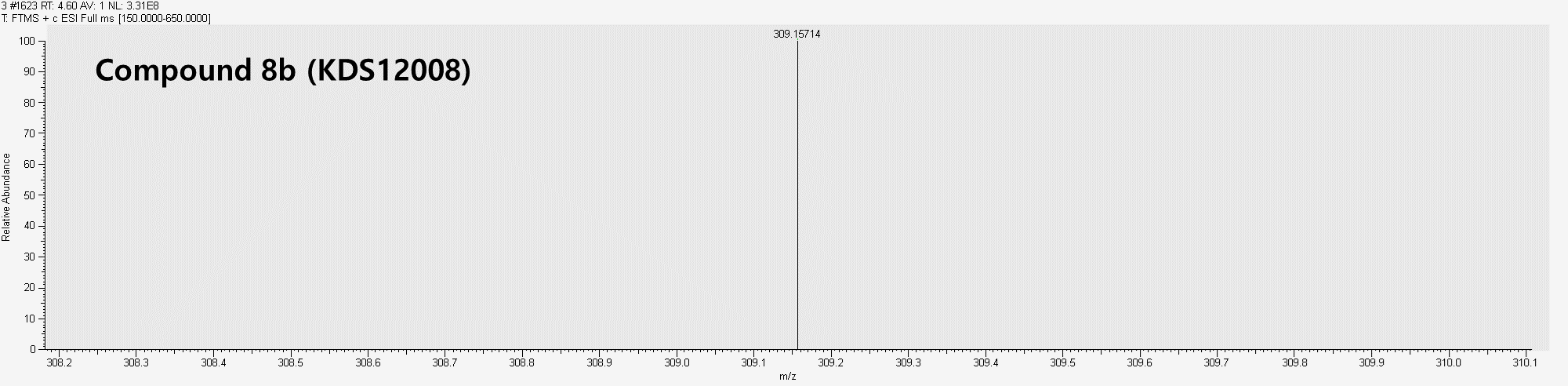


**CI 3h.** HRMS data of **8c (KDS12025)**


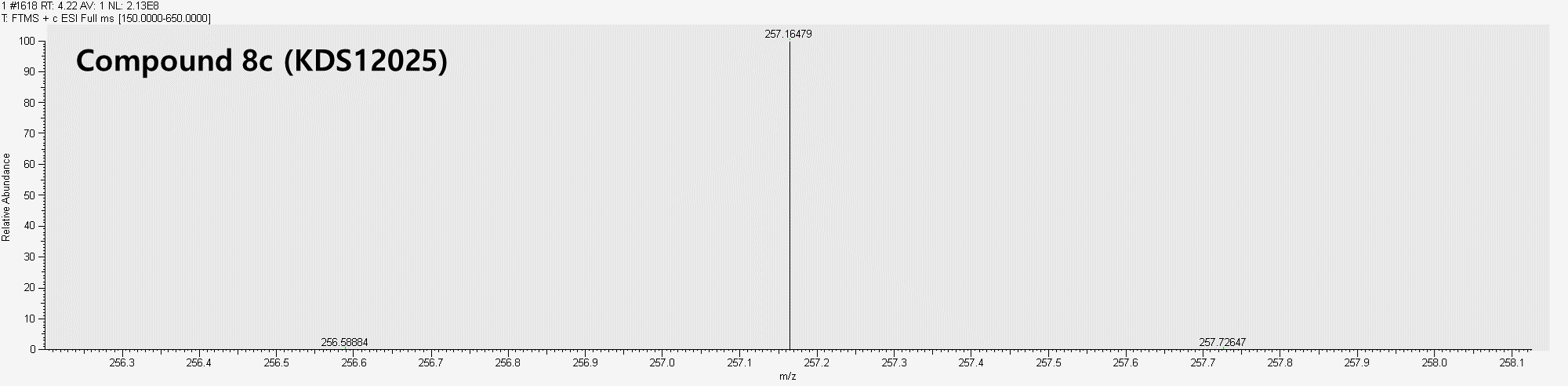


**CI 3i.** HRMS data of **8d**


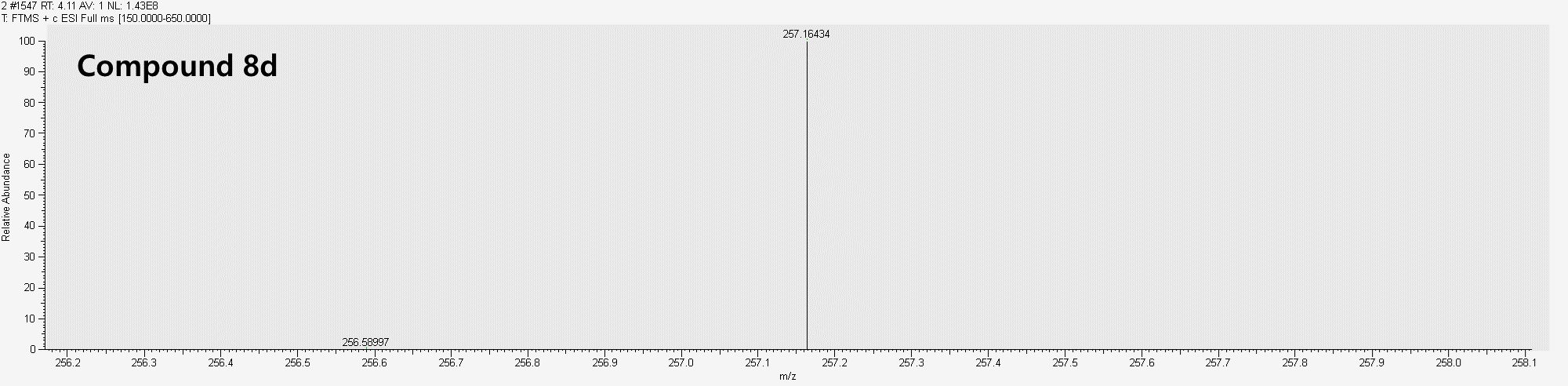


**CI 3j.** HRMS data of **8e**


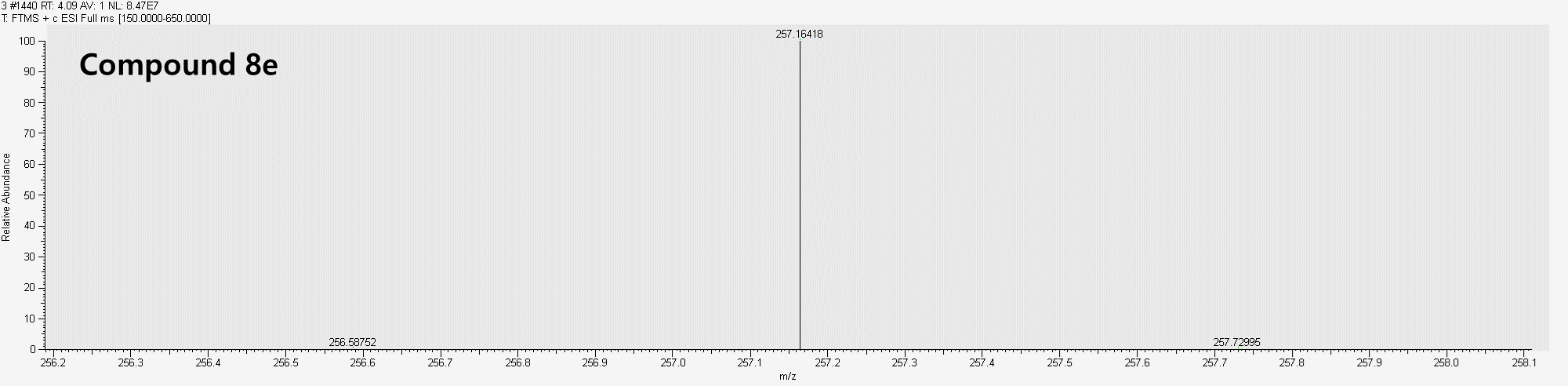


**CI 3k.** HRMS data of **8f**


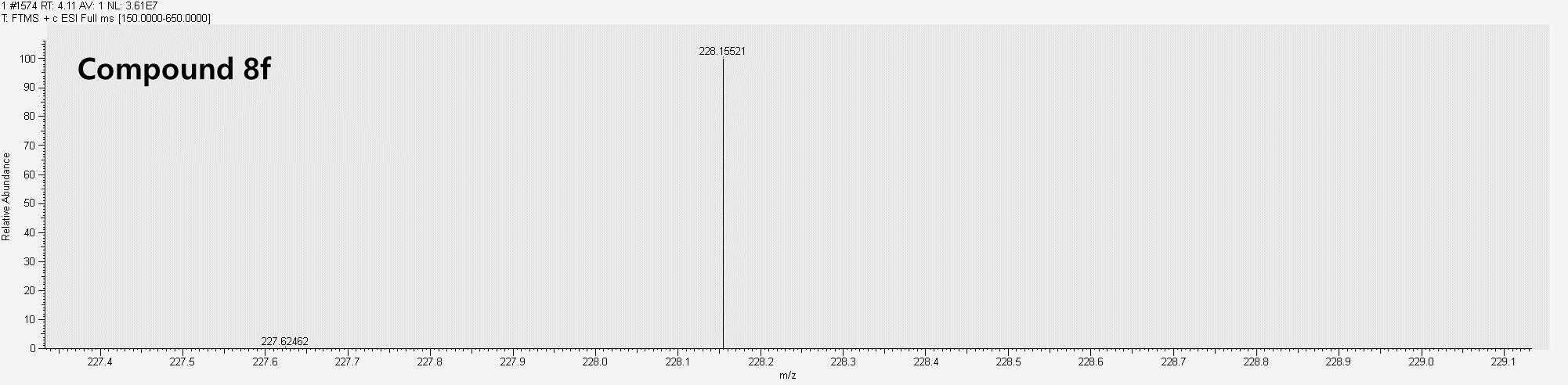


**CI 3l.** HRMS data of **8g**


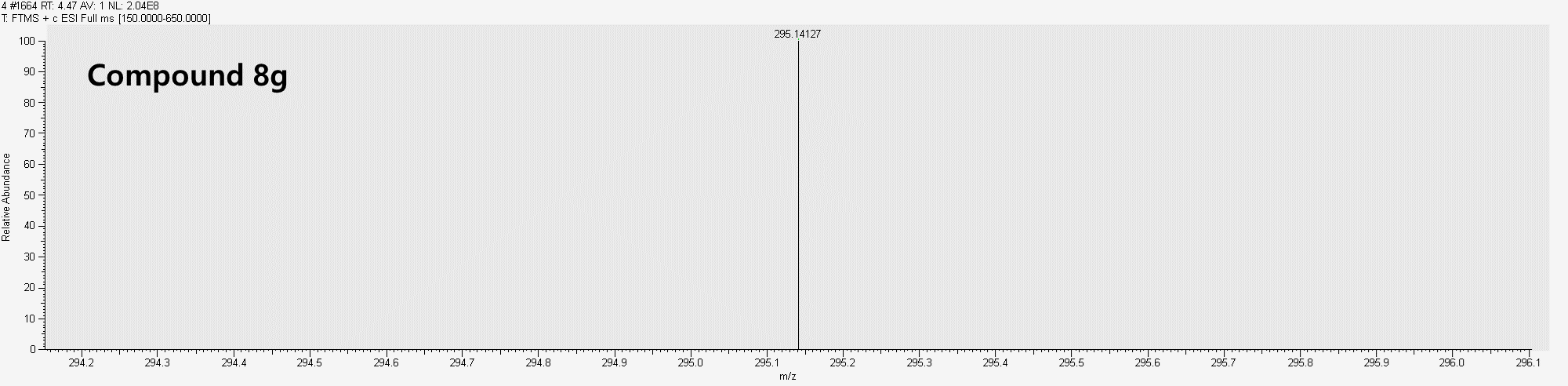


**CI 3m.** HRMS data of **8h**


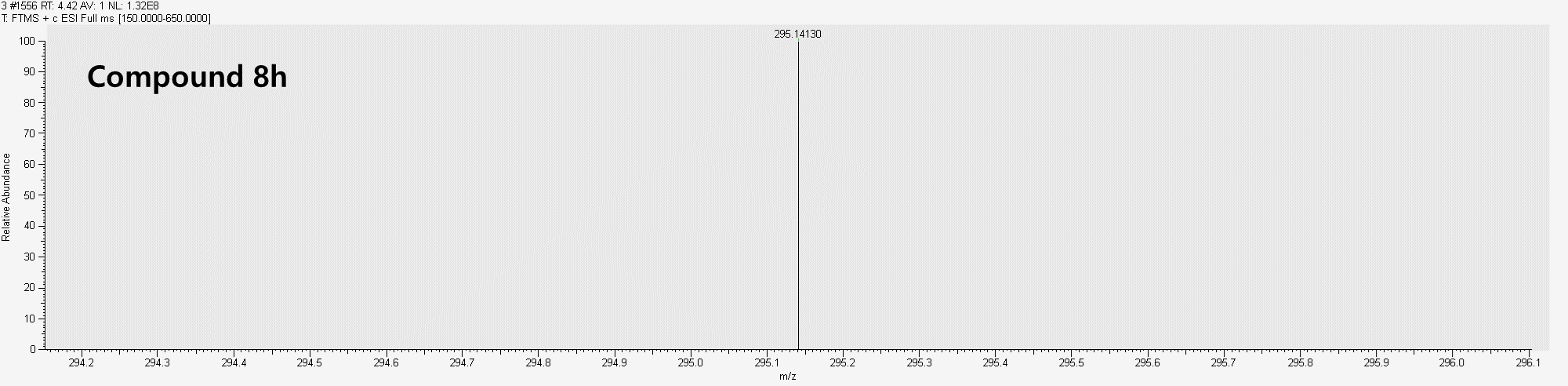

Supplement: Supplementary file 1 — Supplementary Materials [file 41392_2025_2366_MOESM1_ESM.docx]
